# The emergence and development of behavioral individuality in clonal fish

**DOI:** 10.1038/s41467-022-34113-y

**Published:** 2022-10-28

**Authors:** Kate L. Laskowski, David Bierbach, Jolle W. Jolles, Carolina Doran, Max Wolf

**Affiliations:** 1 Department of Biology and Ecology of Fishes, Leibniz Institute of Freshwater Ecology and Inland Fisheries, Berlin, Germany; 2 Department of Evolution and Ecology, University of California Davis, Davis, CA, USA; 3 Faculty of Life Sciences, Albrecht Daniel Thaer-Institute of Agricultural and Horticultural Sciences, Humboldt-Universität zu Berlin, Invalidenstrasse 42, 10115 Berlin, Germany; 4 Cluster of Excellence “Science of Intelligence,” Technical University of Berlin, Berlin, Germany, Marchstr. 23, 10587 Berlin, Germany; 5 Centre for Ecological Research and Forestry Applications (CREAF), Campus UAB. Edifici C., 08193 Bellaterra, Spain

**Keywords:** Behavioural ecology, Animal behaviour

## Abstract

Behavioral individuality is a ubiquitous phenomenon in animal populations, yet the origins and developmental trajectories of individuality, especially very early in life, are still a black box. Using a high-resolution tracking system, we mapped the behavioral trajectories of genetically identical fish (*Poecilia formosa*), separated immediately after birth into identical environments, over the first 10 weeks of their life at 3 s resolution. We find that (i) strong behavioral individuality is present at the very first day after birth, (ii) behavioral differences at day 1 of life predict behavior up to at least 10 weeks later, and (iii) patterns of individuality strengthen gradually over developmental time. Our results establish a null model for how behavioral individuality can develop in the absence of genetic and environmental variation and provide experimental evidence that later-in-life individuality can be strongly shaped by factors pre-dating birth like maternal provisioning, epigenetics and pre-birth developmental stochasticity.

## Abstract (web summary)

You’re unique just like everyone else. But when does such individuality appear? Laskowski et al. find that clonal fish show unique behavioral patterns on their first day of life, and these patterns predict their behavior up to at least 10 weeks later.

## Introduction

For decades, human twin studies have been one of the most influential and powerful paradigms to push the boundaries of our understanding how genetic and environmental factors generate behavioral individuality^1^. In sharp contrast, up to now, little use has been made of the enormous potential of naturally clonal vertebrates as model systems^2,3^. Experiments with naturally clonal vertebrates provide a unique opportunity for a true replicate-individual approach, with the study of a large number of “twins” with naturally occurring genotypes under highly controlled experimental settings, making it possible to tackle some of the most fundamental open questions concerning the mechanisms underlying the emergence and development of individuality early in life.

**Fig. 1:**
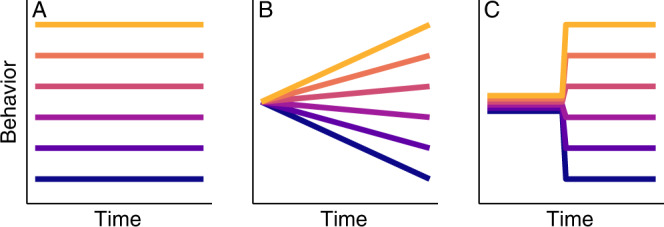
Three stylized scenarios of how individuality can emerge. Individuality might be present at birth (**A**) or emerge gradually (**B**) or in an abrupt fashion (**C**) after birth (see main text for details).

Behavioral individuality is commonly thought to be caused by differences in genes and/or environmental conditions, including the social environment. Challenging this paradigm, there is accumulating evidence that substantial between-individual variation still develops even among genetically identical individuals reared under highly standardized conditions^4–13^. While much of this evidence is based on highly inbred animals (which can limit interpretations about the ecological relevance of the observed variation), and/or experiments that do not (or cannot due to necessary parental care) control for the social environment that individuals experience after birth, in a recent study, we have shown that naturally clonal fish (the Amazon molly, *Poecilia formosa*), separated on day one of their life into identical environments, still exhibited behavioral individuality at seven weeks of age^14^. To determine the causes and mechanisms that can generate behavioral individuality in the absence of genetic and environmental differences, it is essential to first pinpoint when behavioral individuality emerges and how it continues to unfold after emergence. Birth marks a critical time point: if individuality is present at birth, this points to pre-birth influences––such as epigenetics^15,16^, maternal effects^17,18^, and/or pre-birth developmental stochasticity^19,20^––as being key drivers of individuality (Fig. 1A). Alternatively, it could be that individuality primarily emerges after birth. This emergence could happen both gradually throughout early life, which would suggest that individuality is driven by positive feedbacks between behavior and the internal and/or external environment^21,22^ (Fig. 1B), or abruptly at particular points early in life, if it is linked to critical sensitive windows^23,24^ (Fig. 1C).

Here we perform an experimental “twin study” that was designed to distinguish between these scenarios. Specifically, using naturally clonal fish, the Amazon molly, we aim to (i) pinpoint the onset of behavioral individuality during post-birth ontogeny, (ii) continuously map its further change during early-life development and (iii) investigate the link between early-life individuality and later-in-life behavior, thereby also generating a null model for how behavioral individuality can develop in the absence of genetic and environmental variation. To do so, we separate genetically identical individuals directly after birth into identical environments and then track their behavior at 3 s resolution for the first 70 days of their lives using a custom-built recording system^25,26^. Importantly, unlike many other vertebrate model systems, the Amazon molly requires no parental care, thus enabling us to separate the experimental animals (*N* = 26) immediately after birth and allowing near complete experimental control over the environment the animal experiences during their early life.

Experimental fish were reared in highly standardized conditions, and housed in identical individual tanks under controlled light and temperature conditions and with a tightly controlled food regime. To limit maternal variation, all experimental fish were born to mothers (*N*_*moth*_ = 8, Supplementary Table 1) that themselves were reared under standardized conditions with similar social densities, access to resources and environmental conditions. Thus, we made every effort to limit potential variation among mothers and provide identical rearing conditions for the experimental offspring.

To follow individual behavior, we built a custom automated recording system using Raspberry Pi computers and cameras^25^. Specifically, we programmed cameras above each of the experimental tanks (using pirecorder^26^) to take a photo every three seconds during the daylight period (11 h per day) for the first 10 weeks of the animals’ lives. We then convert these photos to a video, one per tank per day, and save the video on an external server (Supplementary Fig. 1). Altogether over the entire experiment, our system generated close to 1 million data points per animal (13,200 photos per day per animal x 70 days of tracking = 924,000 photos). We use a custom tracking software^27^ to extract the fish’s x and y coordinates in each frame, and then use these coordinates to compute fish’s swimming speed and distance to the tank walls for each frame, which we use to estimate their median swimming speed, activity (proportion of time spent moving >0.5 cm/s), variation in activity (inter-quartile range of swimming speed over the hour or day) and median distance from the tank walls for each hour and day. We then use Bayesian linear hierarchical models to quantify the magnitude of among-individual behavioral variation (i.e. behavioral individuality) and test how patterns of individual behavioral variation change over time.

Our findings show that substantial behavioral individuality is already present at the very first day of life after birth among genetically identical individuals, suggesting that pre-birth processes like epigenetics, pre-birth developmental stochasticity and/or maternal effects might play considerably more important roles in shaping behavioral individuality than commonly thought. Importantly, we find that these early signatures of individuality gradually strengthen over ontogeny and predict behavior up to at least ten weeks later (the end of our observation period), suggesting that once individuality is triggered, it sets the starting point for further behavioral differentiation.

## Results

### Behavioral individuality is present on the first day of life after birth

**Fig. 2:**
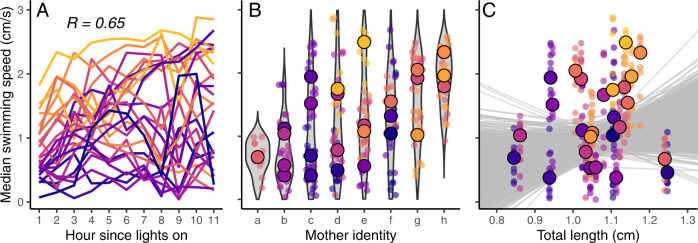
Individuality is present at day one after birth and is not explained by differences in maternal identity or body size. Repeatability of median swimming speed at hourly intervals on the first day after birth (**A**); each line represents one individual (*N* = 26). Maternal identity (**B**) did not explain variation in swimming speeds among individuals. Small and large points indicate the hourly (i.e. 11 data points per individual) and daily median swimming speeds, respectively, of individuals from each mother on day one after birth; see also Table 1. Behavior on day one after birth (**C**) was not related to an individual’s total length on their first day of life; see also Supplementary Table 3. Small and large points indicate hourly and daily median swimming speeds for each individual respectively; gray lines indicate posterior estimates for the effects of body size on behavior. Throughout, lines and points are colored according to the individual’s behavior in hour one on day one (yellow represents higher swimming speeds; purple indicates lower swimming speeds). Source Data are provided as a Source Data file.

Our first goal was to detect when behavioral individuality first appeared among our genetically identical and identically reared individuals. We found that even on the very first day after birth individuals showed large consistent individual differences in behavior (repeatability (*R*) of hourly swimming speed = 0.65, 95% Credible Interval = [0.48, 0.80]; activity = 0.71 [0.56, 0.84]; variation in activity = 0.68 [0.52, 0.81]; border distance = 0.80 [0.64, 0.87]; Fig. 2A; Table 1, Supplementary Table 2). This extremely early onset of individuality is a robust finding: we find that behavior was also significantly repeatable on each of the subsequent days of life (days 2–7; Supplementary Table 3) and when considering behavior across the entire first week of life combined (R = 0.57 [0.41, 0.50], Supplementary 4). As median swimming speed was tightly correlated with the other behavioral variables throughout the entire observation period (Supplementary Fig. 2), we focus on swimming speed throughout (results for other behaviors show a similar pattern and are reported in Supplementary Table 2).

**Table 1:** Maternal identity does not explain behavioral individuality

Model	Random effects	df	DIC (day one)	DIC (week one)	DIC (10 weeks)
0	Null	4	611.96	4617.31	3666.76
1	Intercepts ID	5	335.06	3772.22	2473.94
2	Intercepts mother	5	548.34	3999.10	2823.29
3	Intercepts ID Intercepts mother	6	335.06	3771.72	2473.57
4	Intercepts ID Slopes ID	6	**234.68**^a^	**3488.37**	**2313.16**
5	Slopes ID Slopes mother	6	543.77	3900.98	2777.04
6	Intercepts ID Slopes ID Intercepts mother	7	234.57	3487.79	2312.49
7	Intercepts ID Slopes ID Intercepts mother Slopes mother	8	236.72	3487.93	2312.84

There is strong support for the inclusion of individual intercepts and slopes both on the first day of life, first week of life and over the entire 10-week experiment. There was no evidence that including maternal identity as a random effect improved the models (reductions in DIC of at least 5 are considered support for inclusion of the effect in the model). All models included the fixed effects of ‘hour’ (day one models) or ‘observation day’ (week one and 10-week models) and ‘body size (TL)’ (mean-centered).

^a^best supported model (lowest DIC) is bolded.

There was no evidence that the observed variation in swimming speed can be explained by maternal identity, be it on the first day of life, the first week of life or over the entire 10-week experiment (Table 1; Fig. 1B; days 2–7 in Supplementary 5). Similarly, there was no evidence that body size (automatically extracted from the tracking photos) explained the observed variation in swimming speed on the first days of life (effect of total length on day 1 = 1.49 [−0.53, 3.39]; Fig. 1C; days 2–7 in Supplementary Table 3) nor did body size or growth rate appear to influence individual behavior across the first week of life (Supplementary Table 4) or the entire 10-week experiment (Table 2). There was also no evidence that brood size affected individual body size (effect of brood size = −0.004 [−0.02, 0.01]). Altogether, considering either the first week of life or any of the first seven days of life separately, the fixed effects of observation (hour or day), body size, and growth rate explained comparatively little of the total behavioral variance (marginal R^2^ of day 1 = 0.13 [0.02, 0.30]; days 2–7 all <0.04], Supplementary Tables 3, 4) compared to the random and fixed effects together (conditional R^2^ first day = 0.74 [0.57, 0.83]; days 2–7 in Supplementary Table 3), indicating that individual identity was a substantially better predictor of individual behavior than maternal identity, body size, growth rate or observation.

**Table 2:** Morphological and physiological differences do not explain individuality

Model (DIC)	Effects	Estimates [95% CI]	R^2^_marg_	R^2^_cond_
1 (288.13)	Obs week Weekly TL Intercepts ID Slopes ID Residual	0.02 [−.011, 0.16] −0.02 [−0.75, 0.56] 0.09 [0.02, 0.26] 0.004 [0.001, 0.009] 0.18 [0.14, 0.21]	<0.001 [0, 0.06]	0.36 [0.14, 0.62]
2 (288.39)	Obs week Weekly growth Intercepts ID Slopes ID Residual	0.02 [−0.02, 0.05] 0.09 [−0.66, 0.99] 0.11 [0.02, 0.24] 0.003 [0, 0.009] 0.18 [0.14, 0.21]	<0.001 [0, 0.04]	0.39 [0.14, 0.63]
3 (286.68)	Obs week Overall growth Intercepts ID Slopes ID Residual	0.009 [−0.02, 0.05] 38.36 [−36.60, 112.13] 0.09 [0.01, 0.27] 0.003 [0, 0.009] 0.17 [0.14, 0.21]	0.002 [0, 0.15]	0.40 [0.15, 0.65]
4 (290.46)	Obs week Weekly TL Weekly growth Overall growth Intercepts ID Slopes ID Residual	0.001 [−0.13, 0.19] 0.03 [−0.87, 0.74] 0.08 [−0.98, 1.12] 29.48 [−39.18, 116.96] 0.10 [0.01, 0.27] 0.003 [0, 0.009] 0.18 [0.14, 0.22]	0.01 [0, 0.16]	0.42 [0.16, 0.65]
5 (288.79)	Obs week Overall average TL Weekly diff TL Intercepts ID Slopes ID Residual	0.03 [−0.10, 0.19] 0.38 [−1.11, 1.68] −0.05 [−0.82, 0.57] 0.09 [0.02, 0.27] 0.004 [0, 0.009] 0.18 [0.14, 0.21]	0.007 [0, 0.12]	0.38 [0.16, 0.64]

Comparison of the explanatory power of different aspects of body size (TL = total length) and growth rates on weekly median swimming speed behavior. Values of less than 0.001 are listed as ‘0’. R^2^_marg_ and R^2^_cond_ are the marginal and conditional R^2^ values which are the proportion of total behavioral variation explained by the fixed effects only or by the fixed and random effects combined, respectively.

### Behavioral individuality gradually strengthens over ontogeny

**Fig. 3:**
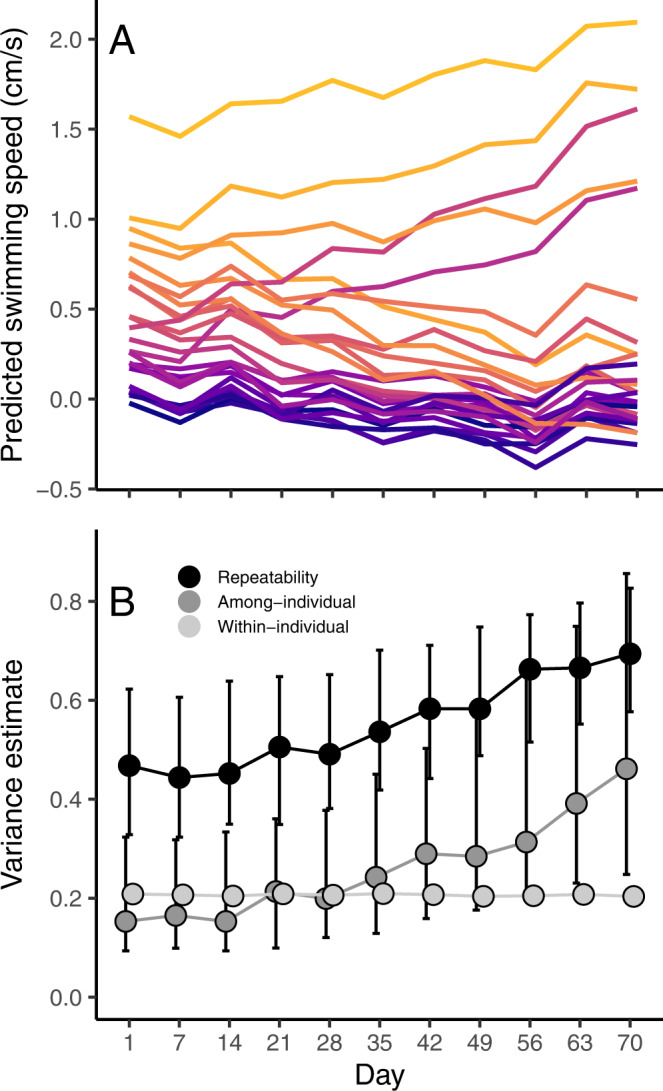
Individuality increases gradually throughout the first 70 days of development. The predicted values of median individual swimming speed diverge over time (**A**) leading to gradual increases in the among-individual variance and hence repeatability (**B**) of behavior. These models included only the 26 individuals on which we had complete data for the first 10 weeks of life to ensure that absolute levels of variation would remain comparable over time. Individual lines in (**A**) are colored according to their predicted behavior in week 1 with yellow indicating greater swimming speeds and purple indicating lower swimming speeds. In (**B**), shown are the posterior modes of each variance estimate and its 95% CI. Source Data are provided as a Source Data file.

Our second goal was to map the continued development of individuality over the entire 70-day observation period. At this longer timescale the support for the inclusion of random slopes at the individual level (Table 1), suggests that patterns of individual variation systematically changed over time. To account for this effect and enable the estimation of among- and within-individual variation across development, we ran a series of models where we centered the data on different observation days: the first model was centered on day one, the second on day seven, the third on day 14 and so on until the 10^th^ week after birth (day 70). This revealed a “fanning-out” pattern of predicted intercepts of individual behavior (Fig. 3A), indicating a gradual increase in among-individual variation and therefore repeatability of behavior throughout the first ten weeks of the fish’s lives (Fig. 3B).

We found that individuals consistently differed in their absolute body size over the entire observation period (*R* = 0.57 [0.37, 0.74]; Supplementary Fig. 3), however there was no evidence for consistent differences in weekly growth rates showing that while some individuals were always larger (or smaller) than others, individuals were not growing at consistently different rates over time (*R* < 0.01 [0,0.03]). Overall, considering the entire 70-day observation period, there was no evidence that individual behavioral variation was significantly explained by variation in any aspect of body size or growth rates (these fixed factors all explained less than 1% of the behavioral variation (R^2^_m_), Table 2).

### Early life individuality is predictive of later life individuality

**Fig. 4:**
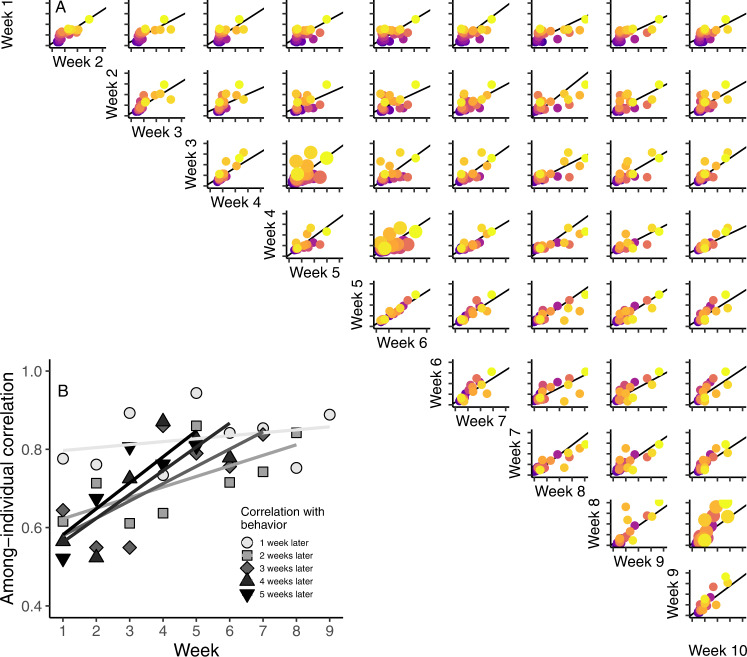
Earlier-in-life individuality predict later-in-life individuality. **A** Individual behavior was significantly positively correlated across all 10 weeks of observation. Each plot represents the among-individual correlation in behavior between two particular weeks in the ten-week experiment. The points in the plot represent the individual’s behavior for that week; throughout, individuals are colored according to their week 1 behavior where yellow represents greater swimming speeds and purple represents lower swimming speeds. The line on each plot is the estimated among-individual correlation between behavior in those two weeks; correlations were significant across all weeks. **B** Shown are the among-individual correlations between behavior in a given week and behavior 1, 2, 3, 4, or 5 weeks later. The correlations with behavior 2–5 weeks later have significantly steeper slopes than the correlation with behavior 1 week later showing that the among-individual correlation in behavior becomes more predictive of later behavior, later in life. Notice that the y-axis starts above zero. Source Data are provided as a Source Data file.

Our third goal was to investigate how predictive individual differences in behavior were, at any given point in time, for behavioral differences later in life. We estimated the among-individual behavioral correlations in daily median swimming speed across all weeks in the first ten weeks of life. We found that the among-individual correlations were significantly and strongly positively correlated across all weeks, including from the first to the tenth week of life (Fig. 4A). Additionally, we found that the among-individual correlation in behavior further separated in time became stronger as the animals age (interaction between week and time interval = 0.011 [0.002, 0.02], Fig. 4B). That is, behavior in a given week is always highly predictive of behavior in the following week (correlation with behavior one week apart, Fig. 4B), but behavior early in life (e.g. weeks 1–3) is not as predictive of behavior several weeks later as is behavior later in life (correlation with behavior five weeks apart, Fig. 4B). Altogether, our results thus demonstrate that individual differences in behavior diverge and stabilize over development.

## Discussion

Evidence is accumulating that even genetically identical animals reared under near identical conditions develop behavioral individuality^4–13^, yet little is known about when exactly these differences emerge during ontogeny and how they continue to change during early life development. We show that genetically identical individuals already exhibit substantial behavioral individuality on their first day of life, highlighting pre-birth influences as being of critical importance to initializing durable behavioral differences among individuals. Epigenetic and maternal effects mediated through mechanisms such as changes to DNA methylation patterns or differential resource or hormone allocation, could influence the phenotype of offspring^15,17,28^. For example, behavioral differences caused by changes in DNA methylation status have previously been related to differences in pre-natal maternal experience^29^. While Amazon mollies do not actively provision their growing embryos through a placenta^30^, it is plausible that differences in the internal maternal environment could still influence the growing embryos, for example, through differences in maternal resources^31^ or hormones^32^. In this study, we limited variation in maternal experience by rearing mothers under similar social densities and environmental conditions and indeed did not find evidence that maternal identity explained offspring behavior. Future work like ours that carefully controls both genetic and environmental effects from birth but purposefully and systematically varies the environment and/or conditions the mother fish experience could help further elucidate the potential of maternal effects on offspring behavioral divergence.

Another non-mutually exclusive hypothesis is that the behavioral variation we observed is the result of developmental stochasticity, that is, stochastic variation in any molecular, neurological or physiological markers that occur over ontogeny^12,13,19,20,33^. An intriguing possibility is that the phenotypic variation we observed here––whether arising from epigenetic, maternal, and/or developmental stochasticity effects––might itself be adaptive, for example, as a potential bet-hedging strategy^34^. Generating phenotypic variation among one’s offspring by such non-genetic means might be especially relevant in clonal organisms such as the Amazon molly^2^. There is, for example, evidence in clonal fish^35^, and poecilid fish specifically^36^, that DNA methylation mechanisms and developmental plasticity more generally^37^ might be especially sensitive to environmental influences, offering a mechanism through which mothers can generate variation among their otherwise genetically identical offspring.

It is important to note that, as with any experiment, a limitation of our study is that it is impossible to perfectly control every aspect of the environment. Thus, while every effort was made to minimize experiential differences among the newly born individuals, it remains possible that they could have experienced some aspects of their environments differently in the first hours of life (i.e. between birth and transfer into the experimental tanks), triggering both the observed behavioral individuality on the first day of life and the associated behavioral divergence later in life, thereby providing an alternative explanation for our findings.

Regardless of which specific mechanism originally generates behavioral differences, we show here that these initial differences continue to diverge and strengthen gradually over development. Such ‘fanning out’ patterns are considered indicative of positive feedback loops, which can occur in response to the internal or external environment^21,22,38,39^. For example, increasing activity may lead to greater access or intake of resources, in turn leading to further behavioral change. Phenotypic change can also be generated from internal changes. There is evidence of allelic expression differences even among clonal sisters of the Amazon molly^40^, which could contribute to behavioral variation among individuals. In human monozygotic twins, the accumulation of differences in methylation patterns across the genome correlates with patterns of phenotypic discordance over the lifetime^41^. Our work suggests that, in whatever way they are initiated, these early behavioral differences can influence future trajectories of behavioral development, which could have meaningful impacts on later fitness. For example, movement-related behaviors such as average and maximum swimming speed, have been shown to be fundamental to fishes’ ability to successfully forage, escape predation and school with conspecifics^42,43^. Our findings therefore provide support for two hypotheses about the emergence of individuality: individuality was evident at birth (e.g. Fig. 1A), and while these initial differences set the seed for individuality, further differentiation in behavior was still possible (e.g. Fig. 1B). We saw that very early life behavior predicted later life behavior under benign conditions where external environmental variation was controlled as much as possible. Importantly, this does not to suggest that individuals are incapable of more dramatic behavioral change (i.e. rank-order change) when put under greater environmental pressure. Future work that manipulates environmental variation can better investigate the patterns and mechanisms determining how individuals adjust to external environmental cues.

Behavioral individuality is a fundamental characteristic of animal populations; our study provides a null model about how such between-individual differences can change and develop in the absence of genetic and environmental differences. The work presented here offers critical insights into the drivers of behavioral individuality: as it is commonly thought, individuality arises mainly through genetic differences, differential experiences and particular ecological conditions; our findings suggest that subtler processes pre-dating birth may play a fundamental role in setting the seed around which behavioral individuality crystalizes. Stated simply, individuals are born unique, but this does not preclude further behavioral change throughout life.

## Methods

All animal care and experimental protocols complied with local and federal laws and guidelines and were approved by the appropriate governing body in Berlin, Germany, the Landesamt fur Gesundheit und Soziales (LaGeSo G-0224/20).

### Experimental breeding and design

The all-female Amazon molly (*Poecilia formosa*) is a naturally clonal, live-bearing fish species that gives birth to broods of genetically identical offspring. Like all unisexual vertebrates, Amazon mollies are the result of inter-specific hybridization^44,45^. As such, this ‘frozen hybrid’ has a heterozygous genome from its ancestral *P. mexicana* mother and *P. latipinna* father alleviating concerns about reduced genetic variation and the resulting inbreeding depression often associated with artificially selected isogenic animals. Additionally, despite their clonal nature, the Amazon’s genome shows no evidence of increased mutation accumulation, genomic decay or transposable element activity suggesting the genomes of these animals are evolving in similar ways as sexual species^46^. They reproduce through gynogenesis where the meiotic process is disrupted so that the eggs contain a full maternal genome. The egg must be fused with a sperm from one of their ancestral species to stimulate embryogenesis, but this paternal DNA is not incorporated into the egg. This provides the opportunity to control when reproduction occurs by controlling the females’ access to male sperm donors.

We placed adult females, as potential mothers of experimental fish, in individual (5-gallon) breeding tanks with two Atlantic molly (*P. mexicana*) males for one week to act as sperm donors. Amazon mollies give birth to broods of generally ~8-30 individuals. A brood is born at once (i.e. all individuals are born within minutes of each other) and birth generally happens early in the day close to dawn. These parental fish were lab-bred and themselves sisters, so of the same age and lineage, and were kept at similar social densities and under standardized environmental conditions throughout their lives to further minimize potential variation in maternal experience. Each breeding tank contained an artificial plant as refuge and was checked frequently each day for the presence of offspring, especially during the morning hours when births are most likely. Newborn mollies were always found in the morning and then singly netted by trained animal caretakers, into individual experimental tanks where their behavior was automatically recorded for the next 70 days (see below). Moving the fish from the maternal tank to the experimental tanks was done in a standardized manner (i.e. individual fish were netted and placed into small dishes of water and then placed in the tracking tanks to limit exposure to the air) by the same caretakers to minimize variation in experience among individual fish. Altogether, eight mothers provided offspring that completed the entire 10-week experiment (Supplementary Table 1).

Experimental tanks (27 x 27 cm), made of white Perspex, consisted of four equally sized compartments, and were evenly lit from below using 6500K-LEDs. Environmental conditions were highly standardized across tanks: all tanks were on the same 11:13 (L:D) light schedule, water depth was maintained at 10 cm depth, temperature was maintained at 25 ± 1 °C by a room air conditioning system, and fish received a standardized amount of powdered flake fish food (TetraMin™) twice daily. Opaque blinds surrounded the tanks to further limit outside disturbances. All experimental tanks were connected to the same filtration system where water could mix in the sump tank, allowing chemical cues to be shared across all experimental fish. Previous work has shown exposure to just chemical cues of conspecifics is sufficient in preventing the developmental of pathological behavior that could be associated with development in complete isolation^14^. We initially placed a total of 40 newborn individuals into the tracking tanks. At the end of the 10-week experiment, we were able to achieve complete tracking data on 26 individuals; camera malfunctions prevented data collection on four individuals, two individuals jumped into neighboring tanks causing the loss of data of all four individuals as we could not verify their identity; four newborn individuals escaped through holes in the water outlet of the tanks; and four individuals died as newborns. All results in the manuscript are on these 26 animals, though including data from all 40 (e.g. patterns of individual variation on the first day post birth) did not change the results or their interpretation (see Supplementary Table 2).

### Behavioral tracking

We developed a custom recording system using Raspberry Pi computers, which are an upcoming low-cost, highly adaptable solution for many applications in the biological sciences^25^. Specifically, we created a local network of Raspberry Pi 3B + ’s, each connected to a Raspberry Pi camera positioned exactly above an experimental tank, commanded by a lab computer, and connected to the server on the institute network (Supplementary Fig. 1). We programmed the Raspberry Pi’s using pirecorder^26^ to take timestamped photos every 3 s across the daily light period, each day, for 10 weeks, and store them automatically in dedicated, automatically named folders on the server. Image settings and resolution were thereby optimized to minimize file size while assuring image quality. After the experimental period, we created videos of all the recorded images of each fish of each day. These videos were subsequently tracked with the Biotracker software^27^, using background subtraction, providing the x, y coordinates of each fish in each frame. We then processed the data, including scaling and converting the coordinates to mm, and, for each frame, computed fish’s swimming speed (cm/s) and distance from the tank walls (cm). We then summarized these variables both on an hourly and daily basis to compute fish’s median swimming speed, inter-quartile range of swimming speeds, activity (proportion of time spent moving >0.5 cm/s), and median border distance. To quantify fish’s body size over time, we randomly selected five photos per week of each compartment, making sure the fish was away from the compartment walls and did not show strong body curvature, and then used ImageJ software to measure total body length (mm) from the tip of the snout to the end of the body. By averaging the measurements of the five images, we acquired one body size measurement per week.

### Error checking

We collected up to 924,000 photos on each individual throughout the experimental period resulting in a total of over 24 million data points collected on our experimental animals (*N* = 26 individuals). To ensure that our tracking software accurately captured the behavior of our fish, we checked for potential tracking errors in two ways. First, we estimated overall error rates. To do this, we selected at random a starting frame from within a day; then we manually checked each of the subsequent 200 frames and identified whether an error was made (fish was not properly located by BioTracker) or not (fish was properly located) by visual inspection of the videos. We estimated the error rate as the number of errors divided by the total number of checked frames. The overall median error rate over the entire observation period was estimated to be 7%. Error rates increased earlier in the observation period when the fish were smaller (Supplementary Note I). As such, as a second step, we manually went through and corrected all frames for the very first day of tracking (i.e. day 1 post-birth) for all fish (~13,200 frames per individual) as this is a critical time period for one of our research questions. This ensured that the resulting behavioral data were completely accurate for this day. This manual correction allowed us the additional opportunity to compare how well our automatically tracked (i.e. not manually corrected) data performed compared to the manually corrected data. We found that the automatically tracked data re-created near identical estimates of among- and within-individual variance components and most importantly the among-individual correlation between the automatically tracked and manually corrected data was over 0.98 for our behavioral variables (Supplementary Note I). This strongly suggests that any errors introduced by our automated tracking software have minimal influence of our behavioral variables at best and do not affect our interpretation of the results.

### Statistical analyses

We used linear mixed, or hierarchical, models to partition the behavioral variation across different times periods into its among- and within-individual components. Throughout we focused our analysis on the 26 individuals for which we had complete data for the entire 10-week observation period to ensure comparable variation over time and across models.

Our first question of interest was to test when individual differences in behavior first appeared over the course of the experiment. We started by investigating behavior on the first day post birth (Fig. 1A, Supplementary Table 2) and then planned to proceed in a day-by-day fashion until significant repeatability in behavior was apparent (Supplementary Table 3). We used hourly median swimming speed (11 observations for each of 26 individuals) as our response variable and included ‘hour’ and ‘total length (TL)’ as fixed effects and ‘individual’ was included as our random effect of interest. Including TL as a covariate allowed us to test whether behavior was related to an offspring’s body size on its first day of life. We set the first hour of the day as 0 and mean-centered TL as this would allow the among- (and within-) individual variance components to be estimated at these values (i.e. the earliest possible moment from when we could record behavior in the fish). We estimated the adjusted repeatability of median swimming speed as the variance attributable to individual identity over the total variance not explained by the fixed effects. We additionally estimated both marginal and conditional R-squared values which estimate the variance explained by the fixed effects only and the variance explained by the fixed and random effects combined, respectively. As our individual experimental fish came from different mothers, we first explored a number of different variance structures including random intercepts and slopes for both individual ID and maternal ID. This allowed us to test whether maternal identity explained variation in individual behavior. However, the most supported model included random intercepts and slopes for individual ID and not for mother ID, indicating that our methods to reduce variation among mothers were successful (Table 1). We used median swimming speed as our behavioral variable of interest throughout the main manuscript, as this behavior was tightly correlated with most of our other behavioral variables (Supplementary Fig. 2); though results using the other behavioral variables yielded the same interpretation (i.e. that significant individuality in (any) behavior was present on the very first day post-birth; Supplementary Table 2).

Our second research question was to investigate how individual behavioral variance changed over the course of the entire observation period (70 days). Again, we first explored several different variance structures to test the importance of maternal identity and/or individual identity on behavioral variation. We found support for the inclusion of random slopes at the individual level, but not maternal level (Table 1). This indicates that levels of among- (and within-) individual variation may differ throughout the observation period. To investigate patterns of change in the variance components, we ran a series of models where we centered the observation covariate on different days. Individual intercepts are estimated when all covariates are set to zero, so this allowed us to ‘slice’ the data to estimate the among- and within-individual variance at different time points over the ten weeks. We ran 11 models as we chose to center the data every 7 days (first model was centered on observation 1; 11^th^ model was centered on observation 70). The predicted individual intercepts (best linear unbiased predictors) and estimated variance components from each model are plotted in Fig. 3.

We also closely investigated any potential influence of body size and/or growth rate differences on behavioral expression and individual behavioral variation in this entire 10-week data set. First, we estimated the repeatability of both weekly total length and weekly growth rates to determine if individuals consistently differed in these traits. Then, we ran a series of models with median weekly swimming speed as the response variable and included either weekly total length, weekly growth rate, and/or overall growth rate (estimated over the entire 10 weeks), as our fixed effects of interest. Each model also included the random effects of individual intercepts and slopes. Finally, because body size varies both among individuals (some individuals are on average larger than others) and within individuals (as they grow), we also performed within-individual centering of total length. In this fifth model, we included each individual’s average total length and their weekly deviation from their average length as the two fixed effects of interest. Individual identity and slopes were included as random effects. For all models, we estimated the variance explained by the fixed effects (marginal R^2^) and the fixed and random effects together (conditional R^2^). These results are reported in Table 2.

For our third and final research question, we tested whether early-life behavior predicted later-life behavior. To test this, we estimated the among-individual correlation (including ‘individual ID’ as our random effect) in behavior using multivariate mixed models where the daily median swimming speeds in each week were the response variables (7 observations per week per individual; 10 weeks total; Fig. 4A). Then to investigate how the strength of these correlations may change over development, we used a linear model to test whether the correlation strength was predicted by the interaction between the first week included in the correlation and distance to the next week in the correlation (1, 2, 3, 4 or 5 weeks away in time; Fig. 4B).

All models were performed using Markov Chain Monte Carlo estimation with the MCMCglmm package^38^ in R v3.6.1^39^. We set our models to run 510,000 iterations with a 10,000 burn-in and thinning every 200 iterations. To ensure proper model mixing and convergence, we initially ran 5 independent chains and inspected posterior trace plots of parameter estimates (Supplementary Note II). In a preliminary analysis we tested three different prior settings (Supplementary Note II); results did not change with prior settings so we chose parameter-expanded priors for all models reported here as these are generally considered to be more robust. An R Markdown file with all the results presented here is included in Supplementary Note II.

### Reporting summary

Further information on research design is available in the Nature Research Reporting Summary linked to this article.

## Supplementary information

Supplementary Information

Reporting Summary

## Data Availability

The data generated in this study have been deposited in Dryad Depository [10.25338/B8XW7G].^47^ An R markdown file recreating the results and figures is included in the Supplemental Information (Supplemental Note II – Code to Reproduce Results). Source data are provided with this paper.

## Source data

Source Data

## Code availability

All code to reproduce the results are deposited in Dryad (10.25338/B8XW7G)^47^.
