# Supplementary material for: The emergence and development of behavioral individuality in clonal fish
Source: Nat Commun. 2022 Oct 28;13:6419. doi: 10.1038/s41467-022-34113-y (PMC9616841; doi:10.1038/s41467-022-34113-y)
Supplement: Supplementary file 1 — Supplementary Information [file 41467_2022_34113_MOESM1_ESM.pdf]

Supplementary Information for

The emergence and development of behavioral individuality in clonal fish

Authors: Kate L. Laskowski[1,2,†,*], David Bierbach[2,3,4,†], Jolle W. Jolles[5], Carolina Doran[2], Max Wolf[2,4]

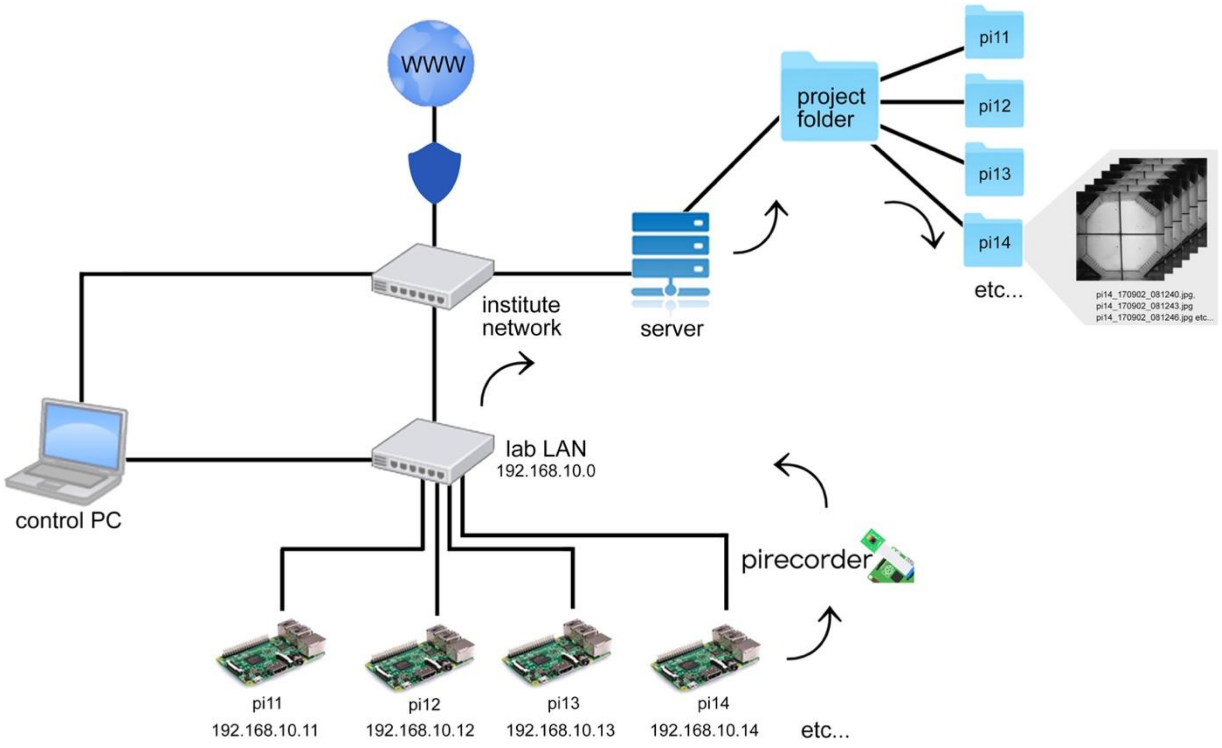

Supplementary Figure 1. Overview of the automated recording system, using a local network of Raspberry Pi computers and pirecorder recording software to store timestamped images on an external server.

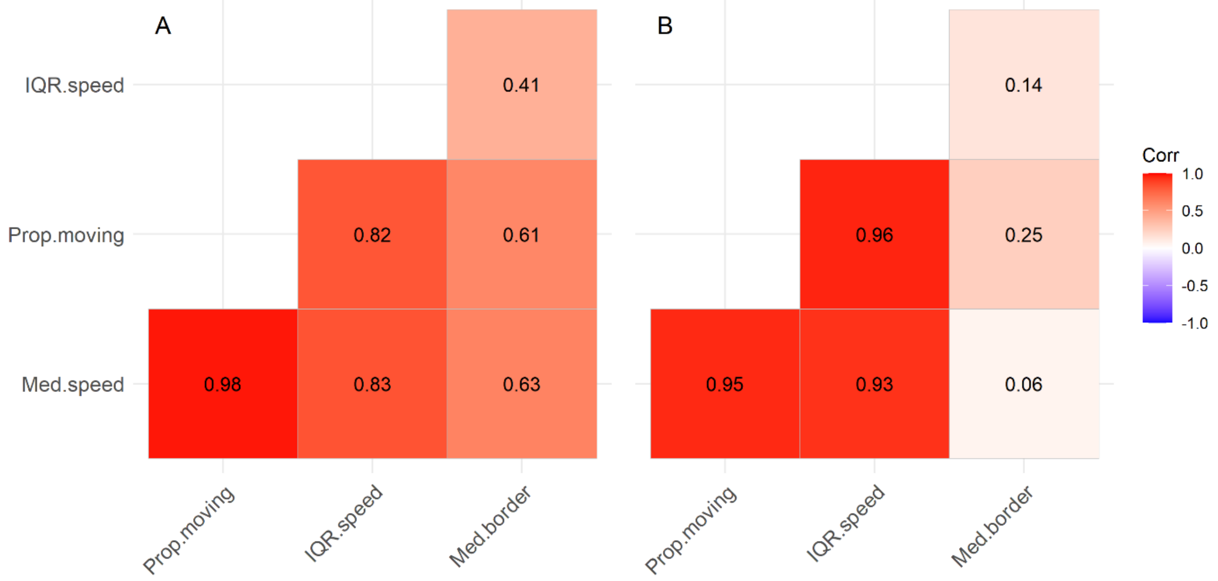

Supplementary Fig. 2. We found strongly significant among-individual correlations between most of our computed behavioral variables both on the first day of life (A) and over the entire observation period (B).

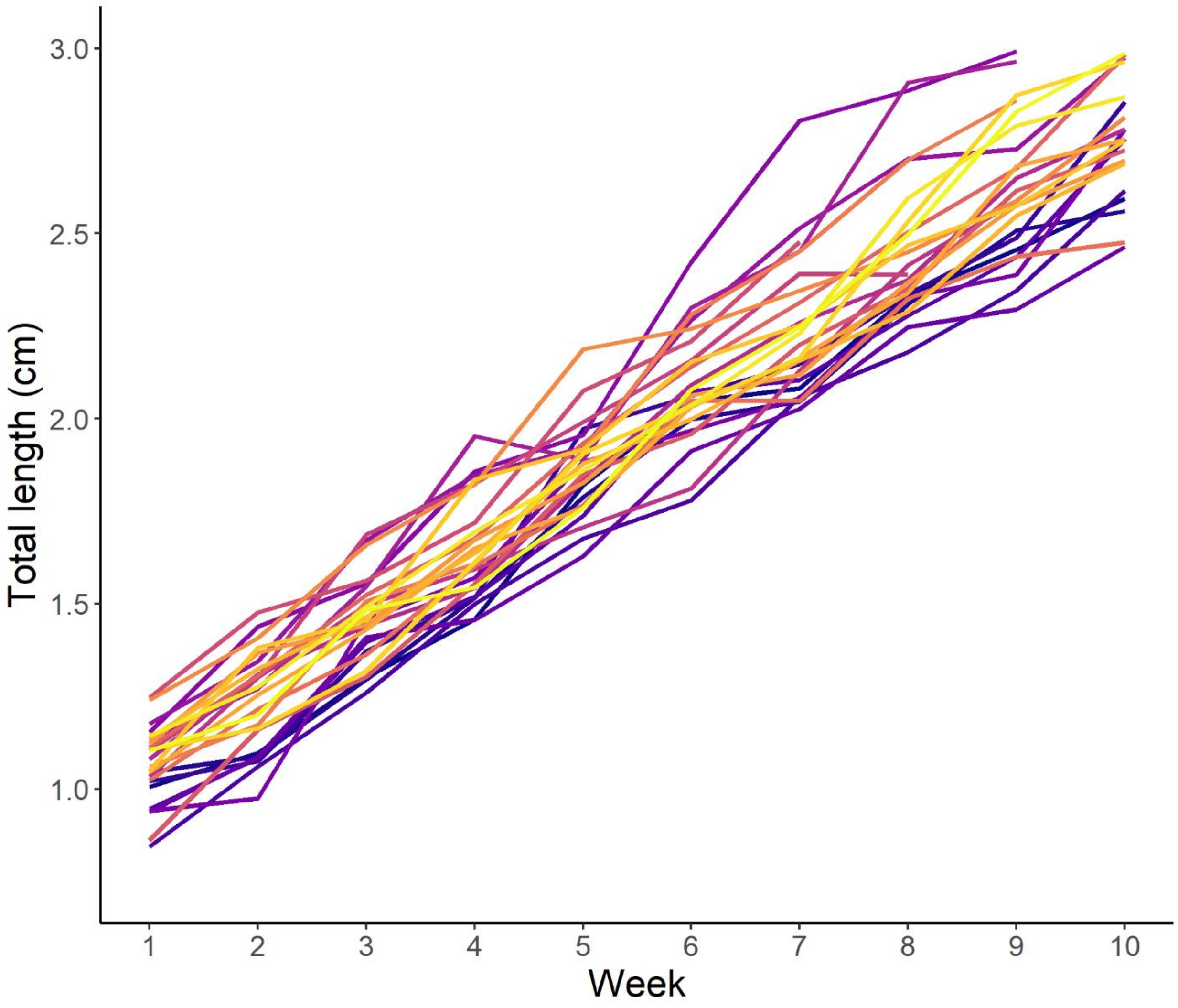

Supplementary Fig. 3. Plot of individual body sizes over the course of the experiment. Individuals consistently differed in their absolute body size over time, though these differences did not relate to differences in behavior.

Supplementary Table 1. Number of offspring for which we had complete data through observation 70 that were included in the study from each mother. Brood size refers to the number of offspring born in each brood (not all offspring from a brood were used in the study).

Mother	Experimental offspring	Birth date	Brood size
70.2 (c)	4	Feb 15, 2018	11
80.1 (f)	3	Feb 28, 2018	15
90.2 (a)	1	March 19 2018	12
75.2 (e)	4	March 19 2018	15
69.3 (b)	4	March 20 2018	30
70.3 (d)	4	March 26 2018	15
77.2 (g)	3	March 29 2018	11
80.2 (h)	3	April 4 2018	8

Supplementary Table 2. There was evidence for significant repeatability in the hourly measurements of all computed behavioral variables in our experimental animals in the very first day post birth. Shown are the results for the 26 individuals for which we have complete data until observation 70 (week 10) and all 40 individuals that were initially placed in the tracking tanks. Models were run including the fixed effects of 'hour' (1st hour set to zero) and 'body size' (mean-centered) and random intercepts and slopes for each individual. Var_{IND} and Var_{RES} report the posterior mode estimates for the among- and within-individual variation, respectively; among-individual variation in slopes was small and accounted for less than 0.1% of the variation in all behaviors and so is not reported here. Repeatability is estimated as the proportion of total variation (among-individual variation in intercepts, slopes and residual) that is not explained by the fixed effects (adjusted repeatability) that is due to among-individual variation in intercepts.

**Behavioral variable**	**Var_{IND} [95% CI]**	**Var_{RES} [95% CI]**	**Repeatabilty [95% CI]**
26 individuals with complete data to observation 70			
Median speed	0.18 [0.10, 0.42]	0.11 [0.10, 0.14]	0.65 [0.48, 0.80]
Speed IQR	85.00 [53.39, 210.61]	49.68 [41.87, 59.68]	0.68 [0.52, 0.81]
Proportion time spent moving	0.03 [0.01, 0.05]	0.01 [0.01, 0.02]	0.71 [0.56, 0.84]
Distance from tank border	7.52 [4.32, 45.48]	2.46 [2.14, 3.07]	0.80 [0.64, 0.87]
All 40 individuals initially placed in tracking tanks			
Median speed	0.24 [0.16, 0.45]	0.11 [0.09, 0.13]	0.71 [0.59, 0.81]
Speed IQR	136.73 [72.09, 205.02]	49.13 [43.03, 57.56]	0.70 [0.60, 0.81]
Proportion time spent moving	0.03 [0.01, 0.04]	0.01 [0.01, 0.01]	0.69 [0.59, 0.81]
Distance from tank border	7.33 [4.51, 12.66]	3.02 [2.57, 3.44]	0.71 [0.61, 0.82]

Supplementary Table 3. In addition to the results for the first day post birth shown above, there was evidence for significant repeatability of hourly median swimming speed on each day in the first week of life. Models were run including the fixed effects of 'hour' (1st hour set to zero) and 'body size' (mean-centered) and random intercepts and slopes for individual; day 6 also included random intercepts and slopes for mother. Reported are the posterior mode estimates for all effects; among-mother variation in intercepts and slopes was only estimated for day 6 (see Extended Data Table 4).

Effect	estimate (day 1)	estimate (day 2)	estimate (day 3)	estimate (day 4)	estimate (day 5)	estimate (day 6)	estimate (day 7)
Intercept	0.87	0.60	0.88	0.84	0.58	0.42	0.73
Hour	0.06	0.03	-0.007	0.03	0.007	0.06	-0.01
	[0.03, 0.09]	[0.009, 0.07]	[-0.03, 0.04]	[<0.001, 0.06]	[-0.02, 0.04]	[-0.04, 0.14]	[-0.05, 0.03]
Body size	1.49	2.07	0.84	0.57	1.01	-0.54	0.67
	[-0.53, 3.39]	[-0.89, 4.56]	[-1.61, 3.46]	[-2.18, 2.93]	[-1.57, 3.62]	[-2.77, 1.03]	[-1.87, 3.39]
R^2_{marg}[1]	0.13	0.04	0.001	0.02	0.002	0.02	0.003
	[0.02, 0.30]	[0.002, 0.25]	[<0.001, 0.12]	[0.001, 0.12]	[0.001, 0.13]	[0.001, 0.29]	[0.001, 0.09]
ID intercepts	0.18	0.34	0.44	0.28	0.34	0.04	0.57
	[0.10, 0.42]	[0.18, 0.76]	[0.23, 0.98]	[0.12, 0.67]	[0.13, 0.72]	[0.001, 0.18]	[0.30, 1.17]
ID slopes	0.006	0.003	0.005	0.002	0.003	<0.001	0.006
	[0.003, 0.01]	[0.001, 0.008]	[0.002, 0.01]	[0.001, 0.007]	[0.001, 0.009]	[0.001, 0.002]	[0.003, 0.01]
Mother intercepts	--	--	--	--	--	0.28	
						[0.004, 1.45]	
Mother slopes	--	--	--	--		0.008	
					--	[0.001, 0.04]	
Residual	0.11	0.16	0.21	0.30	0.25	0.25	0.20
	[0.10, 0.14]	[0.14, 0.20]	[0.18, 0.26]	[0.25, 0.36]	[0.21, 0.31]	[0.21, 0.31]	[0.16, 0.24]
Rpt (ID)[2]	0.65	0.69	0.70	0.57	0.57	0.03	0.77
	[0.48, 0.80]	[0.56, 0.84]	[0.56, 0.84]	[0.33, 0.72]	[0.38, 0.75]	[0.001, 0.24]	[0.63, 0.87]
R^2_{cond}[3]	0.74	0.76	0.75	0.52	0.61	0.12	0.78
	[0.57, 0.83]	[0.60, 0.86]	[0.58, 0.84]	[0.35, 0.74]	[0.42, 0.78]	[0.009, 0.52]	[0.65, 0.88]

[1]R^2_{marg} is the marginal R^2 and is the proportion of total behavioral variation that can be explained by the fixed effects in the model (hour and body size).

[2]Rpt (ID) is the repeatability of individual identity and is estimated as the proportion of total behavioral variation (that is not explained by the fixed effects) that can be explained by among individual variation in intercepts.

[3]R^2_{cond} is the conditional R^2 and is the proportion of total behavioral variation that can be explained by the fixed and random effects combined.

Supplementary Table 4. When considering behavior across the entire first week of life (7 days), there was also evidence that individuals consistently differed in their behavior though this variation was not explained by variation in body size or growth rate in the first week of life. Model includes the fixed effects of 'observation' (centered on first day post birth), body size (mean-centered), growth rate in the first week of life, and the interaction between observation and growth rate and the random effects of individual intercepts and slopes.

Effect	Estimate
Intercept	1.26
Observation	-0.03 [-0.17, 0.07]
Body size	-1.36 [-4.33, 2.41]
Growth rate	0.68 [-1.52, 2.63]
Obs x growth rate	-0.31 [-1.01, 0.34]
R^2_{marg}[1]	0.07 [0.03, 0.19]
Intercepts ID	0.39 [0.22, 0.78]
Slopes ID	0.01 [0.009, 0.03]
Residual	0.34 [0.32, 0.36]
Rpt (ID)[2]	0.57 [0.41, 0.70]
R^2_{cond}[3]	0.60 [0.47, 0.72]

[1]R^2_{marg} is the marginal R^2 and is the proportion of total behavioral variation that can be explained by the fixed effects in the model (hour and body size).

[2]Rpt (ID) is the repeatability of individual identity and is estimated as the proportion of total behavioral variation (that is not explained by the fixed effects) that can be explained by among individual variation in intercepts.

[3]R^2_{cond} is the conditional R^2 and is the proportion of total behavioral variation that can be explained by the fixed and random effects combined.

Supplementary Table 5. We tested whether the finding that individuals show significant repeatable variation on the first day post birth was robust. To do this, we explored the random effects structure on each day of the first week of life for hourly median swimming speed. All models were run on the 26 individuals for which we have complete data until observation 70. Each model included the fixed effects of 'hour' (1st hour set to zero) and 'body size' (mean-centered). For all days, except day 6, the most supported model included random intercepts and slopes for individual ID showing that the finding of repeatable individual behavioral variation so early in life is robust. With the exception of day 6 for which behavioral variation can be best explained by a model including both individual and mother identity – the best statistical model explaining the observed behavioral variation includes individual identity but not mother identity. Reductions in DIC of at least 5 are considered support for inclusion of the effect in the model.

Model	Random effects	df	DIC (day 2)	DIC (day 3)	DIC (day 4)	DIC (day 5)	DIC (day 6)	DIC (day 7)
0	Null	4	671.37	678.26	678.08	646.02	650.82	658.50
1	Intercepts ID	5	363.26	468.19	492.84	463.41	503.01	445.77
2	Intercepts mother	5	549.03	559.20	599.89	502.52	510.88	541.47
3	Intercepts ID<br>Intercepts mother	6	363.21	467.79	492.80	461.97	499.61	445.34
4	Intercepts ID<br>Slopes ID	6	**327.83**	**420.07**	**483.31**	**443.98**	448.99	**388.71**
5	Intercepts mother<br>Slopes mother	6	551.02	557.04	600.52	492.35	458.40	521.68
6	Intercepts ID<br>Slopes ID<br>Intercepts mother	7	328.21	419.89	482.09	443.52	448.26	389.35
7	Intercepts ID<br>Slopes ID<br>Intercepts mother<br>Slopes mother	8	329.88	421.24	483.21	443.34	**439.00**	390.85

Supplemental Note I Error checking of automated tracking of molly behavioral development

K Laskowski

Sept 29, 2022

INTRO

We used Biotracker (https://www.igb-berlin.de/en/biotracker) to identify the location of each fish in each frame (1 frame every 3 seconds) from our tracking videos. As with any automated tracking software, errors can be introduced where the tracking software mis-identifies the location of the fish. These errors have the potential affect our estimates of our behavioral variables (which are estimated from the changes in x-y coordinate location of the fish in the frames).

We performed error checking in two ways:

1 - Overall error rates

For each pi (compartment), we selected every fourth video (starting with the first =day 1 video), then within each video (day), we chose a random start frame ("Start Frame") and then checked for roughly 200 frames per hand the number of tracking errors (num_errors) i.e. each frame was either an error or not. We then calculated the error rate from this "error_rate", i.e.: number of errors /number of checked frames.

- What is the overall error rate across all compartments?
- Does this error rate change over time (as the animals grow)?

2 - Manual correction of Day 1 tracks

we then manually corrected the full first day of tracking for each pi (fish). Now we have the behavioral variables for the automatically tracked (uncorrected) and manually corrected data allowing us to see how closely the automatic tracking software is able to capture the true behavioral measures. We chose to look at Day 1 because 1) this is a critical time frame for our major research question of when individual differences in behavior appear and 2) this is when fish are the smallest and so errors are likely to be highest and so this is the hardest tracking that the software has to perform.

- What is the correlation between automatically tracked and manually corrected data?

DATA PREP

```
knitr::opts_chunk$set(tidy.opts = list(width.cutoff = 60), tidy = TRUE)

library(dplyr)
```

```
##
## Attaching package: 'dplyr'

## The following objects are masked from 'package:stats':
##
##     filter, lag

## The following objects are masked from 'package:base':
##
##     intersect, setdiff, setequal, union
```

```
library(tidyr)
library(ggplot2)
library(MCMCglmm)
```

```
## Loading required package: Matrix

##
## Attaching package: 'Matrix'

## The following objects are masked from 'package:tidyr':
##
##     expand, pack, unpack

## Loading required package: coda

## Loading required package: ape
```

```
library(formatR)

setwd("C:/Users/katel/Dropbox/7_Molly_Power/FINAL/analysis/Individual analysis/individual_molly_develop
rates <- read.csv("error rates_overall errors_for analysis 210816.csv") #overall error rates across ent
```

Now I need to wrangle the day 1 manually corrected data - the 'sumdat' data file has both the corrected and uncorrected data, split into 15 minute bins and a 'full' bin that includes the entire day. The dataWide file only contains the 15 min intervals, whereas the dataDay only includes the full day measurements

```
# manually corrected data
sumdat <- read.csv("sumdat_entire first day 210816.csv")
sumdat$compnr <- recode(sumdat$comp, leftup = 1, rightup = 2,
    leftdown = 3, rightdown = 4)
sumdat$picomp <- factor(paste(sumdat$rpi, sumdat$compnr, sep = "_"))

dataWide <- sumdat %>%
    filter(timesection != "full") %>%
    mutate(obs = as.numeric(timesection)) %>%
    select(rpi, picomp, type, obs, distmoved.iqr, speed.iqr,
        speed.med, propmoving, borderdist.med, se.pos) %>%
    pivot_wider(id_cols = c(rpi, picomp, obs), names_from = type,
        values_from = c(distmoved.iqr, speed.iqr, speed.med,
            propmoving, borderdist.med, se.pos))
```

```r
dataWide <- as.data.frame(dataWide)

dataDay <- sumdat %>%
    filter(timesection == "full") %>%
    select(rpi, picomp, type, distmoved.iqr, speed.iqr, speed.med,
        propmoving, borderdist.med, se.pos) %>%
    pivot_wider(id_cols = c(rpi, picomp), names_from = type,
        values_from = c(distmoved.iqr, speed.iqr, speed.med,
            propmoving, borderdist.med, se.pos))

dataDay <- as.data.frame(dataDay)
```

1 - What is the overall error rate?

First let's just look at the overall mean, median, mode error rate. The most common (mode) error_rate is 0, which is good. Median error rate is only 0.07, which means that on average the location of the fish was misidentified in just 7% (14) of the frames in each sampling bout (~200 frames). Mean error rate is 0.14, but clear to see that this value is not a good descriptor of the data given that it is not normally distributed.

```r
mean(rates$error_rate, na.rm = T)  # 0.14
```

```
## [1] 0.1428249
```

```r
median(rates$error_rate, na.rm = T)  #0.07
```

```
## [1] 0.07
```

```r
# no built in function to estimate mode (most common
# number)
getmode <- function(v) {
    uniqv <- unique(v)
    uniqv[which.max(tabulate(match(v, uniqv)))]
}
getmode(rates$error_rate)
```

```
## [1] 0
```

```r
ggplot(rates) + geom_histogram(aes(x = error_rate), color = "black",
    fill = "white", binwidth = 0.03) + xlab("Error rate") + annotate("text",
    x = 0.75, y = 150, label = "mean = 0.14", color = "red") +
    annotate("text", x = 0.75, y = 130, label = "median = 0.07",
        color = "red") + annotate("text", x = 0.75, y = 110,
    label = "mode = 0", color = "red") + theme_classic()
```

```
## Warning: Removed 4 rows containing non-finite values (stat_bin).
```

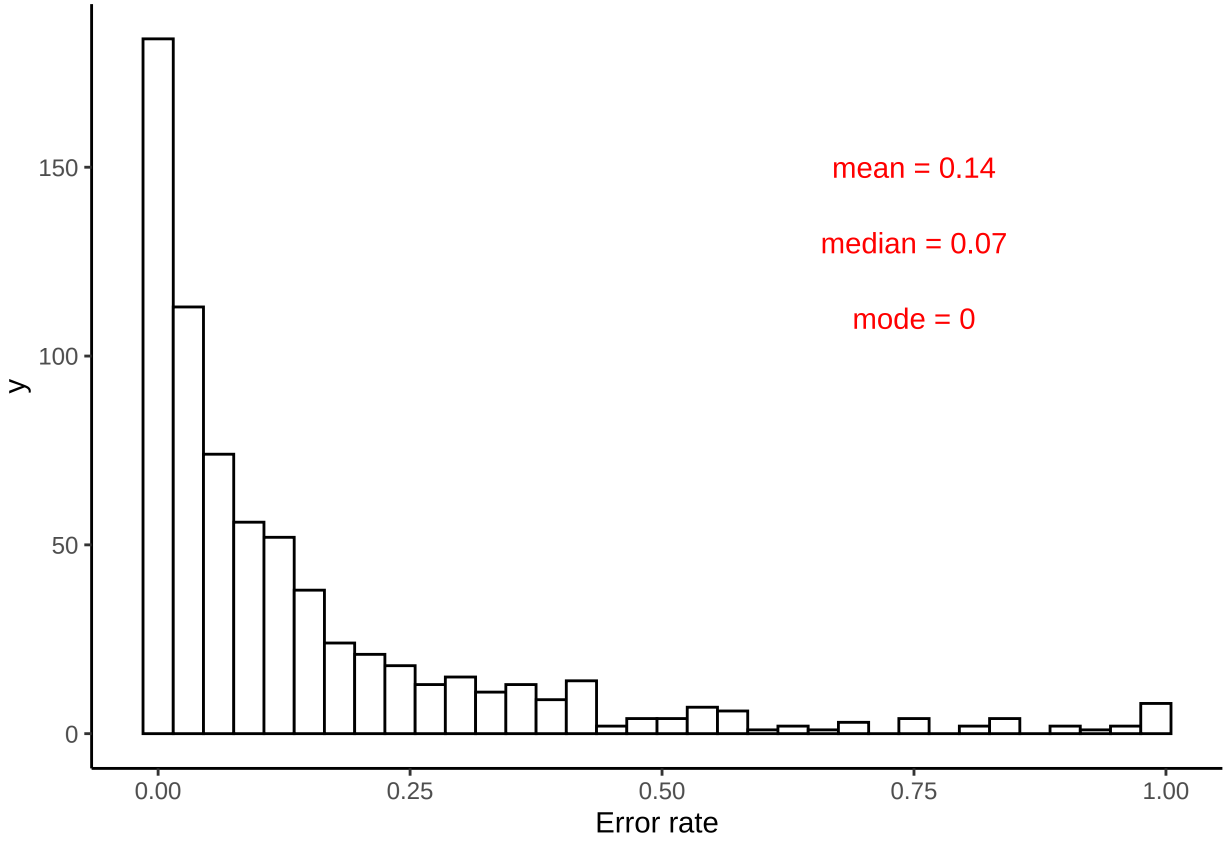

Might also be useful to look at the overall error rates by compartment. Here we can see that the overall median error rate of each pi is around 0.13, so about 13% of the selected frames were misidentified for each pi. However, 13% error rate seems quite good given the amount of data we are working with.

```
error.avg <- rates %>%
    group_by(picomp) %>%
    summarise(tot.errors = sum(num_errors, na.rm = T), tot.frames = sum(tracked_frames,
        na.rm = T), overall.error = tot.errors/tot.frames)

ggplot(error.avg) + geom_histogram(aes(x = overall.error), color = "black",
    fill = "white", binwidth = 0.03) + xlab("Overall error rate per compartment") +
    theme_classic()
```

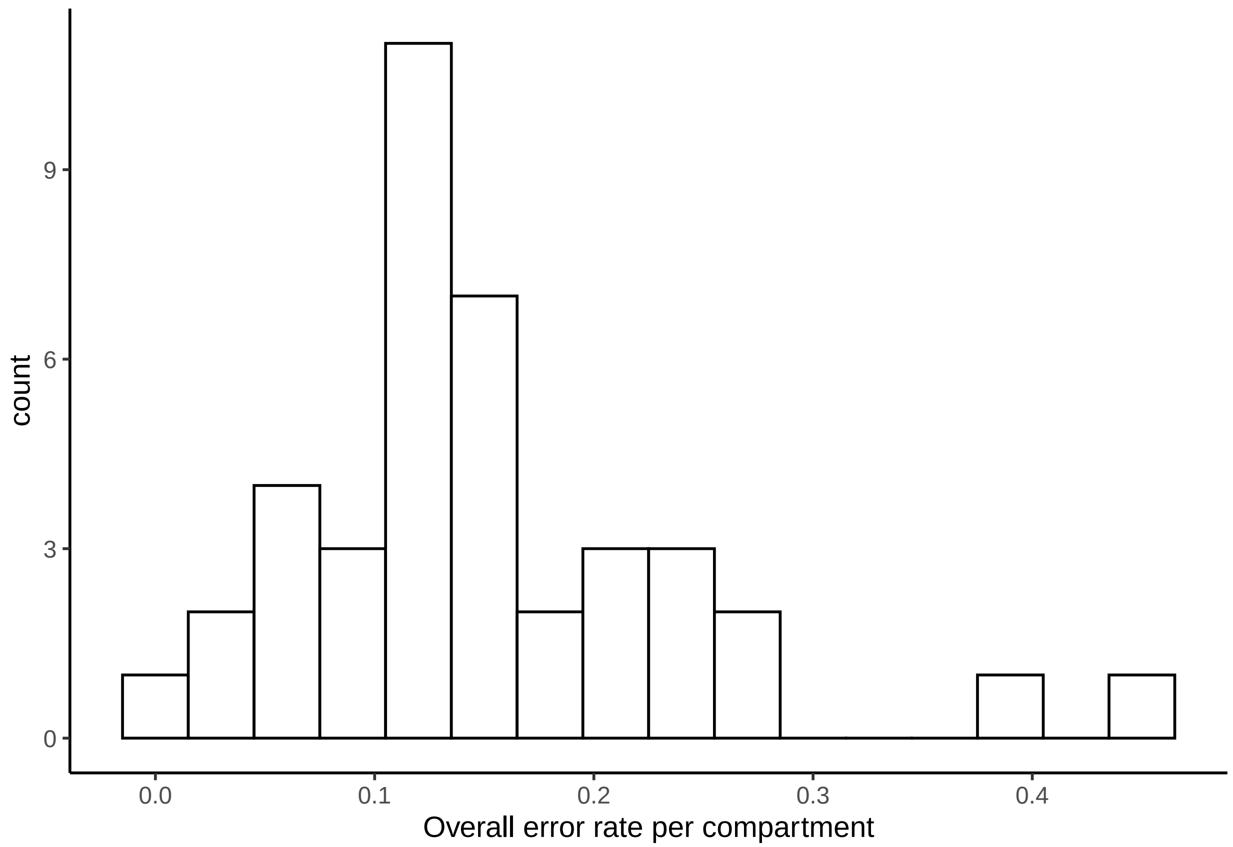


```
median(error.avg$overall.error, na.rm = T)
```

```
## [1] 0.1319394
```

2 - Does the error rate change over time?

Yes, the error rate is higher at the earlier sampling dates which makes sense given that fish are smaller when younger and so it's likely harder for the software to find them.

```
ggplot(rates) + geom_boxplot(aes(x = factor(sample), y = error_rate)) +
    xlab("Sampling date") + ylab("Error rate") + theme_classic()
```

```
## Warning: Removed 4 rows containing non-finite values (stat_boxplot).
```

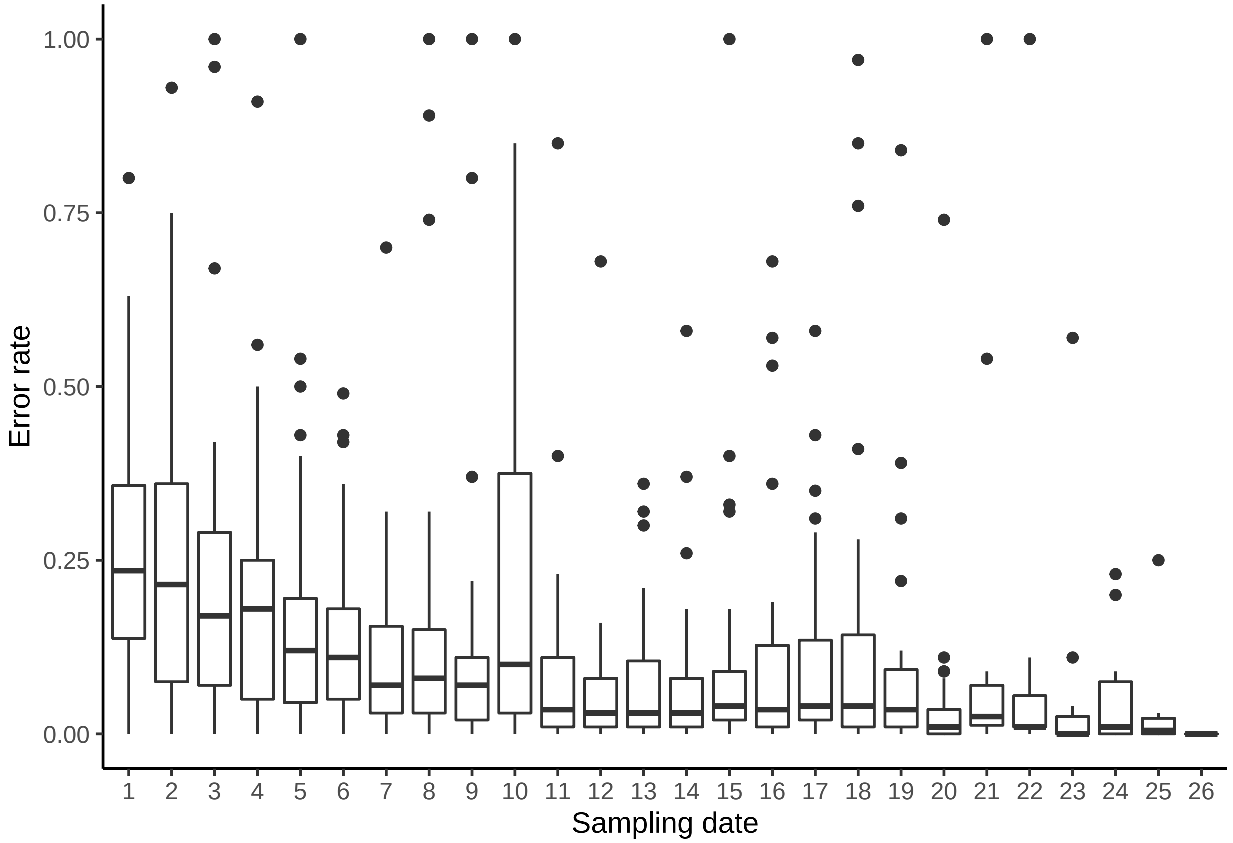

> **Bottom line:**

Overall median error rates are about 7% over the entire experiment, though these rates are higher earlier in life when the animals are smaller. This is why we manually corrected the tracking data for the first day of life (as this is a critical time period for our research questions).

3 - Correlation between corrected & uncorrected data?

Given that error rates are highest early in life when fish are smallest, and that one of our key questions is when individual differences in behavior first appear, we manually corrected the entire first day of tracking for all fish. In addition to ensuring that our data for this day are accurate, this gives us the opportunity to compare the relationship between corrected and uncorrected data. That is, if the uncorrected data closely correlates with the corrected data than we can feel confident that the errors are truly just introducing noise into our data and shouldn't systematically bias our data in any particular way.

First, let's look at the full day measurements (dataDay file) - we can see that really for all the behavioral variables we have pretty tight relationships between the manually corrected and automatically tracked (uncorrected) daily behavioral value.

If anything, for our main behavioral variable of intrest, median speed, the uncorrected values are somewhat underestimating the true behavior. This means that our automatically tracked (uncorrected) data has less variation in it to partition among the random effects which should make our estimates of among-individual variation more conservative (than if the uncorrected data was instead OVER estimating the true behavioral value).

```
ggplot(dataDay) + geom_point(aes(x = speed.med_cor, y = speed.med_unc)) +
    geom_abline(intercept = 0, slope = 1) + theme_classic()
```

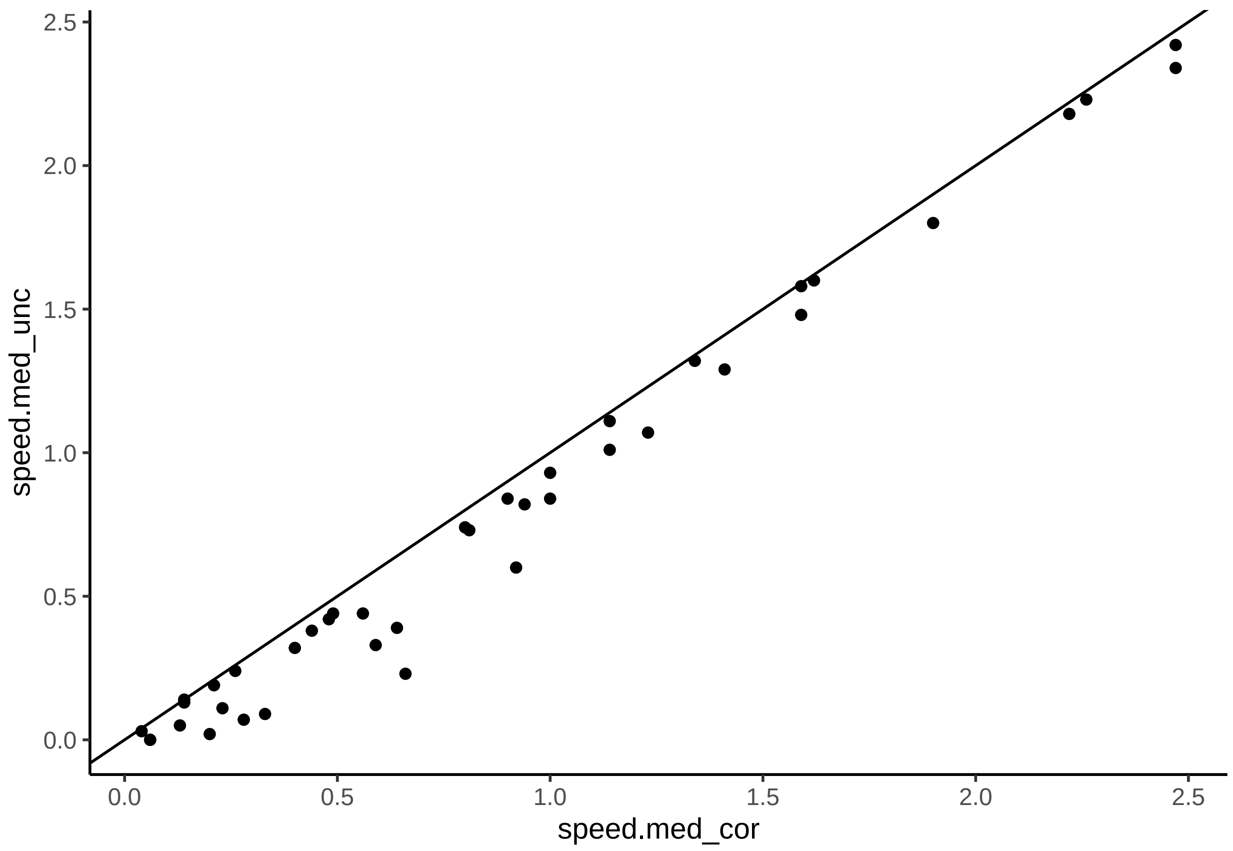


```
ggplot(dataDay) + geom_point(aes(x = distmoved.iqr_cor, y = distmoved.iqr_unc)) +
    geom_abline(intercept = 0, slope = 1) + theme_classic()
```

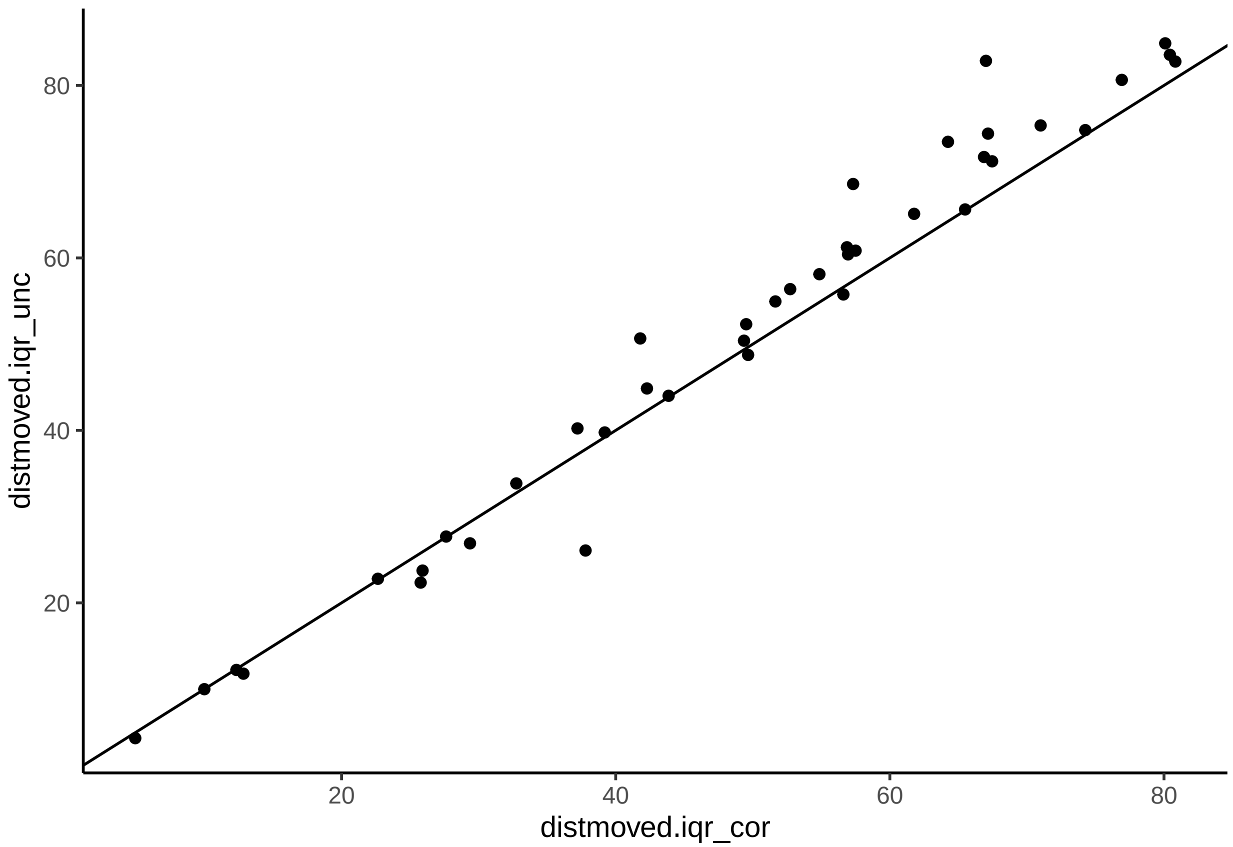


```
ggplot(dataDay) + geom_point(aes(x = speed.iqr_cor, y = speed.iqr_unc)) +
    geom_abline(intercept = 0, slope = 1) + theme_classic()
```

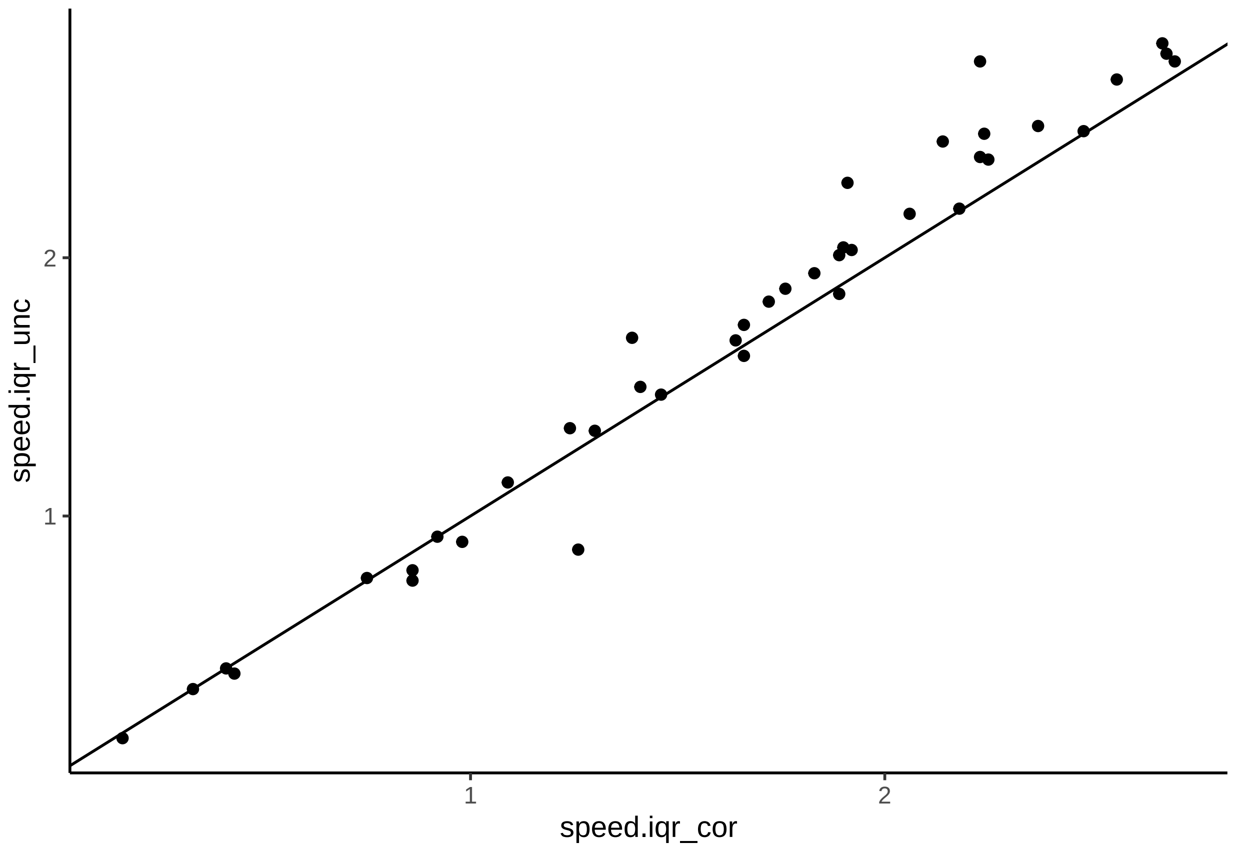


```r
ggplot(dataDay) + geom_point(aes(x = propmoving_cor, y = propmoving_unc)) +
    geom_abline(intercept = 0, slope = 1) + theme_classic()
```

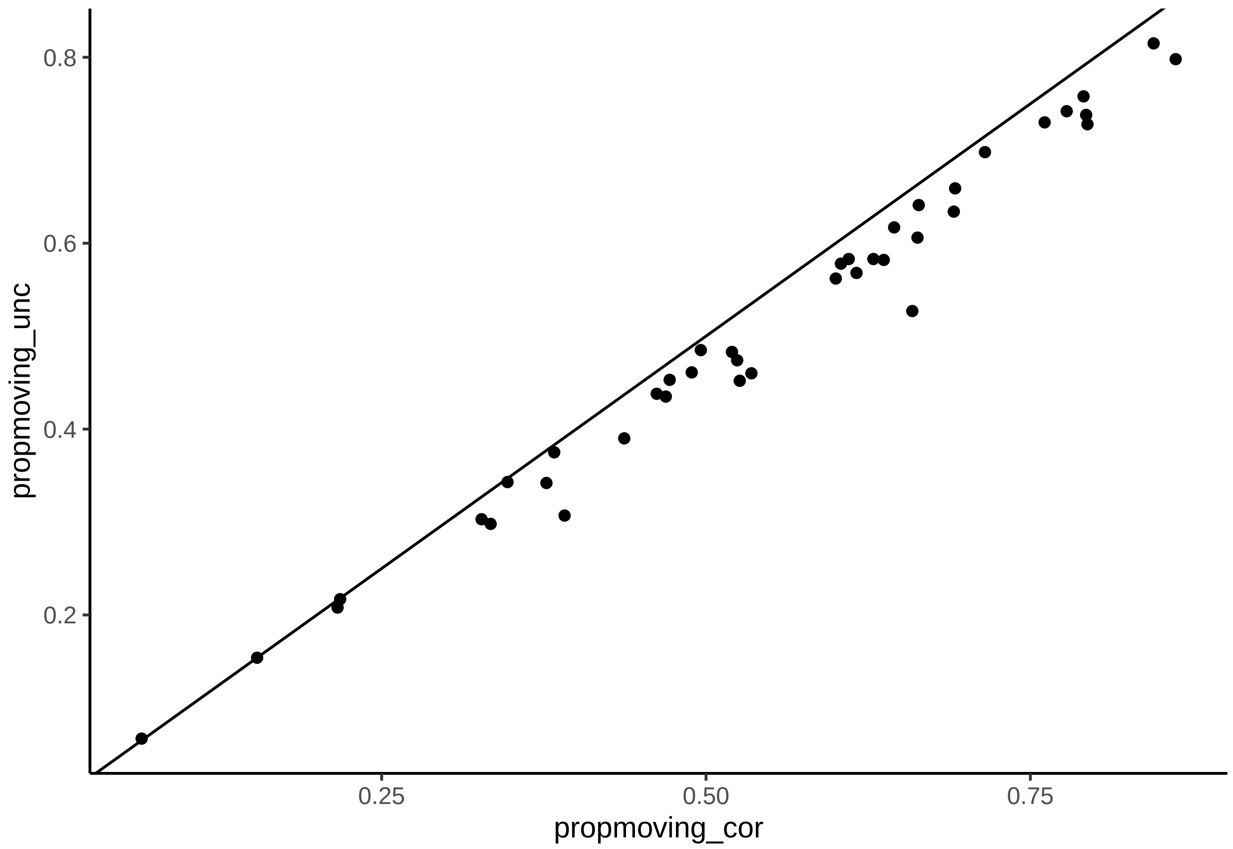


```r
ggplot(dataDay) + geom_point(aes(x = borderdist.med_cor, y = borderdist.med_unc)) +
    geom_abline(intercept = 0, slope = 1) + theme_classic()
```

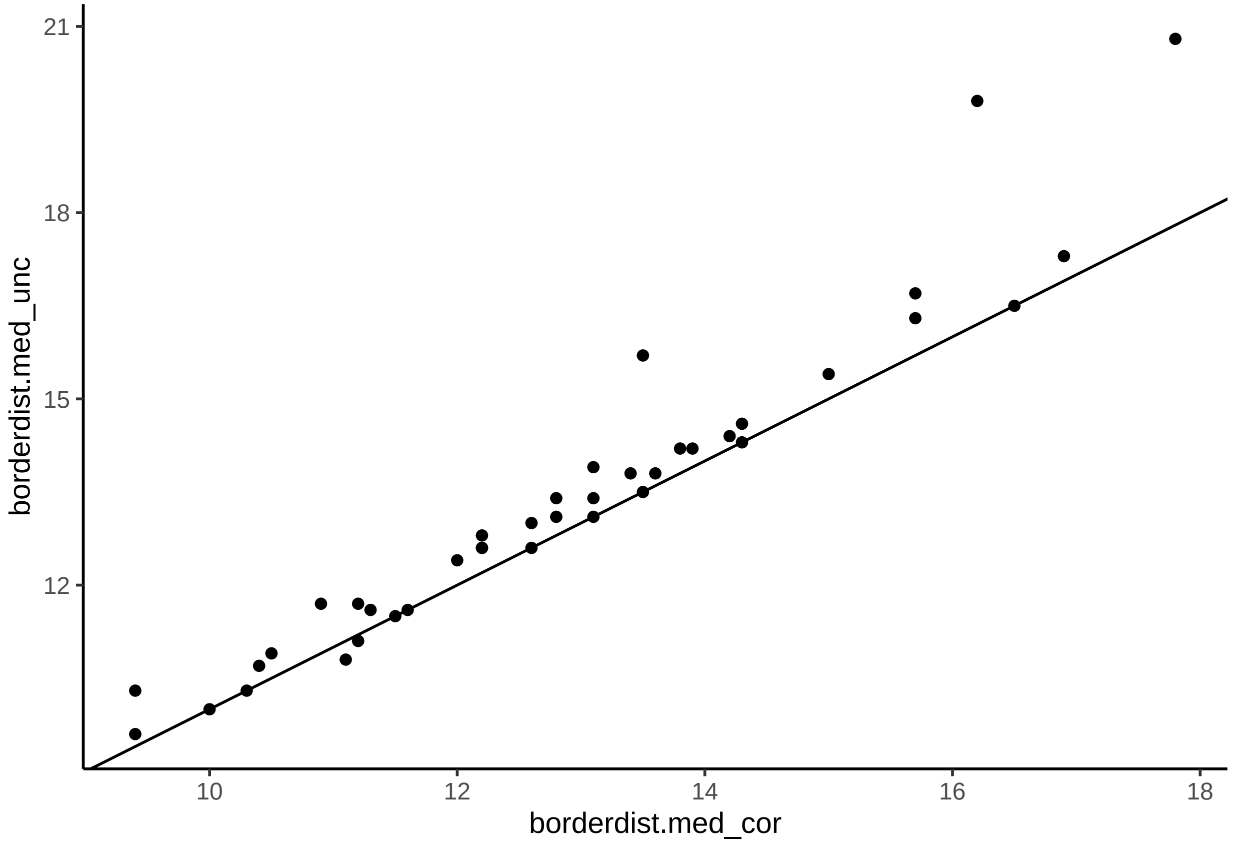


```
ggplot(dataDay) + geom_point(aes(x = se.pos_cor, y = se.pos_unc)) +
    geom_abline(intercept = 0, slope = 1) + theme_classic()
```

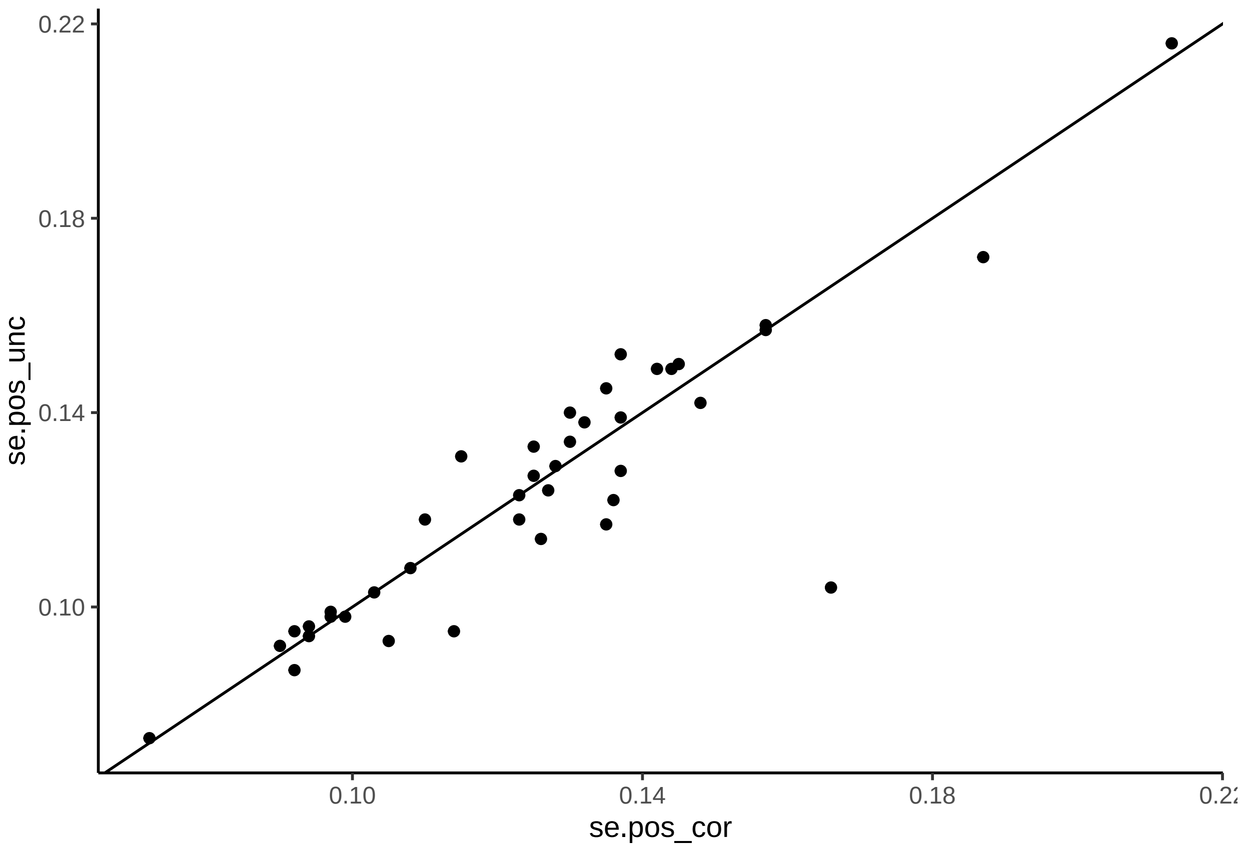

Now, the most important questions we want to answer are:

1 - how tight is the relationship between corrected and uncorrected values across individuals?

2 - do both types of data recreate similar results in terms of variance decomposition?

If the relationship between corrected and uncorrected data is very high and the resulting variance component estimates from both types of data very similar, then we can feel confident that any tracking errors are introducing true 'noise' into the system and are not systematically influencing our results or their interpretation.

Here we will use the day 1 data that has been binned into 15 minute increments so we can see how tight the relationships are throughout the first day.

Bottom line:

```
rpt.mcmc <- function(x) {
    var.e <- x$VCV[, "units"]
    var.a <- x$VCV[, "picomp"]
    postR <- var.a/(var.a + var.e)
    return(c(posterior.mode(postR), posterior.mode(var.a), posterior.mode(var.e)))
}

bivar.cor <- function(x) {
    var.x <- x$VCV[, 1]
    var.y <- x$VCV[, 4]
    cov.xy <- x$VCV[, 2]
    post.cor <- cov.xy/(sqrt(var.x * var.y))
    return(posterior.mode(post.cor))
}
```

```r
prior.ex <- list(R = list(V = 1, nu = 0.002), G = list(G1 = list(V = 1,
    nu = 0.002, alpha.mu = 0, alpha.V = 25^2)))

prior.bi <- list(R = list(V = diag(2), nu = 0.002), G = list(G1 = list(V = diag(2),
    nu = 0.002, alpha.mu = c(0, 0), alpha.V = diag(2) * 1000)))

# repeatability of uncorrected data
spd.unc <- MCMCglmm(speed.med_unc ~ obs, random = ~picomp, family = "gaussian",
    prior = prior.ex, data = dataWide, nitt = 201000, burnin = 1000,
    thin = 100, verbose = FALSE)

# repeatability of corrected data
spd.cor <- MCMCglmm(speed.med_cor ~ obs, random = ~picomp, family = "gaussian",
    prior = prior.ex, data = dataWide, nitt = 201000, burnin = 1000,
    thin = 100, verbose = FALSE)

# what is the covariance between the two?
spd.bi <- MCMCglmm(cbind(speed.med_unc, speed.med_cor) ~ obs,
    random = ~us(trait):picomp, rcov = ~us(trait):units, prior = prior.bi,
    data = dataWide, family = c("gaussian", "gaussian"), nitt = 201000,
    burnin = 1000, thin = 100, verbose = FALSE)
```

We can see that variance component estimates (repeatability, among-indivdual and within-individual variation) are very similar for both the manually corrected and automatically tracked (uncorrected) data, and that the among-individual correlation is nearly perfect! This is the case for median speed which is our main behavioral variable of interest, and also the other behavioral measures (e.g. distmoved.iqr, propmoving)

```r
rpt.mcmc(spd.unc)
```

```
##      var1      var1      var1
## 0.6989191 0.4310367 0.1934130
```

```r
rpt.mcmc(spd.cor)
```

```
##      var1      var1      var1
## 0.6907005 0.4074563 0.1931337
```

```r
bivar.cor(spd.bi)
```

```
##      var1
## 0.9898591
```

```r
# repeatability of uncorrected data
dist.unc <- MCMCglmm(distmoved.iqr_unc ~ obs, random = ~picomp,
    family = "gaussian", prior = prior.ex, data = dataWide, nitt = 201000,
    burnin = 1000, thin = 100, verbose = FALSE)

# repeatability of corrected data
dist.cor <- MCMCglmm(distmoved.iqr_cor ~ obs, random = ~picomp,
    family = "gaussian", prior = prior.ex, data = dataWide, nitt = 201000,
```

```
    burnin = 1000, thin = 100, verbose = FALSE)

# what is the covariance between the two?
dist.bi <- MCMCglmm(cbind(distmoved.iqr_unc, distmoved.iqr_cor) ~
    obs, random = ~us(trait):picomp, rcov = ~us(trait):units,
    prior = prior.bi, data = dataWide, family = c("gaussian",
        "gaussian"), nitt = 201000, burnin = 1000, thin = 100,
    verbose = FALSE)

rpt.mcmc(dist.unc)
```

```
##       var1       var1       var1
##   0.614115 379.793966 257.187858
```

```
rpt.mcmc(dist.cor)
```

```
##        var1        var1        var1
##   0.6295948 353.5827462 229.4252594
```

```
bivar.cor(dist.bi)
```

```
##      var1
## 0.9930411
```

```
# repeatability of uncorrected data
prop.unc <- MCMCglmm(propmoving_unc ~ obs, random = ~picomp,
    family = "gaussian", prior = prior.ex, data = dataWide, nitt = 201000,
    burnin = 1000, thin = 100, verbose = FALSE)

# repeatability of corrected data
prop.cor <- MCMCglmm(propmoving_cor ~ obs, random = ~picomp,
    family = "gaussian", prior = prior.ex, data = dataWide, nitt = 201000,
    burnin = 1000, thin = 100, verbose = FALSE)

# what is the covariance between the two?
prop.bi <- MCMCglmm(cbind(propmoving_unc, propmoving_cor) ~ obs,
    random = ~us(trait):picomp, rcov = ~us(trait):units, prior = prior.bi,
    data = dataWide, family = c("gaussian", "gaussian"), nitt = 201000,
    burnin = 1000, thin = 100, verbose = FALSE)

rpt.mcmc(prop.unc)
```

```
##       var1       var1       var1
## 0.69749477 0.03596995 0.01504533
```

```
rpt.mcmc(prop.cor)
```

```
##       var1       var1       var1
## 0.68119157 0.03445545 0.01872111
```

```
bivar.cor(prop.bi)
```

```
##      var1
## 0.9878863
```

> **Bottom line:**

There is a near-perfect among-individual correlation between manually corrected and automatically tracked (uncorrected data) and patterns of individual variation (among/within-individual variation) are extremely similar giving us confidence that our automatically tracked data accurately captures the true behavioral variation in the fish. Despite this, we still retain the manually corrected data for Day 1 as this is a critical time point for our research questions.

Supplemental Note II - Code to reproduce results

K Laskowski

Sept 29, 2022

DATA COLLECTION

Newborn mollies were placed into individual identical tanks and observed using overhead Raspberry Pi computer & cameras that took a photo every 3 seconds during daylight hours. From these photos, we used Biotracker to identify the fish and record its x-y coordinates. Then from these coordinates we estimated a number of behavioral variables.

Importantly, because one of our main research questions focused on behavior during the first day of life, we manually checked/corrected Day 1 tracks for all fish. This had the added bonus of letting us compare how well Biotracker performs (in terms of accurately measuring behavior) in comparison to manually tracking (which we know is "perfect")

- speed.med: median of distance moved (mm) /number of tracking frames (corrects for missing data)
- speed.iqr: inter-quartile range of distance moved/number of tracking frames (corrects for missing data)
- propmoving: propotion of the day that fish were moving at more than 0.5cm/s
- borderdist.med: median border distance from the tank edge across the day

We then summarized these behavioral variables either hourly or daily and used these data to investigate patterns of individual behavioral variation. Specifically we asked 3 key research questions (below)

RESEARCH QUESTIONS

1. When do individual differences in behavior emerge?

- test for significant rpt on Day 1
- if not apparent then, will look later in time

2. How does individual variation change over development?

- does among-individual variation increase over time - behavioral divergence?
- does within-individual variation decrease over time - behavioral consistency?

3. Does individual behavior early in life predict behavior later in life?

- test for significant among-individual correlations between week 1 and week n

DATA INPUT —-

```r
knitr::opts_chunk$set(tidy.opts = list(width.cutoff = 75), tidy = TRUE)

library(nlme)
library(ggplot2)
library(dplyr)
```

```
##
## Attaching package: 'dplyr'

## The following object is masked from 'package:nlme':
##
##     collapse

## The following objects are masked from 'package:stats':
##
##     filter, lag

## The following objects are masked from 'package:base':
##
##     intersect, setdiff, setequal, union
```

```r
library(tidyr)
library(MCMCglmm)
```

```
## Loading required package: Matrix

##
## Attaching package: 'Matrix'

## The following objects are masked from 'package:tidyr':
##
##     expand, pack, unpack

## Loading required package: coda

## Loading required package: ape
```

```r
library(parallel)
library(reshape2)
```

```
##
## Attaching package: 'reshape2'

## The following object is masked from 'package:tidyr':
##
##     smiths
```

```r
library(corrplot)
```

```
## corrplot 0.92 loaded
```

```r
library(MuMIn)
library(gridExtra)
```

```
##
## Attaching package: 'gridExtra'

## The following object is masked from 'package:dplyr':
##
##     combine
```

```r
library(ggcorrplot)
library(zoo)
```

```
##
## Attaching package: 'zoo'

## The following objects are masked from 'package:base':
##
##     as.Date, as.Date.numeric
```

```r
library(egg)
library(patchwork)
library(viridis)
```

```
## Loading required package: viridisLite
```

```r
library(formatR)

setwd("C:/Users/katel/Dropbox/7_Molly_Power/FINAL/analysis/Individual analysis/individual_molly_develop

# daily summary data
indv <- read.csv("IDsumdat_daily only_for deposit.csv")
indv$date <- as.Date(indv$date, format = "%m/%d/%Y")
indv$birth.date <- as.Date(indv$birth.date, format = "%m/%d/%Y")
indv$mother <- as.factor(indv$mother)
indv$picomp <- as.factor(indv$picomp)

# hourly summary data
hourly <- read.csv("IDsumdat_hourly only_for deposit.csv")
hourly$date <- as.Date(hourly$date, format = "%m/%d/%Y")
hourly$birth.date <- as.Date(hourly$birth.date, format = "%m/%d/%Y")
hourly$mother <- as.factor(hourly$mother)
hourly$picomp <- as.factor(hourly$picomp)

#create datafiles that only include the 26 indv with complete data through 70
complete <- as.vector(indv$picomp[indv$obs == 70])
```

```
indv.com <- indv %>%
  mutate(obs.cen = obs-1) %>%
  filter(picomp %in% complete)

# generating weekly growth rates:
growth <- indv.com %>%
  group_by(picomp, week) %>%
  arrange(picomp, week) %>%
  slice(1) %>%
  ungroup() %>%
  mutate(growth.rate = (TL - lag(TL))/lag(TL)) %>%
  mutate(growth.rate = round(growth.rate, 3)) %>%
  naniar::replace_with_na_at(.vars = "growth.rate",
                   condition = ~.x < 0) %>%
  mutate_at(c("growth.rate"), funs(lead), n = 1) %>%
  select(picomp, week, growth.rate)
```

```
## Warning: `funs()` was deprecated in dplyr 0.8.0.
## Please use a list of either functions or lambdas:
##
##   # Simple named list:
##   list(mean = mean, median = median)
##
##   # Auto named with `tibble::lst()`:
##   tibble::lst(mean, median)
##
##   # Using lambdas
##   list(~ mean(., trim = .2), ~ median(., na.rm = TRUE))
## This warning is displayed once every 8 hours.
## Call `lifecycle::last_lifecycle_warnings()` to see where this warning was generated.
```

```
#generate Day1 only data
#re-center hour (so 1st hour = 0)
day1 <- hourly %>%
  filter(obs == 1) %>%
  mutate(hour.cen = hour-1)

day1 <- left_join(day1, growth)
```

```
## Joining, by = c("picomp", "week")
```

```
indv.com <- left_join(indv.com, growth)
```

```
## Joining, by = c("picomp", "week")
```

```
hourly.com <- hourly %>%
  mutate(obs.cen = obs-1) %>%
  filter(picomp %in% complete)
hourly.com <- left_join(hourly.com, growth)
```

```
## Joining, by = c("picomp", "week")
```

```r
day1.com <- day1 %>%
  filter(picomp %in% complete) %>%
  mutate(TL.cen = TL - (sum(unique(TL))/26))
```

Prior specifications

```r
prior.null <- list(R = list(V = 1, nu = 0.002))

prior.id <- list(R = list(V = 1, nu = 0.002), G = list(G1 = list(V = 1, nu = 0.002,
    alpha.mu = 0, alpha.V = 25^2)))

prior.id.mom <- list(R = list(V = 1, nu = 0.002), G = list(G1 = list(V = 1,
    nu = 0.002, alpha.mu = 0, alpha.V = 25^2), G2 = list(V = 1, nu = 0.002,
    alpha.mu = 0, alpha.V = 25^2)))

prior.id.slope <- list(R = list(V = 1, nu = 0.002), G = list(G1 = list(V = diag(2),
    nu = 0.002, alpha.mu = c(0, 0), alpha.V = diag(2) * 25^2)))

prior.id.slope.mom <- list(R = list(V = 1, nu = 0.002), G = list(G1 = list(V = diag(2),
    nu = 0.002, alpha.mu = c(0, 0), alpha.V = diag(2) * 25^2), G2 = list(V = 1,
    nu = 0.002, alpha.mu = 0, alpha.V = 25^2)))

prior.id.slope.mom.slope <- list(R = list(V = 1, nu = 1), G = list(G1 = list(V = diag(2) *
    0.1, nu = 0.002, alpha.mu = c(0, 0), alpha.V = diag(2) * 25^2), G2 = list(V = diag(2) *
    0.1, nu = 0.002, alpha.mu = c(0, 0), alpha.V = diag(2) * 25^2)))

prior.cov4 <- list(R = list(V = diag(4), nu = 4.002), G = list(G1 = list(V = diag(4),
    nu = 4.002, alpha.mu = rep(0, 4), alpha.V = 1000 * diag(4))))


prior.cov10 <- list(R = list(V = diag(10), nu = 10.002), G = list(G1 = list(V = diag(10),
    nu = 10.002, alpha.mu = rep(0, 10), alpha.V = 1000 * diag(10))))
```

DATA EXPLORATION

Daily behavior

Can see pretty tight correlations between speed.med, propmoving and distmoved.iqr. Looks like borderdist.med more independent of these other measures.

```
pairs(indv[, c("speed.med", "propmoving", "borderdist.med", "distmoved.iqr")])
```

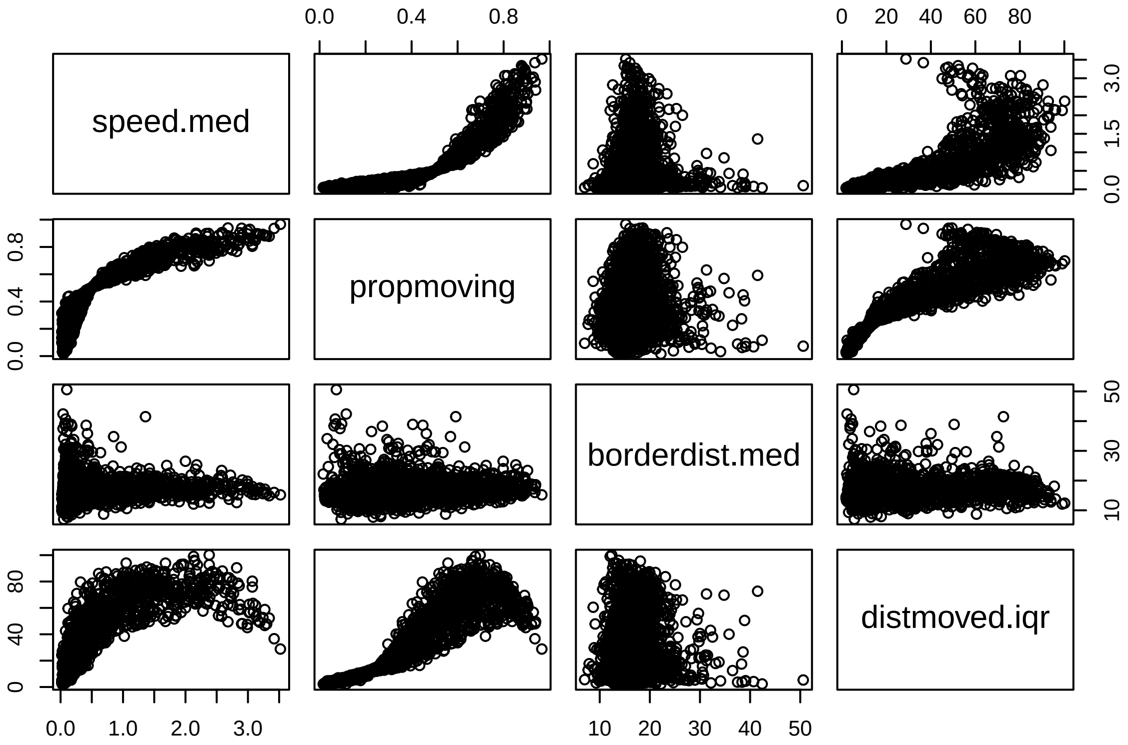


```
pairs(indv.com[, c("speed.med", "propmoving", "borderdist.med", "distmoved.iqr")])
```

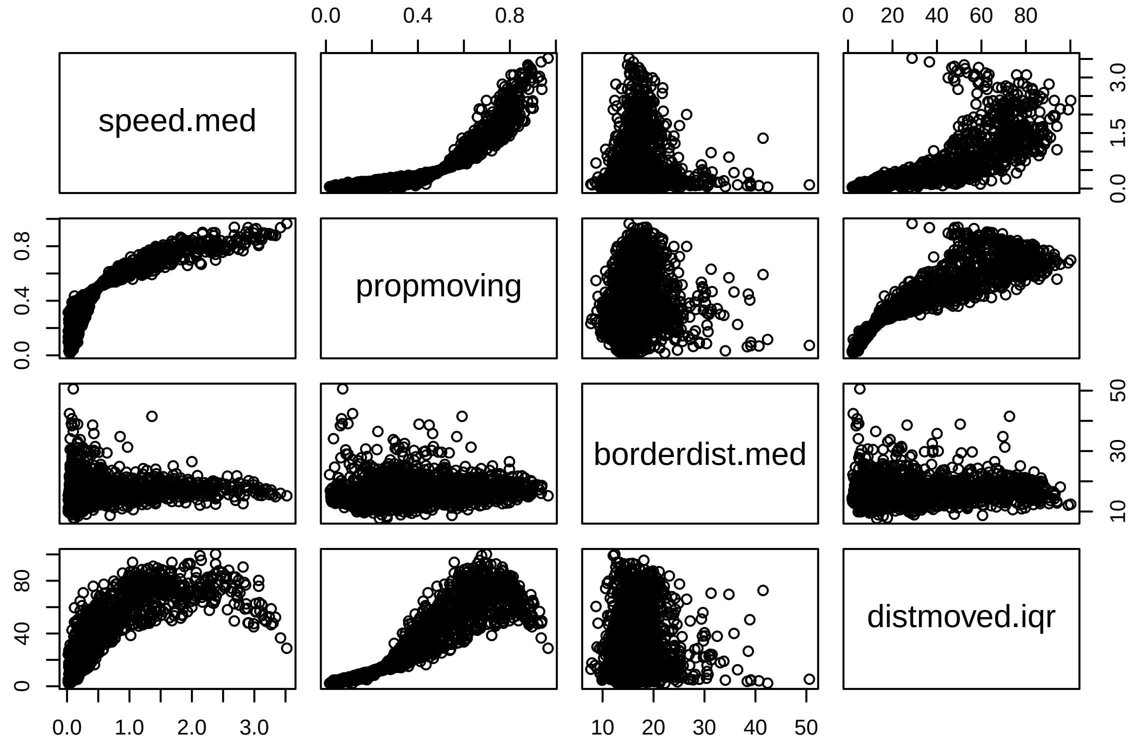

Hourly behavior

Also looks like good variation here, similar correlations among traits as with the daily measures.

```
pairs(day1[, c("speed.med", "propmoving", "borderdist.med", "distmoved.iqr")])
```

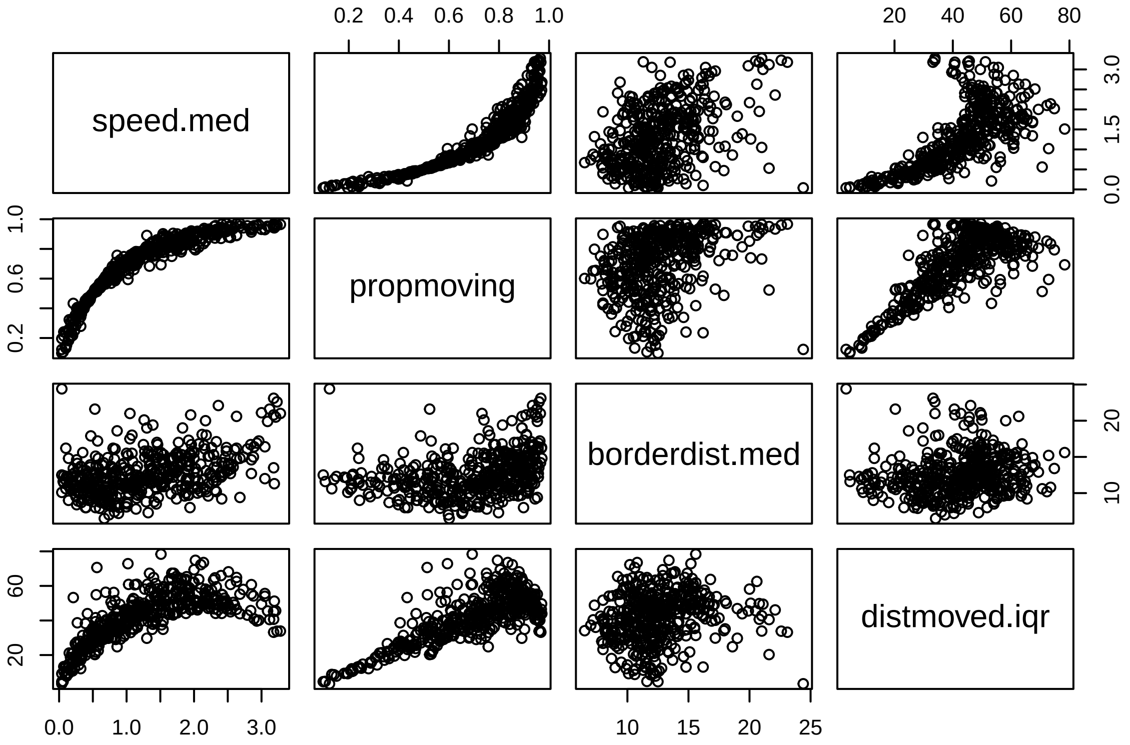


```r
pairs(day1.com[, c("speed.med", "propmoving", "borderdist.med", "distmoved.iqr")])
```

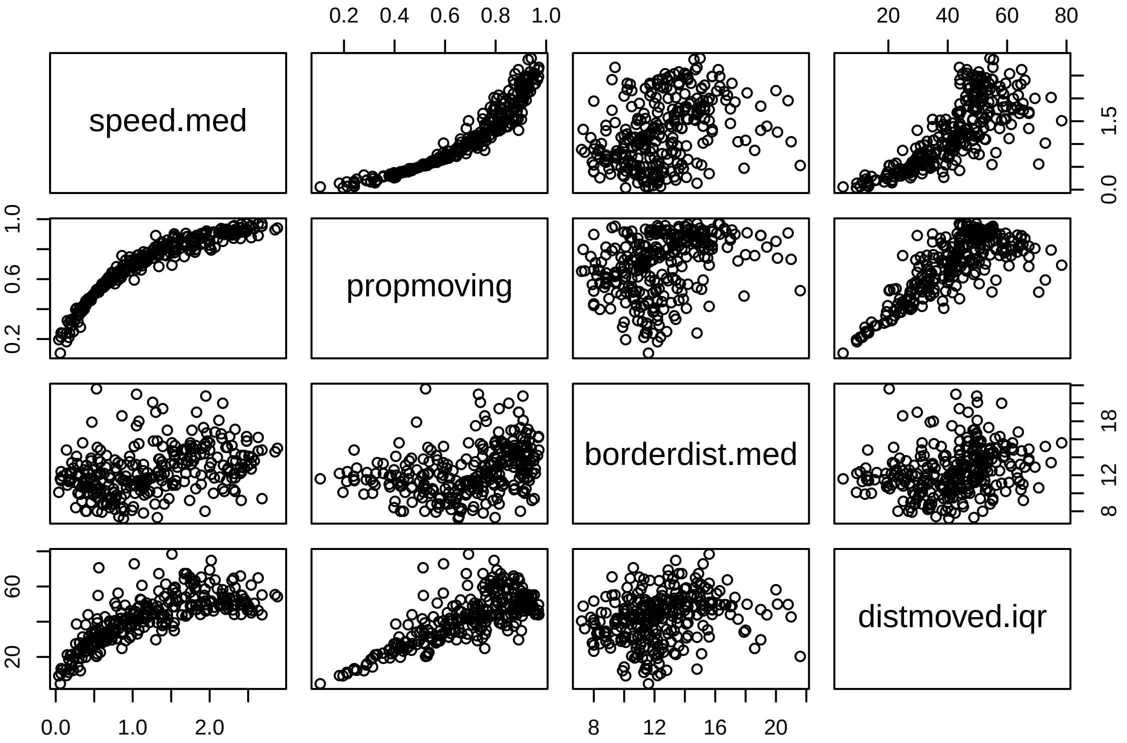


```
hist(day1.com$speed.med)
```

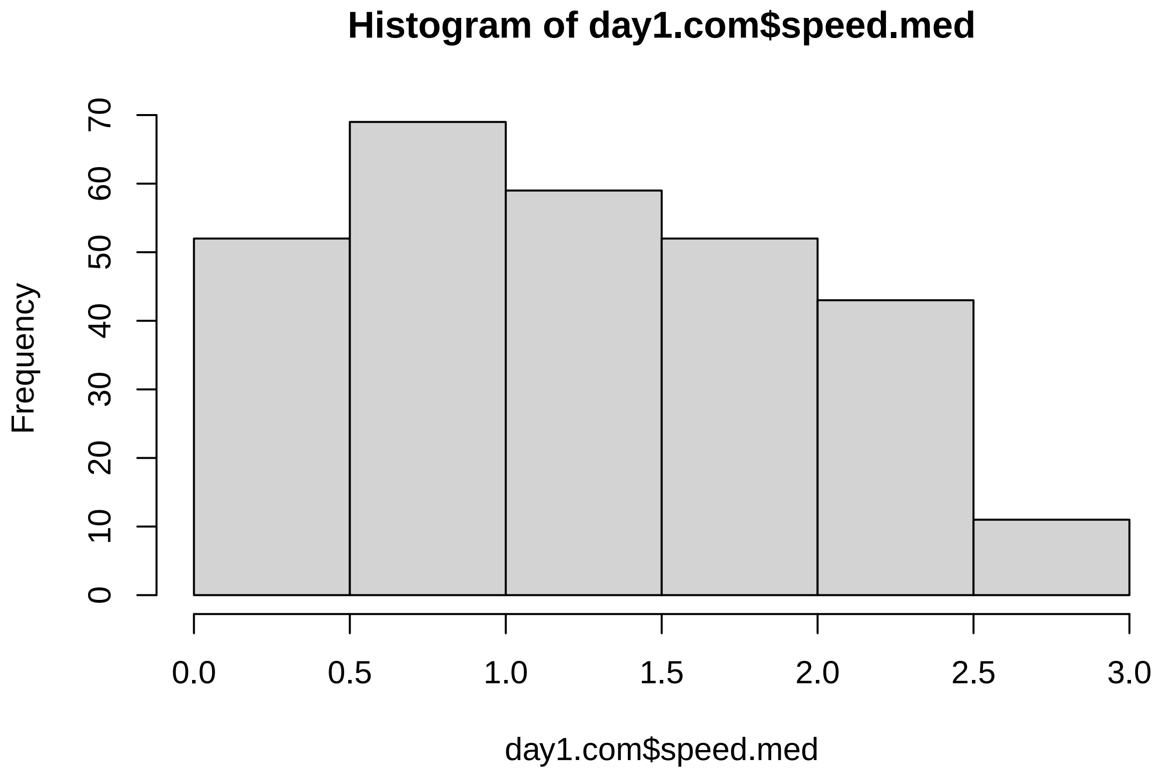


```
range(day1.com$speed.med)
```

```
## [1] 0.04 2.88
```

```
indv.com %>%
    group_by(mother) %>%
    summarise_each(funs(n_distinct(picomp)))
```

```
## Warning: `summarise_each_()` was deprecated in dplyr 0.7.0.
## Please use `across()` instead.
## This warning is displayed once every 8 hours.
## Call `lifecycle::last_lifecycle_warnings()` to see where this warning was generated.
```

```
## # A tibble: 8 x 21
##   mother   rpi  comp picomp  date  hour distmo~1 speed~2 speed~3 propm~4 borde~5
##   <fct>  <int> <int>  <int> <int> <int>    <int>   <int>   <int>   <int>   <int>
## 1 69.3       4     4      4     1     1        1       1       1       1       1
## 2 70.2       4     4      4     1     1        1       1       1       1       1
## 3 70.3       4     4      4     1     1        1       1       1       1       1
## 4 75.2       4     4      4     1     1        1       1       1       1       1
## 5 77.2       3     3      3     1     1        1       1       1       1       1
## 6 80.1       3     3      3     1     1        1       1       1       1       1
## 7 80.2       3     3      3     1     1        1       1       1       1       1
## 8 90.2       1     1      1     1     1        1       1       1       1       1
```

```
## # ... with 10 more variables: borderdist.mean <int>, first_obs <int>,
## #   last_obs <int>, obs <int>, week <int>, brood.size <int>, birth.date <int>,
## #   TL <int>, obs.cen <int>, growth.rate <int>, and abbreviated variable names
## #   1: distmoved.iqr, 2: speed.med, 3: speed.mean, 4: propmoving,
## #   5: borderdist.med
## # i Use `colnames()` to see all variable names
```

1 - WHEN DO INDIVDIUAL DIFFERENCES APPEAR?

Our goal here is, starting with Day 1, determine if/when significant individual differences in behavior appear.

But first, we explored which variance structures are the best fit for our data. In total we ran 5 models with differing variance structures model 0: null model (no random effects) model 1: individual intercepts model 2: individual & mother intercepts model 3: individual intercepts & slopes model 4: individual intercepts & slopes and mother intercepts model 5: individual intercepts & slopes and mother intercepts & slopes

We included hour (first hour of the day set to 0) and SL (mean-centered) as fixed effects in all models.

RESULTS: Strong support for inclusion of individual intercepts and slopes. Since hour is centered (with first hour as 0) then the estimate of among-individual variation is in fact for the first hour of tracking. Which means that as early as we can measure bheavior individual differences are present on (the first hour of) day 1! There are very small effects of hour and none of body size (NB: can only use absolute TL in these models as the animals do not yet have a growth rate).

Day 1- swim speed: First, want to check residuals of model to determine if assumptions of gaussian error distribution are appropriate - Yes, the residuals look great!

```
lm.test <- lme(speed.med ~ hour.cen + TL.cen, random = ~1 | picomp, data = day1.com)

par(mfrow = c(2, 2))
plot(lm.test)
```

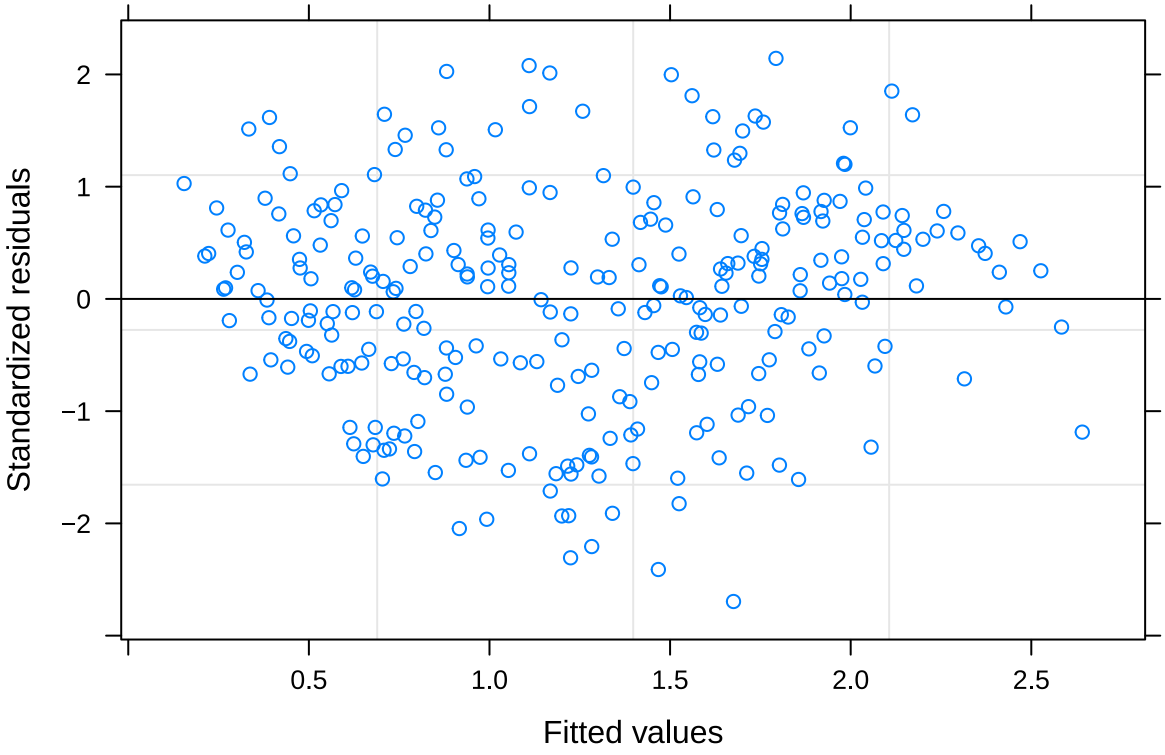


```
hist(resid(lm.test))
qqnorm(resid(lm.test))
qqline(resid(lm.test))
boxplot(resid(lm.test) ~ day1.com$hour.cen)
plot(resid(lm.test) ~ day1.com$TL.cen)
```

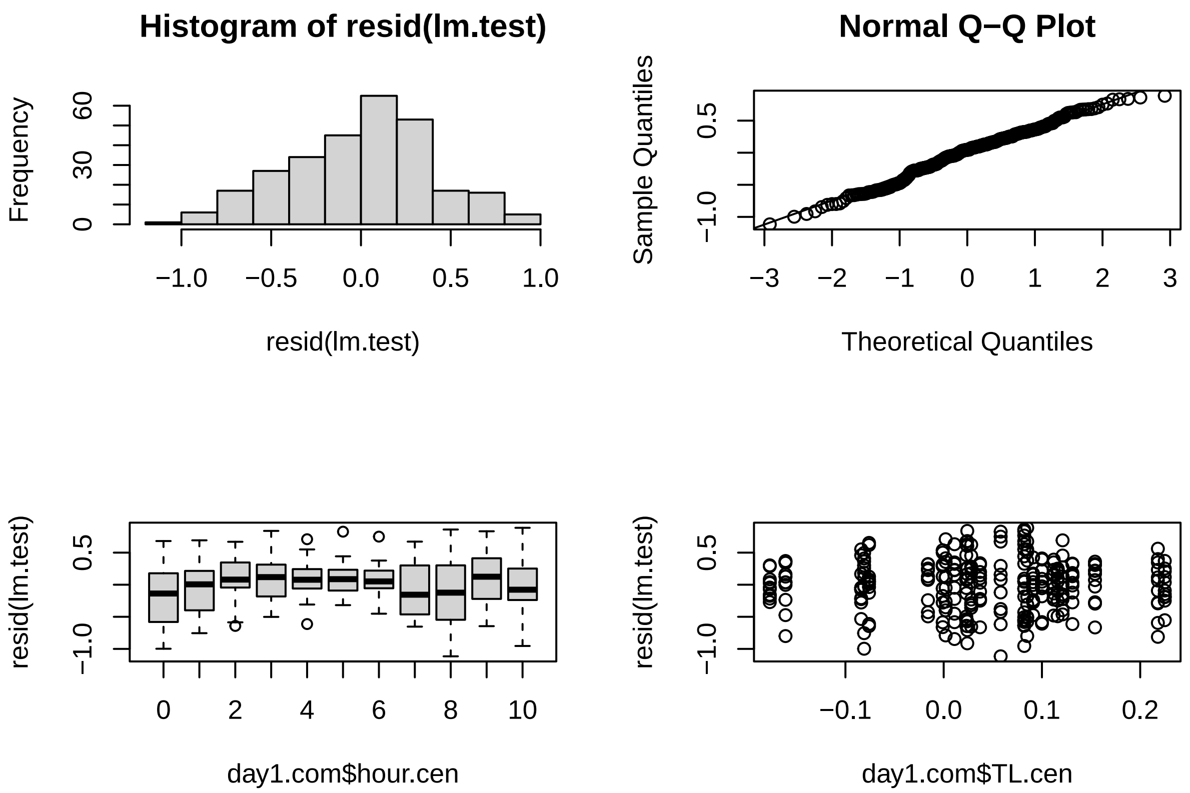

> **model comparison** Now we want to investigate the appropriate random structure for the data including intercepts and/or slopes for individuals and mothers

```
# Null model -----------
set.seed(58)
speed.day1.0 <- MCMCglmm(speed.med ~ hour.cen + TL.cen, data = day1.com, family = "gaussian",
    prior = prior.null, nitt = 510000, burnin = 10000, thin = 200, verbose = F)


# Intercepts ID ----------------------
set.seed(3432)
speed.day1.1 <- MCMCglmm(speed.med ~ hour.cen + TL.cen, random = ~picomp, data = day1.com,
    family = "gaussian", prior = prior.id, nitt = 510000, burnin = 10000, thin = 200,
    verbose = F)

# only including intercepts for mother ------
set.seed(42021)
speed.day1.2 <- MCMCglmm(speed.med ~ hour.cen + TL.cen, random = ~mother, data = day1.com,
    family = "gaussian", prior = prior.id, nitt = 510000, burnin = 10000, thin = 200,
    verbose = F)


# Intercepts ID and Mom-----------
set.seed(258)
speed.day1.3 <- MCMCglmm(speed.med ~ hour.cen + TL.cen, random = ~picomp + mother,
```

```r
    data = day1.com, family = "gaussian", prior = prior.id.mom, nitt = 510000,
    burnin = 10000, thin = 200, verbose = F)


# Intercepts and Slopes ID -----------------------
set.seed(472)
speed.day1.4 <- MCMCglmm(speed.med ~ hour.cen + TL.cen, random = ~us(1 + hour.cen):picomp,
    data = day1.com, family = "gaussian", prior = prior.id.slope, nitt = 510000,
    burnin = 10000, thin = 200, verbose = F)

# Intercepts Mom, Slopes Mom ----------------
set.seed(318)
speed.day1.5 <- MCMCglmm(speed.med ~ hour.cen + TL.cen, random = ~us(1 + hour.cen):mother,
    data = day1.com, family = "gaussian", prior = prior.id.slope, nitt = 510000,
    burnin = 10000, thin = 200, verbose = F)


# Intercepts ID and Mom, Slopes ID ----------------
set.seed(918)
speed.day1.6 <- MCMCglmm(speed.med ~ hour.cen + TL.cen, random = ~us(1 + hour.cen):picomp +
    mother, data = day1.com, family = "gaussian", prior = prior.id.slope.mom,
    nitt = 510000, burnin = 10000, thin = 200, verbose = F)


# Intercepts ID & Mom, Slopes ID & Mom -----------------
set.seed(78)
speed.day1.7 <- MCMCglmm(speed.med ~ hour.cen + TL.cen, random = ~us(1 + hour.cen):picomp +
    us(1 + hour.cen):mother, data = day1.com, family = "gaussian", prior = prior.id.slope.mom.slope,
    nitt = 510000, burnin = 10000, thin = 200, verbose = F)
```

Including individual intercepts and slopes dramatically improves the DIC of the models. Including mother intercepts or slopes really has no effect so those should be dropped. Since individual slopes seem to be important, then it is important that we appropriately center our covariates (hour 1 = 0 and average body size = 0) so that the variance (among/within individual) components are being estimated here as this makes the most biological sense.

```r
DIC(speed.day1.0, speed.day1.1, speed.day1.2, speed.day1.3, speed.day1.4, speed.day1.5,
    speed.day1.6, speed.day1.7)
```

```
##              df      DIC
## speed.day1.0  4 611.9636
## speed.day1.1  5 335.0621
## speed.day1.2  5 548.3438
## speed.day1.3  6 335.0658
## speed.day1.4  6 234.6791
## speed.day1.5  6 543.7717
## speed.day1.6  7 234.5784
## speed.day1.7  8 236.7037
```

> model to report These are the estimates for the most well supported model (including individual intercepts and slopes, model 3). These are the values reported in the main manuscript

```r
set.seed(472)
speed.day1.4 <- MCMCglmm(speed.med ~ hour.cen + TL.cen, random = ~us(1 + hour.cen):picomp,
    prior = prior.id.slope, data = day1.com, family = "gaussian", nitt = 510000,
    burnin = 10000, thin = 200, verbose = F)
```

```r
summary(speed.day1.4)
```

```
##
##  Iterations = 10001:509801
##  Thinning interval  = 200
##  Sample size  = 2500
##
##  DIC: 234.6791
##
##  G-structure:  ~us(1 + hour.cen):picomp
##
##                                  post.mean  l-95% CI u-95% CI eff.samp
## (Intercept):(Intercept).picomp 2.401e-01  0.096683  0.41582     2500
## hour.cen:(Intercept).picomp    4.972e-05 -0.023651  0.01831     2500
## (Intercept):hour.cen.picomp    4.972e-05 -0.023651  0.01831     2500
## hour.cen:hour.cen.picomp       6.677e-03  0.002659  0.01151     2500
##
##  R-structure:  ~units
##
##       post.mean l-95% CI u-95% CI eff.samp
## units    0.1135  0.09501    0.136     2325
##
##  Location effects: speed.med ~ hour.cen + TL.cen
##
##             post.mean l-95% CI u-95% CI eff.samp  pMCMC
## (Intercept)   0.89681  0.66903  1.12809     2175 <4e-04 ***
## hour.cen      0.05747  0.02528  0.09378     2500 0.0008 ***
## TL.cen        1.48869 -0.52690  3.39880     2725 0.1384
## ---
## Signif. codes:  0 '***' 0.001 '**' 0.01 '*' 0.05 '.' 0.1 ' ' 1
```

```r
posterior.mode(speed.day1.4$Sol)
```

```
## (Intercept)    hour.cen      TL.cen
##  0.86823920  0.05755465  1.49292296
```

```r
HPDinterval(speed.day1.4$Sol)
```

```
##                   lower      upper
## (Intercept)  0.66903167 1.12808944
## hour.cen     0.02528032 0.09377976
## TL.cen      -0.52690036 3.39879682
## attr(,"Probability")
## [1] 0.95
```

```r
posterior.mode(speed.day1.4$VCV)
```

```
## (Intercept):(Intercept).picomp    hour.cen:(Intercept).picomp
##                    0.1750928818                   0.0007295398
##    (Intercept):hour.cen.picomp       hour.cen:hour.cen.picomp
##                    0.0007295398                   0.0060554490
##                           units
##                    0.1073052468
```

```r
HPDinterval(speed.day1.4$VCV)
```

```
##                                        lower      upper
## (Intercept):(Intercept).picomp  0.096683120 0.41582272
## hour.cen:(Intercept).picomp    -0.023651140 0.01831348
## (Intercept):hour.cen.picomp    -0.023651140 0.01831348
## hour.cen:hour.cen.picomp        0.002658925 0.01151310
## units                           0.095014047 0.13599975
## attr(,"Probability")
## [1] 0.95
```

```r
# only ID repeatability
rpt.spd1 <- speed.day1.4$VCV[, "(Intercept):(Intercept).picomp"]/(speed.day1.4$VCV[,
    "(Intercept):(Intercept).picomp"] + speed.day1.4$VCV[, "hour.cen:hour.cen.picomp"] +
    speed.day1.4$VCV[, "units"])
posterior.mode(rpt.spd1)
```

```
##      var1
## 0.6505839
```

```r
HPDinterval(rpt.spd1)
```

```
##          lower     upper
## var1 0.4841208 0.8007834
## attr(,"Probability")
## [1] 0.95
```

```r
# to get marginal R2 to explain fixed effects variance
vmVarF <- numeric(2500)

for (i in 1:2500) {
    Var <- var(as.vector(speed.day1.4$Sol[i, ] %*% t(speed.day1.4$X)))
    vmVarF[i] <- Var
}


R2m <- vmVarF/(vmVarF + speed.day1.4$VCV[, 1] + speed.day1.4$VCV[, 4] + speed.day1.4$VCV[,
    5])

posterior.mode(R2m)
```

```
##      var1
## 0.1334692
```

```
HPDinterval(R2m)
```

```
##           lower     upper
## var1 0.02089727 0.3040472
## attr(,"Probability")
## [1] 0.95
```

```
R2c <- (vmVarF + speed.day1.4$VCV[, 1])/(vmVarF + speed.day1.4$VCV[, 1] + speed.day1.4$VCV[,
    4] + speed.day1.4$VCV[, 5])

posterior.mode(R2c)
```

```
##      var1
## 0.7441373
```

```
HPDinterval(R2c)
```

```
##          lower     upper
## var1 0.5690082 0.8255776
## attr(,"Probability")
## [1] 0.95
```

```
# extract estimates for later graphing

tl.slope <- as.vector(speed.day1.4$Sol[, "TL.cen"])
tl.inter <- as.vector(speed.day1.4$Sol[, "(Intercept)"])
body.effects <- data.frame(tl.slope, tl.inter)
```

> model validation Just want to double check that the results we're seeing here aren't a result of prior specification. The results above are all with parameter expanded priors, so I will also check weak priors and then two different stronger priors where I partition the variance to the different random effects.

You can see that even putting the majority of the behavioral variance in the residual does not really alter the variance component estimates. Therefore we feel confident using the parameter expanded priors throughout.

```
# parameter expanded
prior.id.slope <- list(R = list(V = 1, nu = 0.002), G = list(G1 = list(V = diag(2),
    nu = 0.002, alpha.mu = c(0, 0), alpha.V = diag(2) * 25^2)))


# weak priors (inverse gamma)
prior.weak <- list(R = list(V = 1, nu = 0.002), G = list(G1 = list(V = diag(2),
    nu = 0.002)))

# strong priors
p.var <- var(day1.com$speed.med, na.rm = TRUE)

# setting most variance in among-indv intercepts (and a bit in slopes)
```

```
prior.strong.id <- list(R = list(V = matrix(p.var * 0.05), nu = 1), G = list(G1 = list(V = diag(c(0.8 *
    p.var, 0.15 * p.var)), nu = 1)))

# setting most variance in the within-indv residual and very little to
# among individual intercepts & slopes
prior.strong.res <- list(R = list(V = matrix(p.var * 0.95), nu = 1), G = list(G1 = list(V = diag(c(0.025
    p.var, 0.025 * p.var)), nu = 1)))


set.seed(472)
speed.day1.param <- MCMCglmm(speed.med ~ hour.cen + TL.cen, random = ~us(1 +
    hour.cen):picomp, prior = prior.id.slope, data = day1.com, family = "gaussian",
    nitt = 510000, burnin = 10000, thin = 200, verbose = F)

set.seed(472)
speed.day1.weak <- MCMCglmm(speed.med ~ hour.cen + TL.cen, random = ~us(1 +
    hour.cen):picomp, prior = prior.weak, data = day1.com, family = "gaussian",
    nitt = 510000, burnin = 10000, thin = 200, verbose = F)

set.seed(472)
speed.day1.strong1 <- MCMCglmm(speed.med ~ hour.cen + TL.cen, random = ~us(1 +
    hour.cen):picomp, prior = prior.strong.id, data = day1.com, family = "gaussian",
    nitt = 510000, burnin = 10000, thin = 200, verbose = F)

set.seed(472)
speed.day1.strong2 <- MCMCglmm(speed.med ~ hour.cen + TL.cen, random = ~us(1 +
    hour.cen):picomp, prior = prior.strong.res, data = day1.com, family = "gaussian",
    nitt = 510000, burnin = 10000, thin = 200, verbose = F)

posterior.mode(speed.day1.param$VCV)
```

```
## (Intercept):(Intercept).picomp    hour.cen:(Intercept).picomp
##                    0.1750928818                   0.0007295398
##    (Intercept):hour.cen.picomp       hour.cen:hour.cen.picomp
##                    0.0007295398                   0.0060554490
##                           units
##                    0.1073052468
```

```
posterior.mode(speed.day1.weak$VCV)
```

```
## (Intercept):(Intercept).picomp    hour.cen:(Intercept).picomp
##                     0.191066281                    0.001804820
##    (Intercept):hour.cen.picomp       hour.cen:hour.cen.picomp
##                     0.001804820                    0.005250233
##                           units
##                     0.111321479
```

```
posterior.mode(speed.day1.strong1$VCV)
```

```
## (Intercept):(Intercept).picomp    hour.cen:(Intercept).picomp
##                     0.214483238                    0.001288724
```

```
##    (Intercept):hour.cen.picomp       hour.cen:hour.cen.picomp
##                    0.001288724                    0.008149634
##                          units
##                    0.115134104
```

```
posterior.mode(speed.day1.strong2$VCV)
```

```
## (Intercept):(Intercept).picomp    hour.cen:(Intercept).picomp
##                    0.199585874                    0.001785059
##    (Intercept):hour.cen.picomp       hour.cen:hour.cen.picomp
##                    0.001785059                    0.005373721
##                          units
##                    0.115412200
```

```
# plot(speed.day1.param$VCV) plot(speed.day1.weak$VCV)
# plot(speed.day1.strong1$VCV) plot(speed.day1.strong2$VCV)
```

Then I'll also run five different chains of the model, setting different seeds to make sure that independent chains are coming up with the same answer!

And yes, we see that the variance components estimates (which are our main parameters of interest) are converged on very similar values.

```
set.seed(472)
speed.day1a <- MCMCglmm(speed.med ~ hour.cen + TL.cen, random = ~us(1 + hour.cen):picomp,
    prior = prior.id.slope, data = day1.com, family = "gaussian", nitt = 510000,
    burnin = 10000, thin = 200, verbose = F)

set.seed(9702)
speed.day1b <- MCMCglmm(speed.med ~ hour.cen + TL.cen, random = ~us(1 + hour.cen):picomp,
    prior = prior.id.slope, data = day1.com, family = "gaussian", nitt = 510000,
    burnin = 10000, thin = 200, verbose = F)

set.seed(130)
speed.day1c <- MCMCglmm(speed.med ~ hour.cen + TL.cen, random = ~us(1 + hour.cen):picomp,
    prior = prior.id.slope, data = day1.com, family = "gaussian", nitt = 510000,
    burnin = 10000, thin = 200, verbose = F)


set.seed(4)
speed.day1d <- MCMCglmm(speed.med ~ hour.cen + TL.cen, random = ~us(1 + hour.cen):picomp,
    prior = prior.id.slope, data = day1.com, family = "gaussian", nitt = 510000,
    burnin = 10000, thin = 200, verbose = F)


set.seed(30681)
speed.day1e <- MCMCglmm(speed.med ~ hour.cen + TL.cen, random = ~us(1 + hour.cen):picomp,
    prior = prior.id.slope, data = day1.com, family = "gaussian", nitt = 510000,
    burnin = 10000, thin = 200, verbose = F)

posterior.mode(speed.day1a$VCV)
```

```
## (Intercept):(Intercept).picomp    hour.cen:(Intercept).picomp
```

```
##                    0.1750928818                  0.0007295398
##    (Intercept):hour.cen.picomp      hour.cen:hour.cen.picomp
##                    0.0007295398                  0.0060554490
##                           units
##                    0.1073052468
```

```
posterior.mode(speed.day1b$VCV)
```

```
## (Intercept):(Intercept).picomp    hour.cen:(Intercept).picomp
##                     0.196560946                   0.002570999
##    (Intercept):hour.cen.picomp      hour.cen:hour.cen.picomp
##                     0.002570999                   0.005554283
##                           units
##                     0.108106205
```

```
posterior.mode(speed.day1c$VCV)
```

```
## (Intercept):(Intercept).picomp    hour.cen:(Intercept).picomp
##                     0.195244839                  -0.003806159
##    (Intercept):hour.cen.picomp      hour.cen:hour.cen.picomp
##                    -0.003806159                   0.005370003
##                           units
##                     0.112002054
```

```
posterior.mode(speed.day1d$VCV)
```

```
## (Intercept):(Intercept).picomp    hour.cen:(Intercept).picomp
##                     0.221080515                   0.003760566
##    (Intercept):hour.cen.picomp      hour.cen:hour.cen.picomp
##                     0.003760566                   0.005359790
##                           units
##                     0.108055349
```

```
posterior.mode(speed.day1e$VCV)
```

```
## (Intercept):(Intercept).picomp    hour.cen:(Intercept).picomp
##                     0.195477346                   0.002142067
##    (Intercept):hour.cen.picomp      hour.cen:hour.cen.picomp
##                     0.002142067                   0.005426571
##                           units
##                     0.112732864
```

```
# plot(speed.day1a$VCV) plot(speed.day1b$VCV) plot(speed.day1c$VCV)
# plot(speed.day1d$VCV) plot(speed.day1e$VCV)
```

> all 40 individuals Our analyses are generally restricted to those animals for which we have complete data on through week 10, but here we'll check and make sure that we find similar patterns of behavioral variation on Day 1 using all individuals that were inititally placed in the tracking tanks

ANSWER - yes, results are robust regardless of which subset of individuals we use

```r
day1 <- day1 %>%
    mutate(TL.cen = TL - (sum(unique(TL))/40))

set.seed(6987)
speed.day1.idA <- MCMCglmm(speed.med ~ hour.cen + TL.cen, random = ~us(1 + hour.cen):picomp,
    prior = prior.id.slope, data = day1, family = "gaussian", nitt = 510000,
    burnin = 10000, thin = 200, verbose = F)

summary(speed.day1.idA)
```

```
##
##  Iterations = 10001:509801
##  Thinning interval  = 200
##  Sample size  = 2500
##
##  DIC: 340.2339
##
##  G-structure:  ~us(1 + hour.cen):picomp
##
##                                  post.mean  l-95% CI u-95% CI eff.samp
## (Intercept):(Intercept).picomp  0.291537  0.156436 0.454494     2129
## hour.cen:(Intercept).picomp     0.005365 -0.009233 0.020706     2874
## (Intercept):hour.cen.picomp     0.005365 -0.009233 0.020706     2874
## hour.cen:hour.cen.picomp        0.005048  0.002324 0.007951     2270
##
##  R-structure:  ~units
##
##       post.mean l-95% CI u-95% CI eff.samp
## units    0.1084  0.09299    0.125     2500
##
##  Location effects: speed.med ~ hour.cen + TL.cen
##
##             post.mean l-95% CI u-95% CI eff.samp  pMCMC
## (Intercept)   0.90375  0.70706  1.10261     2500 <4e-04 ***
## hour.cen      0.05740  0.03360  0.08057     2500 <4e-04 ***
## TL.cen        1.73415  0.16072  3.43036     2500  0.048 *
## ---
## Signif. codes:  0 '***' 0.001 '**' 0.01 '*' 0.05 '.' 0.1 ' ' 1
```

```r
posterior.mode(speed.day1.idA$VCV)
```

```
## (Intercept):(Intercept).picomp    hour.cen:(Intercept).picomp
##                    0.261787098                    0.006201699
##    (Intercept):hour.cen.picomp       hour.cen:hour.cen.picomp
##                    0.006201699                    0.004629381
##                          units
##                    0.104161933
```

```r
HPDinterval(speed.day1.idA$VCV)
```

```
##                                       lower       upper
```

```
## (Intercept):(Intercept).picomp  0.156436125 0.454494176
## hour.cen:(Intercept).picomp    -0.009232813 0.020705918
## (Intercept):hour.cen.picomp    -0.009232813 0.020705918
## hour.cen:hour.cen.picomp        0.002323781 0.007951043
## units                           0.092985074 0.124957537
## attr(,"Probability")
## [1] 0.95
```

```
# only ID repeatability
rpt.spd1.40 <- speed.day1.idA$VCV[, "(Intercept):(Intercept).picomp"]/(speed.day1.idA$VCV[,
    "(Intercept):(Intercept).picomp"] + speed.day1.idA$VCV[, "hour.cen:hour.cen.picomp"] +
    speed.day1.idA$VCV[, "units"])


# to get marginal R2 to explain fixed effects variance
vmVarF <- numeric(2500)

for (i in 1:2500) {
    Var <- var(as.vector(speed.day1.idA$Sol[i, ] %*% t(speed.day1.idA$X)))
    vmVarF[i] <- Var
}


R2m <- vmVarF/(vmVarF + speed.day1.idA$VCV[, 1] + speed.day1.idA$VCV[, 4] +
    speed.day1.idA$VCV[, 5])

posterior.mode(R2m)
```

```
##      var1
## 0.1050187
```

```
HPDinterval(R2m)
```

```
##           lower    upper
## var1 0.04170539 0.281275
## attr(,"Probability")
## [1] 0.95
```

```
R2c <- (vmVarF + speed.day1.idA$VCV[, 1])/(vmVarF + speed.day1.idA$VCV[, 1] +
    speed.day1.idA$VCV[, 4] + speed.day1.idA$VCV[, 5])

posterior.mode(R2c)
```

```
##      var1
## 0.7513314
```

```
HPDinterval(R2c)
```

```
##          lower     upper
## var1 0.6626275 0.8438251
## attr(,"Probability")
## [1] 0.95
```

> **other behaviors** We also used the x, y coordinates from our tracking software to estimate a number of other behavioral variables. First, let's check our model assumptions quickly

```
# Distmoved.iqr - some skews from normality on the tails, but homorgeneity
# of variance looks really good
lm.test <- lme(distmoved.iqr ~ hour.cen + TL.cen, random = ~1 | picomp, data = day1.com)
par(mfrow = c(2, 2))
plot(lm.test)
```

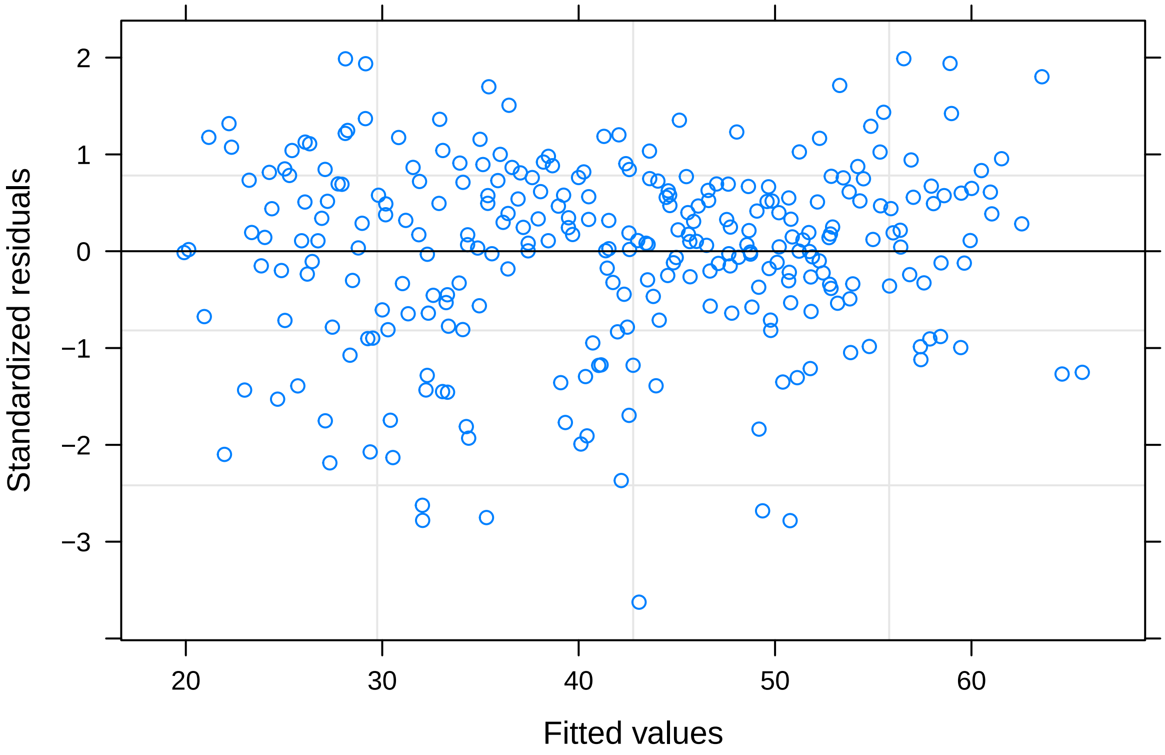


```
hist(resid(lm.test))
qqnorm(resid(lm.test))
qqline(resid(lm.test))


# Prop moving - decreasing variance, but no standard transformation seems
# helpful
lm.test <- lme(propmoving ~ hour.cen + TL.cen, random = ~1 | picomp, data = day1.com)
par(mfrow = c(2, 2))
```

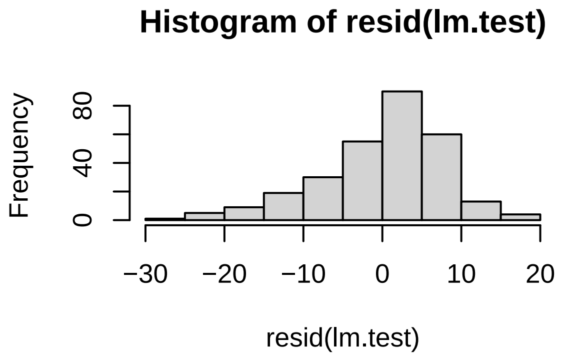
Histogram of resid(lm.test)
Frequency
0
40
80
−30
−20
−10
0
10
20
resid(lm.test)

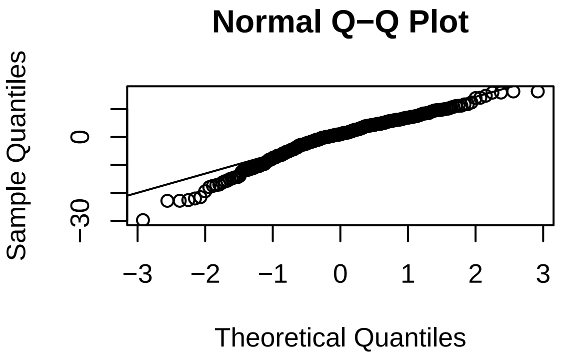
Normal Q−Q Plot
Sample Quantiles
−30
0
−3
−2
−1
0
1
2
3
Theoretical Quantiles

```
plot(lm.test)
```

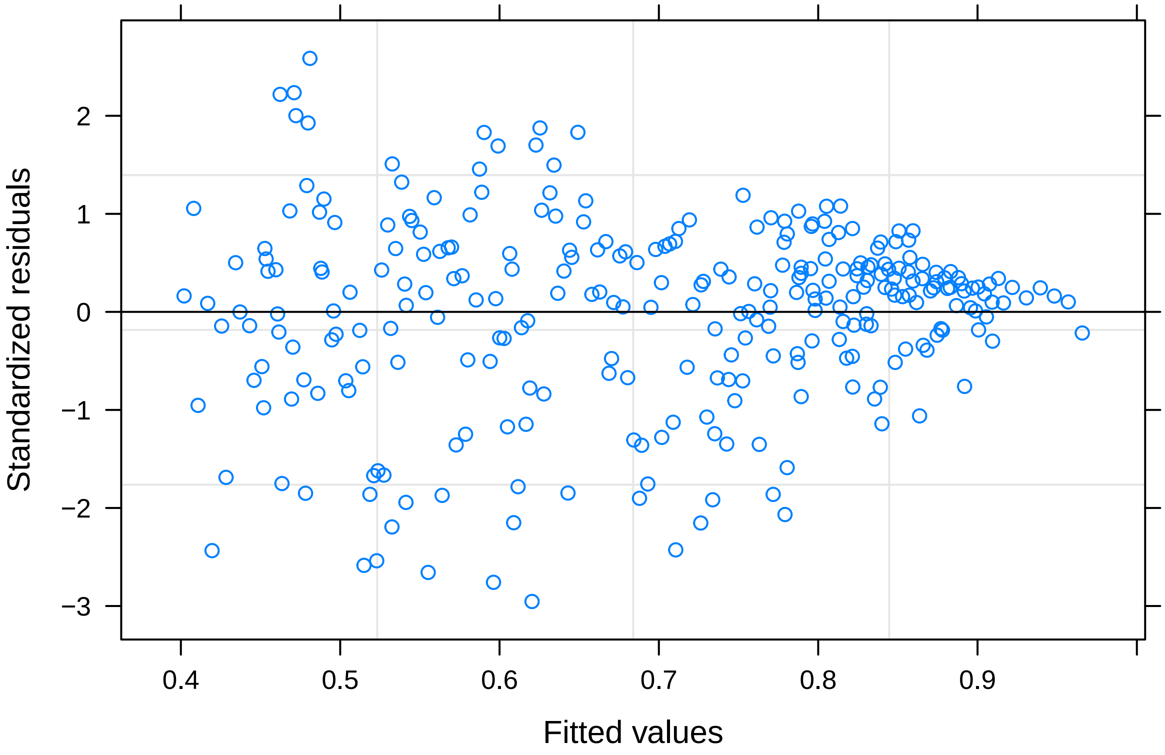


```
hist(resid(lm.test))
qqnorm(resid(lm.test))
qqline(resid(lm.test))


# Median distance from tank border - looks good!
lm.test <- lme(borderdist.med ~ hour.cen + TL.cen, random = ~1 | picomp, data = day1.com)
par(mfrow = c(2, 2))
```

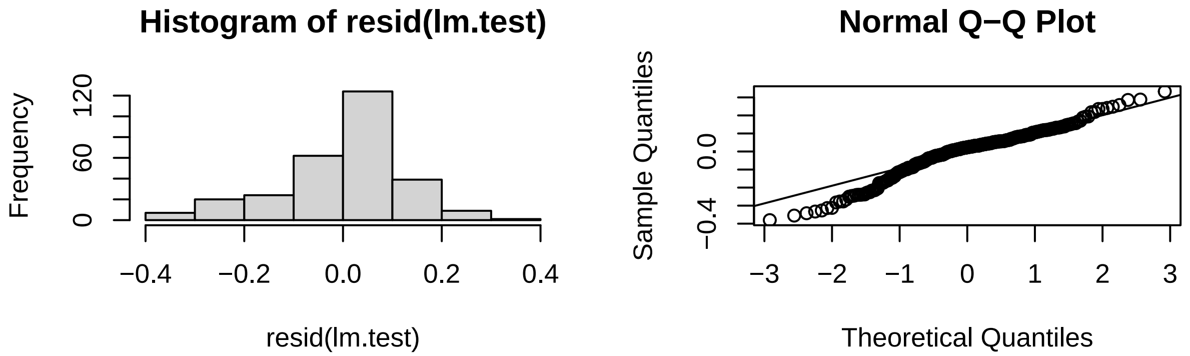
Histogram of resid(lm.test)
Frequency
0
60
120
−0.4
−0.2
0.0
0.2
0.4
resid(lm.test)
Normal Q−Q Plot
Sample Quantiles
−0.4
0.0
−3
−2
−1
0
1
2
3
Theoretical Quantiles

```
plot(lm.test)
```

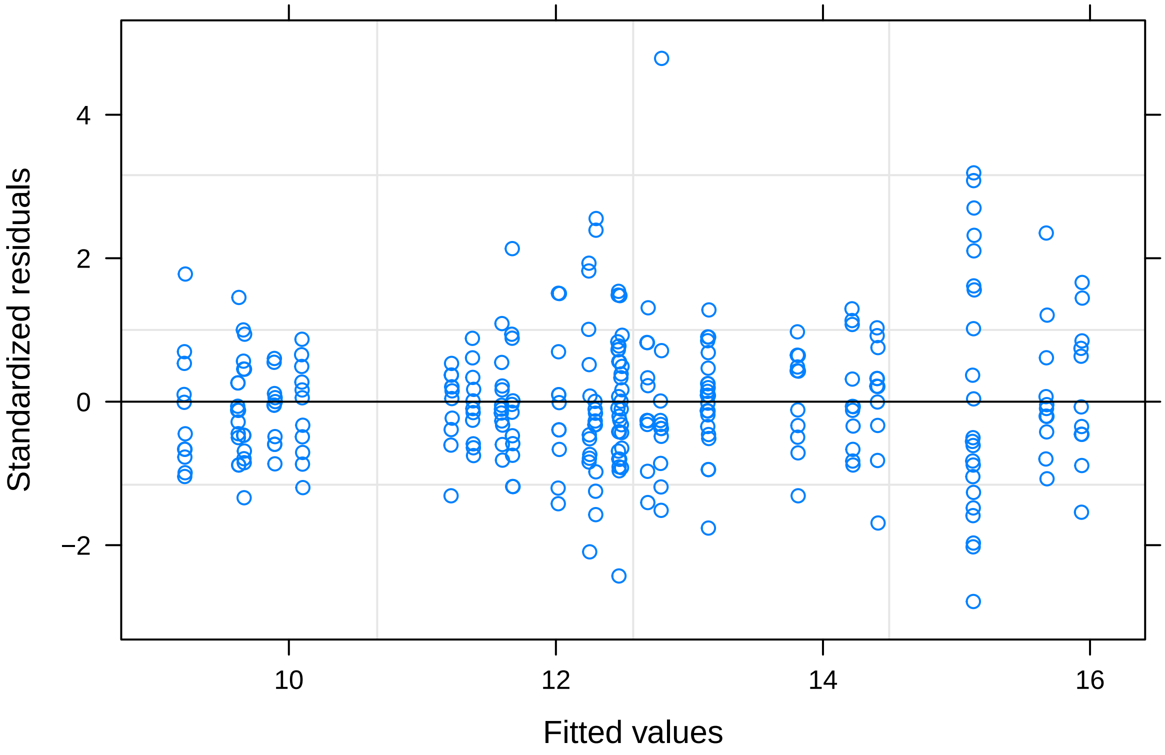


```
hist(resid(lm.test))
qqnorm(resid(lm.test))
qqline(resid(lm.test))
```

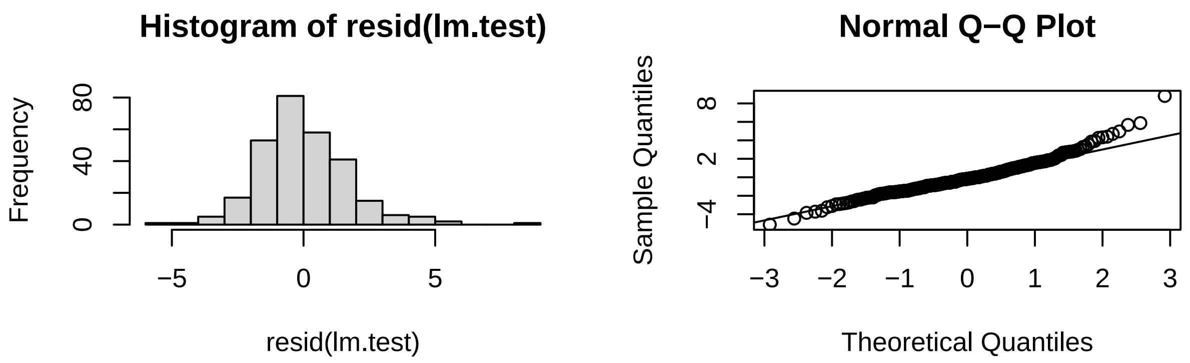

.. 26 individuals Here we test for repeatability on day 1 of life in those behaviors - all are repeatable!

```
# Variation in activity - speed IQR
set.seed(591)
iqr.day1.id <- MCMCglmm(distmoved.iqr ~ hour.cen + TL.cen, random = ~us(1 +
    hour.cen):picomp, prior = prior.id.slope, data = day1.com, family = "gaussian",
    nitt = 510000, burnin = 10000, thin = 200, verbose = F)

summary(iqr.day1.id)
```

```
##
##  Iterations = 10001:509801
##  Thinning interval  = 200
##  Sample size  = 2500
##
##  DIC: 1981.587
##
##  G-structure:  ~us(1 + hour.cen):picomp
##
##                                  post.mean l-95% CI u-95% CI eff.samp
## (Intercept):(Intercept).picomp    119.287  53.3913  210.611     2710
## hour.cen:(Intercept).picomp        -2.576 -11.0206    4.859     2500
## (Intercept):hour.cen.picomp        -2.576 -11.0206    4.859     2500
## hour.cen:hour.cen.picomp            1.849   0.6232    3.303     2500
##
```

```
##  R-structure:  ~units
##
##       post.mean l-95% CI u-95% CI eff.samp
## units     51.06    41.87    59.68     2500
##
##  Location effects: distmoved.iqr ~ hour.cen + TL.cen
##
##             post.mean l-95% CI u-95% CI eff.samp  pMCMC
## (Intercept)   35.6589  30.8526  40.6405     2500 <4e-04 ***
## hour.cen       1.0263   0.4355   1.6143     2744 0.0016 **
## TL.cen        27.2160 -18.4053  67.6026     2500 0.2032
## ---
## Signif. codes:  0 '***' 0.001 '**' 0.01 '*' 0.05 '.' 0.1 ' ' 1
```

```
posterior.mode(iqr.day1.id$VCV)
```

```
## (Intercept):(Intercept).picomp    hour.cen:(Intercept).picomp
##                      85.000199                      -2.654759
##    (Intercept):hour.cen.picomp       hour.cen:hour.cen.picomp
##                      -2.654759                       1.430073
##                          units
##                      49.684393
```

```
HPDinterval(iqr.day1.id$VCV)
```

```
##                                      lower      upper
## (Intercept):(Intercept).picomp  53.3912553 210.611328
## hour.cen:(Intercept).picomp    -11.0205736   4.858572
## (Intercept):hour.cen.picomp    -11.0205736   4.858572
## hour.cen:hour.cen.picomp         0.6231612   3.303360
## units                           41.8665917  59.676047
## attr(,"Probability")
## [1] 0.95
```

```
rpt.iqr1 <- iqr.day1.id$VCV[, "(Intercept):(Intercept).picomp"]/(iqr.day1.id$VCV[,
    "(Intercept):(Intercept).picomp"] + iqr.day1.id$VCV[, "hour.cen:hour.cen.picomp"] +
    iqr.day1.id$VCV[, "units"])
posterior.mode(rpt.iqr1)
```

```
##      var1
## 0.6766486
```

```
HPDinterval(rpt.iqr1)
```

```
##         lower     upper
## var1 0.520051 0.8119163
## attr(,"Probability")
## [1] 0.95
```

```r
# Activity - Proportion of time spent moving
set.seed(8547)
prop.day1.id <- MCMCglmm(propmoving ~ hour.cen + TL.cen, random = ~us(1 + hour.cen):picomp,
    prior = prior.id.slope, data = day1.com, family = "gaussian", nitt = 510000,
    burnin = 10000, thin = 200, verbose = F)

summary(prop.day1.id)
```

```
##
##  Iterations = 10001:509801
##  Thinning interval  = 200
##  Sample size  = 2500
##
##  DIC: -423.5535
##
##  G-structure:  ~us(1 + hour.cen):picomp
##
##                                  post.mean   l-95% CI  u-95% CI eff.samp
## (Intercept):(Intercept).picomp  0.0307809  0.0127094 0.0537298     2500
## hour.cen:(Intercept).picomp    -0.0015771 -0.0041944 0.0003421     2500
## (Intercept):hour.cen.picomp    -0.0015771 -0.0041944 0.0003421     2500
## hour.cen:hour.cen.picomp        0.0006085  0.0002524 0.0010643     1930
##
##  R-structure:  ~units
##
##       post.mean l-95% CI u-95% CI eff.samp
## units   0.01133 0.009351  0.01343     2500
##
##  Location effects: propmoving ~ hour.cen + TL.cen
##
##             post.mean  l-95% CI  u-95% CI eff.samp  pMCMC
## (Intercept)  0.638673  0.560370  0.714963     2500 <4e-04 ***
## hour.cen     0.008659 -0.001941  0.018248     2654  0.092 .
## TL.cen       0.348924 -0.343156  1.007917     2500  0.271
## ---
## Signif. codes:  0 '***' 0.001 '**' 0.01 '*' 0.05 '.' 0.1 ' ' 1
```

```r
posterior.mode(prop.day1.id$VCV)
```

```
## (Intercept):(Intercept).picomp    hour.cen:(Intercept).picomp
##                   0.0253877145                  -0.0012824261
##    (Intercept):hour.cen.picomp       hour.cen:hour.cen.picomp
##                  -0.0012824261                   0.0004792935
##                          units
##                   0.0108856321
```

```r
HPDinterval(prop.day1.id$VCV)
```

```
##                                        lower        upper
## (Intercept):(Intercept).picomp  0.0127094390 0.0537298151
## hour.cen:(Intercept).picomp    -0.0041944205 0.0003421329
## (Intercept):hour.cen.picomp    -0.0041944205 0.0003421329
```

```
## hour.cen:hour.cen.picomp        0.0002524125 0.0010643306
## units                           0.0093512811 0.0134333686
## attr(,"Probability")
## [1] 0.95
```

```
rpt.prop1 <- prop.day1.id$VCV[, "(Intercept):(Intercept).picomp"]/(prop.day1.id$VCV[,
    "(Intercept):(Intercept).picomp"] + prop.day1.id$VCV[, "hour.cen:hour.cen.picomp"] +
    prop.day1.id$VCV[, "units"])

posterior.mode(rpt.prop1)
```

```
##      var1
## 0.7064532
```

```
HPDinterval(rpt.prop1)
```

```
##          lower     upper
## var1 0.5566278 0.8361055
## attr(,"Probability")
## [1] 0.95
```

```
# median distance to tank border
set.seed(693)
border.day1.id <- MCMCglmm(borderdist.med ~ hour.cen + TL.cen, random = ~us(1 +
    hour.cen):picomp, prior = prior.id.slope, data = day1.com, family = "gaussian",
    nitt = 510000, burnin = 10000, thin = 200, verbose = F)

summary(border.day1.id)
```

```
##
##  Iterations = 10001:509801
##  Thinning interval  = 200
##  Sample size  = 2500
##
##  DIC: 1128.802
##
##  G-structure:  ~us(1 + hour.cen):picomp
##
##                                  post.mean l-95% CI u-95% CI eff.samp
## (Intercept):(Intercept).picomp    9.30895  4.32976  15.4862     2500
## hour.cen:(Intercept).picomp      -0.68755 -1.29761  -0.1958     2500
## (Intercept):hour.cen.picomp      -0.68755 -1.29761  -0.1958     2500
## hour.cen:hour.cen.picomp          0.08998  0.03332   0.1648     2500
##
##  R-structure:  ~units
##
##       post.mean l-95% CI u-95% CI eff.samp
## units      2.59    2.146    3.075     2500
##
##  Location effects: borderdist.med ~ hour.cen + TL.cen
##
##              post.mean   l-95% CI   u-95% CI eff.samp  pMCMC
```

```
## (Intercept) 12.5000241 11.1668531 13.7961153     2406 <4e-04 ***
## hour.cen    -0.0006894 -0.1255196  0.1408577     2500  0.994
## TL.cen      -0.4324584 -7.8740827  9.1485220     2500  0.905
## ---
## Signif. codes:  0 '***' 0.001 '**' 0.01 '*' 0.05 '.' 0.1 ' ' 1
```

```
posterior.mode(border.day1.id$VCV)
```

```
## (Intercept):(Intercept).picomp    hour.cen:(Intercept).picomp
##                     7.51796696                    -0.51163783
##    (Intercept):hour.cen.picomp       hour.cen:hour.cen.picomp
##                    -0.51163783                     0.07945216
##                          units
##                     2.46201241
```

```
HPDinterval(border.day1.id$VCV)
```

```
##                                      lower      upper
## (Intercept):(Intercept).picomp  4.32976071 15.4862035
## hour.cen:(Intercept).picomp    -1.29760655 -0.1958071
## (Intercept):hour.cen.picomp    -1.29760655 -0.1958071
## hour.cen:hour.cen.picomp        0.03331914  0.1647879
## units                           2.14612499  3.0745324
## attr(,"Probability")
## [1] 0.95
```

```
rpt.bord1 <- border.day1.id$VCV[, "(Intercept):(Intercept).picomp"]/(border.day1.id$VCV[,
    "(Intercept):(Intercept).picomp"] + border.day1.id$VCV[, "hour.cen:hour.cen.picomp"] +
    border.day1.id$VCV[, "units"])
posterior.mode(rpt.bord1)
```

```
##      var1
## 0.8012109
```

```
HPDinterval(rpt.bord1)
```

```
##          lower     upper
## var1 0.6420183 0.8659903
## attr(,"Probability")
## [1] 0.95
```

.. 40 individuals Our analyses are generally restricted to those animals for which we have complete data on through week 10, but here we'll check and make sure that we find similar patterns of behavioral variation on Day 1 using all individuals that were inititally placed in the tracking tanks

ANSWER - yes, all results hold regardless of which set of individuals we look at

```
day1 <- hourly %>%
    filter(obs == 1) %>%
    mutate(hour.cen = hour - 1, TL.cen = TL - (sum(unique(TL))/40))
```

median speed
```r
# median speed
speed.day1.40 <- MCMCglmm(speed.med ~ hour.cen + TL.cen, random = ~us(1 + hour.cen):picomp,
    prior = prior.id.slope, data = day1, family = "gaussian", nitt = 510000,
    burnin = 10000, thin = 200, verbose = F)



summary(speed.day1.40)
```

```
##
##  Iterations = 10001:509801
##  Thinning interval  = 200
##  Sample size  = 2500
##
##  DIC: 340.2276
##
##  G-structure:  ~us(1 + hour.cen):picomp
##
##                                 post.mean  l-95% CI u-95% CI eff.samp
## (Intercept):(Intercept).picomp  0.289186  0.155941 0.452136     2326
## hour.cen:(Intercept).picomp     0.005350 -0.009024 0.020935     2500
## (Intercept):hour.cen.picomp     0.005350 -0.009024 0.020935     2500
## hour.cen:hour.cen.picomp        0.004995  0.002376 0.008047     1934
##
##  R-structure:  ~units
##
##       post.mean l-95% CI u-95% CI eff.samp
## units    0.1087  0.09268    0.125     2500
##
##  Location effects: speed.med ~ hour.cen + TL.cen
##
##             post.mean l-95% CI u-95% CI eff.samp  pMCMC
## (Intercept)   0.90159  0.70804  1.09894     2797 <4e-04 ***
## hour.cen      0.05799  0.03367  0.08216     2500 <4e-04 ***
## TL.cen        1.77364  0.02572  3.42089     2500 0.0432 *
## ---
## Signif. codes:  0 '***' 0.001 '**' 0.01 '*' 0.05 '.' 0.1 ' ' 1
```

```r
plot(speed.day1.40$VCV)
```

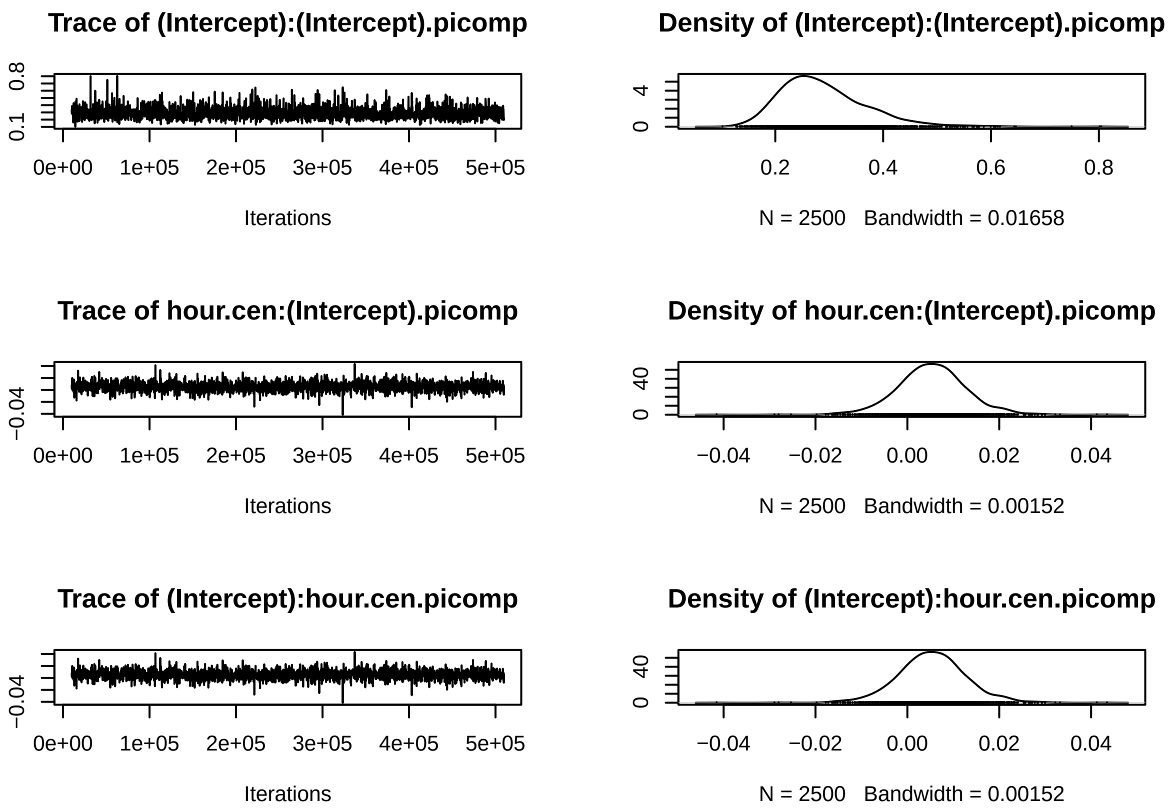
Trace of (Intercept):(Intercept).picomp
0.1
0.8
0e+00
1e+05
2e+05
3e+05
4e+05
5e+05
Iterations
Density of (Intercept):(Intercept).picomp
0
4
0.2
0.4
0.6
0.8
N = 2500 Bandwidth = 0.01658
Trace of hour.cen:(Intercept).picomp
−0.04
0e+00
1e+05
2e+05
3e+05
4e+05
5e+05
Iterations
Density of hour.cen:(Intercept).picomp
0
40
−0.04
−0.02
0.00
0.02
0.04
N = 2500 Bandwidth = 0.00152
Trace of (Intercept):hour.cen.picomp
−0.04
0e+00
1e+05
2e+05
3e+05
4e+05
5e+05
Iterations
Density of (Intercept):hour.cen.picomp
0
40
−0.04
−0.02
0.00
0.02
0.04
N = 2500 Bandwidth = 0.00152

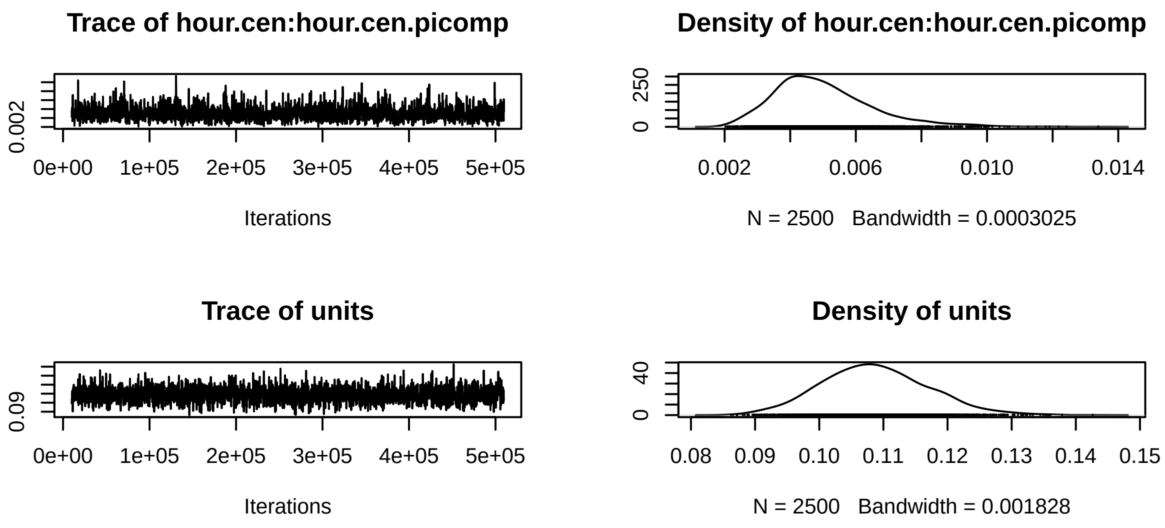


```
posterior.mode(speed.day1.40$VCV)
```

```
## (Intercept):(Intercept).picomp    hour.cen:(Intercept).picomp
##                     0.241138406                    0.003239799
##    (Intercept):hour.cen.picomp       hour.cen:hour.cen.picomp
##                     0.003239799                    0.004022300
##                           units
##                     0.107981939
```

```
HPDinterval(speed.day1.40$VCV)
```

```
##                                        lower      upper
## (Intercept):(Intercept).picomp   0.155940540 0.45213613
## hour.cen:(Intercept).picomp     -0.009024380 0.02093502
## (Intercept):hour.cen.picomp     -0.009024380 0.02093502
## hour.cen:hour.cen.picomp         0.002376438 0.00804711
## units                            0.092676608 0.12503040
## attr(,"Probability")
## [1] 0.95
```

```
# only ID repeatability
rpt.spd40 <- speed.day1.40$VCV[, "(Intercept):(Intercept).picomp"]/(speed.day1.40$VCV[,
    "(Intercept):(Intercept).picomp"] + speed.day1.40$VCV[, "hour.cen:hour.cen.picomp"] +
    speed.day1.40$VCV[, "units"])
posterior.mode(rpt.spd40)
```

```
##      var1
## 0.7054354
```

```
HPDinterval(rpt.spd40)
```

```
##          lower     upper
## var1 0.5948525 0.8142106
## attr(,"Probability")
## [1] 0.95
```

```
# speed IQR
set.seed(900)
iqr.day1.id <- MCMCglmm(distmoved.iqr ~ hour.cen + TL.cen, random = ~us(1 +
    hour.cen):picomp, prior = prior.id.slope, data = day1, family = "gaussian",
    nitt = 510000, burnin = 10000, thin = 200, verbose = F)

summary(iqr.day1.id)
```

```
##
##  Iterations = 10001:509801
##  Thinning interval  = 200
##  Sample size  = 2500
##
##  DIC: 3038.199
##
##  G-structure:  ~us(1 + hour.cen):picomp
##
##                                  post.mean l-95% CI u-95% CI eff.samp
## (Intercept):(Intercept).picomp   135.203   72.090  205.018     2500
## hour.cen:(Intercept).picomp       -4.831  -12.006    1.481     2500
## (Intercept):hour.cen.picomp       -4.831  -12.006    1.481     2500
## hour.cen:hour.cen.picomp           2.087    1.018    3.408     2500
##
##  R-structure:  ~units
##
##       post.mean l-95% CI u-95% CI eff.samp
## units     49.68    43.03    57.56     2500
##
##  Location effects: distmoved.iqr ~ hour.cen + TL.cen
##
##             post.mean l-95% CI u-95% CI eff.samp  pMCMC
## (Intercept)   36.2492  32.1419  40.4002     2500 <4e-04 ***
## hour.cen       0.8479   0.3342   1.3125     2500 0.0008 ***
## TL.cen        27.8660  -6.3393  61.4391     2500 0.0984 .
## ---
## Signif. codes:  0 '***' 0.001 '**' 0.01 '*' 0.05 '.' 0.1 ' ' 1
```

```
posterior.mode(iqr.day1.id$VCV)
```

```
## (Intercept):(Intercept).picomp    hour.cen:(Intercept).picomp
##                     136.731522                      -3.843599
##    (Intercept):hour.cen.picomp       hour.cen:hour.cen.picomp
```

```
##                       -3.843599                       1.597489
##                           units
##                       49.132089
```

```
HPDinterval(iqr.day1.id$VCV)
```

```
##                                        lower      upper
## (Intercept):(Intercept).picomp  72.089987 205.017990
## hour.cen:(Intercept).picomp    -12.006274   1.480872
## (Intercept):hour.cen.picomp    -12.006274   1.480872
## hour.cen:hour.cen.picomp         1.017605   3.408304
## units                           43.026451  57.561900
## attr(,"Probability")
## [1] 0.95
```

```
rpt.iqr1 <- iqr.day1.id$VCV[, "(Intercept):(Intercept).picomp"]/(iqr.day1.id$VCV[,
    "(Intercept):(Intercept).picomp"] + iqr.day1.id$VCV[, "hour.cen:hour.cen.picomp"] +
    iqr.day1.id$VCV[, "units"])
posterior.mode(rpt.iqr1)
```

```
##      var1
## 0.6950842
```

```
HPDinterval(rpt.iqr1)
```

```
##         lower     upper
## var1 0.603514 0.8102665
## attr(,"Probability")
## [1] 0.95
```

```
# Proportion of time spent moving
set.seed(810)
prop.day1.id <- MCMCglmm(propmoving ~ hour.cen + TL.cen, random = ~us(1 + hour.cen):picomp,
    prior = prior.id.slope, data = day1, family = "gaussian", nitt = 510000,
    burnin = 10000, thin = 200, verbose = F)

summary(prop.day1.id)
```

```
##
##  Iterations = 10001:509801
##  Thinning interval  = 200
##  Sample size  = 2500
##
##  DIC: -672.9462
##
##  G-structure:  ~us(1 + hour.cen):picomp
##
##                                  post.mean   l-95% CI  u-95% CI eff.samp
## (Intercept):(Intercept).picomp  0.0277472  0.0144136 0.0434898     2339
## hour.cen:(Intercept).picomp    -0.0007160 -0.0022626 0.0006277     2500
## (Intercept):hour.cen.picomp    -0.0007160 -0.0022626 0.0006277     2500
```

```
## hour.cen:hour.cen.picomp        0.0004378  0.0002266 0.0007406     2500
##
##  R-structure:  ~units
##
##       post.mean l-95% CI u-95% CI eff.samp
## units   0.01082 0.009218  0.01255     2500
##
##  Location effects: propmoving ~ hour.cen + TL.cen
##
##             post.mean  l-95% CI  u-95% CI eff.samp  pMCMC
## (Intercept)  0.630142  0.566389  0.684575     2500 <4e-04 ***
## hour.cen     0.008625  0.002133  0.016439     2835 0.0184 *
## TL.cen       0.459872 -0.048249  0.965476     2593 0.0784 .
## ---
## Signif. codes:  0 '***' 0.001 '**' 0.01 '*' 0.05 '.' 0.1 ' ' 1
```

```
posterior.mode(prop.day1.id$VCV)
```

```
## (Intercept):(Intercept).picomp    hour.cen:(Intercept).picomp
##                    0.026981070                   -0.000866961
##    (Intercept):hour.cen.picomp       hour.cen:hour.cen.picomp
##                   -0.000866961                    0.000406013
##                          units
##                    0.010847988
```

```
HPDinterval(prop.day1.id$VCV)
```

```
##                                        lower        upper
## (Intercept):(Intercept).picomp  0.0144135606 0.0434897911
## hour.cen:(Intercept).picomp    -0.0022625975 0.0006276740
## (Intercept):hour.cen.picomp    -0.0022625975 0.0006276740
## hour.cen:hour.cen.picomp        0.0002265631 0.0007406139
## units                           0.0092175140 0.0125470664
## attr(,"Probability")
## [1] 0.95
```

```
rpt.prop1 <- prop.day1.id$VCV[, "(Intercept):(Intercept).picomp"]/(prop.day1.id$VCV[,
    "(Intercept):(Intercept).picomp"] + prop.day1.id$VCV[, "hour.cen:hour.cen.picomp"] +
    prop.day1.id$VCV[, "units"])
posterior.mode(rpt.prop1)
```

```
##      var1
## 0.6934451
```

```
HPDinterval(rpt.prop1)
```

```
##          lower     upper
## var1 0.5860356 0.8115755
## attr(,"Probability")
## [1] 0.95
```

```r
# median distance to tank border
set.seed(320)
border.day1.id <- MCMCglmm(borderdist.med ~ hour.cen + TL.cen, random = ~us(1 +
    hour.cen):picomp, prior = prior.id.slope, data = day1, family = "gaussian",
    nitt = 510000, burnin = 10000, thin = 200, verbose = F)

summary(border.day1.id)
```

```
##
##  Iterations = 10001:509801
##  Thinning interval  = 200
##  Sample size  = 2500
##
##  DIC: 1798.642
##
##  G-structure:  ~us(1 + hour.cen):picomp
##
##                               post.mean l-95% CI u-95% CI eff.samp
## (Intercept):(Intercept).picomp   8.13336  4.50890 12.66174     2500
## hour.cen:(Intercept).picomp     -0.40597 -0.76921 -0.07922     2500
## (Intercept):hour.cen.picomp     -0.40597 -0.76921 -0.07922     2500
## hour.cen:hour.cen.picomp         0.06322  0.02409  0.10654     2500
##
##  R-structure:  ~units
##
##       post.mean l-95% CI u-95% CI eff.samp
## units     3.008    2.576    3.446     2500
##
##  Location effects: borderdist.med ~ hour.cen + TL.cen
##
##             post.mean l-95% CI u-95% CI eff.samp  pMCMC
## (Intercept)   12.3287  11.2842  13.3001     2500 <4e-04 ***
## hour.cen       0.0202  -0.0712   0.1171     2500  0.656
## TL.cen         3.1624  -4.1112   9.7378     3083  0.359
## ---
## Signif. codes:  0 '***' 0.001 '**' 0.01 '*' 0.05 '.' 0.1 ' ' 1
```

```r
posterior.mode(border.day1.id$VCV)
```

```
## (Intercept):(Intercept).picomp    hour.cen:(Intercept).picomp
##                     7.33520497                    -0.36949267
##    (Intercept):hour.cen.picomp       hour.cen:hour.cen.picomp
##                    -0.36949267                     0.05596061
##                          units
##                     3.01922216
```

```r
HPDinterval(border.day1.id$VCV)
```

```
##                                      lower       upper
## (Intercept):(Intercept).picomp  4.50890391 12.66173697
## hour.cen:(Intercept).picomp    -0.76920512 -0.07921901
## (Intercept):hour.cen.picomp    -0.76920512 -0.07921901
```

```
## hour.cen:hour.cen.picomp        0.02409226  0.10653793
## units                           2.57552828  3.44616700
## attr(,"Probability")
## [1] 0.95
```

```
rpt.bord1 <- border.day1.id$VCV[, "(Intercept):(Intercept).picomp"]/(border.day1.id$VCV[,
    "(Intercept):(Intercept).picomp"] + border.day1.id$VCV[, "hour.cen:hour.cen.picomp"] +
    border.day1.id$VCV[, "units"])
posterior.mode(rpt.bord1)
```

```
##     var1
## 0.717202
```

```
HPDinterval(rpt.bord1)
```

```
##          lower     upper
## var1 0.6116683 0.8190378
## attr(,"Probability")
## [1] 0.95
```

Days 2 - 7 : We found evidence that individuals significantly differed in their behavior on Day 1 of their lives. Here we then test for repeatability of behavior on each subsequent day of life (days 2-7) to see how robust this finding is.

Bottom line: Individuals show significant repeatability in behavior in each day in the first week of their lives. This is a robust result!

```
day2 <- hourly.com %>%
    filter(obs == 2) %>%
    mutate(hour.cen = hour - 1, TL.cen = TL - (sum(unique(TL))/26))

# Null model -----------
set.seed(58)
speed.day2.0 <- MCMCglmm(speed.med ~ hour.cen + TL.cen, data = day2, family = "gaussian",
    prior = prior.null, nitt = 510000, burnin = 10000, thin = 200, verbose = F)


# Intercepts ID ----------------------
set.seed(3432)
speed.day2.1 <- MCMCglmm(speed.med ~ hour.cen + TL.cen, random = ~picomp, data = day2,
    family = "gaussian", prior = prior.id, nitt = 510000, burnin = 10000, thin = 200,
    verbose = F)

# only including intercepts for mother ------
set.seed(42021)
speed.day2.2 <- MCMCglmm(speed.med ~ hour.cen + TL.cen, random = ~mother, data = day2,
    family = "gaussian", prior = prior.id, nitt = 510000, burnin = 10000, thin = 200,
    verbose = F)
```

```
# Intercepts ID and Mom-----------
set.seed(258)
speed.day2.3 <- MCMCglmm(speed.med ~ hour.cen + TL.cen, random = ~picomp + mother,
    data = day2, family = "gaussian", prior = prior.id.mom, nitt = 510000, burnin = 10000,
    thin = 200, verbose = F)


# Intercepts and Slopes ID -----------------------
set.seed(472)
speed.day2.4 <- MCMCglmm(speed.med ~ hour.cen + TL.cen, random = ~us(1 + hour.cen):picomp,
    data = day2, family = "gaussian", prior = prior.id.slope, nitt = 510000,
    burnin = 10000, thin = 200, verbose = F)

# Intercepts Mom, Slopes Mom ----------------
set.seed(318)
speed.day2.5 <- MCMCglmm(speed.med ~ hour.cen + TL.cen, random = ~us(1 + hour.cen):mother,
    data = day2, family = "gaussian", prior = prior.id.slope, nitt = 510000,
    burnin = 10000, thin = 200, verbose = F)


# Intercepts ID and Mom, Slopes ID ----------------
set.seed(918)
speed.day2.6 <- MCMCglmm(speed.med ~ hour.cen + TL.cen, random = ~us(1 + hour.cen):picomp +
    mother, data = day2, family = "gaussian", prior = prior.id.slope.mom, nitt = 510000,
    burnin = 10000, thin = 200, verbose = F)


# Intercepts ID & Mom, Slopes ID & Mom -----------------
set.seed(78)
speed.day2.7 <- MCMCglmm(speed.med ~ hour.cen + TL.cen, random = ~us(1 + hour.cen):picomp +
    us(1 + hour.cen):mother, data = day2, family = "gaussian", prior = prior.id.slope.mom.slope,
    nitt = 510000, burnin = 10000, thin = 200, verbose = F)



DIC(speed.day2.0, speed.day2.1, speed.day2.2, speed.day2.3, speed.day2.4, speed.day2.5,
    speed.day2.6, speed.day2.7)
```

> **Day 2:**

```
##              df      DIC
## speed.day2.0  4 671.3710
## speed.day2.1  5 363.2638
## speed.day2.2  5 549.0355
## speed.day2.3  6 363.2159
## speed.day2.4  6 327.8335
## speed.day2.5  6 551.0234
## speed.day2.6  7 328.2104
## speed.day2.7  8 329.8870
```

.. report:

```r
set.seed(472)
speed.day2.4 <- MCMCglmm(speed.med ~ hour.cen + TL.cen, random = ~us(1 + hour.cen):picomp,
    data = day2, family = "gaussian", prior = prior.id.slope, nitt = 510000,
    burnin = 10000, thin = 200, verbose = F)

summary(speed.day2.4)
```

```
##
##  Iterations = 10001:509801
##  Thinning interval  = 200
##  Sample size  = 2500
##
##  DIC: 327.8335
##
##  G-structure:  ~us(1 + hour.cen):picomp
##
##                                  post.mean   l-95% CI u-95% CI eff.samp
## (Intercept):(Intercept).picomp  0.434936  0.1772630 0.760316     2500
## hour.cen:(Intercept).picomp     0.005394 -0.0185006 0.028133     2664
## (Intercept):hour.cen.picomp     0.005394 -0.0185006 0.028133     2664
## hour.cen:hour.cen.picomp        0.004007  0.0007252 0.007627     2469
##
##  R-structure:  ~units
##
##       post.mean l-95% CI u-95% CI eff.samp
## units    0.1664   0.1378   0.1993     2500
##
##  Location effects: speed.med ~ hour.cen + TL.cen
##
##             post.mean  l-95% CI  u-95% CI eff.samp  pMCMC
## (Intercept)  0.593302  0.212778  0.939647     2500 0.0024 **
## hour.cen     0.037098  0.009584  0.068748     2212 0.0120 *
## TL.cen       1.920180 -0.890916  4.562977     2136 0.1656
## ---
## Signif. codes:  0 '***' 0.001 '**' 0.01 '*' 0.05 '.' 0.1 ' ' 1
```

```r
posterior.mode(speed.day2.4$Sol)
```

```
## (Intercept)    hour.cen      TL.cen
##  0.60283681  0.03217546  2.06579140
```

```r
HPDinterval(speed.day2.4$Sol)
```

```
##                   lower      upper
## (Intercept)  0.212778228 0.93964658
## hour.cen     0.009583837 0.06874807
## TL.cen      -0.890915792 4.56297651
## attr(,"Probability")
## [1] 0.95
```

```r
posterior.mode(speed.day2.4$VCV)
```

```
## (Intercept):(Intercept).picomp    hour.cen:(Intercept).picomp
##                     0.342849443                    0.002494569
##    (Intercept):hour.cen.picomp       hour.cen:hour.cen.picomp
##                     0.002494569                    0.002883872
##                           units
##                     0.161991600
```

```r
HPDinterval(speed.day2.4$VCV)
```

```
##                                         lower       upper
## (Intercept):(Intercept).picomp  0.1772629775 0.760316250
## hour.cen:(Intercept).picomp    -0.0185005606 0.028133453
## (Intercept):hour.cen.picomp    -0.0185005606 0.028133453
## hour.cen:hour.cen.picomp        0.0007251829 0.007626783
## units                           0.1378065063 0.199268355
## attr(,"Probability")
## [1] 0.95
```

```r
# only ID repeatability
rpt.spd1 <- speed.day2.4$VCV[, "(Intercept):(Intercept).picomp"]/(speed.day2.4$VCV[,
    "(Intercept):(Intercept).picomp"] + speed.day2.4$VCV[, "hour.cen:hour.cen.picomp"] +
    speed.day2.4$VCV[, "units"])
posterior.mode(rpt.spd1)
```

```
##      var1
## 0.6903639
```

```r
HPDinterval(rpt.spd1)
```

```
##          lower     upper
## var1 0.5595239 0.8486966
## attr(,"Probability")
## [1] 0.95
```

```r
# to get marginal R2 to explain fixed effects variance
vmVarF <- numeric(2500)

for (i in 1:2500) {
    Var <- var(as.vector(speed.day2.4$Sol[i, ] %*% t(speed.day2.4$X)))
    vmVarF[i] <- Var
}


R2m <- vmVarF/(vmVarF + speed.day2.4$VCV[, 1] + speed.day2.4$VCV[, 4] + speed.day2.4$VCV[,
    5])

posterior.mode(R2m)
```

```
##      var1
## 0.0420491
```

```
HPDinterval(R2m)
```

```
##             lower     upper
## var1 0.001642288 0.2518172
## attr(,"Probability")
## [1] 0.95
```

```
R2c <- (vmVarF + speed.day2.4$VCV[, 1])/(vmVarF + speed.day2.4$VCV[, 1] + speed.day2.4$VCV[,
    4] + speed.day2.4$VCV[, 5])

posterior.mode(R2c)
```

```
##      var1
## 0.7643663
```

```
HPDinterval(R2c)
```

```
##           lower    upper
## var1 0.6017514 0.862172
## attr(,"Probability")
## [1] 0.95
```

```
day3 <- hourly.com %>%
    filter(obs == 3) %>%
    mutate(hour.cen = hour - 1, TL.cen = TL - (sum(unique(TL))/26))

# Null model -----------
set.seed(58)
speed.day3.0 <- MCMCglmm(speed.med ~ hour.cen + TL.cen, data = day3, family = "gaussian",
    prior = prior.null, nitt = 510000, burnin = 10000, thin = 200, verbose = F)


# Intercepts ID ----------------------
set.seed(3432)
speed.day3.1 <- MCMCglmm(speed.med ~ hour.cen + TL.cen, random = ~picomp, data = day3,
    family = "gaussian", prior = prior.id, nitt = 510000, burnin = 10000, thin = 200,
    verbose = F)

# only including intercepts for mother ------
set.seed(42021)
speed.day3.2 <- MCMCglmm(speed.med ~ hour.cen + TL.cen, random = ~mother, data = day3,
    family = "gaussian", prior = prior.id, nitt = 510000, burnin = 10000, thin = 200,
    verbose = F)


# Intercepts ID and Mom-----------
```

```r
set.seed(258)
speed.day3.3 <- MCMCglmm(speed.med ~ hour.cen + TL.cen, random = ~picomp + mother,
    data = day3, family = "gaussian", prior = prior.id.mom, nitt = 510000, burnin = 10000,
    thin = 200, verbose = F)


# Intercepts and Slopes ID -----------------------
set.seed(472)
speed.day3.4 <- MCMCglmm(speed.med ~ hour.cen + TL.cen, random = ~us(1 + hour.cen):picomp,
    data = day3, family = "gaussian", prior = prior.id.slope, nitt = 510000,
    burnin = 10000, thin = 200, verbose = F)

# Intercepts Mom, Slopes Mom ----------------
set.seed(318)
speed.day3.5 <- MCMCglmm(speed.med ~ hour.cen + TL.cen, random = ~us(1 + hour.cen):mother,
    data = day3, family = "gaussian", prior = prior.id.slope, nitt = 510000,
    burnin = 10000, thin = 200, verbose = F)


# Intercepts ID and Mom, Slopes ID ----------------
set.seed(918)
speed.day3.6 <- MCMCglmm(speed.med ~ hour.cen + TL.cen, random = ~us(1 + hour.cen):picomp +
    mother, data = day3, family = "gaussian", prior = prior.id.slope.mom, nitt = 510000,
    burnin = 10000, thin = 200, verbose = F)


# Intercepts ID & Mom, Slopes ID & Mom -----------------
set.seed(78)
speed.day3.7 <- MCMCglmm(speed.med ~ hour.cen + TL.cen, random = ~us(1 + hour.cen):picomp +
    us(1 + hour.cen):mother, data = day3, family = "gaussian", prior = prior.id.slope.mom.slope,
    nitt = 510000, burnin = 10000, thin = 200, verbose = F)



DIC(speed.day3.0, speed.day3.1, speed.day3.2, speed.day3.3, speed.day3.4, speed.day3.5,
    speed.day3.6, speed.day3.7)
```

> **Day 3:**

```
##              df      DIC
## speed.day3.0  4 678.2679
## speed.day3.1  5 468.1906
## speed.day3.2  5 559.2013
## speed.day3.3  6 467.7955
## speed.day3.4  6 420.0719
## speed.day3.5  6 557.0478
## speed.day3.6  7 419.8940
## speed.day3.7  8 421.2377
```

.. report

```r
set.seed(472)
speed.day3.4 <- MCMCglmm(speed.med ~ hour.cen + TL.cen, random = ~us(1 + hour.cen):picomp,
    data = day3, family = "gaussian", prior = prior.id.slope, nitt = 510000,
    burnin = 10000, thin = 200, verbose = F)

summary(speed.day3.4)
```

```
##
##  Iterations = 10001:509801
##  Thinning interval  = 200
##  Sample size  = 2500
##
##  DIC: 420.0719
##
##  G-structure:  ~us(1 + hour.cen):picomp
##
##                                  post.mean l-95% CI  u-95% CI eff.samp
## (Intercept):(Intercept).picomp   0.567597  0.22651 0.9788347     2500
## hour.cen:(Intercept).picomp    -0.028513 -0.06598 0.0008464     2500
## (Intercept):hour.cen.picomp    -0.028513 -0.06598 0.0008464     2500
## hour.cen:hour.cen.picomp        0.006489  0.00206 0.0117151     2500
##
##  R-structure:  ~units
##
##       post.mean l-95% CI u-95% CI eff.samp
## units    0.2178   0.1819   0.2591     2461
##
##  Location effects: speed.med ~ hour.cen + TL.cen
##
##              post.mean   l-95% CI   u-95% CI eff.samp  pMCMC
## (Intercept)  0.8438560  0.5193010  1.1731976     2691 <4e-04 ***
## hour.cen    -0.0009096 -0.0329975  0.0388752     2500  0.934
## TL.cen       0.9509422 -1.6111635  3.4573687     2715  0.485
## ---
## Signif. codes:  0 '***' 0.001 '**' 0.01 '*' 0.05 '.' 0.1 ' ' 1
```

```r
posterior.mode(speed.day3.4$Sol)
```

```
##  (Intercept)     hour.cen       TL.cen
##  0.886172952 -0.007891026  0.843824272
```

```r
HPDinterval(speed.day3.4$Sol)
```

```
##                   lower      upper
## (Intercept)  0.51930101 1.17319757
## hour.cen    -0.03299751 0.03887522
## TL.cen      -1.61116350 3.45736869
## attr(,"Probability")
## [1] 0.95
```

```r
posterior.mode(speed.day3.4$VCV)
```

```
## (Intercept):(Intercept).picomp    hour.cen:(Intercept).picomp
##                     0.437635740                   -0.027299389
##    (Intercept):hour.cen.picomp       hour.cen:hour.cen.picomp
##                    -0.027299389                    0.005429921
##                           units
##                     0.210484485
```

```r
HPDinterval(speed.day3.4$VCV)
```

```
##                                       lower        upper
## (Intercept):(Intercept).picomp  0.226510122 0.9788346948
## hour.cen:(Intercept).picomp    -0.065982474 0.0008464298
## (Intercept):hour.cen.picomp    -0.065982474 0.0008464298
## hour.cen:hour.cen.picomp        0.002059507 0.0117150982
## units                           0.181866617 0.2590720373
## attr(,"Probability")
## [1] 0.95
```

```r
# only ID repeatability
rpt.spd1 <- speed.day3.4$VCV[, "(Intercept):(Intercept).picomp"]/(speed.day3.4$VCV[,
    "(Intercept):(Intercept).picomp"] + speed.day3.4$VCV[, "hour.cen:hour.cen.picomp"] +
    speed.day3.4$VCV[, "units"])
posterior.mode(rpt.spd1)
```

```
##      var1
## 0.7046443
```

```r
HPDinterval(rpt.spd1)
```

```
##         lower     upper
## var1 0.556949 0.8354121
## attr(,"Probability")
## [1] 0.95
```

```r
# to get marginal R2 to explain fixed effects variance
vmVarF <- numeric(2500)

for (i in 1:2500) {
    Var <- var(as.vector(speed.day3.4$Sol[i, ] %*% t(speed.day3.4$X)))
    vmVarF[i] <- Var
}


R2m <- vmVarF/(vmVarF + speed.day3.4$VCV[, 1] + speed.day3.4$VCV[, 4] + speed.day3.4$VCV[,
    5])

posterior.mode(R2m)
```

```
##         var1
## 0.001302835
```

```
HPDinterval(R2m)
```

```
##               lower     upper
## var1 4.990263e-06 0.1179169
## attr(,"Probability")
## [1] 0.95
```

```
R2c <- (vmVarF + speed.day3.4$VCV[, 1])/(vmVarF + speed.day3.4$VCV[, 1] + speed.day3.4$VCV[,
    4] + speed.day3.4$VCV[, 5])

posterior.mode(R2c)
```

```
##      var1
## 0.7493815
```

```
HPDinterval(R2c)
```

```
##          lower     upper
## var1 0.5786247 0.8447411
## attr(,"Probability")
## [1] 0.95
```

```
day4 <- hourly.com %>%
    filter(obs == 4) %>%
    mutate(hour.cen = hour - 1, TL.cen = TL - (sum(unique(TL))/26))

# Null model -----------
set.seed(58)
speed.day4.0 <- MCMCglmm(speed.med ~ hour.cen + TL.cen, data = day4, family = "gaussian",
    prior = prior.null, nitt = 510000, burnin = 10000, thin = 200, verbose = F)


# Intercepts ID ----------------------
set.seed(3432)
speed.day4.1 <- MCMCglmm(speed.med ~ hour.cen + TL.cen, random = ~picomp, data = day4,
    family = "gaussian", prior = prior.id, nitt = 510000, burnin = 10000, thin = 200,
    verbose = F)

# only including intercepts for mother ------
set.seed(42021)
speed.day4.2 <- MCMCglmm(speed.med ~ hour.cen + TL.cen, random = ~mother, data = day4,
    family = "gaussian", prior = prior.id, nitt = 510000, burnin = 10000, thin = 200,
    verbose = F)


# Intercepts ID and Mom-----------
```

```r
set.seed(258)
speed.day4.3 <- MCMCglmm(speed.med ~ hour.cen + TL.cen, random = ~picomp + mother,
    data = day4, family = "gaussian", prior = prior.id.mom, nitt = 510000, burnin = 10000,
    thin = 200, verbose = F)


# Intercepts and Slopes ID -----------------------
set.seed(472)
speed.day4.4 <- MCMCglmm(speed.med ~ hour.cen + TL.cen, random = ~us(1 + hour.cen):picomp,
    data = day4, family = "gaussian", prior = prior.id.slope, nitt = 510000,
    burnin = 10000, thin = 200, verbose = F)

# Intercepts Mom, Slopes Mom ----------------
set.seed(318)
speed.day4.5 <- MCMCglmm(speed.med ~ hour.cen + TL.cen, random = ~us(1 + hour.cen):mother,
    data = day4, family = "gaussian", prior = prior.id.slope, nitt = 510000,
    burnin = 10000, thin = 200, verbose = F)


# Intercepts ID and Mom, Slopes ID ----------------
set.seed(918)
speed.day4.6 <- MCMCglmm(speed.med ~ hour.cen + TL.cen, random = ~us(1 + hour.cen):picomp +
    mother, data = day4, family = "gaussian", prior = prior.id.slope.mom, nitt = 510000,
    burnin = 10000, thin = 200, verbose = F)


# Intercepts ID & Mom, Slopes ID & Mom -----------------
set.seed(78)
speed.day4.7 <- MCMCglmm(speed.med ~ hour.cen + TL.cen, random = ~us(1 + hour.cen):picomp +
    us(1 + hour.cen):mother, data = day4, family = "gaussian", prior = prior.id.slope.mom.slope,
    nitt = 510000, burnin = 10000, thin = 200, verbose = F)



DIC(speed.day4.0, speed.day4.1, speed.day4.2, speed.day4.3, speed.day4.4, speed.day4.5,
    speed.day4.6, speed.day4.7)
```

> Day 4:

```
##              df      DIC
## speed.day4.0  4 678.0763
## speed.day4.1  5 492.8375
## speed.day4.2  5 599.8889
## speed.day4.3  6 492.8026
## speed.day4.4  6 483.3065
## speed.day4.5  6 600.5203
## speed.day4.6  7 482.0919
## speed.day4.7  8 483.2055
```

.. report

```r
set.seed(472)
speed.day4.4 <- MCMCglmm(speed.med ~ hour.cen + TL.cen, random = ~us(1 + hour.cen):picomp,
    data = day4, family = "gaussian", prior = prior.id.slope, nitt = 510000,
    burnin = 10000, thin = 200, verbose = F)


summary(speed.day4.4)
```

```
##
##  Iterations = 10001:509801
##  Thinning interval  = 200
##  Sample size  = 2500
##
##  DIC: 483.3065
##
##  G-structure:  ~us(1 + hour.cen):picomp
##
##                                 post.mean   l-95% CI u-95% CI eff.samp
## (Intercept):(Intercept).picomp  0.364036  1.169e-01 0.666641     2355
## hour.cen:(Intercept).picomp     0.002349 -2.086e-02 0.024215     2223
## (Intercept):hour.cen.picomp     0.002349 -2.086e-02 0.024215     2223
## hour.cen:hour.cen.picomp        0.002907  2.402e-08 0.006599     1895
##
##  R-structure:  ~units
##
##       post.mean l-95% CI u-95% CI eff.samp
## units    0.2989   0.2466   0.3571     2500
##
##  Location effects: speed.med ~ hour.cen + TL.cen
##
##              post.mean   l-95% CI   u-95% CI eff.samp  pMCMC
## (Intercept)  0.7649941  0.4186849  1.1234203     2500 <4e-04 ***
## hour.cen     0.0307448  0.0008879  0.0606228     2500 0.0464 *
## TL.cen       0.4757478 -2.1793124  2.9311744     2326 0.6984
## ---
## Signif. codes:  0 '***' 0.001 '**' 0.01 '*' 0.05 '.' 0.1 ' ' 1
```

```r
posterior.mode(speed.day4.4$Sol)
```

```
## (Intercept)    hour.cen      TL.cen
##  0.83858349  0.03056214  0.56645443
```

```r
HPDinterval(speed.day4.4$Sol)
```

```
##                    lower      upper
## (Intercept)  0.4186849027 1.12342030
## hour.cen     0.0008878536 0.06062279
## TL.cen      -2.1793123708 2.93117444
## attr(,"Probability")
## [1] 0.95
```

```r
posterior.mode(speed.day4.4$VCV)
```

```
## (Intercept):(Intercept).picomp    hour.cen:(Intercept).picomp
##                     0.284698740                    0.006921316
##    (Intercept):hour.cen.picomp      hour.cen:hour.cen.picomp
##                     0.006921316                    0.001712658
##                           units
##                     0.301888590
```

```r
HPDinterval(speed.day4.4$VCV)
```

```
##                                         lower       upper
## (Intercept):(Intercept).picomp  1.169135e-01 0.666640687
## hour.cen:(Intercept).picomp    -2.085552e-02 0.024214679
## (Intercept):hour.cen.picomp    -2.085552e-02 0.024214679
## hour.cen:hour.cen.picomp        2.402187e-08 0.006598563
## units                           2.466394e-01 0.357115867
## attr(,"Probability")
## [1] 0.95
```

```r
# only ID repeatability
rpt.spd1 <- speed.day4.4$VCV[, "(Intercept):(Intercept).picomp"]/(speed.day4.4$VCV[,
    "(Intercept):(Intercept).picomp"] + speed.day4.4$VCV[, "hour.cen:hour.cen.picomp"] +
    speed.day4.4$VCV[, "units"])
posterior.mode(rpt.spd1)
```

```
##      var1
## 0.5735896
```

```r
HPDinterval(rpt.spd1)
```

```
##          lower     upper
## var1 0.3309984 0.7238141
## attr(,"Probability")
## [1] 0.95
```

```r
# to get marginal R2 to explain fixed effects variance
vmVarF <- numeric(2500)

for (i in 1:2500) {
    Var <- var(as.vector(speed.day4.4$Sol[i, ] %*% t(speed.day4.4$X)))
    vmVarF[i] <- Var
}


R2m <- vmVarF/(vmVarF + speed.day4.4$VCV[, 1] + speed.day4.4$VCV[, 4] + speed.day4.4$VCV[,
    5])

posterior.mode(R2m)
```

```
##        var1
## 0.01609207
```

```
HPDinterval(R2m)
```

```
##              lower     upper
## var1 0.0001082935 0.1168187
## attr(,"Probability")
## [1] 0.95
```

```
R2c <- (vmVarF + speed.day4.4$VCV[, 1])/(vmVarF + speed.day4.4$VCV[, 1] + speed.day4.4$VCV[,
    4] + speed.day4.4$VCV[, 5])

posterior.mode(R2c)
```

```
##      var1
## 0.5181292
```

```
HPDinterval(R2c)
```

```
##          lower     upper
## var1 0.353436 0.7350063
## attr(,"Probability")
## [1] 0.95
```

```
day5 <- hourly.com %>%
    filter(obs == 5) %>%
    mutate(hour.cen = hour - 1, TL.cen = TL - (sum(unique(TL))/26))

# Null model -----------
set.seed(98)
speed.day5.0 <- MCMCglmm(speed.med ~ hour.cen + TL.cen, data = day5, family = "gaussian",
    prior = prior.null, nitt = 510000, burnin = 10000, thin = 200, verbose = F)


# Intercepts ID ----------------------
set.seed(432)
speed.day5.1 <- MCMCglmm(speed.med ~ hour.cen + TL.cen, random = ~picomp, data = day5,
    family = "gaussian", prior = prior.id, nitt = 510000, burnin = 10000, thin = 200,
    verbose = F)

# only including intercepts for mother ------
set.seed(4221)
speed.day5.2 <- MCMCglmm(speed.med ~ hour.cen + TL.cen, random = ~mother, data = day5,
    family = "gaussian", prior = prior.id, nitt = 510000, burnin = 10000, thin = 200,
    verbose = F)


# Intercepts ID and Mom-----------
```

```r
set.seed(58)
speed.day5.3 <- MCMCglmm(speed.med ~ hour.cen + TL.cen, random = ~picomp + mother,
    data = day5, family = "gaussian", prior = prior.id.mom, nitt = 510000, burnin = 10000,
    thin = 200, verbose = F)


# Intercepts and Slopes ID -----------------------
set.seed(472)
speed.day5.4 <- MCMCglmm(speed.med ~ hour.cen + TL.cen, random = ~us(1 + hour.cen):picomp,
    data = day5, family = "gaussian", prior = prior.id.slope, nitt = 510000,
    burnin = 10000, thin = 200, verbose = F)

# Intercepts Mom, Slopes Mom ----------------
set.seed(318)
speed.day5.5 <- MCMCglmm(speed.med ~ hour.cen + TL.cen, random = ~us(1 + hour.cen):mother,
    data = day5, family = "gaussian", prior = prior.id.slope, nitt = 510000,
    burnin = 10000, thin = 200, verbose = F)


# Intercepts ID and Mom, Slopes ID ----------------
set.seed(918)
speed.day5.6 <- MCMCglmm(speed.med ~ hour.cen + TL.cen, random = ~us(1 + hour.cen):picomp +
    mother, data = day5, family = "gaussian", prior = prior.id.slope.mom, nitt = 510000,
    burnin = 10000, thin = 200, verbose = F)


# Intercepts ID & Mom, Slopes ID & Mom -----------------
set.seed(78)
speed.day5.7 <- MCMCglmm(speed.med ~ hour.cen + TL.cen, random = ~us(1 + hour.cen):picomp +
    us(1 + hour.cen):mother, data = day5, family = "gaussian", prior = prior.id.slope.mom.slope,
    nitt = 510000, burnin = 10000, thin = 200, verbose = F)



DIC(speed.day5.0, speed.day5.1, speed.day5.2, speed.day5.3, speed.day5.4, speed.day5.5,
    speed.day5.6, speed.day5.7)
```

> **Day 5:**

```
##              df      DIC
## speed.day5.0  4 646.0225
## speed.day5.1  5 463.4091
## speed.day5.2  5 502.5157
## speed.day5.3  6 461.9713
## speed.day5.4  6 443.9833
## speed.day5.5  6 492.3501
## speed.day5.6  7 443.5182
## speed.day5.7  8 443.3452
```

.. report

```r
set.seed(472)
speed.day5.4 <- MCMCglmm(speed.med ~ hour.cen + TL.cen, random = ~us(1 + hour.cen):picomp,
    data = day5, family = "gaussian", prior = prior.id.slope, nitt = 510000,
    burnin = 10000, thin = 200, verbose = F)


summary(speed.day5.4)
```

```
##
##  Iterations = 10001:509801
##  Thinning interval  = 200
##  Sample size  = 2500
##
##  DIC: 443.9833
##
##  G-structure:  ~us(1 + hour.cen):picomp
##
##                                 post.mean   l-95% CI  u-95% CI eff.samp
## (Intercept):(Intercept).picomp  0.399373  0.1347813  0.71771     2500
## hour.cen:(Intercept).picomp    -0.007640 -0.0357521  0.01562     2500
## (Intercept):hour.cen.picomp    -0.007640 -0.0357521  0.01562     2500
## hour.cen:hour.cen.picomp        0.004122  0.0003598  0.00851     2500
##
##  R-structure:  ~units
##
##       post.mean l-95% CI u-95% CI eff.samp
## units    0.2615   0.2141    0.313     2500
##
##  Location effects: speed.med ~ hour.cen + TL.cen
##
##             post.mean  l-95% CI  u-95% CI eff.samp  pMCMC
## (Intercept)  0.542758  0.190271  0.864697     2500 0.0024 **
## hour.cen     0.009573 -0.024076  0.042209     2500 0.5288
## TL.cen       0.925000 -1.566604  3.618391     2500 0.4848
## ---
## Signif. codes:  0 '***' 0.001 '**' 0.01 '*' 0.05 '.' 0.1 ' ' 1
```

```r
posterior.mode(speed.day5.4$Sol)
```

```
## (Intercept)    hour.cen      TL.cen
##  0.58357119  0.00677083  1.00824251
```

```r
HPDinterval(speed.day5.4$Sol)
```

```
##                   lower     upper
## (Intercept)  0.19027117 0.8646972
## hour.cen    -0.02407554 0.0422091
## TL.cen      -1.56660385 3.6183910
## attr(,"Probability")
## [1] 0.95
```

```r
posterior.mode(speed.day5.4$VCV)
```

```
## (Intercept):(Intercept).picomp    hour.cen:(Intercept).picomp
##                     0.337489557                   -0.003416255
##    (Intercept):hour.cen.picomp      hour.cen:hour.cen.picomp
##                    -0.003416255                    0.002585669
##                           units
##                     0.249961502
```

```r
HPDinterval(speed.day5.4$VCV)
```

```
##                                          lower       upper
## (Intercept):(Intercept).picomp  0.1347813018 0.717714554
## hour.cen:(Intercept).picomp    -0.0357520685 0.015624729
## (Intercept):hour.cen.picomp    -0.0357520685 0.015624729
## hour.cen:hour.cen.picomp        0.0003598117 0.008509955
## units                           0.2141137225 0.313004669
## attr(,"Probability")
## [1] 0.95
```

```r
# only ID repeatability
rpt.spd1 <- speed.day5.4$VCV[, "(Intercept):(Intercept).picomp"]/(speed.day5.4$VCV[,
    "(Intercept):(Intercept).picomp"] + speed.day5.4$VCV[, "hour.cen:hour.cen.picomp"] +
    speed.day5.4$VCV[, "units"])
posterior.mode(rpt.spd1)
```

```
##      var1
## 0.5682457
```

```r
HPDinterval(rpt.spd1)
```

```
##          lower     upper
## var1 0.3768156 0.7541062
## attr(,"Probability")
## [1] 0.95
```

```r
# to get marginal R2 to explain fixed effects variance
vmVarF <- numeric(2500)

for (i in 1:2500) {
    Var <- var(as.vector(speed.day5.4$Sol[i, ] %*% t(speed.day5.4$X)))
    vmVarF[i] <- Var
}


R2m <- vmVarF/(vmVarF + speed.day5.4$VCV[, 1] + speed.day5.4$VCV[, 4] + speed.day5.4$VCV[,
    5])

posterior.mode(R2m)
```

```
##        var1
## 0.002495821
```

```
HPDinterval(R2m)
```

```
##              lower    upper
## var1 3.000935e-06 0.131921
## attr(,"Probability")
## [1] 0.95
```

```
R2c <- (vmVarF + speed.day5.4$VCV[, 1])/(vmVarF + speed.day5.4$VCV[, 1] + speed.day5.4$VCV[,
    4] + speed.day5.4$VCV[, 5])

posterior.mode(R2c)
```

```
##      var1
## 0.6113138
```

```
HPDinterval(R2c)
```

```
##          lower     upper
## var1 0.4187854 0.7807376
## attr(,"Probability")
## [1] 0.95
```

```
day6 <- hourly.com %>%
    filter(obs == 6) %>%
    mutate(hour.cen = hour - 1, TL.cen = TL - (sum(unique(TL))/26))

# Null model -----------
set.seed(58)
speed.day6.0 <- MCMCglmm(speed.med ~ hour.cen + TL.cen, data = day6, family = "gaussian",
    prior = prior.null, nitt = 510000, burnin = 10000, thin = 200, verbose = F)


# Intercepts ID ----------------------
set.seed(3432)
speed.day6.1 <- MCMCglmm(speed.med ~ hour.cen + TL.cen, random = ~picomp, data = day6,
    family = "gaussian", prior = prior.id, nitt = 510000, burnin = 10000, thin = 200,
    verbose = F)

# only including intercepts for mother ------
set.seed(42021)
speed.day6.2 <- MCMCglmm(speed.med ~ hour.cen + TL.cen, random = ~mother, data = day6,
    family = "gaussian", prior = prior.id, nitt = 510000, burnin = 10000, thin = 200,
    verbose = F)


# Intercepts ID and Mom-----------
```

```
set.seed(258)
speed.day6.3 <- MCMCglmm(speed.med ~ hour.cen + TL.cen, random = ~picomp + mother,
    data = day6, family = "gaussian", prior = prior.id.mom, nitt = 510000, burnin = 10000,
    thin = 200, verbose = F)


# Intercepts and Slopes ID -----------------------
set.seed(472)
speed.day6.4 <- MCMCglmm(speed.med ~ hour.cen + TL.cen, random = ~us(1 + hour.cen):picomp,
    data = day6, family = "gaussian", prior = prior.id.slope, nitt = 510000,
    burnin = 10000, thin = 200, verbose = F)

# Intercepts Mom, Slopes Mom ----------------
set.seed(318)
speed.day6.5 <- MCMCglmm(speed.med ~ hour.cen + TL.cen, random = ~us(1 + hour.cen):mother,
    data = day6, family = "gaussian", prior = prior.id.slope, nitt = 510000,
    burnin = 10000, thin = 200, verbose = F)


# Intercepts ID and Mom, Slopes ID ----------------
set.seed(918)
speed.day6.6 <- MCMCglmm(speed.med ~ hour.cen + TL.cen, random = ~us(1 + hour.cen):picomp +
    mother, data = day6, family = "gaussian", prior = prior.id.slope.mom, nitt = 510000,
    burnin = 10000, thin = 200, verbose = F)


# Intercepts ID & Mom, Slopes ID & Mom -----------------
set.seed(78)
speed.day6.7 <- MCMCglmm(speed.med ~ hour.cen + TL.cen, random = ~us(1 + hour.cen):picomp +
    us(1 + hour.cen):mother, data = day6, family = "gaussian", prior = prior.id.slope.mom.slope,
    nitt = 510000, burnin = 10000, thin = 200, verbose = F)



DIC(speed.day6.0, speed.day6.1, speed.day6.2, speed.day6.3, speed.day6.4, speed.day6.5,
    speed.day6.6, speed.day6.7)
```

> Day 6:

```
##              df      DIC
## speed.day6.0  4 650.8213
## speed.day6.1  5 503.0155
## speed.day6.2  5 510.8793
## speed.day6.3  6 499.6059
## speed.day6.4  6 448.9886
## speed.day6.5  6 458.4026
## speed.day6.6  7 448.2602
## speed.day6.7  8 438.9988
```

Interestingly, on Day 6, the most well supported model includes random intercepts and slopes for mother as well at individual (model 7). let's see how much variation is explained by mother compared to individual

.. report

```r
set.seed(78)
speed.day6.7 <- MCMCglmm(speed.med ~ hour.cen + TL.cen, random = ~us(1 + hour.cen):picomp +
    us(1 + hour.cen):mother, data = day6, family = "gaussian", prior = prior.id.slope.mom.slope,
    nitt = 510000, burnin = 10000, thin = 200, verbose = F)

summary(speed.day6.7)
```

```
##
##  Iterations = 10001:509801
##  Thinning interval  = 200
##  Sample size  = 2500
##
##  DIC: 438.9988
##
##  G-structure:  ~us(1 + hour.cen):picomp
##
##                                 post.mean   l-95% CI u-95% CI eff.samp
## (Intercept):(Intercept).picomp  0.0676712  4.533e-09 0.182465     2315
## hour.cen:(Intercept).picomp    -0.0020200 -1.382e-02 0.004965     2210
## (Intercept):hour.cen.picomp    -0.0020200 -1.382e-02 0.004965     2210
## hour.cen:hour.cen.picomp        0.0006506  6.228e-11 0.002393     2119
##
##                ~us(1 + hour.cen):mother
##
##                                post.mean  l-95% CI u-95% CI eff.samp
## (Intercept):(Intercept).mother   0.57962  0.043676  1.45485     2500
## hour.cen:(Intercept).mother     -0.03896 -0.156343  0.05418     2500
## (Intercept):hour.cen.mother     -0.03896 -0.156343  0.05418     2500
## hour.cen:hour.cen.mother         0.01604  0.001352  0.04194     2500
##
##  R-structure:  ~units
##
##       post.mean l-95% CI u-95% CI eff.samp
## units    0.2576   0.2137   0.3054     2427
##
##  Location effects: speed.med ~ hour.cen + TL.cen
##
##             post.mean l-95% CI u-95% CI eff.samp  pMCMC
## (Intercept)   0.47111 -0.12257  0.98021     2500 0.0968 .
## hour.cen      0.04927 -0.03934  0.14052     2500 0.2440
## TL.cen       -0.86540 -2.77445  1.03228     2240 0.3496
## ---
## Signif. codes:  0 '***' 0.001 '**' 0.01 '*' 0.05 '.' 0.1 ' ' 1
```

```r
posterior.mode(speed.day6.7$Sol)
```

```
## (Intercept)    hour.cen      TL.cen
##  0.41659254  0.06462006 -0.54099237
```

```r
HPDinterval(speed.day6.7$Sol)
```

```
##                   lower     upper
```

```
## (Intercept) -0.12256943 0.9802118
## hour.cen    -0.03933574 0.1405249
## TL.cen      -2.77444565 1.0322796
## attr(,"Probability")
## [1] 0.95
```

```r
posterior.mode(speed.day6.7$VCV)
```

```
## (Intercept):(Intercept).picomp    hour.cen:(Intercept).picomp
##                   4.284007e-02                   3.078618e-05
##    (Intercept):hour.cen.picomp       hour.cen:hour.cen.picomp
##                   3.078618e-05                   2.375753e-05
## (Intercept):(Intercept).mother    hour.cen:(Intercept).mother
##                   2.776558e-01                  -1.414078e-02
##    (Intercept):hour.cen.mother       hour.cen:hour.cen.mother
##                  -1.414078e-02                   8.066965e-03
##                          units
##                   2.471178e-01
```

```r
HPDinterval(speed.day6.7$VCV)
```

```
##                                        lower       upper
## (Intercept):(Intercept).picomp  4.532742e-09 0.182465267
## hour.cen:(Intercept).picomp    -1.381577e-02 0.004965170
## (Intercept):hour.cen.picomp    -1.381577e-02 0.004965170
## hour.cen:hour.cen.picomp        6.227928e-11 0.002392794
## (Intercept):(Intercept).mother  4.367649e-02 1.454847050
## hour.cen:(Intercept).mother    -1.563430e-01 0.054180772
## (Intercept):hour.cen.mother    -1.563430e-01 0.054180772
## hour.cen:hour.cen.mother        1.351595e-03 0.041943481
## units                           2.137061e-01 0.305375147
## attr(,"Probability")
## [1] 0.95
```

```r
# proportion of variation due to individual intercepts
day6.id <- speed.day6.7$VCV[, "(Intercept):(Intercept).picomp"]/(speed.day6.7$VCV[,
    "(Intercept):(Intercept).picomp"] + speed.day6.7$VCV[, "hour.cen:hour.cen.picomp"] +
    speed.day6.7$VCV[, "(Intercept):(Intercept).mother"] + speed.day6.7$VCV[,
    "hour.cen:hour.cen.mother"] + speed.day6.7$VCV[, "units"])
posterior.mode(day6.id)
```

```
##       var1
## 0.02934972
```

```r
HPDinterval(day6.id)
```

```
##             lower     upper
## var1 6.242116e-09 0.2390201
## attr(,"Probability")
## [1] 0.95
```

```r
# proportion of variation due mother intercepts
day6.mom <- speed.day6.7$VCV[, "(Intercept):(Intercept).mother"]/(speed.day6.7$VCV[,
    "(Intercept):(Intercept).picomp"] + speed.day6.7$VCV[, "hour.cen:hour.cen.picomp"] +
    speed.day6.7$VCV[, "(Intercept):(Intercept).mother"] + speed.day6.7$VCV[,
    "hour.cen:hour.cen.mother"] + speed.day6.7$VCV[, "units"])
posterior.mode(day6.mom)
```

```
##      var1
## 0.5665137
```

```r
HPDinterval(day6.mom)
```

```
##          lower    upper
## var1 0.2633498 0.862011
## attr(,"Probability")
## [1] 0.95
```

```r
# proportion of variation due mother intercepts
day6.units <- speed.day6.7$VCV[, "units"]/(speed.day6.7$VCV[, "(Intercept):(Intercept).picomp"] +
    speed.day6.7$VCV[, "hour.cen:hour.cen.picomp"] + speed.day6.7$VCV[, "(Intercept):(Intercept).mother
    speed.day6.7$VCV[, "hour.cen:hour.cen.mother"] + speed.day6.7$VCV[, "units"])
posterior.mode(day6.units)
```

```
##      var1
## 0.3731586
```

```r
HPDinterval(day6.units)
```

```
##           lower     upper
## var1 0.09599891 0.5509751
## attr(,"Probability")
## [1] 0.95
```

```r
# to get marginal R2 to explain fixed effects variance
vmVarF <- numeric(2500)

for (i in 1:2500) {
    Var <- var(as.vector(speed.day6.7$Sol[i, ] %*% t(speed.day6.7$X)))
    vmVarF[i] <- Var
}


R2m <- vmVarF/(vmVarF + speed.day6.7$VCV[, 1] + speed.day6.7$VCV[, 4] + speed.day6.7$VCV[,
    5])

posterior.mode(R2m)
```

```
##       var1
## 0.02048799
```

```r
HPDinterval(R2m)
```

```
##             lower     upper
## var1 1.908369e-06 0.2878823
## attr(,"Probability")
## [1] 0.95
```

```r
R2c <- (vmVarF + speed.day6.7$VCV[, 1])/(vmVarF + speed.day6.7$VCV[, 1] + speed.day6.7$VCV[,
    4] + speed.day6.7$VCV[, 5])

posterior.mode(R2c)
```

```
##      var1
## 0.1211246
```

```r
HPDinterval(R2c)
```

```
##            lower    upper
## var1 0.009055556 0.524962
## attr(,"Probability")
## [1] 0.95
```

```r
day7 <- hourly.com %>%
    filter(obs == 7) %>%
    mutate(hour.cen = hour - 1, TL.cen = TL - (sum(unique(TL))/26))

# Null model -----------
set.seed(58)
speed.day7.0 <- MCMCglmm(speed.med ~ hour.cen + TL.cen, data = day7, family = "gaussian",
    prior = prior.null, nitt = 510000, burnin = 10000, thin = 200, verbose = F)


# Intercepts ID ----------------------
set.seed(3432)
speed.day7.1 <- MCMCglmm(speed.med ~ hour.cen + TL.cen, random = ~picomp, data = day7,
    family = "gaussian", prior = prior.id, nitt = 510000, burnin = 10000, thin = 200,
    verbose = F)

# only including intercepts for mother ------
set.seed(42021)
speed.day7.2 <- MCMCglmm(speed.med ~ hour.cen + TL.cen, random = ~mother, data = day7,
    family = "gaussian", prior = prior.id, nitt = 510000, burnin = 10000, thin = 200,
    verbose = F)


# Intercepts ID and Mom-----------
set.seed(258)
speed.day7.3 <- MCMCglmm(speed.med ~ hour.cen + TL.cen, random = ~picomp + mother,
    data = day7, family = "gaussian", prior = prior.id.mom, nitt = 510000, burnin = 10000,
```

```r
    thin = 200, verbose = F)


# Intercepts and Slopes ID -----------------------
set.seed(472)
speed.day7.4 <- MCMCglmm(speed.med ~ hour.cen + TL.cen, random = ~us(1 + hour.cen):picomp,
    data = day7, family = "gaussian", prior = prior.id.slope, nitt = 510000,
    burnin = 10000, thin = 200, verbose = F)

# Intercepts Mom, Slopes Mom ----------------
set.seed(318)
speed.day7.5 <- MCMCglmm(speed.med ~ hour.cen + TL.cen, random = ~us(1 + hour.cen):mother,
    data = day7, family = "gaussian", prior = prior.id.slope, nitt = 510000,
    burnin = 10000, thin = 200, verbose = F)


# Intercepts ID and Mom, Slopes ID ----------------
set.seed(968)
speed.day7.6 <- MCMCglmm(speed.med ~ hour.cen + TL.cen, random = ~us(1 + hour.cen):picomp +
    mother, data = day7, family = "gaussian", prior = prior.id.slope.mom, nitt = 510000,
    burnin = 10000, thin = 200, verbose = F)


# Intercepts ID & Mom, Slopes ID & Mom -----------------
set.seed(178)
speed.day7.7 <- MCMCglmm(speed.med ~ hour.cen + TL.cen, random = ~us(1 + hour.cen):picomp +
    us(1 + hour.cen):mother, data = day7, family = "gaussian", prior = prior.id.slope.mom.slope,
    nitt = 510000, burnin = 10000, thin = 200, verbose = F)



DIC(speed.day7.0, speed.day7.1, speed.day7.2, speed.day7.3, speed.day7.4, speed.day7.5,
    speed.day7.6, speed.day7.7)
```

> **Day 7:**

```
##              df      DIC
## speed.day7.0  4 658.5002
## speed.day7.1  5 445.7669
## speed.day7.2  5 541.4688
## speed.day7.3  6 445.3436
## speed.day7.4  6 388.7121
## speed.day7.5  6 521.6844
## speed.day7.6  7 389.3534
## speed.day7.7  8 390.8515
```

.. report

```r
set.seed(472)
speed.day7.4 <- MCMCglmm(speed.med ~ hour.cen + TL.cen, random = ~us(1 + hour.cen):picomp,
    data = day7, family = "gaussian", prior = prior.id.slope, nitt = 510000,
    burnin = 10000, thin = 200, verbose = F)
```

```r
summary(speed.day7.4)
```

```
##
##  Iterations = 10001:509801
##  Thinning interval  = 200
##  Sample size  = 2500
##
##  DIC: 388.7121
##
##  G-structure:  ~us(1 + hour.cen):picomp
##
##                                post.mean  l-95% CI  u-95% CI eff.samp
## (Intercept):(Intercept).picomp  0.699977  0.296218  1.174234     2467
## hour.cen:(Intercept).picomp    -0.044601 -0.092315 -0.007016     2287
## (Intercept):hour.cen.picomp    -0.044601 -0.092315 -0.007016     2287
## hour.cen:hour.cen.picomp        0.007548  0.002721  0.013964     2293
##
##  R-structure:  ~units
##
##       post.mean l-95% CI u-95% CI eff.samp
## units    0.1993   0.1638   0.2376     2500
##
##  Location effects: speed.med ~ hour.cen + TL.cen
##
##             post.mean l-95% CI u-95% CI eff.samp  pMCMC
## (Intercept)   0.68566  0.33489  1.05783     2500 0.0008 ***
## hour.cen     -0.01223 -0.04893  0.02554     1948 0.5096
## TL.cen        0.73476 -1.87203  3.39216     2500 0.5720
## ---
## Signif. codes:  0 '***' 0.001 '**' 0.01 '*' 0.05 '.' 0.1 ' ' 1
```

```r
posterior.mode(speed.day7.4$Sol)
```

```
## (Intercept)    hour.cen      TL.cen
##  0.73428552 -0.01023717  0.66886278
```

```r
HPDinterval(speed.day7.4$Sol)
```

```
##                   lower      upper
## (Intercept)  0.33488596 1.05783060
## hour.cen    -0.04893163 0.02553764
## TL.cen      -1.87203097 3.39215605
## attr(,"Probability")
## [1] 0.95
```

```r
posterior.mode(speed.day7.4$VCV)
```

```
## (Intercept):(Intercept).picomp    hour.cen:(Intercept).picomp
##                    0.572253878                   -0.032205275
##    (Intercept):hour.cen.picomp       hour.cen:hour.cen.picomp
```

```
##                   -0.032205275                    0.006141325
##                          units
##                    0.203574279
```

```
HPDinterval(speed.day7.4$VCV)
```

```
##                                          lower        upper
## (Intercept):(Intercept).picomp  0.296218427  1.174233990
## hour.cen:(Intercept).picomp    -0.092315256 -0.007015817
## (Intercept):hour.cen.picomp    -0.092315256 -0.007015817
## hour.cen:hour.cen.picomp        0.002720734  0.013963580
## units                           0.163783228  0.237641681
## attr(,"Probability")
## [1] 0.95
```

```
# only ID repeatability
rpt.spd1 <- speed.day7.4$VCV[, "(Intercept):(Intercept).picomp"]/(speed.day7.4$VCV[,
    "(Intercept):(Intercept).picomp"] + speed.day7.4$VCV[, "hour.cen:hour.cen.picomp"] +
    speed.day7.4$VCV[, "units"])
posterior.mode(rpt.spd1)
```

```
##      var1
## 0.7725805
```

```
HPDinterval(rpt.spd1)
```

```
##          lower     upper
## var1 0.6323528 0.8656939
## attr(,"Probability")
## [1] 0.95
```

```
# to get marginal R2 to explain fixed effects variance
vmVarF <- numeric(2500)

for (i in 1:2500) {
    Var <- var(as.vector(speed.day7.4$Sol[i, ] %*% t(speed.day7.4$X)))
    vmVarF[i] <- Var
}


R2m <- vmVarF/(vmVarF + speed.day7.4$VCV[, 1] + speed.day7.4$VCV[, 4] + speed.day7.4$VCV[,
    5])

posterior.mode(R2m)
```

```
##        var1
## 0.003165999
```

```
HPDinterval(R2m)
```

```
##              lower     upper
## var1 1.431154e-06 0.0905166
## attr(,"Probability")
## [1] 0.95
```

```
R2c <- (vmVarF + speed.day7.4$VCV[, 1])/(vmVarF + speed.day7.4$VCV[, 1] + speed.day7.4$VCV[,
    4] + speed.day7.4$VCV[, 5])

posterior.mode(R2c)
```

```
##      var1
## 0.7804428
```

```
HPDinterval(R2c)
```

```
##          lower     upper
## var1 0.6514933 0.8777478
## attr(,"Probability")
## [1] 0.95
```

Week 1: Now let's just look across the entire first week of life to make sure that individuals are consistent ACROSS days as well

Bottom line: Yes, they are!

```
# Null model -----------
set.seed(58)
speed.week1.0 <- MCMCglmm(speed.med ~ obs.cen + TL, data = hourly.com[which(hourly.com$week ==
    1), ], family = "gaussian", prior = prior.null, nitt = 510000, burnin = 10000,
    thin = 200, verbose = F)


# Intercepts ID ----------------------
set.seed(3432)
speed.week1.1 <- MCMCglmm(speed.med ~ obs.cen + TL, random = ~picomp, data = hourly.com[which(hourly.co
    1), ], family = "gaussian", prior = prior.id, nitt = 510000, burnin = 10000,
    thin = 200, verbose = F)

# only including intercepts for mother ------
set.seed(42021)
speed.week1.2 <- MCMCglmm(speed.med ~ obs.cen + TL, random = ~mother, data = hourly.com[which(hourly.co
    1), ], family = "gaussian", prior = prior.id, nitt = 510000, burnin = 10000,
    thin = 200, verbose = F)


# Intercepts ID and Mom-----------
set.seed(258)
speed.week1.3 <- MCMCglmm(speed.med ~ obs.cen + TL, random = ~picomp + mother,
    data = hourly.com[which(hourly.com$week == 1), ], family = "gaussian", prior = prior.id.mom,
```

```
    nitt = 510000, burnin = 10000, thin = 200, verbose = F)


# Intercepts and Slopes ID -----------------------
set.seed(472)
speed.week1.4 <- MCMCglmm(speed.med ~ obs.cen + TL, random = ~us(1 + obs.cen):picomp,
    data = hourly.com[which(hourly.com$week == 1), ], family = "gaussian", prior = prior.id.slope,
    nitt = 510000, burnin = 10000, thin = 200, verbose = F)

# Intercepts Mom, Slopes Mom ----------------
set.seed(318)
speed.week1.5 <- MCMCglmm(speed.med ~ obs.cen + TL, random = ~us(1 + obs.cen):mother,
    data = hourly.com[which(hourly.com$week == 1), ], family = "gaussian", prior = prior.id.slope,
    nitt = 510000, burnin = 10000, thin = 200, verbose = F)


# Intercepts ID and Mom, Slopes ID ----------------
set.seed(918)
speed.week1.6 <- MCMCglmm(speed.med ~ obs.cen + TL, random = ~us(1 + obs.cen):picomp +
    mother, data = hourly.com[which(hourly.com$week == 1), ], family = "gaussian",
    prior = prior.id.slope.mom, nitt = 510000, burnin = 10000, thin = 200, verbose = F)


# Intercepts ID & Mom, Slopes ID & Mom -----------------
set.seed(78)
speed.week1.7 <- MCMCglmm(speed.med ~ obs.cen + TL, random = ~us(1 + obs.cen):picomp +
    us(1 + obs.cen):mother, data = hourly.com[which(hourly.com$week == 1), ],
    family = "gaussian", prior = prior.id.slope.mom.slope, nitt = 510000, burnin = 10000,
    thin = 200, verbose = F)




DIC(speed.week1.0, speed.week1.1, speed.week1.2, speed.week1.3, speed.week1.4,
    speed.week1.5, speed.week1.6, speed.week1.7)
```

> model comparison

```
##                 df      DIC
## speed.week1.0  4 4617.310
## speed.week1.1  5 3772.220
## speed.week1.2  5 3999.101
## speed.week1.3  6 3771.720
## speed.week1.4  6 3488.369
## speed.week1.5  6 3900.975
## speed.week1.6  7 3487.796
## speed.week1.7  8 3487.927
```

```
set.seed(472)
speed.week1.4 <- MCMCglmm(speed.med ~ obs.cen * growth.rate + TL, random = ~us(1 +
```

```r
    obs.cen):picomp, data = hourly.com[which(hourly.com$week == 1), ], family = "gaussian",
    prior = prior.id.slope, nitt = 510000, burnin = 10000, thin = 200, verbose = F)

posterior.mode(speed.week1.4$Sol)
```

.. model to report

```
##          (Intercept)              obs.cen          growth.rate                   TL
##           1.26213832          -0.02789741          -1.36396027           0.67572988
## obs.cen:growth.rate
##          -0.30901087
```

```r
HPDinterval(speed.week1.4$Sol)
```

```
##                          lower      upper
## (Intercept)         -1.4274044 3.32154528
## obs.cen             -0.1664903 0.06792222
## growth.rate         -4.3257810 2.40909607
## TL                  -1.5222118 2.63208391
## obs.cen:growth.rate -1.0122724 0.34493051
## attr(,"Probability")
## [1] 0.95
```

```r
posterior.mode(speed.week1.4$VCV)
```

```
## (Intercept):(Intercept).picomp     obs.cen:(Intercept).picomp
##                     0.38697595                    -0.03979564
##     (Intercept):obs.cen.picomp         obs.cen:obs.cen.picomp
##                    -0.03979564                     0.01338808
##                          units
##                     0.33988932
```

```r
HPDinterval(speed.week1.4$VCV)
```

```
##                                      lower       upper
## (Intercept):(Intercept).picomp  0.222792432  0.78379154
## obs.cen:(Intercept).picomp     -0.110576855 -0.01674356
## (Intercept):obs.cen.picomp     -0.110576855 -0.01674356
## obs.cen:obs.cen.picomp          0.008630652  0.02985858
## units                           0.320063845  0.36347974
## attr(,"Probability")
## [1] 0.95
```

```r
# only ID repeatability
rpt.spd.wk1 <- speed.week1.4$VCV[, "(Intercept):(Intercept).picomp"]/(speed.week1.4$VCV[,
    "(Intercept):(Intercept).picomp"] + speed.week1.4$VCV[, "obs.cen:obs.cen.picomp"] +
    speed.week1.4$VCV[, "units"])
posterior.mode(rpt.spd.wk1)
```

```
##      var1
## 0.5670351
```

```r
HPDinterval(rpt.spd.wk1)
```

```
##          lower     upper
## var1 0.4105794 0.7026743
## attr(,"Probability")
## [1] 0.95
```

```r
# to get marginal R2 to explain fixed effects variance
vmVarF <- numeric(2500)

for (i in 1:2500) {
    Var <- var(as.vector(speed.week1.4$Sol[i, ] %*% t(speed.week1.4$X)))
    vmVarF[i] <- Var
}


R2m <- vmVarF/(vmVarF + speed.week1.4$VCV[, 1] + speed.week1.4$VCV[, 4] + speed.week1.4$VCV[,
    5])

posterior.mode(R2m)
```

```
##       var1
## 0.07063754
```

```r
HPDinterval(R2m)
```

```
##           lower     upper
## var1 0.02535748 0.1911144
## attr(,"Probability")
## [1] 0.95
```

```r
R2c <- (vmVarF + speed.week1.4$VCV[, 1])/(vmVarF + speed.week1.4$VCV[, 1] +
    speed.week1.4$VCV[, 4] + speed.week1.4$VCV[, 5])

posterior.mode(R2c)
```

```
##      var1
## 0.5988204
```

```r
HPDinterval(R2c)
```

```
##          lower     upper
## var1 0.4665778 0.7238406
## attr(,"Probability")
## [1] 0.95
```

> **other behaviors** We also used the x,y coordinates returned by our tracking software to estimate a number of other behavioral variables. Here we test for repeatability in these behaviors in the first week of life.

```r
set.seed(309)
iqr.week1.id <- MCMCglmm(distmoved.iqr ~ obs.cen + TL, random = ~us(1 + obs.cen):picomp,
    data = hourly.com[which(hourly.com$week == 1), ], family = "gaussian", prior = prior.id.slope,
    nitt = 510000, burnin = 10000, thin = 200, verbose = F)

set.seed(504)
prop.week1.id <- MCMCglmm(propmoving ~ obs.cen + TL, random = ~us(1 + obs.cen):picomp,
    data = hourly.com[which(hourly.com$week == 1), ], family = "gaussian", prior = prior.id.slope,
    nitt = 510000, burnin = 10000, thin = 200, verbose = F)

set.seed(987)
border.week1.id <- MCMCglmm(borderdist.med ~ obs.cen + TL, random = ~us(1 +
    obs.cen):picomp, data = hourly.com[which(hourly.com$week == 1), ], family = "gaussian",
    prior = prior.id.slope, nitt = 510000, burnin = 10000, thin = 200, verbose = F)


posterior.mode(iqr.week1.id$VCV)
```

```
## (Intercept):(Intercept).picomp     obs.cen:(Intercept).picomp
##                       220.47605                      -29.27691
##     (Intercept):obs.cen.picomp         obs.cen:obs.cen.picomp
##                       -29.27691                       11.40182
##                           units
##                       293.47398
```

```r
HPDinterval(iqr.week1.id$VCV)
```

```
##                                      lower      upper
## (Intercept):(Intercept).picomp 127.236477 485.568785
## obs.cen:(Intercept).picomp     -65.813763  -3.113078
## (Intercept):obs.cen.picomp     -65.813763  -3.113078
## obs.cen:obs.cen.picomp           5.678902  20.607076
## units                          275.912058 312.029774
## attr(,"Probability")
## [1] 0.95
```

```r
iqr.rpt.wk1 <- iqr.week1.id$VCV[, "(Intercept):(Intercept).picomp"]/(iqr.week1.id$VCV[,
    "(Intercept):(Intercept).picomp"] + iqr.week1.id$VCV[, "obs.cen:obs.cen.picomp"] +
    iqr.week1.id$VCV[, "units"])
posterior.mode(iqr.rpt.wk1)
```

```
##      var1
## 0.4572179
```

```r
HPDinterval(iqr.rpt.wk1)
```

```
##          lower     upper
## var1 0.3389236 0.6365694
## attr(,"Probability")
## [1] 0.95
```

```r
posterior.mode(prop.week1.id$VCV)
```

```
## (Intercept):(Intercept).picomp     obs.cen:(Intercept).picomp
##                     0.030203876                   -0.002733661
##     (Intercept):obs.cen.picomp         obs.cen:obs.cen.picomp
##                    -0.002733661                    0.001427673
##                           units
##                     0.029817194
```

```r
HPDinterval(prop.week1.id$VCV)
```

```
##                                       lower        upper
## (Intercept):(Intercept).picomp  0.017108482 0.0606583440
## obs.cen:(Intercept).picomp     -0.007187632 0.0004364022
## (Intercept):obs.cen.picomp     -0.007187632 0.0004364022
## obs.cen:obs.cen.picomp          0.000735252 0.0027138604
## units                           0.028155557 0.0319569507
## attr(,"Probability")
## [1] 0.95
```

```r
prop.rpt.wk1 <- prop.week1.id$VCV[, "(Intercept):(Intercept).picomp"]/(prop.week1.id$VCV[,
    "(Intercept):(Intercept).picomp"] + prop.week1.id$VCV[, "obs.cen:obs.cen.picomp"] +
    prop.week1.id$VCV[, "units"])
posterior.mode(prop.rpt.wk1)
```

```
##      var1
## 0.5162739
```

```r
HPDinterval(prop.rpt.wk1)
```

```
##          lower   upper
## var1 0.3693184 0.65981
## attr(,"Probability")
## [1] 0.95
```

```r
posterior.mode(border.week1.id$VCV)
```

```
## (Intercept):(Intercept).picomp     obs.cen:(Intercept).picomp
##                      0.05435530                    -0.01104941
##     (Intercept):obs.cen.picomp         obs.cen:obs.cen.picomp
##                     -0.01104941                     0.73041207
##                           units
##                    306.37989111
```

```r
HPDinterval(border.week1.id$VCV)
```

```
##                                        lower      upper
## (Intercept):(Intercept).picomp  2.551274e-09   6.025875
## obs.cen:(Intercept).picomp     -6.818446e-01   1.863805
```

```
## (Intercept):obs.cen.picomp     -6.818446e-01   1.863805
## obs.cen:obs.cen.picomp          6.780166e-02   1.895296
## units                           2.860069e+02 324.620770
## attr(,"Probability")
## [1] 0.95
```

```
border.rpt.wk1 <- border.week1.id$VCV[, "(Intercept):(Intercept).picomp"]/(border.week1.id$VCV[,
    "(Intercept):(Intercept).picomp"] + border.week1.id$VCV[, "obs.cen:obs.cen.picomp"] +
    border.week1.id$VCV[, "units"])
posterior.mode(border.rpt.wk1)
```

```
##         var1
## 0.0001237781
```

```
HPDinterval(border.rpt.wk1)
```

```
##             lower      upper
## var1 8.776027e-12 0.01941638
## attr(,"Probability")
## [1] 0.95
```

Behavioral correlations

Let's test how correlated these different behaviors are on Day 1 and the entire observation period. Generally, the behaviors are well correlated, especially the ones directly relating to movement.

```
set.seed(410)
behav.day1.cor <- MCMCglmm(cbind(speed.med, propmoving, distmoved.iqr, borderdist.med) ~
    trait - 1, random = ~us(trait):picomp, rcov = ~us(trait):units, family = c(rep("gaussian",
    4)), prior = prior.cov4, nitt = 510000, thin = 200, burnin = 10000, verbose = F,
    data = day1.com)

# Among indv corr on Day 1 ----
behav.day1.among <- matrix(posterior.mode(posterior.cor(behav.day1.cor$VCV[,
    1:16])), 4, 4, dimnames = list(c("Med.speed", "Prop.moving", "IQR.speed",
    "Med.border"), c("Med.speed", "Prop.moving", "IQR.speed", "Med.border")))

# now to extract the CI estimates
ci.day1.among <- data.frame(HPDinterval(posterior.cor(behav.day1.cor$VCV[, 1:16])))

# for corrplot need 3 matrices - estimates, lower CI, upper CI
lower.day1 <- matrix(ci.day1.among[, 1], 4, 4)
upper.day1 <- matrix(ci.day1.among[, 2], 4, 4)

test <- melt(lower.day1) %>%
    mutate(p.value = ifelse(value < 0, 0.99, 0.01)) %>%
    select(Var1, Var2, p.value)

p.mat <- diag(4)
```

```r
p.mat[cbind(test$Var1, test$Var2)] <- p.mat[cbind(test$Var2, test$Var1)] <- test$p.value

colnames(p.mat) <- c("Med.speed", "Prop.moving", "IQR.speed", "Med.border")
row.names(p.mat) <- c("Med.speed", "Prop.moving", "IQR.speed", "Med.border")

corrplot(behav.day1.among, type = "upper", method = "ellipse", p.mat = p.mat,
    insig = "blank")
```

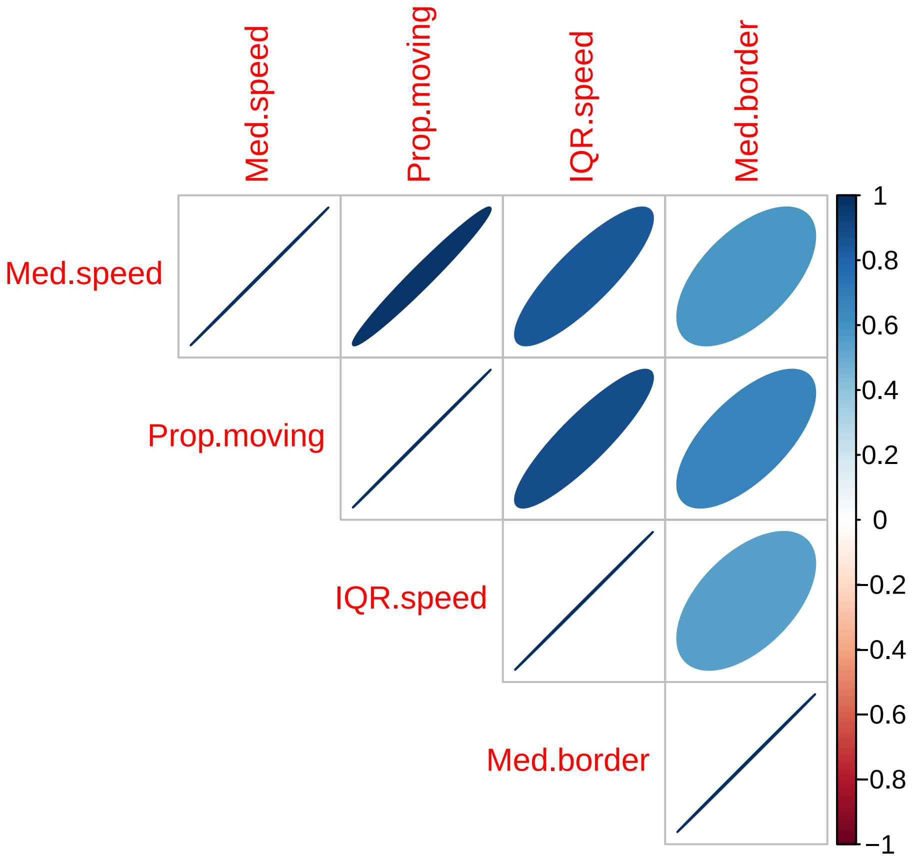

> **Day 1**

```r
set.seed(100)
behav.all.cor <- MCMCglmm(cbind(speed.med, propmoving, distmoved.iqr, borderdist.med) ~
    trait - 1, random = ~us(trait):picomp, rcov = ~us(trait):units, family = c(rep("gaussian",
    4)), prior = prior.cov4, nitt = 510000, thin = 200, burnin = 10000, verbose = F,
    data = indv.com)

# Model for entire observation period ----
behav.matrix.all <- matrix(posterior.mode(posterior.cor(behav.all.cor$VCV[,
    1:16])), 4, 4, dimnames = list(c("Med.speed", "Prop.moving", "IQR.speed",
    "Med.border"), c("Med.speed", "Prop.moving", "IQR.speed", "Med.border")))


# now to extract the CI estimates
ci.all <- data.frame(HPDinterval(posterior.cor(behav.all.cor$VCV[, 1:16])))
```

```r
# for corrplot need 3 matrices - estimates, lower CI, upper CI
lower.all <- matrix(ci.all[, 1], 4, 4)
upper.all <- matrix(ci.all[, 2], 4, 4)

test <- melt(lower.all) %>%
    mutate(p.value = ifelse(value < 0, 1, 0)) %>%
    select(Var1, Var2, p.value)

p.mat <- diag(4)
p.mat[cbind(test$Var1, test$Var2)] <- p.mat[cbind(test$Var2, test$Var1)] <- test$p.value

colnames(p.mat) <- c("Med.speed", "Prop.moving", "IQR.speed", "Med.border")
row.names(p.mat) <- c("Med.speed", "Prop.moving", "IQR.speed", "Med.border")

corrplot(behav.matrix.all, type = "upper", method = "ellipse", p.mat = p.mat,
    insig = "blank")
```

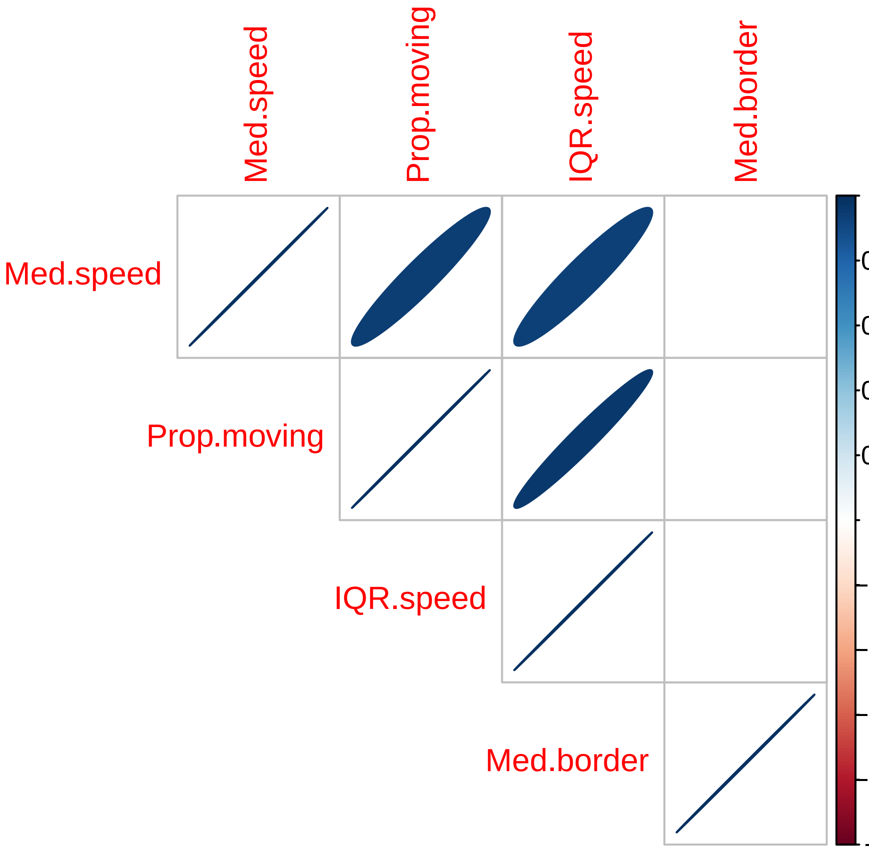

> **Entire observation period**

1a - GROWTH & BODY SIZE

Variation in individual behavior might be related to differences in indivdiual morphology or physiology. We have measurements of key morphological (body size) and physiological (growth rates) traits in these fish so we want to test whether/how much behavioral variation is explained by these other traits.

The key questions we want to ask about body size/growth rate:

1 - does brood size predict body size?

2 - Do individuals consistently differ in their absolute body sizes?

3 - Do individuals consistently differ in their growth rates?

4 - Does variation in body size and/or growth rate explain variation in behavior?

5 - Do the effects of body size and/or growth rate differ AMONG versus WITHIN indivdiuals? This is because some individuals are larger than others (among-individual effects) and then all individuals grow over time (within-individual effects)

The most important thing in running all these models is to look at the marginal/conditional R-squared values. If body size/growth rates *do* explain variation in behavior, then including these as fixed effects in our models should increase the marginal R-squared value considerably (and reduce the relative size of the variance explained by the random effects)

Brood size

No, it does not appear that brood size relates to individual body size at all (which is maybe a bit interesting in itself!)

```
set.seed(290)
size.mcmc <- MCMCglmm(TL ~ brood.size, random = ~mother, data = day1.com, family = "gaussian",
    prior = prior.id, nitt = 510000, burnin = 10000, thin = 200, verbose = F)

summary(size.mcmc)
```

```
##
##  Iterations = 10001:509801
##  Thinning interval  = 200
##  Sample size  = 2500
##
##  DIC: -833.6721
##
##  G-structure:  ~mother
##
##        post.mean l-95% CI u-95% CI eff.samp
## mother    0.0206 0.003241    0.053     2090
##
##  R-structure:  ~units
##
##       post.mean l-95% CI u-95% CI eff.samp
## units  0.003087 0.002578 0.003624     2648
##
##  Location effects: TL ~ brood.size
##
##             post.mean  l-95% CI  u-95% CI eff.samp  pMCMC
## (Intercept)  1.135950  0.905934  1.397128     2500 <4e-04 ***
## brood.size  -0.003524 -0.018051  0.013152     1912  0.612
## ---
## Signif. codes:  0 '***' 0.001 '**' 0.01 '*' 0.05 '.' 0.1 ' ' 1
```

```
posterior.mode(size.mcmc$Sol)
```

```
## (Intercept)   brood.size
## 1.146587480 -0.004380853
```

```
HPDinterval(size.mcmc$Sol)
```

```
##                   lower      upper
## (Intercept)  0.90593401 1.39712829
## brood.size  -0.01805059 0.01315194
## attr(,"Probability")
## [1] 0.95
```

SL repeatabilty

We want to see whether individual's consistently differ in their body size. But we only measured body size once per week, so want to only select one observation per individual each week so that we're not artificially inflating our sample size

ANSWER: Yes, individuals do consistently differ in body size which is not surprising and also evidence that individuals differ in slopes of body size, which we'll investigate further below (by extracting overall growth rates and using that as a predictor)

```
# want to remove multiple observations per week (we only measured TL once
# per week)

indv.week <- indv.com %>%
    group_by(picomp, week) %>%
    arrange(picomp, week) %>%
    slice(1) %>%
    ungroup()


set.seed(156)
tl.mcmc1 <- MCMCglmm(TL ~ obs, random = ~picomp, data = indv.week, family = "gaussian",
    prior = prior.id, nitt = 510000, burnin = 10000, thin = 200, verbose = F)
```

```
## Warning: Unknown or uninitialised column: `family`.
```

```
set.seed(81)
tl.mcmc2 <- MCMCglmm(TL ~ obs, random = ~picomp + mother, data = indv.week,
    family = "gaussian", prior = prior.id.mom, nitt = 510000, burnin = 10000,
    thin = 200, verbose = F)
```

```
## Warning: Unknown or uninitialised column: `family`.
```

```
set.seed(167)
tl.mcmc3 <- MCMCglmm(TL ~ obs, random = ~us(1 + obs):picomp, data = indv.week,
    family = "gaussian", prior = prior.id.slope, nitt = 510000, burnin = 10000,
    thin = 200, verbose = F)
```

```
## Warning: Unknown or uninitialised column: `family`.
```

```
DIC(tl.mcmc1, tl.mcmc2, tl.mcmc3)
```

```
##           df       DIC
## tl.mcmc1  4 -434.2211
## tl.mcmc2  5 -435.2924
## tl.mcmc3  5 -504.5193
```

```
# individual intercepts and slopes best supported

plot(tl.mcmc3$VCV)
```

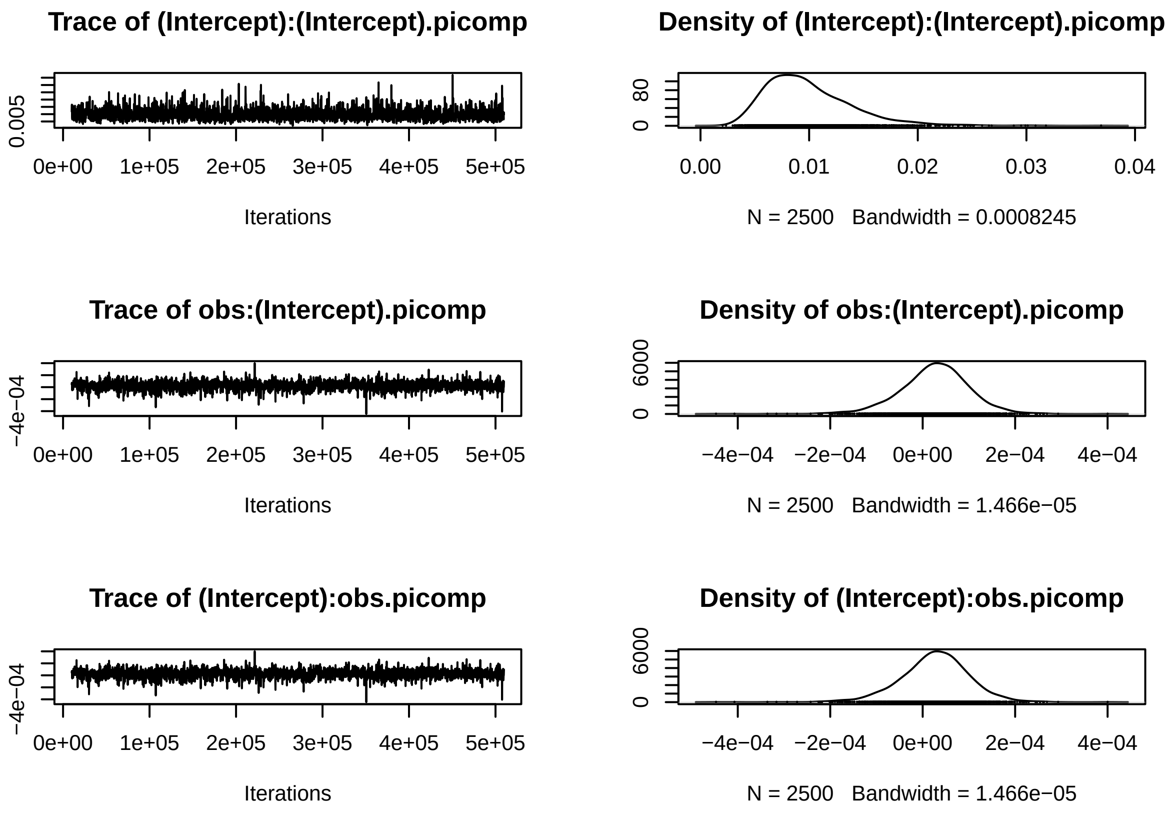

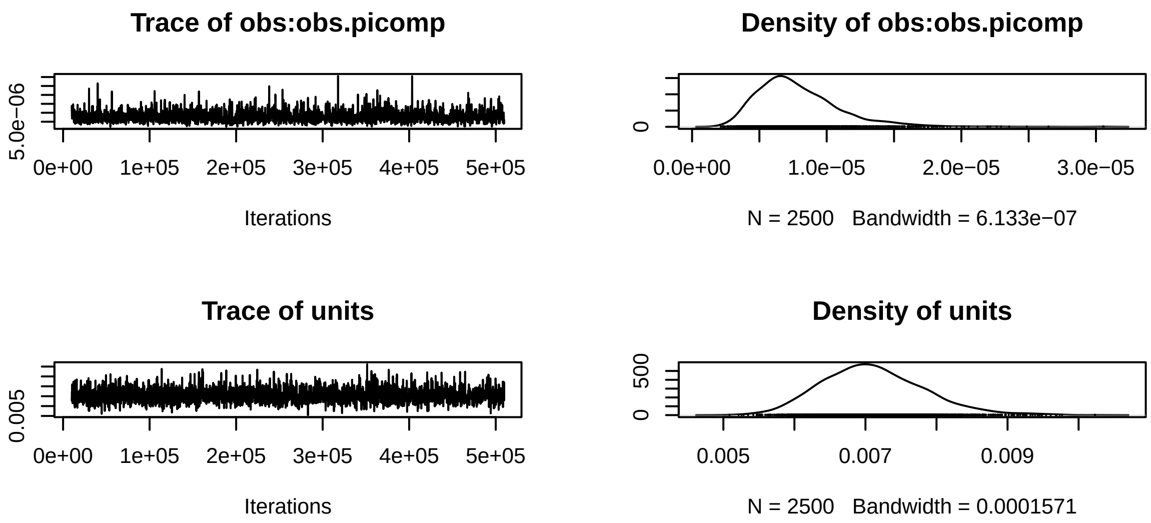


```
summary(tl.mcmc3)
```

```
##
##  Iterations = 10001:509801
##  Thinning interval  = 200
##  Sample size  = 2500
##
##  DIC: -504.5193
##
##  G-structure:  ~us(1 + obs):picomp
##
##                                post.mean   l-95% CI  u-95% CI eff.samp
## (Intercept):(Intercept).picomp 9.928e-03  3.535e-03 1.775e-02     2361
## obs:(Intercept).picomp         2.614e-05 -1.258e-04 1.677e-04     1825
## (Intercept):obs.picomp         2.614e-05 -1.258e-04 1.677e-04     1825
## obs:obs.picomp                 7.895e-06  3.306e-06 1.471e-05     2500
##
##  R-structure:  ~units
##
##       post.mean l-95% CI u-95% CI eff.samp
## units  0.007109 0.005767 0.008531     2500
##
##  Location effects: TL ~ obs
##
##             post.mean l-95% CI u-95% CI eff.samp  pMCMC
```

```
## (Intercept)   1.02924  0.98643  1.07281     2500 <4e-04 ***
## obs           0.02847  0.02733  0.02962     2705 <4e-04 ***
## ---
## Signif. codes:  0 '***' 0.001 '**' 0.01 '*' 0.05 '.' 0.1 ' ' 1
```

```
posterior.mode(tl.mcmc3$VCV)
```

```
## (Intercept):(Intercept).picomp         obs:(Intercept).picomp
##                   9.550397e-03                   1.584207e-05
##         (Intercept):obs.picomp                 obs:obs.picomp
##                   1.584207e-05                   6.308417e-06
##                          units
##                   7.224010e-03
```

```
HPDinterval(tl.mcmc3$VCV)
```

```
##                                        lower        upper
## (Intercept):(Intercept).picomp  3.534949e-03 1.774947e-02
## obs:(Intercept).picomp         -1.257705e-04 1.676562e-04
## (Intercept):obs.picomp         -1.257705e-04 1.676562e-04
## obs:obs.picomp                  3.305722e-06 1.470607e-05
## units                           5.767485e-03 8.530782e-03
## attr(,"Probability")
## [1] 0.95
```

```
rpt.tl <- tl.mcmc3$VCV[, "(Intercept):(Intercept).picomp"]/(tl.mcmc3$VCV[, "(Intercept):(Intercept).pic
    tl.mcmc3$VCV[, "obs:obs.picomp"] + tl.mcmc3$VCV[, "units"])
posterior.mode(rpt.tl)
```

```
##     var1
## 0.566833
```

```
HPDinterval(rpt.tl)
```

```
##          lower     upper
## var1 0.3735629 0.7420184
## attr(,"Probability")
## [1] 0.95
```

growth rate repeatability

Here using weekly growth rate (Week2 - Week1/Week1) as the response variable. our goal now is test whether there is evidence for individual differences in growth rate (i.e. some individuals are consistently growing faster/slower each week compared to others)

ANSWER: No evidence of significant among-individual variation in weekly growth rates. So the above models suggest that some individuals are consistently bigger/smaller than others in absolute terms, but that individuals are not consistently GROWING faster/slower across the weeks.

```r
# all individuals have NA growth rate for first week, so need to remove
set.seed(421)
growth.mcmc1 <- MCMCglmm(growth.rate ~ obs, random = ~picomp, data = indv.week[which(!is.na(indv.week[,
    "growth.rate"])), ], family = "gaussian", prior = prior.id, nitt = 510000,
    burnin = 10000, thin = 200, verbose = F)
```

```
## Warning: Unknown or uninitialised column: ‘family‘.
```

```r
set.seed(606)
growth.mcmc2 <- MCMCglmm(growth.rate ~ obs, random = ~picomp + mother, data = indv.week[which(!is.na(in
    "growth.rate"])), ], family = "gaussian", prior = prior.id.mom, nitt = 510000,
    burnin = 10000, thin = 200, verbose = F)
```

```
## Warning: Unknown or uninitialised column: ‘family‘.
```

```r
set.seed(810)
growth.mcmc3 <- MCMCglmm(growth.rate ~ obs, random = ~us(1 + obs):picomp, data = indv.week[which(!is.na
    "growth.rate"])), ], family = "gaussian", prior = prior.id.slope, nitt = 510000,
    burnin = 10000, thin = 200, verbose = F)
```

```
## Warning: Unknown or uninitialised column: ‘family‘.
```

```r
DIC(growth.mcmc1, growth.mcmc2, growth.mcmc3)
```

```
##              df       DIC
## growth.mcmc1  4 -590.8475
## growth.mcmc2  5 -589.0667
## growth.mcmc3  5 -588.9064
```

```r
# individual intercepts best supported

plot(growth.mcmc1$VCV)
```

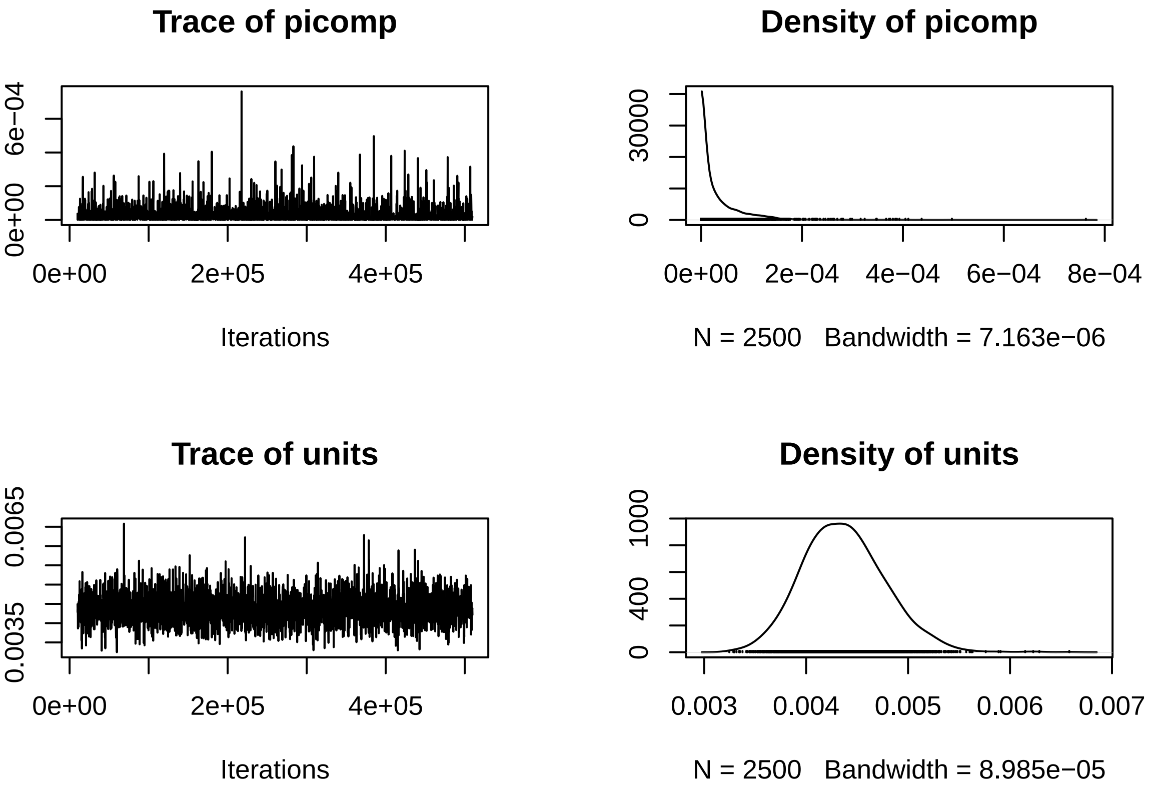


```
summary(growth.mcmc1)
```

```
##
##  Iterations = 10001:509801
##  Thinning interval  = 200
##  Sample size  = 2500
##
##  DIC: -590.8475
##
##  G-structure:  ~picomp
##
##        post.mean  l-95% CI  u-95% CI eff.samp
## picomp 3.566e-05 3.867e-11 0.0001308     2500
##
##  R-structure:  ~units
##
##       post.mean l-95% CI u-95% CI eff.samp
## units  0.004371 0.003628 0.005205     2500
##
##  Location effects: growth.rate ~ obs
##
##             post.mean  l-95% CI  u-95% CI eff.samp  pMCMC
## (Intercept)  0.174759  0.158794  0.190499     2594 <4e-04 ***
## obs         -0.001882 -0.002331 -0.001428     2353 <4e-04 ***
## ---
## Signif. codes:  0 '***' 0.001 '**' 0.01 '*' 0.05 '.' 0.1 ' ' 1
```

```
rpt.gr <- growth.mcmc1$VCV[, "picomp"]/(growth.mcmc1$VCV[, "picomp"] + growth.mcmc1$VCV[,
    "units"])
posterior.mode(rpt.gr)
```

```
##         var1
## 0.0001601799
```

```
HPDinterval(rpt.gr)  # no evidence of repeatability in weekly growth
```

```
##             lower      upper
## var1 8.681155e-09 0.02877301
## attr(,"Probability")
## [1] 0.95
```

However, overall growth rate (over the entire 10 week observation period) may still be informative for behavior so now I want to include the overall slope of an individual's absolute TL over time (from a model that includes random slopes) as this likely our best estimate of an individual's overall (as opposed to weekly) growth rate

So need to re-run random slopes model and extract slopes then use those as predictors of behavior

```
set.seed(167)
tl.mcmc3 <- MCMCglmm(TL ~ obs, random = ~us(1 + obs):picomp, family = "gaussian",
    data = indv.week, prior = prior.id.slope, pr = T, nitt = 510000, burnin = 10000,
    thin = 200, verbose = F)
```

```
## Warning: Unknown or uninitialised column: `family`.
```

```
plot(tl.mcmc3$VCV)
```

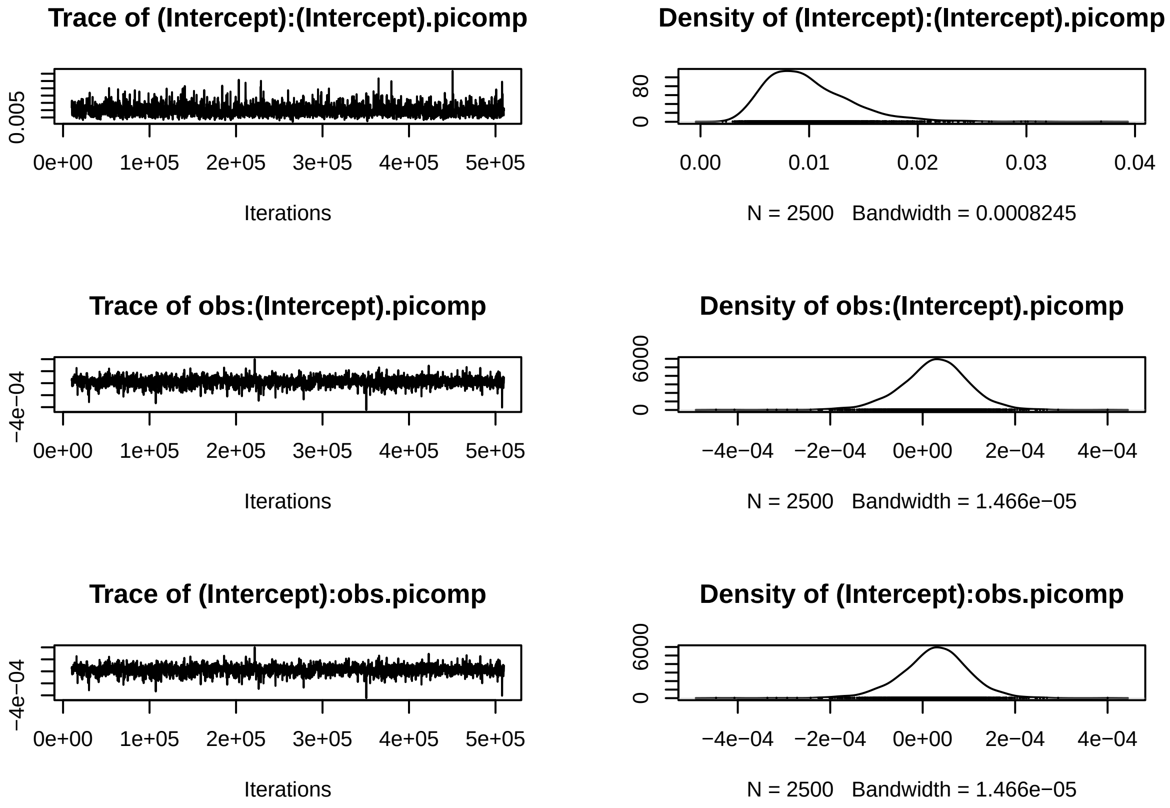
Trace of (Intercept):(Intercept).picomp
0.005
0e+00 1e+05 2e+05 3e+05 4e+05 5e+05
Iterations
Density of (Intercept):(Intercept).picomp
0 80
0.00 0.01 0.02 0.03 0.04
N = 2500 Bandwidth = 0.0008245
Trace of obs:(Intercept).picomp
−4e−04
0e+00 1e+05 2e+05 3e+05 4e+05 5e+05
Iterations
Density of obs:(Intercept).picomp
0 6000
−4e−04 −2e−04 0e+00 2e−04 4e−04
N = 2500 Bandwidth = 1.466e−05
Trace of (Intercept):obs.picomp
−4e−04
0e+00 1e+05 2e+05 3e+05 4e+05 5e+05
Iterations
Density of (Intercept):obs.picomp
0 6000
−4e−04 −2e−04 0e+00 2e−04 4e−04
N = 2500 Bandwidth = 1.466e−05

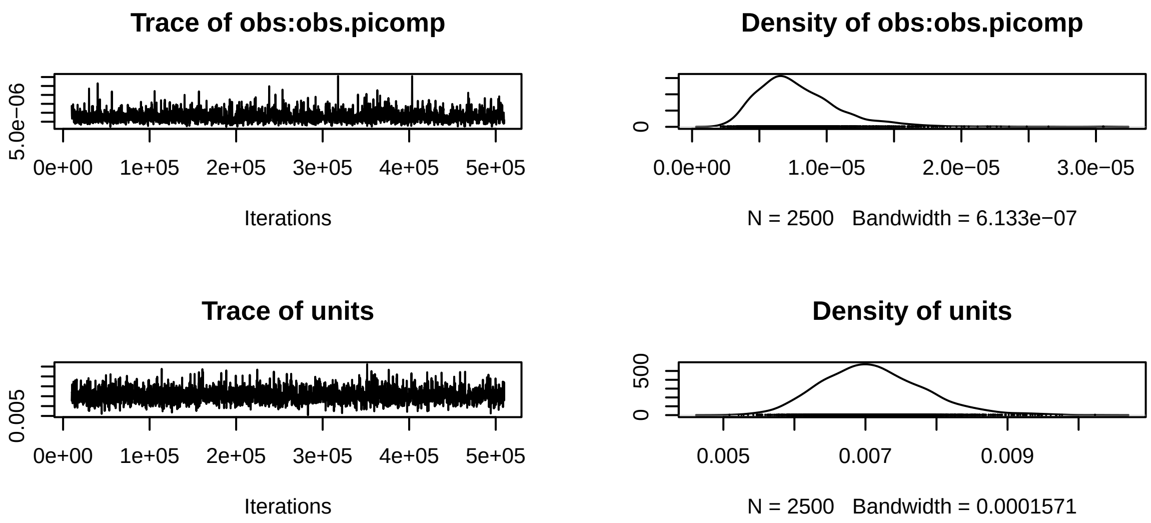


```
summary(tl.mcmc3)
```

```
##
##  Iterations = 10001:509801
##  Thinning interval  = 200
##  Sample size  = 2500
##
##  DIC: -504.5193
##
##  G-structure:  ~us(1 + obs):picomp
##
##                                post.mean   l-95% CI  u-95% CI eff.samp
## (Intercept):(Intercept).picomp 9.928e-03  3.535e-03 1.775e-02     2361
## obs:(Intercept).picomp         2.614e-05 -1.258e-04 1.677e-04     1825
## (Intercept):obs.picomp         2.614e-05 -1.258e-04 1.677e-04     1825
## obs:obs.picomp                 7.895e-06  3.306e-06 1.471e-05     2500
##
##  R-structure:  ~units
##
##       post.mean l-95% CI u-95% CI eff.samp
## units  0.007109 0.005767 0.008531     2500
##
##  Location effects: TL ~ obs
##
##             post.mean l-95% CI u-95% CI eff.samp  pMCMC
```

```
## (Intercept)   1.02924  0.98643  1.07281     2500 <4e-04 ***
## obs           0.02847  0.02733  0.02962     2705 <4e-04 ***
## ---
## Signif. codes:  0 '***' 0.001 '**' 0.01 '*' 0.05 '.' 0.1 ' ' 1
```

```
# extract the estimates for each individual's growth rate
colnames(tl.mcmc3$Sol)
```

```
##  [1] "(Intercept)"                "obs"
##  [3] "(Intercept).picomp.pi21_2" "(Intercept).picomp.pi21_3"
##  [5] "(Intercept).picomp.pi21_4" "(Intercept).picomp.pi22_1"
##  [7] "(Intercept).picomp.pi22_2" "(Intercept).picomp.pi22_3"
##  [9] "(Intercept).picomp.pi22_4" "(Intercept).picomp.pi23_2"
## [11] "(Intercept).picomp.pi23_3" "(Intercept).picomp.pi23_4"
## [13] "(Intercept).picomp.pi24_1" "(Intercept).picomp.pi24_2"
## [15] "(Intercept).picomp.pi24_3" "(Intercept).picomp.pi24_4"
## [17] "(Intercept).picomp.pi43_3" "(Intercept).picomp.pi44_2"
## [19] "(Intercept).picomp.pi44_3" "(Intercept).picomp.pi44_4"
## [21] "(Intercept).picomp.pi61_1" "(Intercept).picomp.pi63_1"
## [23] "(Intercept).picomp.pi63_2" "(Intercept).picomp.pi63_3"
## [25] "(Intercept).picomp.pi63_4" "(Intercept).picomp.pi64_1"
## [27] "(Intercept).picomp.pi64_2" "(Intercept).picomp.pi64_3"
## [29] "obs.picomp.pi21_2"         "obs.picomp.pi21_3"
## [31] "obs.picomp.pi21_4"         "obs.picomp.pi22_1"
## [33] "obs.picomp.pi22_2"         "obs.picomp.pi22_3"
## [35] "obs.picomp.pi22_4"         "obs.picomp.pi23_2"
## [37] "obs.picomp.pi23_3"         "obs.picomp.pi23_4"
## [39] "obs.picomp.pi24_1"         "obs.picomp.pi24_2"
## [41] "obs.picomp.pi24_3"         "obs.picomp.pi24_4"
## [43] "obs.picomp.pi43_3"         "obs.picomp.pi44_2"
## [45] "obs.picomp.pi44_3"         "obs.picomp.pi44_4"
## [47] "obs.picomp.pi61_1"         "obs.picomp.pi63_1"
## [49] "obs.picomp.pi63_2"         "obs.picomp.pi63_3"
## [51] "obs.picomp.pi63_4"         "obs.picomp.pi64_1"
## [53] "obs.picomp.pi64_2"         "obs.picomp.pi64_3"
```

```
colnames(tl.mcmc3$Sol[, 29:54])
```

```
##  [1] "obs.picomp.pi21_2" "obs.picomp.pi21_3" "obs.picomp.pi21_4"
##  [4] "obs.picomp.pi22_1" "obs.picomp.pi22_2" "obs.picomp.pi22_3"
##  [7] "obs.picomp.pi22_4" "obs.picomp.pi23_2" "obs.picomp.pi23_3"
## [10] "obs.picomp.pi23_4" "obs.picomp.pi24_1" "obs.picomp.pi24_2"
## [13] "obs.picomp.pi24_3" "obs.picomp.pi24_4" "obs.picomp.pi43_3"
## [16] "obs.picomp.pi44_2" "obs.picomp.pi44_3" "obs.picomp.pi44_4"
## [19] "obs.picomp.pi61_1" "obs.picomp.pi63_1" "obs.picomp.pi63_2"
## [22] "obs.picomp.pi63_3" "obs.picomp.pi63_4" "obs.picomp.pi64_1"
## [25] "obs.picomp.pi64_2" "obs.picomp.pi64_3"
```

```
slopes <- posterior.mode(tl.mcmc3$Sol[, 29:54])

# slopes is named vector so unname the numbers
gr.slope <- unname(posterior.mode(tl.mcmc3$Sol[, 29:54]))
```

```r
# slopes is a named vector, so just extract the relevant picomp info
picomp <- as.vector(substr(names(slopes), nchar(names(slopes)) - 5, nchar(names(slopes))))

growth.slopes <- data.frame(picomp, gr.slope)

# but also these measures of slopes are DIFFERENCES from the overall
# effect of obs on SL so should include the overall growth slope estimate

growth.slopes <- growth.slopes %>%
    mutate(gr.slope = gr.slope + posterior.mode(tl.mcmc3$Sol[, "obs"]))
```

best predictors?

So now the question is, are weekly body size, weekly growth rate, or overall growth rate (slope) better predictors of behavior? Do they significantly predict (average) behavior? And/or do they account for any of the among-individual variation in behavior?

Here because body size and growth rate could only be measured on a weekly basis, I will summarise the behavioral data on a weekly basis too. I will run some models investigating weekly (absolute) body size and growth rates, but because body size differ both between individuals (some individuals are on average bigger than others) and within individuals (as they grow), I will also compute average TLs and weekly deviation TL (i.e. within-subject centering).

```r
# summarising data for weekly median swimming speed
indv.week <- hourly.com %>%
    group_by(picomp, week) %>%
    mutate(med.speed.wk = median(speed.med, na.rm = T)) %>%
    arrange(picomp, week) %>%
    slice(1)

# add in overall growth slopes from above
indv.week <- left_join(indv.week, growth.slopes)
```

```
## Joining, by = "picomp"
```

```r
# get overall average TL and weekly deviation TL for each individual
indv.week <- indv.week %>%
    group_by(picomp) %>%
    mutate(ave.TL = mean(TL, na.rm = T), dev.TL = TL - ave.TL)

# now because I want to be able to compare all models, I need to use the
# same dataset for each model but because individuals don't have a growth
# rate for week 10, need to remove this from all models

indv.week <- indv.week %>%
    filter(!is.na(growth.rate))


set.seed(472)
speed.growth1 <- MCMCglmm(med.speed.wk ~ week + TL, random = ~us(1 + week):picomp,
    data = indv.week, family = "gaussian", prior = prior.id.slope, nitt = 510000,
    burnin = 10000, thin = 200, verbose = F)
```

```
## Warning: Unknown or uninitialised column: `family`.
```

```r
set.seed(6012)
speed.growth2 <- MCMCglmm(med.speed.wk ~ week + growth.rate, random = ~us(1 +
    week):picomp, data = indv.week, family = "gaussian", prior = prior.id.slope,
    nitt = 510000, burnin = 10000, thin = 200, verbose = F)
```

```
## Warning: Unknown or uninitialised column: `family`.
```

```r
set.seed(841)
speed.growth3 <- MCMCglmm(med.speed.wk ~ week + gr.slope, random = ~us(1 + week):picomp,
    data = indv.week, family = "gaussian", prior = prior.id.slope, nitt = 510000,
    burnin = 10000, thin = 200, verbose = F)
```

```
## Warning: Unknown or uninitialised column: `family`.
```

```r
set.seed(581)
speed.growth4 <- MCMCglmm(med.speed.wk ~ week + TL + growth.rate + gr.slope,
    random = ~us(1 + week):picomp, data = indv.week, family = "gaussian", prior = prior.id.slope,
    nitt = 510000, burnin = 10000, thin = 200, verbose = F)
```

```
## Warning: Unknown or uninitialised column: `family`.
```

```r
set.seed(431)
speed.growth5 <- MCMCglmm(med.speed.wk ~ week + ave.TL + dev.TL, random = ~us(1 +
    week):picomp, data = indv.week, family = "gaussian", prior = prior.id.slope,
    nitt = 510000, burnin = 10000, thin = 200, verbose = F)
```

```
## Warning: Unknown or uninitialised column: `family`.
```

```r
# model comparison
DIC(speed.growth1, speed.growth2, speed.growth3, speed.growth4, speed.growth5)
```

```
##                df      DIC
## speed.growth1  6 288.1301
## speed.growth2  6 288.3878
## speed.growth3  6 286.6803
## speed.growth4  8 290.4587
## speed.growth5  7 288.7900
```

.. report weekly tl

```r
posterior.mode(speed.growth1$Sol)
```

```
## (Intercept)        week          TL
##  0.42159822  0.01906751 -0.01640736
```

```r
HPDinterval(speed.growth1$Sol)
```

```
##                    lower     upper
## (Intercept) -0.005178792 1.1485874
## week        -0.111526665 0.1634813
## TL          -0.750363462 0.5586073
## attr(,"Probability")
## [1] 0.95
```

```r
posterior.mode(speed.growth1$VCV)
```

```
## (Intercept):(Intercept).picomp        week:(Intercept).picomp
##                    0.087360380                    0.011525709
##        (Intercept):week.picomp               week:week.picomp
##                    0.011525709                    0.004011765
##                          units
##                    0.175607278
```

```r
HPDinterval(speed.growth1$VCV)
```

```
##                                       lower       upper
## (Intercept):(Intercept).picomp  0.020500743 0.259243873
## week:(Intercept).picomp        -0.011167323 0.023112739
## (Intercept):week.picomp        -0.011167323 0.023112739
## week:week.picomp                0.001066587 0.009219378
## units                           0.139523507 0.214179715
## attr(,"Probability")
## [1] 0.95
```

```r
vmVarF <- numeric(2500)
for (i in 1:2500) {
    Var <- var(as.vector(speed.growth1$Sol[i, ] %*% t(speed.growth1$X)))
    vmVarF[i] <- Var
}

R2m <- vmVarF/(vmVarF + speed.growth1$VCV[, 1] + speed.growth1$VCV[, 2] + speed.growth1$VCV[,
    4] + speed.growth1$VCV[, 5])
posterior.mode(R2m)
```

```
##         var1
## 0.0008897738
```

```r
HPDinterval(R2m)
```

```
##             lower      upper
## var1 7.703189e-06 0.05729064
## attr(,"Probability")
## [1] 0.95
```

```r
R2c <- (vmVarF + speed.growth1$VCV[, 1])/(vmVarF + speed.growth1$VCV[, 1] +
    speed.growth1$VCV[, 2] + speed.growth1$VCV[, 4] + speed.growth1$VCV[, 5])
posterior.mode(R2c)
```

```
##     var1
## 0.356489
```

```r
HPDinterval(R2c)
```

```
##          lower     upper
## var1 0.1385735 0.6172215
## attr(,"Probability")
## [1] 0.95
```

```r
posterior.mode(speed.growth2$Sol)
```

.. report weekly growth

```
## (Intercept)        week growth.rate
##  0.46303227  0.01186355  0.09277727
```

```r
HPDinterval(speed.growth2$Sol)
```

```
##                   lower      upper
## (Intercept)  0.23336987 0.69475192
## week        -0.02229652 0.05008673
## growth.rate -0.66365475 0.98781449
## attr(,"Probability")
## [1] 0.95
```

```r
posterior.mode(speed.growth2$VCV)
```

```
## (Intercept):(Intercept).picomp        week:(Intercept).picomp
##                    0.105409581                    0.009929603
##        (Intercept):week.picomp               week:week.picomp
##                    0.009929603                    0.003550077
##                          units
##                    0.179588089
```

```r
HPDinterval(speed.growth2$VCV)
```

```
##                                        lower       upper
## (Intercept):(Intercept).picomp  0.0160408061 0.249519969
## week:(Intercept).picomp        -0.0110691713 0.022985571
## (Intercept):week.picomp        -0.0110691713 0.022985571
## week:week.picomp                0.0006867782 0.009490958
## units                           0.1416065910 0.213973098
## attr(,"Probability")
## [1] 0.95
```

```
vmVarF <- numeric(2500)
for (i in 1:2500) {
    Var <- var(as.vector(speed.growth2$Sol[i, ] %*% t(speed.growth2$X)))
    vmVarF[i] <- Var
}

R2m <- vmVarF/(vmVarF + speed.growth2$VCV[, 1] + speed.growth2$VCV[, 2] + speed.growth2$VCV[,
    4] + speed.growth2$VCV[, 5])
posterior.mode(R2m)
```

```
##        var1
## 0.000373817
```

```
HPDinterval(R2m)
```

```
##             lower      upper
## var1 6.210501e-06 0.04124463
## attr(,"Probability")
## [1] 0.95
```

```
R2c <- (vmVarF + speed.growth2$VCV[, 1])/(vmVarF + speed.growth2$VCV[, 1] +
    speed.growth2$VCV[, 2] + speed.growth2$VCV[, 4] + speed.growth2$VCV[, 5])
posterior.mode(R2c)
```

```
##      var1
## 0.3873041
```

```
HPDinterval(R2c)
```

```
##          lower     upper
## var1 0.1410628 0.6251173
## attr(,"Probability")
## [1] 0.95
```

.. report overall growth

```
posterior.mode(speed.growth3$Sol)
```

```
##  (Intercept)         week     gr.slope
## -0.518024504  0.008728433 38.362861806
```

```
HPDinterval(speed.growth3$Sol)
```

```
##                    lower        upper
## (Intercept)  -2.78824833   1.49980243
## week         -0.02322836   0.04661203
## gr.slope    -36.59771515 112.13192753
## attr(,"Probability")
## [1] 0.95
```

```r
posterior.mode(speed.growth3$VCV)
```

```
## (Intercept):(Intercept).picomp        week:(Intercept).picomp
##                     0.085222737                    0.008790850
##        (Intercept):week.picomp               week:week.picomp
##                     0.008790850                    0.002733505
##                           units
##                     0.169891819
```

```r
HPDinterval(speed.growth3$VCV)
```

```
##                                         lower       upper
## (Intercept):(Intercept).picomp  0.0149638269 0.267647426
## week:(Intercept).picomp        -0.0117976289 0.023553264
## (Intercept):week.picomp        -0.0117976289 0.023553264
## week:week.picomp                0.0008971304 0.009416842
## units                           0.1427623216 0.214683533
## attr(,"Probability")
## [1] 0.95
```

```r
vmVarF <- numeric(2500)
for (i in 1:2500) {
    Var <- var(as.vector(speed.growth3$Sol[i, ] %*% t(speed.growth3$X)))
    vmVarF[i] <- Var
}

R2m <- vmVarF/(vmVarF + speed.growth3$VCV[, 1] + speed.growth3$VCV[, 2] + speed.growth3$VCV[,
    4] + speed.growth3$VCV[, 5])
posterior.mode(R2m)
```

```
##        var1
## 0.002153866
```

```r
HPDinterval(R2m)
```

```
##             lower     upper
## var1 4.34236e-05 0.1482263
## attr(,"Probability")
## [1] 0.95
```

```r
R2c <- (vmVarF + speed.growth3$VCV[, 1])/(vmVarF + speed.growth3$VCV[, 1] +
    speed.growth3$VCV[, 2] + speed.growth3$VCV[, 4] + speed.growth3$VCV[, 5])
posterior.mode(R2c)
```

```
##      var1
## 0.3958761
```

```r
HPDinterval(R2c)
```

```
##         lower     upper
## var1 0.149129 0.6454733
## attr(,"Probability")
## [1] 0.95
```

.. report combo

```
posterior.mode(speed.growth4$Sol)
```

```
##  (Intercept)         week           TL  growth.rate     gr.slope 
## -0.596517459  0.001473012  0.028424486  0.078852658 29.479127788
```

```
HPDinterval(speed.growth4$Sol)
```

```
##                   lower       upper
## (Intercept)  -2.8554275   1.8656286
## week         -0.1273565   0.1857909
## TL           -0.8716539   0.7362440
## growth.rate  -0.9804849   1.1215557
## gr.slope    -39.1806938 116.9639961
## attr(,"Probability")
## [1] 0.95
```

```
posterior.mode(speed.growth4$VCV)
```

```
## (Intercept):(Intercept).picomp        week:(Intercept).picomp 
##                    0.103474074                    0.008284844 
##        (Intercept):week.picomp               week:week.picomp 
##                    0.008284844                    0.003119347 
##                          units 
##                    0.175953244
```

```
HPDinterval(speed.growth4$VCV)
```

```
##                                        lower       upper
## (Intercept):(Intercept).picomp  0.0138885962 0.269587702
## week:(Intercept).picomp        -0.0100153390 0.023091893
## (Intercept):week.picomp        -0.0100153390 0.023091893
## week:week.picomp                0.0009120905 0.009470098
## units                           0.1417178851 0.215003857
## attr(,"Probability")
## [1] 0.95
```

```
vmVarF <- numeric(2500)
for (i in 1:2500) {
    Var <- var(as.vector(speed.growth4$Sol[i, ] %*% t(speed.growth4$X)))
    vmVarF[i] <- Var
}

R2m <- vmVarF/(vmVarF + speed.growth4$VCV[, 1] + speed.growth4$VCV[, 2] + speed.growth4$VCV[,
    4] + speed.growth4$VCV[, 5])
posterior.mode(R2m)
```

```
##       var1
## 0.01270488
```

```
HPDinterval(R2m)
```

```
##             lower     upper
## var1 0.0007583652 0.1618964
## attr(,"Probability")
## [1] 0.95
```

```
R2c <- (vmVarF + speed.growth4$VCV[, 1])/(vmVarF + speed.growth4$VCV[, 1] +
    speed.growth4$VCV[, 2] + speed.growth4$VCV[, 4] + speed.growth4$VCV[, 5])
posterior.mode(R2c)
```

```
##      var1
## 0.4232587
```

```
HPDinterval(R2c)
```

```
##          lower     upper
## var1 0.1588218 0.6465723
## attr(,"Probability")
## [1] 0.95
```

Altogether this seems pretty convincing that differences in body size and/or growth rates do not explain much (any?) of the variation in behavior among individuals

.. report within-subj centering

```
posterior.mode(speed.growth5$Sol)
```

```
## (Intercept)        week      ave.TL      dev.TL
##  0.03653551  0.02690495  0.38180968 -0.04634654
```

```
HPDinterval(speed.growth5$Sol)
```

```
##                  lower     upper
## (Intercept) -2.7348971 2.4880130
## week        -0.1016342 0.1871014
## ave.TL      -1.1150315 1.6775429
## dev.TL      -0.8165326 0.5730206
## attr(,"Probability")
## [1] 0.95
```

```
posterior.mode(speed.growth5$VCV)
```

```
## (Intercept):(Intercept).picomp        week:(Intercept).picomp
##                     0.09198658                     0.01113281
##        (Intercept):week.picomp               week:week.picomp
##                     0.01113281                     0.00398096
##                          units
##                     0.17541371
```

```r
HPDinterval(speed.growth5$VCV)
```

```
##                                       lower       upper
## (Intercept):(Intercept).picomp  0.023413309 0.273775620
## week:(Intercept).picomp        -0.008815277 0.026233521
## (Intercept):week.picomp        -0.008815277 0.026233521
## week:week.picomp                0.000900482 0.009493835
## units                           0.139717634 0.213382287
## attr(,"Probability")
## [1] 0.95
```

```r
vmVarF <- numeric(2500)
for (i in 1:2500) {
    Var <- var(as.vector(speed.growth5$Sol[i, ] %*% t(speed.growth5$X)))
    vmVarF[i] <- Var
}

R2m <- vmVarF/(vmVarF + speed.growth5$VCV[, 1] + speed.growth5$VCV[, 2] + speed.growth5$VCV[,
    4] + speed.growth5$VCV[, 5])
posterior.mode(R2m)
```

```
##        var1
## 0.006709825
```

```r
HPDinterval(R2m)
```

```
##             lower     upper
## var1 0.0002367933 0.1156769
## attr(,"Probability")
## [1] 0.95
```

```r
R2c <- (vmVarF + speed.growth5$VCV[, 1])/(vmVarF + speed.growth5$VCV[, 1] +
    speed.growth5$VCV[, 2] + speed.growth5$VCV[, 4] + speed.growth5$VCV[, 5])
posterior.mode(R2c)
```

```
##      var1
## 0.3836049
```

```r
HPDinterval(R2c)
```

```
##          lower     upper
## var1 0.1562043 0.6369589
## attr(,"Probability")
## [1] 0.95
```

So both estimates are negative (as we expect), but neither is significant nor is the marginal R2 very high.

Bottom line: No matter which way we analyze it, variation in morphology (as measured by body size) or physiology (as measured by growth rate) does not seem to explain really any significant variation in behavior among individuals.

2 - CHANGE OVER TIME?

A next major question is whether/how does individual variation change over time? The first thing we will do is to explore which random structure best fits our data as this will then determine how we proceed with the analysis (same process as we did for Day 1 data).

Going to run series of models with different random structures to best characterize the individual variation: 1 - null model, no random effects 2 - ID intercept only 3 - ID + mom intercept only 4 - ID slopes + mom slopes 5 - ID intercepts + slopes only

A. Entire observation period

Now we'll explore the random structure for the behavioral variation taken over the entire 70 day observation period

```
# m0 - null model

set.seed(17)
speed.0 <- MCMCglmm(speed.med ~ obs + TL, data = indv.com, family = "gaussian",
    prior = prior.null, nitt = 510000, burnin = 10000, thin = 200, verbose = F)


# Intercepts ID ----------------------
set.seed(343)
speed.1 <- MCMCglmm(speed.med ~ obs + TL, random = ~picomp, data = indv.com,
    family = "gaussian", prior = prior.id, nitt = 510000, burnin = 10000, thin = 200,
    verbose = F)

# only including intercepts for mother ------
set.seed(1021)
speed.2 <- MCMCglmm(speed.med ~ obs + TL, random = ~mother, data = indv.com,
    family = "gaussian", prior = prior.id, nitt = 510000, burnin = 10000, thin = 200,
    verbose = F)


# Intercepts ID and Mom-----------
set.seed(842)
speed.3 <- MCMCglmm(speed.med ~ obs + TL, random = ~picomp + mother, data = indv.com,
    family = "gaussian", prior = prior.id.mom, nitt = 510000, burnin = 10000,
    thin = 200, verbose = F)


# Intercepts and Slopes ID -----------------------
set.seed(372)
speed.4 <- MCMCglmm(speed.med ~ obs + TL, random = ~us(1 + obs):picomp, data = indv.com,
    family = "gaussian", prior = prior.id.slope, nitt = 510000, burnin = 10000,
    thin = 200, verbose = F)

# Intercepts Mom, Slopes Mom ----------------
set.seed(918)
speed.5 <- MCMCglmm(speed.med ~ obs + TL, random = ~us(1 + obs):mother, data = indv.com,
```

```
    family = "gaussian", prior = prior.id.slope, nitt = 510000, burnin = 10000,
    thin = 200, verbose = F)


# Intercepts ID and Mom, Slopes ID ----------------
set.seed(327)
speed.6 <- MCMCglmm(speed.med ~ obs + TL, random = ~us(1 + obs):picomp + mother,
    data = indv.com, family = "gaussian", prior = prior.id.slope.mom, nitt = 510000,
    burnin = 10000, thin = 200, verbose = F)


# Intercepts ID & Mom, Slopes ID & Mom -----------------
set.seed(178)
speed.7 <- MCMCglmm(speed.med ~ obs + TL, random = ~us(1 + obs):picomp + us(1 +
    obs):mother, data = indv.com, family = "gaussian", prior = prior.id.slope.mom.slope,
    nitt = 510000, burnin = 10000, thin = 200, verbose = F)





DIC(speed.0, speed.1, speed.2, speed.3, speed.4, speed.5, speed.6, speed.7)
```

> **model comparison**

```
##         df      DIC
## speed.0  4 3666.757
## speed.1  5 2473.937
## speed.2  5 2823.293
## speed.3  6 2473.567
## speed.4  6 2313.158
## speed.5  6 2777.040
## speed.6  7 2312.492
## speed.7  8 2312.836
```

> **model to report** Model 4: ID intercepts & slopes by far the most supported model. The fact that there is support for random slopes for individuals indicates that patterns of among/within-individual behavioral variation may be changing over the observation period. We'll explore this in more detail below.

```
summary(speed.4)
```

```
##
##  Iterations = 10001:509801
##  Thinning interval  = 200
##  Sample size  = 2500
##
##  DIC: 2313.158
##
##  G-structure:  ~us(1 + obs):picomp
##
##                                post.mean   l-95% CI  u-95% CI eff.samp
## (Intercept):(Intercept).picomp 1.909e-01  9.176e-02 0.3146493     2342
```

```
## obs:(Intercept).picomp         9.210e-05 -1.592e-03 0.0017690     2500
## (Intercept):obs.picomp         9.210e-05 -1.592e-03 0.0017690     2500
## obs:obs.picomp                 6.655e-05  2.837e-05 0.0001135     2196
##
##  R-structure:  ~units
##
##       post.mean l-95% CI u-95% CI eff.samp
## units    0.2061   0.1926   0.2203     2210
##
##  Location effects: speed.med ~ obs + TL
##
##             post.mean  l-95% CI  u-95% CI eff.samp pMCMC
## (Intercept)  0.402121  0.118973  0.648928     2717 0.004 **
## obs         -0.001849 -0.008221  0.005437     2500 0.599
## TL           0.105937 -0.102487  0.326826     2500 0.327
## ---
## Signif. codes:  0 '***' 0.001 '**' 0.01 '*' 0.05 '.' 0.1 ' ' 1
```

> **model validation** Again, want to validate that this model is not unduly influenced by prior specification and is converging nicely.

Yep, variance component estimates look similar/stable

```
# parameter expanded
prior.id.slope <- list(R = list(V = 1, nu = 0.002), G = list(G1 = list(V = diag(2),
    nu = 0.002, alpha.mu = c(0, 0), alpha.V = diag(2) * 25^2)))


# weak priors (inverse gamma)
prior.weak <- list(R = list(V = 1, nu = 0.002), G = list(G1 = list(V = diag(2),
    nu = 0.002)))

# strong priors
p.var <- var(indv.com$speed.med, na.rm = TRUE)

# setting most variance in among-indv intercepts (and a bit in slopes)
prior.strong.id <- list(R = list(V = matrix(p.var * 0.05), nu = 1), G = list(G1 = list(V = diag(c(0.8 *
    p.var, 0.15 * p.var)), nu = 1)))

# setting most variance in the within-indv residual and very little to
# among individual intercepts & slopes
prior.strong.res <- list(R = list(V = matrix(p.var * 0.95), nu = 1), G = list(G1 = list(V = diag(c(0.02
    p.var, 0.025 * p.var)), nu = 1)))


set.seed(372)
speed.4.param <- MCMCglmm(speed.med ~ obs + TL, random = ~us(1 + obs):picomp,
    data = indv.com, family = "gaussian", prior = prior.id.slope, nitt = 510000,
    burnin = 10000, thin = 200, verbose = F)


set.seed(372)
speed.4.weak <- MCMCglmm(speed.med ~ obs + TL, random = ~us(1 + obs):picomp,
    data = indv.com, family = "gaussian", prior = prior.weak, nitt = 510000,
```

```r
    burnin = 10000, thin = 200, verbose = F)


set.seed(372)
speed.4.strong1 <- MCMCglmm(speed.med ~ obs + TL, random = ~us(1 + obs):picomp,
    data = indv.com, family = "gaussian", prior = prior.strong.id, nitt = 510000,
    burnin = 10000, thin = 200, verbose = F)


set.seed(372)
speed.4.strong2 <- MCMCglmm(speed.med ~ obs + TL, random = ~us(1 + obs):picomp,
    data = indv.com, family = "gaussian", prior = prior.strong.res, nitt = 510000,
    burnin = 10000, thin = 200, verbose = F)

posterior.mode(speed.4.param$VCV)
```

```
## (Intercept):(Intercept).picomp         obs:(Intercept).picomp
##                    1.794592e-01                   7.503409e-05
##         (Intercept):obs.picomp                 obs:obs.picomp
##                    7.503409e-05                   5.498072e-05
##                           units
##                    2.097083e-01
```

```r
posterior.mode(speed.4.weak$VCV)
```

```
## (Intercept):(Intercept).picomp         obs:(Intercept).picomp
##                    0.1542737275                  -0.0003530469
##         (Intercept):obs.picomp                 obs:obs.picomp
##                   -0.0003530469                   0.0001393238
##                           units
##                    0.2044680559
```

```r
posterior.mode(speed.4.strong1$VCV)
```

```
## (Intercept):(Intercept).picomp         obs:(Intercept).picomp
##                     0.155250399                   -0.001171597
##         (Intercept):obs.picomp                 obs:obs.picomp
##                    -0.001171597                    0.002719649
##                           units
##                     0.204527685
```

```r
posterior.mode(speed.4.strong2$VCV)
```

```
## (Intercept):(Intercept).picomp         obs:(Intercept).picomp
##                    0.1379049425                  -0.0005975391
##         (Intercept):obs.picomp                 obs:obs.picomp
##                   -0.0005975391                   0.0005127281
##                           units
##                    0.2046051121
```

```r
# plot(speed.4.param$VCV) plot(speed.4.weak$VCV) plot(speed.4.strong1$VCV)
# plot(speed.4.strong2$VCV)
```

Now let's run 5 indepedent chains with different seeds to make sure convergence is good.

Yep, all the esimates look very similar, think we're good.

```r
set.seed(372)
speed.4a <- MCMCglmm(speed.med ~ obs + TL, random = ~us(1 + obs):picomp, data = indv.com,
    family = "gaussian", prior = prior.id.slope, nitt = 510000, burnin = 10000,
    thin = 200, verbose = F)

set.seed(39)
speed.4b <- MCMCglmm(speed.med ~ obs + TL, random = ~us(1 + obs):picomp, data = indv.com,
    family = "gaussian", prior = prior.id.slope, nitt = 510000, burnin = 10000,
    thin = 200, verbose = F)

set.seed(6471)
speed.4c <- MCMCglmm(speed.med ~ obs + TL, random = ~us(1 + obs):picomp, data = indv.com,
    family = "gaussian", prior = prior.id.slope, nitt = 510000, burnin = 10000,
    thin = 200, verbose = F)

set.seed(880)
speed.4d <- MCMCglmm(speed.med ~ obs + TL, random = ~us(1 + obs):picomp, data = indv.com,
    family = "gaussian", prior = prior.id.slope, nitt = 510000, burnin = 10000,
    thin = 200, verbose = F)

set.seed(10365)
speed.4e <- MCMCglmm(speed.med ~ obs + TL, random = ~us(1 + obs):picomp, data = indv.com,
    family = "gaussian", prior = prior.id.slope, nitt = 510000, burnin = 10000,
    thin = 200, verbose = F)

posterior.mode(speed.4a$VCV)
```

```
## (Intercept):(Intercept).picomp         obs:(Intercept).picomp
##                    1.794592e-01                   7.503409e-05
##         (Intercept):obs.picomp                 obs:obs.picomp
##                    7.503409e-05                   5.498072e-05
##                           units
##                    2.097083e-01
```

```r
posterior.mode(speed.4b$VCV)
```

```
## (Intercept):(Intercept).picomp         obs:(Intercept).picomp
##                    1.628358e-01                   6.807560e-05
##         (Intercept):obs.picomp                 obs:obs.picomp
##                    6.807560e-05                   6.070238e-05
##                           units
##                    2.048489e-01
```

```r
posterior.mode(speed.4c$VCV)
```

```
## (Intercept):(Intercept).picomp         obs:(Intercept).picomp
##                   1.507097e-01                   2.422019e-04
##         (Intercept):obs.picomp                 obs:obs.picomp
##                   2.422019e-04                   5.717399e-05
##                          units
##                   2.045459e-01
```

```r
posterior.mode(speed.4d$VCV)
```

```
## (Intercept):(Intercept).picomp         obs:(Intercept).picomp
##                   0.1514509453                   0.0001065784
##         (Intercept):obs.picomp                 obs:obs.picomp
##                   0.0001065784                   0.0000538965
##                          units
##                   0.2095057122
```

```r
posterior.mode(speed.4e$VCV)
```

```
## (Intercept):(Intercept).picomp         obs:(Intercept).picomp
##                   1.686418e-01                  -3.198890e-05
##         (Intercept):obs.picomp                 obs:obs.picomp
##                  -3.198890e-05                   4.949964e-05
##                          units
##                   2.067191e-01
```

```r
# plot(speed.4a$VCV) plot(speed.4b$VCV) plot(speed.4c$VCV)
# plot(speed.4d$VCV) plot(speed.4e$VCV)
```

B. Slice - change in variance over time

Given that the above model showed that individuals have different slopes in how their behavior changes over time, this means that the amount of among/within-individual variation is likely changing over time.

For Day 1 data (above) we were interested in when individual differences were first apparent and so it made sense to estimate the among-individual variance components on Hour 1 (the first possible time point). Here, we are more interested in how these patterns of individual variation change over time, so we cannot just estimate the among-individual variance once, but rather want to estimate it at multiple time points across the 10-week experiment.

So, now the goal is to estimate the among- and within-individual variance at different points in the experiment - this can be done by centering the observation covariate at different days as the model will estimate the variance in the intercepts (when all covariates are set to zero). So we will "slice" the dataset every week and estimate run a total of 11 models that estiamte the variance at day 1, day 7, day 14 and so on until day 70

```r


# select only those IDs in that vector & only keep up to obs 70 & make obs
# 1 = 0
```

```r
indv.70obs.cen <- indv.com %>%
    mutate(obs0 = obs - 1, obs7 = obs - 7, obs14 = obs - 14, obs21 = obs - 21,
        obs28 = obs - 28, obs35 = obs - 35, obs42 = obs - 42, obs49 = obs -
            49, obs56 = obs - 56, obs63 = obs - 63, obs70 = obs - 70)

set.seed(403)
speed.obs0 <- MCMCglmm(speed.med ~ obs0 + TL, random = ~us(1 + obs0):picomp,
    data = indv.70obs.cen, family = "gaussian", pr = T, prior = prior.id.slope,
    nitt = 310000, burnin = 10000, thin = 200, verbose = F)

set.seed(765)
speed.obs7 <- MCMCglmm(speed.med ~ obs7 + TL, random = ~us(1 + obs7):picomp,
    data = indv.70obs.cen, family = "gaussian", pr = T, prior = prior.id.slope,
    nitt = 310000, burnin = 10000, thin = 200, verbose = F)

set.seed(9)
speed.obs14 <- MCMCglmm(speed.med ~ obs14 + TL, random = ~us(1 + obs14):picomp,
    data = indv.70obs.cen, family = "gaussian", pr = T, prior = prior.id.slope,
    nitt = 310000, burnin = 10000, thin = 200, verbose = F)

set.seed(471)
speed.obs21 <- MCMCglmm(speed.med ~ obs21 + TL, random = ~us(1 + obs21):picomp,
    data = indv.70obs.cen, family = "gaussian", pr = T, prior = prior.id.slope,
    nitt = 310000, burnin = 10000, thin = 200, verbose = F)

set.seed(530)
speed.obs28 <- MCMCglmm(speed.med ~ obs28 + TL, random = ~us(1 + obs28):picomp,
    data = indv.70obs.cen, family = "gaussian", pr = T, prior = prior.id.slope,
    nitt = 310000, burnin = 10000, thin = 200, verbose = F)

set.seed(341)
speed.obs35 <- MCMCglmm(speed.med ~ obs35 + TL, random = ~us(1 + obs35):picomp,
    data = indv.70obs.cen, family = "gaussian", pr = T, prior = prior.id.slope,
    nitt = 310000, burnin = 10000, thin = 200, verbose = F)

set.seed(876)
speed.obs42 <- MCMCglmm(speed.med ~ obs42 + TL, random = ~us(1 + obs42):picomp,
    data = indv.70obs.cen, family = "gaussian", pr = T, prior = prior.id.slope,
    nitt = 310000, burnin = 10000, thin = 200, verbose = F)

set.seed(525)
speed.obs49 <- MCMCglmm(speed.med ~ obs49 + TL, random = ~us(1 + obs49):picomp,
    data = indv.70obs.cen, family = "gaussian", pr = T, prior = prior.id.slope,
    nitt = 310000, burnin = 10000, thin = 200, verbose = F)

set.seed(612)
speed.obs56 <- MCMCglmm(speed.med ~ obs56 + TL, random = ~us(1 + obs56):picomp,
    data = indv.70obs.cen, family = "gaussian", pr = T, prior = prior.id.slope,
    nitt = 310000, burnin = 10000, thin = 200, verbose = F)

set.seed(947)
speed.obs63 <- MCMCglmm(speed.med ~ obs63 + TL, random = ~us(1 + obs63):picomp,
    data = indv.70obs.cen, family = "gaussian", pr = T, prior = prior.id.slope,
```

```
    nitt = 310000, burnin = 10000, thin = 200, verbose = F)

set.seed(301)
speed.obs70 <- MCMCglmm(speed.med ~ obs70 + TL, random = ~us(1 + obs70):picomp,
    data = indv.70obs.cen, family = "gaussian", pr = T, prior = prior.id.slope,
    nitt = 310000, burnin = 10000, thin = 200, verbose = F)



plot(speed.obs0$VCV)
```

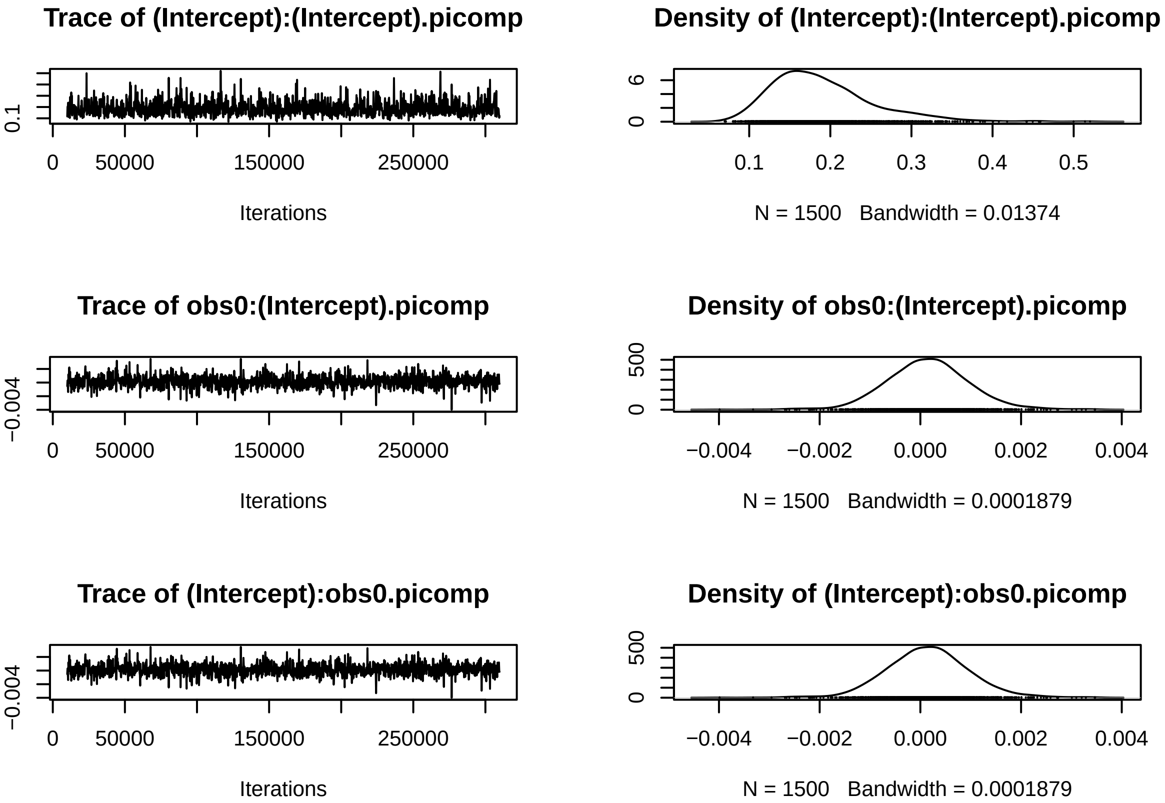
Trace of (Intercept):(Intercept).picomp
0.1
0
50000
150000
250000
Iterations
Density of (Intercept):(Intercept).picomp
6
0
0.1
0.2
0.3
0.4
0.5
N = 1500 Bandwidth = 0.01374
Trace of obs0:(Intercept).picomp
−0.004
0
50000
150000
250000
Iterations
Density of obs0:(Intercept).picomp
500
0
−0.004
−0.002
0.000
0.002
0.004
N = 1500 Bandwidth = 0.0001879
Trace of (Intercept):obs0.picomp
−0.004
0
50000
150000
250000
Iterations
Density of (Intercept):obs0.picomp
500
0
−0.004
−0.002
0.000
0.002
0.004
N = 1500 Bandwidth = 0.0001879

> run models

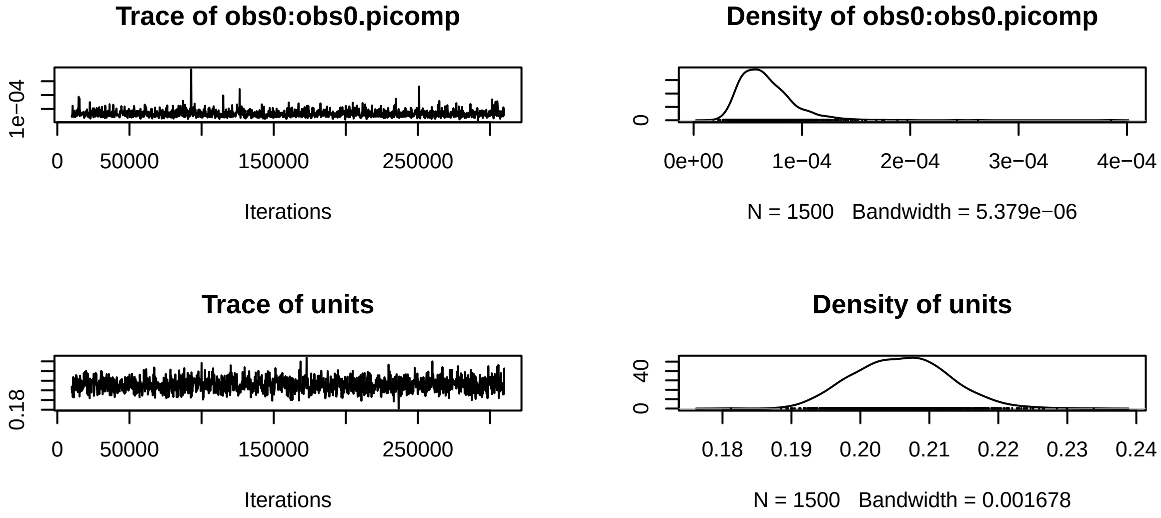
Trace of obs0:obs0.picomp
1e−04
0
50000
150000
250000
Iterations
Density of obs0:obs0.picomp
0
0e+00
1e−04
2e−04
3e−04
4e−04
N = 1500 Bandwidth = 5.379e−06
Trace of units
0.18
0
50000
150000
250000
Iterations
Density of units
40
0
0.18
0.19
0.20
0.21
0.22
0.23
0.24
N = 1500 Bandwidth = 0.001678

> repeatability estimates Here I will extract the variance and repeatability estimates from the models above

```
# now I need to put all these estimates into a vector so I can more easily
# graph them

date <- c(0, 7, 14, 21, 28, 35, 42, 49, 56, 63, 70)

rpt0 <- speed.obs0$VCV[, "(Intercept):(Intercept).picomp"]/(speed.obs0$VCV[,
    "(Intercept):(Intercept).picomp"] + posterior.mode(speed.obs0$VCV[, "units"]))
rpt7 <- speed.obs7$VCV[, "(Intercept):(Intercept).picomp"]/(speed.obs7$VCV[,
    "(Intercept):(Intercept).picomp"] + posterior.mode(speed.obs7$VCV[, "units"]))
rpt14 <- speed.obs14$VCV[, "(Intercept):(Intercept).picomp"]/(speed.obs14$VCV[,
    "(Intercept):(Intercept).picomp"] + posterior.mode(speed.obs14$VCV[, "units"]))
rpt21 <- speed.obs21$VCV[, "(Intercept):(Intercept).picomp"]/(speed.obs21$VCV[,
    "(Intercept):(Intercept).picomp"] + posterior.mode(speed.obs21$VCV[, "units"]))
rpt28 <- speed.obs28$VCV[, "(Intercept):(Intercept).picomp"]/(speed.obs28$VCV[,
    "(Intercept):(Intercept).picomp"] + posterior.mode(speed.obs28$VCV[, "units"]))
rpt35 <- speed.obs35$VCV[, "(Intercept):(Intercept).picomp"]/(speed.obs35$VCV[,
    "(Intercept):(Intercept).picomp"] + posterior.mode(speed.obs35$VCV[, "units"]))
rpt42 <- speed.obs42$VCV[, "(Intercept):(Intercept).picomp"]/(speed.obs42$VCV[,
    "(Intercept):(Intercept).picomp"] + posterior.mode(speed.obs42$VCV[, "units"]))
rpt49 <- speed.obs49$VCV[, "(Intercept):(Intercept).picomp"]/(speed.obs49$VCV[,
    "(Intercept):(Intercept).picomp"] + posterior.mode(speed.obs49$VCV[, "units"]))
rpt56 <- speed.obs56$VCV[, "(Intercept):(Intercept).picomp"]/(speed.obs56$VCV[,
    "(Intercept):(Intercept).picomp"] + posterior.mode(speed.obs56$VCV[, "units"]))
rpt63 <- speed.obs63$VCV[, "(Intercept):(Intercept).picomp"]/(speed.obs63$VCV[,
    "(Intercept):(Intercept).picomp"] + posterior.mode(speed.obs63$VCV[, "units"]))
rpt70 <- speed.obs70$VCV[, "(Intercept):(Intercept).picomp"]/(speed.obs70$VCV[,
    "(Intercept):(Intercept).picomp"] + posterior.mode(speed.obs70$VCV[, "units"]))

rpt <- c(posterior.mode(rpt0), posterior.mode(rpt7), posterior.mode(rpt14),
    posterior.mode(rpt21), posterior.mode(rpt28), posterior.mode(rpt35), posterior.mode(rpt42),
    posterior.mode(rpt49), posterior.mode(rpt56), posterior.mode(rpt63), posterior.mode(rpt70))

ci.rpt <- c(HPDinterval(rpt0)[1:2], HPDinterval(rpt7)[1:2], HPDinterval(rpt14)[1:2],
    HPDinterval(rpt21)[1:2], HPDinterval(rpt28)[1:2], HPDinterval(rpt35)[1:2],
    HPDinterval(rpt42)[1:2], HPDinterval(rpt49)[1:2], HPDinterval(rpt56)[1:2],
    HPDinterval(rpt63)[1:2], HPDinterval(rpt70)[1:2])
ci.rpt <- matrix(ci.rpt, nrow = 11, byrow = T)



post.id <- c(posterior.mode(speed.obs0$VCV[, "(Intercept):(Intercept).picomp"]),
    posterior.mode(speed.obs7$VCV[, "(Intercept):(Intercept).picomp"]), posterior.mode(speed.obs14$VCV[
        "(Intercept):(Intercept).picomp"]), posterior.mode(speed.obs21$VCV[,
        "(Intercept):(Intercept).picomp"]), posterior.mode(speed.obs28$VCV[,
        "(Intercept):(Intercept).picomp"]), posterior.mode(speed.obs35$VCV[,
        "(Intercept):(Intercept).picomp"]), posterior.mode(speed.obs42$VCV[,
        "(Intercept):(Intercept).picomp"]), posterior.mode(speed.obs49$VCV[,
        "(Intercept):(Intercept).picomp"]), posterior.mode(speed.obs56$VCV[,
        "(Intercept):(Intercept).picomp"]), posterior.mode(speed.obs63$VCV[,
        "(Intercept):(Intercept).picomp"]), posterior.mode(speed.obs70$VCV[,
        "(Intercept):(Intercept).picomp"]))
```

```r
ci.id <- c(HPDinterval(speed.obs0$VCV[, "(Intercept):(Intercept).picomp"])[1:2],
    HPDinterval(speed.obs7$VCV[, "(Intercept):(Intercept).picomp"])[1:2], HPDinterval(speed.obs14$VCV[,
        "(Intercept):(Intercept).picomp"])[1:2], HPDinterval(speed.obs21$VCV[,
        "(Intercept):(Intercept).picomp"])[1:2], HPDinterval(speed.obs28$VCV[,
        "(Intercept):(Intercept).picomp"])[1:2], HPDinterval(speed.obs35$VCV[,
        "(Intercept):(Intercept).picomp"])[1:2], HPDinterval(speed.obs42$VCV[,
        "(Intercept):(Intercept).picomp"])[1:2], HPDinterval(speed.obs49$VCV[,
        "(Intercept):(Intercept).picomp"])[1:2], HPDinterval(speed.obs56$VCV[,
        "(Intercept):(Intercept).picomp"])[1:2], HPDinterval(speed.obs63$VCV[,
        "(Intercept):(Intercept).picomp"])[1:2], HPDinterval(speed.obs70$VCV[,
        "(Intercept):(Intercept).picomp"])[1:2])
ci.id <- matrix(ci.id, nrow = 11, byrow = T)

post.w <- c(posterior.mode(speed.obs0$VCV[, "units"]), posterior.mode(speed.obs7$VCV[,
    "units"]), posterior.mode(speed.obs14$VCV[, "units"]), posterior.mode(speed.obs21$VCV[,
    "units"]), posterior.mode(speed.obs28$VCV[, "units"]), posterior.mode(speed.obs35$VCV[,
    "units"]), posterior.mode(speed.obs42$VCV[, "units"]), posterior.mode(speed.obs49$VCV[,
    "units"]), posterior.mode(speed.obs56$VCV[, "units"]), posterior.mode(speed.obs63$VCV[,
    "units"]), posterior.mode(speed.obs70$VCV[, "units"]))

ci.w <- c(HPDinterval(speed.obs0$VCV[, "units"])[1:2], HPDinterval(speed.obs7$VCV[,
    "units"])[1:2], HPDinterval(speed.obs14$VCV[, "units"])[1:2], HPDinterval(speed.obs21$VCV[,
    "units"])[1:2], HPDinterval(speed.obs28$VCV[, "units"])[1:2], HPDinterval(speed.obs35$VCV[,
    "units"])[1:2], HPDinterval(speed.obs42$VCV[, "units"])[1:2], HPDinterval(speed.obs49$VCV[,
    "units"])[1:2], HPDinterval(speed.obs56$VCV[, "units"])[1:2], HPDinterval(speed.obs63$VCV[,
    "units"])[1:2], HPDinterval(speed.obs70$VCV[, "units"])[1:2])
ci.w <- matrix(ci.w, nrow = 11, byrow = T)

plot(post.id ~ date)
```

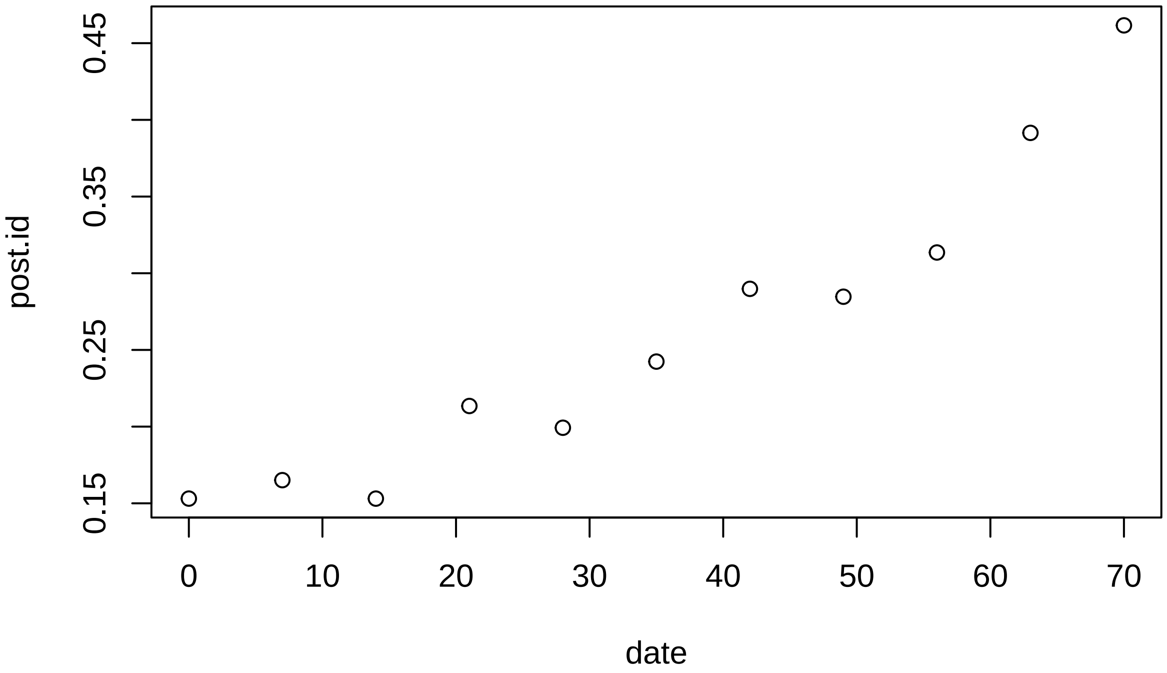


```
plot(post.w ~ date)
```

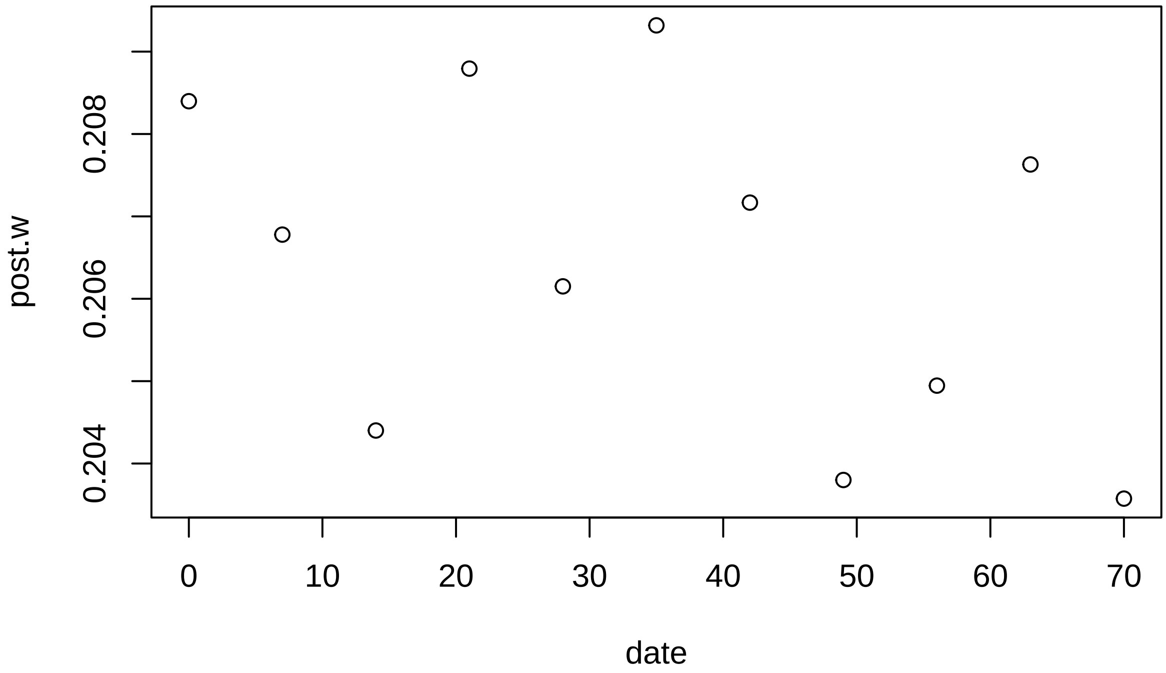


```
plot(rpt ~ date)
```

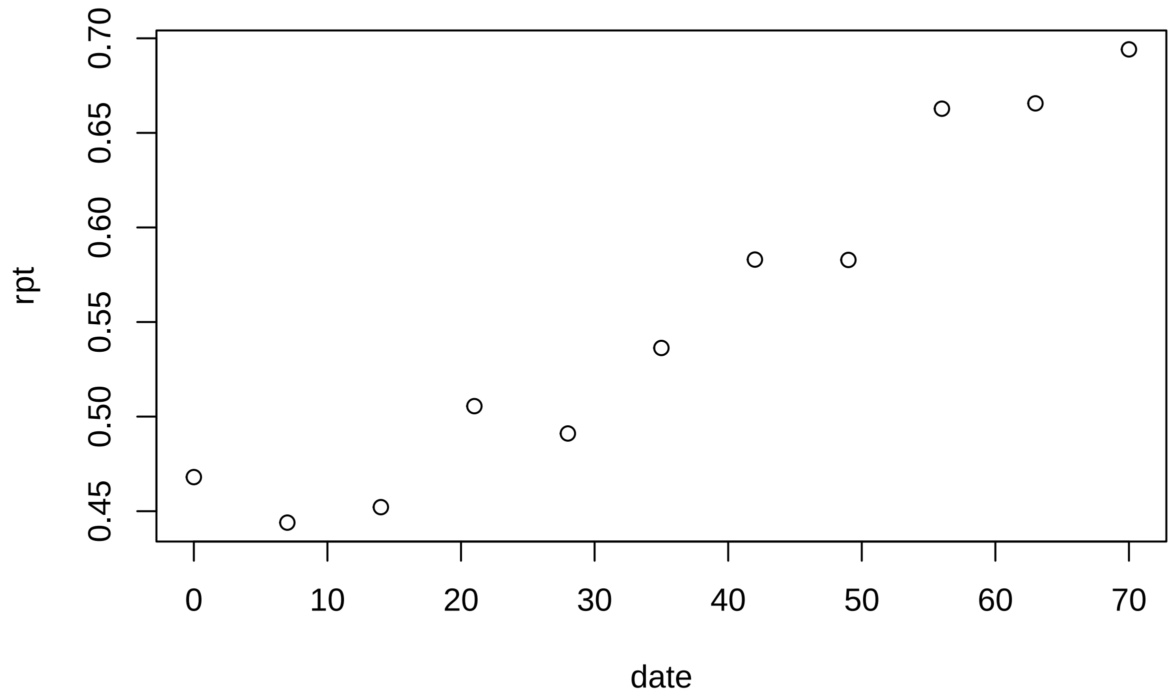


```
rpt.slice.wide <- data.frame(date, rpt, lower.rpt = ci.rpt[, 1], upper.rpt = ci.rpt[,
    2], post.id, lower.id = ci.id[, 1], upper.id = ci.id[, 2], post.w, lower.w = ci.w[,
    1], upper.w = ci.w[, 2])

rpt.slice.long <- data.frame(date = rep(date, 3), type = rep(c("rpt", "id",
    "within"), each = 11), variance = unname(c(rpt, post.id, post.w)), lower = unname(c(ci.rpt[,
    1], ci.id[, 1], ci.w[, 1])), upper = unname(c(ci.rpt[, 2], ci.id[, 2], ci.w[,
    2])))

# write.csv(rpt.slice.long, file = 'repeatability estimates sliced across
# weeks_long_220202.csv')

# write.csv(rpt.slice.wide, file = 'repeatability estimates sliced across
# weeks_wide_220202.csv')
```

> **indv intercepts** Now to visualize these results and see how the patterns of behavioral variation may change over time, I'm going to extract the estimates for the predicted individual intercepts for each time point, which I'll use to graph below in Fig 3

Will need to pull out estimated overall intercept from each model to add to the ID intercepts though (or else they are all difference measures, as opposed to absolute measures). . . . . really need to figure out how to work with named numbers better. . .

```
ids <- colnames(speed.obs0$Sol)[4:29]
ids <- substr(ids, 20, 26)
```

```r
"this pulls out the individual intercepts and adds in the overall intercepts

so that way these numbers are absolute values, as opposed to differences from overall"
```

```
## [1] "this pulls out the individual intercepts and adds in the overall intercepts\n\nso that way these
```

```r
intercepts0 <- unname(posterior.mode(speed.obs0$Sol)[4:29] + posterior.mode(speed.obs0$Sol)["(Intercept
intercepts7 <- unname(posterior.mode(speed.obs7$Sol)[4:29] + posterior.mode(speed.obs7$Sol)["(Intercept
intercepts14 <- unname(posterior.mode(speed.obs14$Sol)[4:29] + posterior.mode(speed.obs14$Sol)["(Interc
intercepts21 <- unname(posterior.mode(speed.obs21$Sol)[4:29] + posterior.mode(speed.obs21$Sol)["(Interc
intercepts28 <- unname(posterior.mode(speed.obs28$Sol)[4:29] + posterior.mode(speed.obs28$Sol)["(Interc
intercepts35 <- unname(posterior.mode(speed.obs35$Sol)[4:29] + posterior.mode(speed.obs35$Sol)["(Interc
intercepts42 <- unname(posterior.mode(speed.obs42$Sol)[4:29] + posterior.mode(speed.obs42$Sol)["(Interc
intercepts49 <- unname(posterior.mode(speed.obs49$Sol)[4:29] + posterior.mode(speed.obs49$Sol)["(Interc
intercepts56 <- unname(posterior.mode(speed.obs56$Sol)[4:29] + posterior.mode(speed.obs56$Sol)["(Interc
intercepts63 <- unname(posterior.mode(speed.obs63$Sol)[4:29] + posterior.mode(speed.obs63$Sol)["(Interc
intercepts70 <- unname(posterior.mode(speed.obs70$Sol)[4:29] + posterior.mode(speed.obs70$Sol)["(Interc


date <- rep(c(0, 7, 14, 21, 28, 35, 42, 49, 56, 63, 70), each = 26)
picomp <- rep(ids, 11)
blup <- c(intercepts0, intercepts7, intercepts14, intercepts21, intercepts28,
    intercepts35, intercepts42, intercepts49, intercepts56, intercepts63, intercepts70)

pred.intercepts <- data.frame(date, picomp, blup)

# write.csv(pred.intercepts, file = 'Predicted individual BLUPs_slice
# model_220202.csv')
```

3 - CORRELATIONS BETWEEN EARLY AND LATER BEHAVIOR?

Given that we know now that individuals are different on Day 1/week 1 of their lives, does this behavior then predict their behavior later in their life?

Here I will run multivariate models that estimate the among-individual correlation in behavior across the 10 weeks of the observation (cannot estimate within-individual correlation as behaviors in week 1 and week x were obviously not expressed at the same time)

```r
"this will spread the data by day, so looking at correlation among 7 days per week"
```

```
## [1] "this will spread the data by day, so looking at correlation among 7 days per week"
```

```r
indv.wide <- indv.com %>%
    mutate(obs.day = case_when(obs %in% seq(1, 70, 7) ~ 1, obs %in% seq(2, 70,
        7) ~ 2, obs %in% seq(3, 70, 7) ~ 3, obs %in% seq(4, 70, 7) ~ 4, obs %in%
        seq(5, 70, 7) ~ 5, obs %in% seq(6, 70, 7) ~ 6, obs %in% seq(7, 70, 7) ~
        7)) %>%
    select(rpi, picomp, week, speed.med, mother, brood.size, birth.date, obs.day) %>%
    spread(week, speed.med, sep = "")

set.seed(2187)
```

```
behav.week.id <- MCMCglmm(cbind(week1, week2, week3, week4, week5, week6, week7,
    week8, week9, week10) ~ trait - 1, random = ~us(trait):picomp, rcov = ~idh(trait):units,
    family = c(rep("gaussian", 10)), prior = prior.cov10, pr = T, nitt = 510000,
    thin = 200, burnin = 10000, verbose = F, data = indv.wide)



# Model with only ID ----
id.matrix.week <- matrix(posterior.mode(posterior.cor(behav.week.id$VCV[, 1:100])),
    10, 10, dimnames = list(c("Week 1", "Week 2", "Week 3", "Week 4", "Week 5",
        "Week 6", "Week 7", "Week 8", "Week 9", "Week 10"), c("Week 1", "Week 2",
        "Week 3", "Week 4", "Week 5", "Week 6", "Week 7", "Week 8", "Week 9",
        "Week 10")))

# now to extract the CI estimates
ci.week <- data.frame(HPDinterval(posterior.cor(behav.week.id$VCV[, 1:100])))

# for corrplot need 3 matrices - estimates, lower CI, upper CI
lower.week <- matrix(ci.week[, 1], 10, 10)
upper.week <- matrix(ci.week[, 2], 10, 10)

test <- melt(lower.week) %>%
    mutate(p.value = ifelse(value < 0, 1, 0)) %>%
    select(Var1, Var2, p.value)

p.mat <- diag(10)
p.mat[cbind(test$Var1, test$Var2)] <- p.mat[cbind(test$Var2, test$Var1)] <- test$p.value


weeks.corr <- ggcorrplot(id.matrix.week, type = "lower", p.mat = p.mat, insig = "blank",
    colors = c("slateblue4", "gray", "mediumorchid1"))
```

> more predictive over time? Something we noticed in the correlation plots is that it looked like the correlation was getting stronger over time, so let's wrangle this data into a dataframe that we can use to test that

```
id.matrix.week
# first pull out among-indv corr
df <- melt(replace(id.matrix.week, lower.tri(id.matrix.week, T), NA), na.rm = T)
str(df)

df$start.week <- as.numeric(substr(df$Var1, 6, 8))
df$end.week <- as.numeric(substr(df$Var2, 6, 8))
df$diff <- df$end.week - df$start.week

# now pull out ci for each corr
lower.week <- matrix(ci.week[, 1], 10, 10)
upper.week <- matrix(ci.week[, 2], 10, 10)

test.lower <- melt(replace(lower.week, lower.tri(lower.week, T), NA), na.rm = T)
test.upper <- melt(replace(upper.week, lower.tri(upper.week, T), NA), na.rm = T)

ci.long <- left_join(test.lower, test.upper, by = c("Var1", "Var2")) %>%
```

```
    rename(start.week = Var1, end.week = Var2, lower = value.x, upper = value.y) %>%
    arrange(start.week, end.week)

among.corr <- left_join(df, ci.long, by = c("start.week", "end.week"))
# write.csv(among.corr, file = 'among individual correlations across weeks
# 220202.csv')
```

```
among.corr <- read.csv("among individual correlations across weeks 220202.csv")


df2 <- among.corr %>%
    filter(diff < 6)

set.seed(421)
corr.mcmc <- MCMCglmm(value ~ start.week * diff, data = df2, family = "gaussian",
    nitt = 510000, burnin = 10000, thin = 200, verbose = F)

summary(corr.mcmc)
```

```
##
##  Iterations = 10001:509801
##  Thinning interval  = 200
##  Sample size  = 2500
##
##  DIC: -64.63589
##
##  R-structure:  ~units
##
##       post.mean l-95% CI u-95% CI eff.samp
## units  0.008345 0.004444  0.01291     2500
##
##  Location effects: value ~ start.week * diff
##
##                  post.mean l-95% CI u-95% CI eff.samp  pMCMC
## (Intercept)        0.76256  0.60655  0.90570     2500 <4e-04 ***
## start.week         0.00252 -0.02565  0.03218     2500 0.8816
## diff              -0.05550 -0.10522 -0.01018     1901 0.0296 *
## start.week:diff    0.01185  0.00014  0.02249     2054 0.0440 *
## ---
## Signif. codes:  0 '***' 0.001 '**' 0.01 '*' 0.05 '.' 0.1 ' ' 1
```

```
# plot(corr.mcmc$Sol)
```

> **extract weekly blups** Now let's extract each individual's predicted behavior for each week - we'll use this to generate the correlation figure (4) below

```
# extract individual BLUPs for each week ----

colnames(behav.week.id$Sol)
```

```
##   [1] "traitweek1"                "traitweek2"
```

```
##   [3] "traitweek3"                 "traitweek4"
##   [5] "traitweek5"                 "traitweek6"
##   [7] "traitweek7"                 "traitweek8"
##   [9] "traitweek9"                 "traitweek10"
##  [11] "traitweek1.picomp.pi21_2"  "traitweek1.picomp.pi21_3"
##  [13] "traitweek1.picomp.pi21_4"  "traitweek1.picomp.pi22_1"
##  [15] "traitweek1.picomp.pi22_2"  "traitweek1.picomp.pi22_3"
##  [17] "traitweek1.picomp.pi22_4"  "traitweek1.picomp.pi23_2"
##  [19] "traitweek1.picomp.pi23_3"  "traitweek1.picomp.pi23_4"
##  [21] "traitweek1.picomp.pi24_1"  "traitweek1.picomp.pi24_2"
##  [23] "traitweek1.picomp.pi24_3"  "traitweek1.picomp.pi24_4"
##  [25] "traitweek1.picomp.pi43_3"  "traitweek1.picomp.pi44_2"
##  [27] "traitweek1.picomp.pi44_3"  "traitweek1.picomp.pi44_4"
##  [29] "traitweek1.picomp.pi61_1"  "traitweek1.picomp.pi63_1"
##  [31] "traitweek1.picomp.pi63_2"  "traitweek1.picomp.pi63_3"
##  [33] "traitweek1.picomp.pi63_4"  "traitweek1.picomp.pi64_1"
##  [35] "traitweek1.picomp.pi64_2"  "traitweek1.picomp.pi64_3"
##  [37] "traitweek2.picomp.pi21_2"  "traitweek2.picomp.pi21_3"
##  [39] "traitweek2.picomp.pi21_4"  "traitweek2.picomp.pi22_1"
##  [41] "traitweek2.picomp.pi22_2"  "traitweek2.picomp.pi22_3"
##  [43] "traitweek2.picomp.pi22_4"  "traitweek2.picomp.pi23_2"
##  [45] "traitweek2.picomp.pi23_3"  "traitweek2.picomp.pi23_4"
##  [47] "traitweek2.picomp.pi24_1"  "traitweek2.picomp.pi24_2"
##  [49] "traitweek2.picomp.pi24_3"  "traitweek2.picomp.pi24_4"
##  [51] "traitweek2.picomp.pi43_3"  "traitweek2.picomp.pi44_2"
##  [53] "traitweek2.picomp.pi44_3"  "traitweek2.picomp.pi44_4"
##  [55] "traitweek2.picomp.pi61_1"  "traitweek2.picomp.pi63_1"
##  [57] "traitweek2.picomp.pi63_2"  "traitweek2.picomp.pi63_3"
##  [59] "traitweek2.picomp.pi63_4"  "traitweek2.picomp.pi64_1"
##  [61] "traitweek2.picomp.pi64_2"  "traitweek2.picomp.pi64_3"
##  [63] "traitweek3.picomp.pi21_2"  "traitweek3.picomp.pi21_3"
##  [65] "traitweek3.picomp.pi21_4"  "traitweek3.picomp.pi22_1"
##  [67] "traitweek3.picomp.pi22_2"  "traitweek3.picomp.pi22_3"
##  [69] "traitweek3.picomp.pi22_4"  "traitweek3.picomp.pi23_2"
##  [71] "traitweek3.picomp.pi23_3"  "traitweek3.picomp.pi23_4"
##  [73] "traitweek3.picomp.pi24_1"  "traitweek3.picomp.pi24_2"
##  [75] "traitweek3.picomp.pi24_3"  "traitweek3.picomp.pi24_4"
##  [77] "traitweek3.picomp.pi43_3"  "traitweek3.picomp.pi44_2"
##  [79] "traitweek3.picomp.pi44_3"  "traitweek3.picomp.pi44_4"
##  [81] "traitweek3.picomp.pi61_1"  "traitweek3.picomp.pi63_1"
##  [83] "traitweek3.picomp.pi63_2"  "traitweek3.picomp.pi63_3"
##  [85] "traitweek3.picomp.pi63_4"  "traitweek3.picomp.pi64_1"
##  [87] "traitweek3.picomp.pi64_2"  "traitweek3.picomp.pi64_3"
##  [89] "traitweek4.picomp.pi21_2"  "traitweek4.picomp.pi21_3"
##  [91] "traitweek4.picomp.pi21_4"  "traitweek4.picomp.pi22_1"
##  [93] "traitweek4.picomp.pi22_2"  "traitweek4.picomp.pi22_3"
##  [95] "traitweek4.picomp.pi22_4"  "traitweek4.picomp.pi23_2"
##  [97] "traitweek4.picomp.pi23_3"  "traitweek4.picomp.pi23_4"
##  [99] "traitweek4.picomp.pi24_1"  "traitweek4.picomp.pi24_2"
## [101] "traitweek4.picomp.pi24_3"  "traitweek4.picomp.pi24_4"
## [103] "traitweek4.picomp.pi43_3"  "traitweek4.picomp.pi44_2"
## [105] "traitweek4.picomp.pi44_3"  "traitweek4.picomp.pi44_4"
## [107] "traitweek4.picomp.pi61_1"  "traitweek4.picomp.pi63_1"
## [109] "traitweek4.picomp.pi63_2"  "traitweek4.picomp.pi63_3"
```

```
## [111] "traitweek4.picomp.pi63_4"  "traitweek4.picomp.pi64_1"
## [113] "traitweek4.picomp.pi64_2"  "traitweek4.picomp.pi64_3"
## [115] "traitweek5.picomp.pi21_2"  "traitweek5.picomp.pi21_3"
## [117] "traitweek5.picomp.pi21_4"  "traitweek5.picomp.pi22_1"
## [119] "traitweek5.picomp.pi22_2"  "traitweek5.picomp.pi22_3"
## [121] "traitweek5.picomp.pi22_4"  "traitweek5.picomp.pi23_2"
## [123] "traitweek5.picomp.pi23_3"  "traitweek5.picomp.pi23_4"
## [125] "traitweek5.picomp.pi24_1"  "traitweek5.picomp.pi24_2"
## [127] "traitweek5.picomp.pi24_3"  "traitweek5.picomp.pi24_4"
## [129] "traitweek5.picomp.pi43_3"  "traitweek5.picomp.pi44_2"
## [131] "traitweek5.picomp.pi44_3"  "traitweek5.picomp.pi44_4"
## [133] "traitweek5.picomp.pi61_1"  "traitweek5.picomp.pi63_1"
## [135] "traitweek5.picomp.pi63_2"  "traitweek5.picomp.pi63_3"
## [137] "traitweek5.picomp.pi63_4"  "traitweek5.picomp.pi64_1"
## [139] "traitweek5.picomp.pi64_2"  "traitweek5.picomp.pi64_3"
## [141] "traitweek6.picomp.pi21_2"  "traitweek6.picomp.pi21_3"
## [143] "traitweek6.picomp.pi21_4"  "traitweek6.picomp.pi22_1"
## [145] "traitweek6.picomp.pi22_2"  "traitweek6.picomp.pi22_3"
## [147] "traitweek6.picomp.pi22_4"  "traitweek6.picomp.pi23_2"
## [149] "traitweek6.picomp.pi23_3"  "traitweek6.picomp.pi23_4"
## [151] "traitweek6.picomp.pi24_1"  "traitweek6.picomp.pi24_2"
## [153] "traitweek6.picomp.pi24_3"  "traitweek6.picomp.pi24_4"
## [155] "traitweek6.picomp.pi43_3"  "traitweek6.picomp.pi44_2"
## [157] "traitweek6.picomp.pi44_3"  "traitweek6.picomp.pi44_4"
## [159] "traitweek6.picomp.pi61_1"  "traitweek6.picomp.pi63_1"
## [161] "traitweek6.picomp.pi63_2"  "traitweek6.picomp.pi63_3"
## [163] "traitweek6.picomp.pi63_4"  "traitweek6.picomp.pi64_1"
## [165] "traitweek6.picomp.pi64_2"  "traitweek6.picomp.pi64_3"
## [167] "traitweek7.picomp.pi21_2"  "traitweek7.picomp.pi21_3"
## [169] "traitweek7.picomp.pi21_4"  "traitweek7.picomp.pi22_1"
## [171] "traitweek7.picomp.pi22_2"  "traitweek7.picomp.pi22_3"
## [173] "traitweek7.picomp.pi22_4"  "traitweek7.picomp.pi23_2"
## [175] "traitweek7.picomp.pi23_3"  "traitweek7.picomp.pi23_4"
## [177] "traitweek7.picomp.pi24_1"  "traitweek7.picomp.pi24_2"
## [179] "traitweek7.picomp.pi24_3"  "traitweek7.picomp.pi24_4"
## [181] "traitweek7.picomp.pi43_3"  "traitweek7.picomp.pi44_2"
## [183] "traitweek7.picomp.pi44_3"  "traitweek7.picomp.pi44_4"
## [185] "traitweek7.picomp.pi61_1"  "traitweek7.picomp.pi63_1"
## [187] "traitweek7.picomp.pi63_2"  "traitweek7.picomp.pi63_3"
## [189] "traitweek7.picomp.pi63_4"  "traitweek7.picomp.pi64_1"
## [191] "traitweek7.picomp.pi64_2"  "traitweek7.picomp.pi64_3"
## [193] "traitweek8.picomp.pi21_2"  "traitweek8.picomp.pi21_3"
## [195] "traitweek8.picomp.pi21_4"  "traitweek8.picomp.pi22_1"
## [197] "traitweek8.picomp.pi22_2"  "traitweek8.picomp.pi22_3"
## [199] "traitweek8.picomp.pi22_4"  "traitweek8.picomp.pi23_2"
## [201] "traitweek8.picomp.pi23_3"  "traitweek8.picomp.pi23_4"
## [203] "traitweek8.picomp.pi24_1"  "traitweek8.picomp.pi24_2"
## [205] "traitweek8.picomp.pi24_3"  "traitweek8.picomp.pi24_4"
## [207] "traitweek8.picomp.pi43_3"  "traitweek8.picomp.pi44_2"
## [209] "traitweek8.picomp.pi44_3"  "traitweek8.picomp.pi44_4"
## [211] "traitweek8.picomp.pi61_1"  "traitweek8.picomp.pi63_1"
## [213] "traitweek8.picomp.pi63_2"  "traitweek8.picomp.pi63_3"
## [215] "traitweek8.picomp.pi63_4"  "traitweek8.picomp.pi64_1"
## [217] "traitweek8.picomp.pi64_2"  "traitweek8.picomp.pi64_3"
```

```
## [219] "traitweek9.picomp.pi21_2"  "traitweek9.picomp.pi21_3"
## [221] "traitweek9.picomp.pi21_4"  "traitweek9.picomp.pi22_1"
## [223] "traitweek9.picomp.pi22_2"  "traitweek9.picomp.pi22_3"
## [225] "traitweek9.picomp.pi22_4"  "traitweek9.picomp.pi23_2"
## [227] "traitweek9.picomp.pi23_3"  "traitweek9.picomp.pi23_4"
## [229] "traitweek9.picomp.pi24_1"  "traitweek9.picomp.pi24_2"
## [231] "traitweek9.picomp.pi24_3"  "traitweek9.picomp.pi24_4"
## [233] "traitweek9.picomp.pi43_3"  "traitweek9.picomp.pi44_2"
## [235] "traitweek9.picomp.pi44_3"  "traitweek9.picomp.pi44_4"
## [237] "traitweek9.picomp.pi61_1"  "traitweek9.picomp.pi63_1"
## [239] "traitweek9.picomp.pi63_2"  "traitweek9.picomp.pi63_3"
## [241] "traitweek9.picomp.pi63_4"  "traitweek9.picomp.pi64_1"
## [243] "traitweek9.picomp.pi64_2"  "traitweek9.picomp.pi64_3"
## [245] "traitweek10.picomp.pi21_2" "traitweek10.picomp.pi21_3"
## [247] "traitweek10.picomp.pi21_4" "traitweek10.picomp.pi22_1"
## [249] "traitweek10.picomp.pi22_2" "traitweek10.picomp.pi22_3"
## [251] "traitweek10.picomp.pi22_4" "traitweek10.picomp.pi23_2"
## [253] "traitweek10.picomp.pi23_3" "traitweek10.picomp.pi23_4"
## [255] "traitweek10.picomp.pi24_1" "traitweek10.picomp.pi24_2"
## [257] "traitweek10.picomp.pi24_3" "traitweek10.picomp.pi24_4"
## [259] "traitweek10.picomp.pi43_3" "traitweek10.picomp.pi44_2"
## [261] "traitweek10.picomp.pi44_3" "traitweek10.picomp.pi44_4"
## [263] "traitweek10.picomp.pi61_1" "traitweek10.picomp.pi63_1"
## [265] "traitweek10.picomp.pi63_2" "traitweek10.picomp.pi63_3"
## [267] "traitweek10.picomp.pi63_4" "traitweek10.picomp.pi64_1"
## [269] "traitweek10.picomp.pi64_2" "traitweek10.picomp.pi64_3"
```

```
# there must be a more elegant way to separate out the columns but
# whatever, it works

weekly.blups <- data_frame(Trait = colnames(behav.week.id$Sol)[11:270], Value = unname(posterior.mode(be
    separate(Trait, into = c("week", "holder", "pi", "comp")) %>%
    mutate(picomp = paste(pi, comp, sep = "_"), week = substr(week, nchar(week) -
        4, nchar(week))) %>%
    select(-holder, -pi, -comp) %>%
    spread(week, Value) %>%
    rename(week10 = eek10)
```

```
## Warning: `data_frame()` was deprecated in tibble 1.1.0.
## Please use `tibble()` instead.
## This warning is displayed once every 8 hours.
## Call `lifecycle::last_lifecycle_warnings()` to see where this warning was generated.
```

```
plot(week5 ~ week4, data = weekly.blups)
```

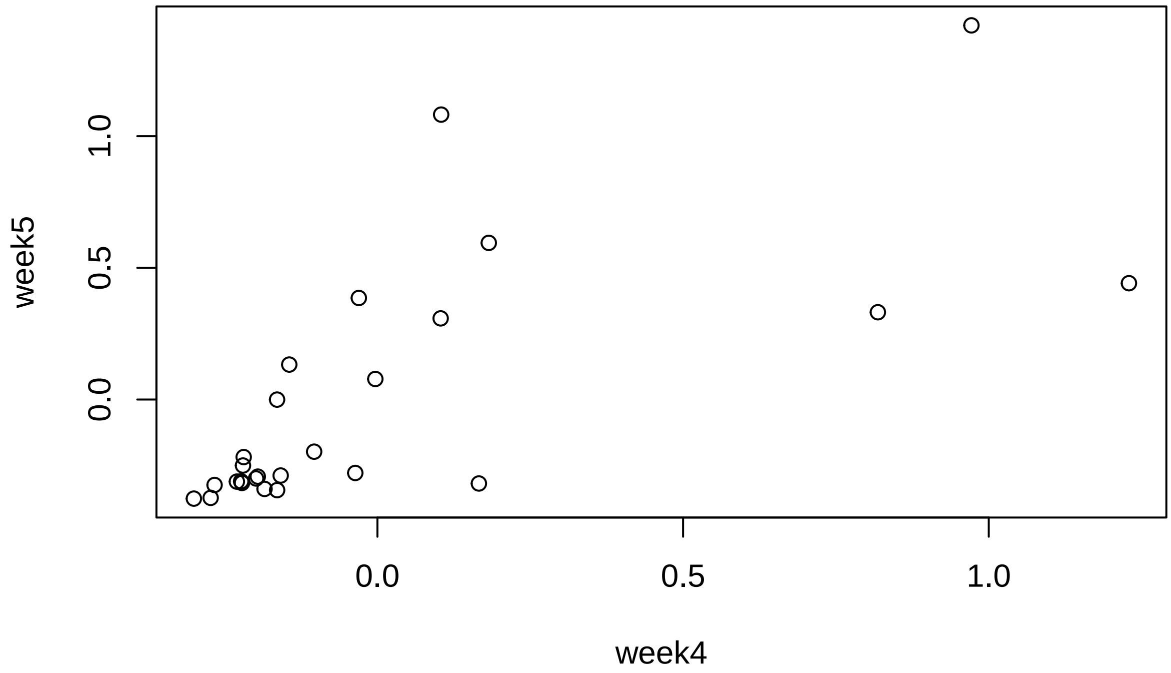


```
plot(week5 ~ week2, data = weekly.blups)
```

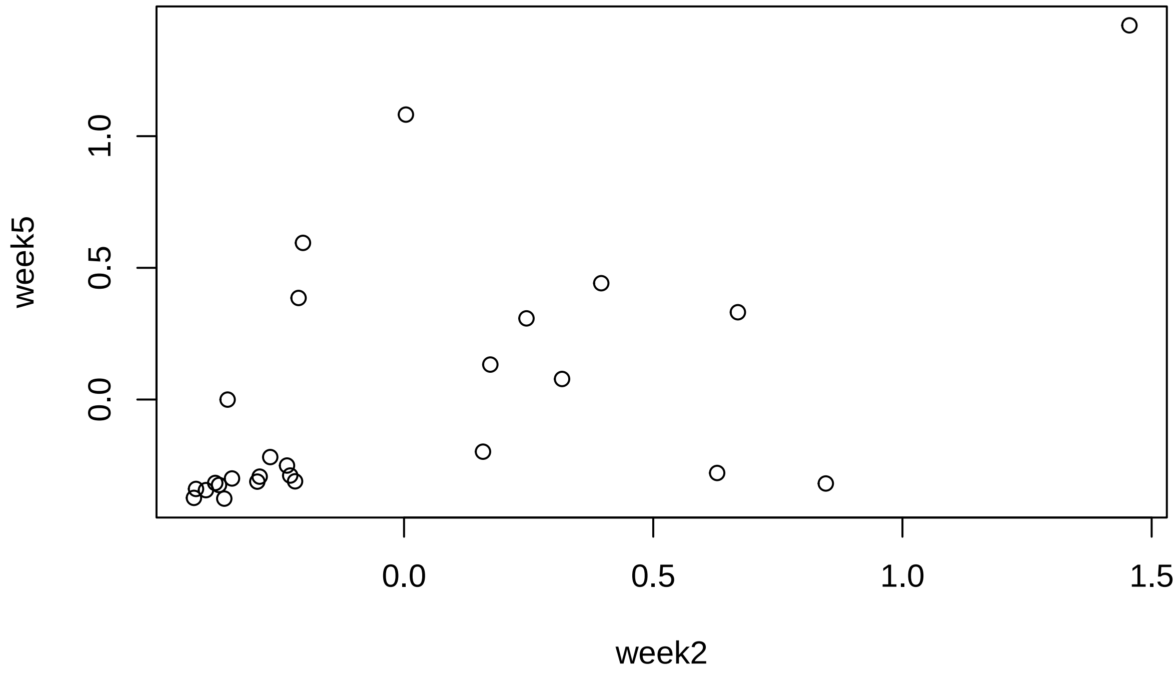


```
# write.csv(weekly.blups, file = 'weekly activity blups across weeks
# 220202.csv')
```

4 - FIGURES

Fig 1 - predictions

This is meant to show 3 potential predictions for how behavioral individuality should develop a - present at birth b - slowly emerge over time c - punctuated emergence at key time points

```
id <- rep(1:6, each = 2)
obs <- rep(c(1, 10), times = 6)
behav <- rep(c(0.5, 1.5, 2.5, 3.5, 4.5, 5.5), each = 2)
flat <- data.frame(factor(id), obs, behav)

behav2 <- c(3, 0.5, 3, 1.5, 3, 2.5, 3, 3.5, 3, 4.5, 3, 5.5)
slow <- data.frame(factor(id), obs, behav2)

id3 <- rep(1:6, each = 4)
obs3 <- rep(c(1, 5, 5.2, 10), times = 6)
behav3 <- c(2.7, 2.7, 0.5, 0.5, 2.8, 2.8, 1.5, 1.5, 2.9, 2.9, 2.5, 2.5, 3, 3,
    3.5, 3.5, 3.1, 3.1, 4.5, 4.5, 3.2, 3.2, 5.5, 5.5)
punct <- data.frame(factor(id3), obs3, behav3)

colors <- c("#0D0887FF", "#6001A6FF", "#A01A9CFF", "#CF4C74FF", "#EB7556FF",
```

```
    "#FDB130FF")

flat.plot <- ggplot(flat, aes(x = obs, y = behav, color = factor(id))) + geom_line(size = 2) +
    xlab("Time") + ylab("Behavior") + ylim(0, 6) + scale_x_continuous(breaks = c(0,
    1, 2, 3, 4, 5, 6, 7, 8, 9, 10)) + scale_color_manual(values = colors) +
    theme_classic() + theme(axis.text = element_blank(), axis.title = element_text(size = 16),
    axis.ticks = element_blank(), legend.position = "none") + annotate("text",
    label = "A", x = 1, y = 6, size = 6)

slow.plot <- ggplot(slow, aes(x = obs, y = behav2, color = factor(id))) + geom_line(size = 2) +
    xlab("Time") + ylab("") + ylim(0, 6) + scale_x_continuous(breaks = c(0,
    1, 2, 3, 4, 5, 6, 7, 8, 9, 10)) + scale_color_manual(values = colors) +
    theme_classic() + theme(axis.text = element_blank(), axis.title = element_text(size = 16),
    axis.ticks = element_blank(), legend.position = "none") + annotate("text",
    label = "B", x = 1, y = 6, size = 6)


punct.plot <- ggplot(punct, aes(x = obs3, y = behav3, color = factor(id3))) +
    geom_line(size = 2) + xlab("Time") + ylab("") + ylim(0, 6) + scale_x_continuous(breaks = c(0,
    1, 2, 3, 4, 5, 6, 7, 8, 9, 10)) + scale_color_manual(values = colors) +
    theme_classic() + theme(axis.text = element_blank(), axis.title = element_text(size = 16),
    axis.ticks = element_blank(), legend.position = "none") + annotate("text",
    label = "C", x = 1, y = 6, size = 6)


# fig1 <- grid.arrange(flat.plot, slow.plot, punct.plot, ncol = 3)

# ggsave(file = 'Fig 1_predictions_220209.tiff', plot = fig1, height = 4,
# width = 12, dpi = 300)
```

Fig 2 - Day 1 results

Figure 2 shows the behavioral reaction norms on the very first day of life along with how this behavior is related to maternal identity and body size.

```
# want to use viridis color palette but don't like the really bright
# yellow so will cut that out
plasma_pal <- viridis::plasma(n = 30)
plasma_pal <- plasma_pal[1:26]

# scales::show_col(plasma_pal)

# want to color the lines according to their behavior in hour 1
day1.rank <- day1.com %>%
    filter(hour == 1) %>%
    mutate(ranking = rank(speed.med, ties.method = "first")) %>%
    select(picomp, ranking)

day1.test <- left_join(day1.com, day1.rank, by = "picomp") %>%
    arrange(ranking) %>%
    group_by(picomp) %>%
    mutate(day1.speed = median(speed.med))
```

```
# day 1 behavior only ----
day1.plot <- ggplot(day1.test, aes(x = hour, y = speed.med, group = picomp)) +
    geom_line(size = 1, aes(color = factor(ranking))) + xlab("Hour since lights on") +
    ylab("Median swimming speed (cm/s)") + scale_x_continuous(breaks = c(0,
    1, 2, 3, 4, 5, 6, 7, 8, 9, 10, 11)) + scale_color_manual(values = plasma_pal) +
    theme_classic() + theme(axis.text.x = element_text(size = 12), axis.title.x = element_text(size = 1
    axis.text.y = element_text(size = 12), axis.title.y = element_text(size = 16),
    legend.position = "none") + annotate("text", x = 1.1, y = 3, label = "A",
    size = 6) + annotate("text", x = 4, y = 2.8, label = "R = 0.65", size = 5,
    fontface = "italic")


# is behavior related to mother identity? one point for every hour in day
# 1
mom.plot <- ggplot(day1.test, aes(x = mother, y = speed.med)) + geom_violin(trim = F,
    fill = "lightgray") + geom_jitter(width = 0.2, size = 2, alpha = 0.6, aes(color = factor(ranking)))
    geom_point(day1.test, mapping = aes(x = mother, y = day1.speed, fill = factor(ranking)),
        shape = 21, size = 5) + scale_color_manual(values = plasma_pal) + scale_fill_manual(values = pl
    xlab("Mother identity") + ylab("Median swimming speed (cm/s)") + ylim(c(0,
    3)) + scale_x_discrete(limits = c("90.2", "69.3", "70.2", "70.3", "75.2",
    "80.1", "77.2", "80.2"), labels = c(`90.2` = "a", `69.3` = "b", `70.2` = "c",
    `70.3` = "d", `75.2` = "e", `80.1` = "f", `77.2` = "g", `80.2` = "h")) +
    theme_classic() + theme(axis.text.x = element_text(size = 12), axis.title.x = element_text(size = 1
    axis.title.y = element_blank(), axis.text.y = element_blank(), legend.position = "none") +
    annotate("text", x = 0.9, y = 3, label = "B", size = 6)




# While the models were all run on mean-centered TL, would be nice to
# graph absolute TL as that is a bit more meaningful so will re-run the
# model with the absolute TL, as opposed to centered TL
set.seed(472)
speed.day1.4.plot <- MCMCglmm(speed.med ~ hour.cen + TL, random = ~us(1 + hour.cen):picomp,
    prior = prior.id.slope, data = day1.com, family = "gaussian", nitt = 510000,
    burnin = 10000, thin = 200, verbose = F)

tl.slope <- as.vector(speed.day1.4.plot$Sol[, "TL"])
tl.inter <- as.vector(speed.day1.4.plot$Sol[, "(Intercept)"])
body.effects <- data.frame(tl.slope, tl.inter)

# is behavior related to body size?  estimates from day 1 model to report
# - only select 1000 of them for graphing
body.effects2 <- body.effects[1:1001, ]


body.plot <- ggplot() + geom_abline(body.effects2, mapping = aes(intercept = tl.inter,
    slope = tl.slope), color = "gray", alpha = 0.5) + geom_point(day1.test,
    mapping = aes(x = TL, y = speed.med, color = factor(ranking)), size = 2,
    alpha = 0.6) + geom_point(day1.test, mapping = aes(x = TL, y = day1.speed,
    fill = factor(ranking)), shape = 21, size = 5) + scale_color_manual(values = plasma_pal) +
    scale_fill_manual(values = plasma_pal) + xlab("Total length (cm)") + ylab("Median swimming speed (c
```

```
    theme_classic() + theme(axis.text.x = element_text(size = 12), axis.title.x = element_text(size = 16
    axis.title.y = element_blank(), axis.text.y = element_blank(), legend.position = "none") +
    annotate("text", x = 0.82, y = 3, label = "C", size = 6)


fig2 <- grid.arrange(day1.plot, mom.plot, body.plot, ncol = 3)

ggsave(file = "Fig 2_day one results_20220927.tiff", plot = fig2, height = 4,
    width = 12, dpi = 300)
ggsave(file = "Fig 2_day one results_20220927.png", plot = fig2, height = 4,
    width = 12, dpi = 300)
```

Fig 3 - rpt slice

This figure is for our second research question (how does behavioral variaton change over time?)

```
plasma_pal <- viridis::plasma(n = 30)
plasma_pal <- plasma_pal[1:26]

test <- gray.colors(6, start = 0, end = 0.8, gamma = 0.5)
scales::show_col(test)


pred.intercepts <- read.csv("Predicted individual BLUPs_slice model_220202.csv")

# to color lines according to behavior during obs 1
pred.rank <- pred.intercepts %>%
    filter(date == 0) %>%
    mutate(ranking = rank(blup)) %>%
    select(picomp, ranking)

pred.rank <- left_join(pred.intercepts, pred.rank) %>%
    arrange(ranking)

# reaction norm plot with predicted intercepts from slice models

blup.plot <- ggplot(pred.rank, aes(x = date, y = blup, group = picomp)) + geom_line(size = 1,
    aes(color = factor(ranking))) + xlab("") + scale_y_continuous(name = "Predicted swimming speed (cm/s
    ylab("Predicted swimming speed") + scale_x_continuous(breaks = c(0, 7, 14,
    21, 28, 35, 42, 49, 56, 63, 70)) + scale_color_manual(values = plasma_pal) +
    theme_classic() + theme(axis.text.x = element_blank(), axis.title.x = element_blank(),
    axis.text.y = element_text(size = 12), axis.title.y = element_text(size = 16),
    legend.position = "none") + annotate("text", x = -1, y = 2, label = "A",
    size = 6)



# repeatability by slices

rpt.slice.wide <- read.csv("repeatability estimates sliced across weeks_wide_220202.csv")
head(rpt.slice.wide)
```

```
rpt.plot <- ggplot(rpt.slice.wide, aes(x = date)) + geom_point(aes(y = rpt,
    color = "#000000"), size = 4) + geom_line(aes(x = date, y = rpt, color = "#000000")) +
    geom_errorbar(aes(ymin = lower.rpt, ymax = upper.rpt, width = 0.75, color = "#000000")) +
geom_errorbar(aes(x = date - 0.5, ymin = lower.id, ymax = upper.id, width = 0.75,
    color = "#000000")) + geom_line(aes(x = date - 0.5, y = post.id, color = "#959595")) +
    geom_point(aes(x = date - 0.5, y = post.id), shape = 21, color = "#000000",
        fill = "#959595", size = 4) +  fill = "#959595", size = 4) +
geom_errorbar(aes(x = date + 0.5, ymin = lower.w, ymax = upper.w, width = 0.75,
    color = "#000000")) + geom_line(aes(x = date + 0.5, y = post.w, color = "#CCCCCC")) +
    geom_point(aes(x = date + 0.5, y = post.w), shape = 21, color = "#000000",
        fill = "#CCCCCC", size = 4) +  fill = "#CCCCCC", size = 4) +
scale_x_continuous(breaks = c(0, 7, 14, 21, 28, 35, 42, 49, 56, 63, 70), labels = c(1,
    7, 14, 21, 28, 35, 42, 49, 56, 63, 70)) + scale_y_continuous(name = "Variance estimate") +
    labs(x = "Day", color = "Legend") + scale_color_manual(name = "", values = c("#000000",
    "#959595", "#CCCCCC"), labels = c("Repeatability", "Among-individual", "Within-individual")) +
    theme_classic() + theme(legend.position = c(0.3, 0.9), legend.text = element_text(size = 14),
    axis.text.x = element_text(size = 12), axis.title.x = element_text(size = 16),
    axis.text.y = element_text(size = 12), axis.title.y = element_text(size = 16)) +
    annotate("text", label = "B", size = 6, x = -1, y = 0.8)



layout <- "
A
B
"

fig3 <- blup.plot + rpt.plot + plot_layout(design = layout)

ggsave(file = "Fig 3_variance over time slide models_220610.tiff", plot = fig3,
    height = 10, width = 7, dpi = 300)
ggsave(file = "Fig 3_variance over time slide models_220610.png", plot = fig3,
    height = 10, width = 7, dpi = 300)
```

Fig 4 - weeks corr over time

Thsi figure is for our third research question (how predictive is early life behavior of later life behavior?)

```
among.corr <- read.csv("among individual correlations across weeks 220202.csv")

test <- gray.colors(5, start = 0, end = 0.9, gamma = 0.8)
scales::show_col(test)

# only select out correlations among 6 weeks diff
among.corr2 <- among.corr %>%
    filter(diff < 6)

colors <- c("#E6E6E6", "#A0A0A0", "#606060", "#292929", "#000000")

corr.over.time <- ggplot(among.corr2, aes(x = start.week, y = value)) + geom_point(aes(shape = factor(di
    fill = factor(diff)), color = "black", size = 4) + stat_smooth(method = "lm",
```

```
    se = F, aes(color = factor(diff))) + scale_color_manual("Correlation with \nbehavior",
    values = colors, labels = c("1 week later", "2 weeks later", "3 weeks later",
        "4 weeks later", "5 weeks later")) + scale_shape_manual("Correlation with \nbehavior",
    values = c(21:25), labels = c("1 week later", "2 weeks later", "3 weeks later",
        "4 weeks later", "5 weeks later")) + scale_fill_manual("Correlation with \nbehavior",
    values = colors, labels = c("1 week later", "2 weeks later", "3 weeks later",
        "4 weeks later", "5 weeks later")) + scale_x_continuous(breaks = c(1,
    2, 3, 4, 5, 6, 7, 8, 9, 10)) + xlab("Week") + ylab("Among-individual correlation") +
    scale_y_continuous(limits = c(0.4, 1), breaks = c(0.4, 0.6, 0.8, 1)) + annotate("text",
    label = "B", size = 10, x = 1, y = 0.95) + theme_classic() + theme(axis.text.x = element_text(size =
    axis.title.x = element_text(size = 18), axis.text.y = element_text(size = 14),
    axis.title.y = element_text(size = 18), legend.position = c(0.8, 0.25),
    legend.title = element_text(size = 18), legend.text = element_text(size = 16))
```

> **scatterplots by week** So I extracted the BLUPs for each individual in each week in the weekly.blups file above, now the goal is to create a scatterplot for each which would be 45 individual graphs, wonder if there is some way I can facet this. . . .

```
# need to get individual behaivor BLUPs from here
weekly.blups <- read.csv("weekly activity blups across weeks 220202.csv")

# want to create ranking based on week1 scores to better visualize who's
# who across weeks
weekly.blups <- weekly.blups %>%
    mutate(ranking = rank(week1)) %>%
    arrange(ranking)

# need to get weekly correlation coefficients from here
among.corr <- read.csv("among individual correlations across weeks 220202.csv")

plasma_pal <- viridis::plasma(n = 30)
plasma_pal <- plasma_pal[1:26]
# scales::show_col(plasma_pal)




# week 1 correlations
week12 <- ggplot(weekly.blups, aes(x = week2, y = week1)) + geom_abline(intercept = 0,
    slope = among.corr$value[1]) + xlab("Week 2") + ylab("Week 1") + annotate("text",
    label = "A", size = 10, x = -0.25, y = 2) + geom_point(aes(color = ranking),
    size = 3.5) + scale_color_viridis_c(option = "plasma") + scale_x_continuous(limits = c(-0.75,
    2.31), breaks = c(-0.75, 0, 0.75, 1.5, 2.25)) + scale_y_continuous(limits = c(-0.75,
    2.31), breaks = c(-0.75, 0, 0.75, 1.5, 2.25)) + theme_classic() + theme(legend.position = "none",
    plot.margin = margin(0, 0, 0, 0), axis.text = element_blank(), axis.title = element_text(size = 16)

week13 <- ggplot(weekly.blups, aes(x = week3, y = week1)) + geom_abline(intercept = 0,
    slope = among.corr$value[2]) + geom_point(aes(color = ranking), size = 3.5) +
    scale_color_viridis_c(option = "plasma") + scale_x_continuous(limits = c(-0.75,
    2.31), breaks = c(-0.75, 0, 0.75, 1.5, 2.25)) + scale_y_continuous(limits = c(-0.75,
    2.31), breaks = c(-0.75, 0, 0.75, 1.5, 2.25)) + theme_classic() + theme(legend.position = "none",
    axis.title = element_blank(), axis.text = element_blank(), plot.margin = margin(0,
        0, 0, 0))
```

```
week14 <- ggplot(weekly.blups, aes(x = week4, y = week1)) + geom_abline(intercept = 0,
    slope = among.corr$value[3]) + geom_point(aes(color = ranking), size = 3.5) +
    scale_color_viridis_c(option = "plasma") + scale_x_continuous(limits = c(-0.75,
    2.31), breaks = c(-0.75, 0, 0.75, 1.5, 2.25)) + scale_y_continuous(limits = c(-0.75,
    2.31), breaks = c(-0.75, 0, 0.75, 1.5, 2.25)) + theme_classic() + theme(legend.position = "none",
    axis.title = element_blank(), axis.text = element_blank(), plot.margin = margin(0,
        0, 0, 0))

week15 <- ggplot(weekly.blups, aes(x = week5, y = week1)) + geom_abline(intercept = 0,
    slope = among.corr$value[4]) + geom_point(aes(color = ranking), size = 3.5) +
    scale_color_viridis_c(option = "plasma") + scale_x_continuous(limits = c(-0.75,
    2.31), breaks = c(-0.75, 0, 0.75, 1.5, 2.25)) + scale_y_continuous(limits = c(-0.75,
    2.31), breaks = c(-0.75, 0, 0.75, 1.5, 2.25)) + theme_classic() + theme(legend.position = "none",
    axis.title = element_blank(), axis.text = element_blank(), plot.margin = margin(0,
        0, 0, 0))

week16 <- ggplot(weekly.blups, aes(x = week6, y = week1)) + geom_abline(intercept = 0,
    slope = among.corr$value[5]) + geom_point(aes(color = ranking), size = 3.5) +
    scale_color_viridis_c(option = "plasma") + scale_x_continuous(limits = c(-0.75,
    2.31), breaks = c(-0.75, 0, 0.75, 1.5, 2.25)) + scale_y_continuous(limits = c(-0.75,
    2.31), breaks = c(-0.75, 0, 0.75, 1.5, 2.25)) + theme_classic() + theme(legend.position = "none",
    axis.title = element_blank(), axis.text = element_blank(), plot.margin = margin(0,
        0, 0, 0))

week17 <- ggplot(weekly.blups, aes(x = week7, y = week1)) + geom_abline(intercept = 0,
    slope = among.corr$value[6]) + geom_point(aes(color = ranking), size = 3.5) +
    scale_color_viridis_c(option = "plasma") + scale_x_continuous(limits = c(-0.75,
    2.31), breaks = c(-0.75, 0, 0.75, 1.5, 2.25)) + scale_y_continuous(limits = c(-0.75,
    2.31), breaks = c(-0.75, 0, 0.75, 1.5, 2.25)) + theme_classic() + theme(legend.position = "none",
    axis.title = element_blank(), axis.text = element_blank(), plot.margin = margin(0,
        0, 0, 0))

week18 <- ggplot(weekly.blups, aes(x = week8, y = week1)) + geom_abline(intercept = 0,
    slope = among.corr$value[7]) + geom_point(aes(color = ranking), size = 3.5) +
    scale_color_viridis_c(option = "plasma") + scale_x_continuous(limits = c(-0.75,
    2.31), breaks = c(-0.75, 0, 0.75, 1.5, 2.25)) + scale_y_continuous(limits = c(-0.75,
    2.31), breaks = c(-0.75, 0, 0.75, 1.5, 2.25)) + theme_classic() + theme(legend.position = "none",
    axis.title = element_blank(), axis.text = element_blank(), plot.margin = margin(0,
        0, 0, 0))

week19 <- ggplot(weekly.blups, aes(x = week9, y = week1)) + geom_abline(intercept = 0,
    slope = among.corr$value[8]) + geom_point(aes(color = ranking), size = 3.5) +
    scale_color_viridis_c(option = "plasma") + scale_x_continuous(limits = c(-0.75,
    2.31), breaks = c(-0.75, 0, 0.75, 1.5, 2.25)) + scale_y_continuous(limits = c(-0.75,
    2.31), breaks = c(-0.75, 0, 0.75, 1.5, 2.25)) + theme_classic() + theme(legend.position = "none",
    axis.title = element_blank(), axis.text = element_blank(), plot.margin = margin(0,
        0, 0, 0))

week110 <- ggplot(weekly.blups, aes(x = week10, y = week1)) + geom_abline(intercept = 0,
    slope = among.corr$value[9]) + geom_point(aes(color = ranking), size = 3.5) +
    scale_color_viridis_c(option = "plasma") + scale_x_continuous(limits = c(-0.75,
    2.31), breaks = c(-0.75, 0, 0.75, 1.5, 2.25)) + scale_y_continuous(limits = c(-0.75,
    2.31), breaks = c(-0.75, 0, 0.75, 1.5, 2.25)) + theme_classic() + theme(legend.position = "none",
```

```
    axis.title = element_blank(), axis.text = element_blank(), plot.margin = margin(0,
        0, 0, 0))

# week 2 correlations
week23 <- ggplot(weekly.blups, aes(x = week3, y = week2)) + geom_abline(intercept = 0,
    slope = among.corr$value[10]) + ylab("Week 2") + xlab("Week 3") + geom_point(aes(color = ranking),
    size = 3.5) + scale_color_viridis_c(option = "plasma") + scale_x_continuous(limits = c(-0.75,
    2.31), breaks = c(-0.75, 0, 0.75, 1.5, 2.25)) + scale_y_continuous(limits = c(-0.75,
    2.31), breaks = c(-0.75, 0, 0.75, 1.5, 2.25)) + theme_classic() + theme(legend.position = "none",
    plot.margin = margin(0, 0, 0, 0), axis.text = element_blank(), axis.title = element_text(size = 16))

week24 <- ggplot(weekly.blups, aes(x = week4, y = week2)) + geom_abline(intercept = 0,
    slope = among.corr$value[11]) + geom_point(aes(color = ranking), size = 3.5) +
    scale_color_viridis_c(option = "plasma") + scale_x_continuous(limits = c(-0.75,
    2.31), breaks = c(-0.75, 0, 0.75, 1.5, 2.25)) + scale_y_continuous(limits = c(-0.75,
    2.31), breaks = c(-0.75, 0, 0.75, 1.5, 2.25)) + theme_classic() + theme(legend.position = "none",
    axis.title = element_blank(), axis.text = element_blank(), plot.margin = margin(0,
        0, 0, 0))

week25 <- ggplot(weekly.blups, aes(x = week5, y = week2)) + geom_abline(intercept = 0,
    slope = among.corr$value[12]) + geom_point(aes(color = ranking), size = 3.5) +
    scale_color_viridis_c(option = "plasma") + scale_x_continuous(limits = c(-0.75,
    2.31), breaks = c(-0.75, 0, 0.75, 1.5, 2.25)) + scale_y_continuous(limits = c(-0.75,
    2.31), breaks = c(-0.75, 0, 0.75, 1.5, 2.25)) + theme_classic() + theme(legend.position = "none",
    axis.title = element_blank(), axis.text = element_blank(), plot.margin = margin(0,
        0, 0, 0))

week26 <- ggplot(weekly.blups, aes(x = week6, y = week2)) + geom_abline(intercept = 0,
    slope = among.corr$value[13]) + geom_point(aes(color = ranking), size = 3.5) +
    scale_color_viridis_c(option = "plasma") + scale_x_continuous(limits = c(-0.75,
    2.31), breaks = c(-0.75, 0, 0.75, 1.5, 2.25)) + scale_y_continuous(limits = c(-0.75,
    2.31), breaks = c(-0.75, 0, 0.75, 1.5, 2.25)) + theme_classic() + theme(legend.position = "none",
    axis.title = element_blank(), axis.text = element_blank(), plot.margin = margin(0,
        0, 0, 0))

week27 <- ggplot(weekly.blups, aes(x = week7, y = week2)) + geom_abline(intercept = 0,
    slope = among.corr$value[14]) + geom_point(aes(color = ranking), size = 3.5) +
    scale_color_viridis_c(option = "plasma") + scale_x_continuous(limits = c(-0.75,
    2.31), breaks = c(-0.75, 0, 0.75, 1.5, 2.25)) + scale_y_continuous(limits = c(-0.75,
    2.31), breaks = c(-0.75, 0, 0.75, 1.5, 2.25)) + theme_classic() + theme(legend.position = "none",
    axis.title = element_blank(), axis.text = element_blank(), plot.margin = margin(0,
        0, 0, 0))

week28 <- ggplot(weekly.blups, aes(x = week8, y = week2)) + geom_abline(intercept = 0,
    slope = among.corr$value[15]) + geom_point(aes(color = ranking), size = 3.5) +
    scale_color_viridis_c(option = "plasma") + scale_x_continuous(limits = c(-0.75,
    2.31), breaks = c(-0.75, 0, 0.75, 1.5, 2.25)) + scale_y_continuous(limits = c(-0.75,
    2.31), breaks = c(-0.75, 0, 0.75, 1.5, 2.25)) + theme_classic() + theme(legend.position = "none",
    axis.title = element_blank(), axis.text = element_blank(), plot.margin = margin(0,
        0, 0, 0))

week29 <- ggplot(weekly.blups, aes(x = week9, y = week2)) + geom_abline(intercept = 0,
    slope = among.corr$value[16]) + geom_point(aes(color = ranking), size = 3.5) +
```

```r
    scale_color_viridis_c(option = "plasma") + scale_x_continuous(limits = c(-0.75,
    2.31), breaks = c(-0.75, 0, 0.75, 1.5, 2.25)) + scale_y_continuous(limits = c(-0.75,
    2.31), breaks = c(-0.75, 0, 0.75, 1.5, 2.25)) + theme_classic() + theme(legend.position = "none",
    axis.title = element_blank(), axis.text = element_blank(), plot.margin = margin(0,
        0, 0, 0))

week210 <- ggplot(weekly.blups, aes(x = week10, y = week2)) + geom_abline(intercept = 0,
    slope = among.corr$value[17]) + geom_point(aes(color = ranking), size = 3.5) +
    scale_color_viridis_c(option = "plasma") + scale_x_continuous(limits = c(-0.75,
    2.31), breaks = c(-0.75, 0, 0.75, 1.5, 2.25)) + scale_y_continuous(limits = c(-0.75,
    2.31), breaks = c(-0.75, 0, 0.75, 1.5, 2.25)) + theme_classic() + theme(legend.position = "none",
    axis.title = element_blank(), axis.text = element_blank(), plot.margin = margin(0,
        0, 0, 0))

# Week 3 plots

week34 <- ggplot(weekly.blups, aes(x = week4, y = week3)) + geom_abline(intercept = 0,
    slope = among.corr$value[18]) + ylab("Week 3") + xlab("Week 4") + geom_point(aes(color = ranking),
    size = 3.5) + scale_color_viridis_c(option = "plasma") + scale_x_continuous(limits = c(-0.75,
    2.31), breaks = c(-0.75, 0, 0.75, 1.5, 2.25)) + scale_y_continuous(limits = c(-0.75,
    2.31), breaks = c(-0.75, 0, 0.75, 1.5, 2.25)) + theme_classic() + theme(legend.position = "none",
    plot.margin = margin(0, 0, 0, 0), axis.text = element_blank(), axis.title = element_text(size = 16))

week35 <- ggplot(weekly.blups, aes(x = week5, y = week3)) + geom_abline(intercept = 0,
    slope = among.corr$value[19]) + geom_point(aes(color = ranking), size = 3.5) +
    scale_color_viridis_c(option = "plasma") + scale_x_continuous(limits = c(-0.75,
    2.31), breaks = c(-0.75, 0, 0.75, 1.5, 2.25)) + scale_y_continuous(limits = c(-0.75,
    2.31), breaks = c(-0.75, 0, 0.75, 1.5, 2.25)) + theme_classic() + theme(legend.position = "none",
    axis.title = element_blank(), axis.text = element_blank(), plot.margin = margin(0,
        0, 0, 0))

week36 <- ggplot(weekly.blups, aes(x = week6, y = week3)) + geom_abline(intercept = 0,
    slope = among.corr$value[20]) + geom_point(aes(color = ranking), size = 3.5) +
    scale_color_viridis_c(option = "plasma") + scale_x_continuous(limits = c(-0.75,
    2.31), breaks = c(-0.75, 0, 0.75, 1.5, 2.25)) + scale_y_continuous(limits = c(-0.75,
    2.31), breaks = c(-0.75, 0, 0.75, 1.5, 2.25)) + theme_classic() + theme(legend.position = "none",
    axis.title = element_blank(), axis.text = element_blank(), plot.margin = margin(0,
        0, 0, 0))

week37 <- ggplot(weekly.blups, aes(x = week7, y = week3)) + geom_abline(intercept = 0,
    slope = among.corr$value[21]) + geom_point(aes(color = ranking), size = 3.5) +
    scale_color_viridis_c(option = "plasma") + scale_x_continuous(limits = c(-0.75,
    2.31), breaks = c(-0.75, 0, 0.75, 1.5, 2.25)) + scale_y_continuous(limits = c(-0.75,
    2.31), breaks = c(-0.75, 0, 0.75, 1.5, 2.25)) + theme_classic() + theme(legend.position = "none",
    axis.title = element_blank(), axis.text = element_blank(), plot.margin = margin(0,
        0, 0, 0))

week38 <- ggplot(weekly.blups, aes(x = week8, y = week3)) + geom_abline(intercept = 0,
    slope = among.corr$value[22]) + geom_point(aes(color = ranking), size = 3.5) +
    scale_color_viridis_c(option = "plasma") + scale_x_continuous(limits = c(-0.75,
    2.31), breaks = c(-0.75, 0, 0.75, 1.5, 2.25)) + scale_y_continuous(limits = c(-0.75,
    2.31), breaks = c(-0.75, 0, 0.75, 1.5, 2.25)) + theme_classic() + theme(legend.position = "none",
    axis.title = element_blank(), axis.text = element_blank(), plot.margin = margin(0,
```

```
        0, 0, 0))

week39 <- ggplot(weekly.blups, aes(x = week9, y = week3)) + geom_abline(intercept = 0,
    slope = among.corr$value[23]) + geom_point(aes(color = ranking), size = 3.5) +
    scale_color_viridis_c(option = "plasma") + scale_x_continuous(limits = c(-0.75,
    2.31), breaks = c(-0.75, 0, 0.75, 1.5, 2.25)) + scale_y_continuous(limits = c(-0.75,
    2.31), breaks = c(-0.75, 0, 0.75, 1.5, 2.25)) + theme_classic() + theme(legend.position = "none",
    axis.title = element_blank(), axis.text = element_blank(), plot.margin = margin(0,
        0, 0, 0))

week310 <- ggplot(weekly.blups, aes(x = week10, y = week3)) + geom_abline(intercept = 0,
    slope = among.corr$value[24]) + geom_point(aes(color = ranking), size = 3.5) +
    scale_color_viridis_c(option = "plasma") + scale_x_continuous(limits = c(-0.75,
    2.31), breaks = c(-0.75, 0, 0.75, 1.5, 2.25)) + scale_y_continuous(limits = c(-0.75,
    2.31), breaks = c(-0.75, 0, 0.75, 1.5, 2.25)) + theme_classic() + theme(legend.position = "none",
    axis.title = element_blank(), axis.text = element_blank(), plot.margin = margin(0,
        0, 0, 0))

# week 4 plots

week45 <- ggplot(weekly.blups, aes(x = week5, y = week4)) + geom_abline(intercept = 0,
    slope = among.corr$value[25]) + ylab("Week 4") + xlab("Week 5") + geom_point(aes(color = ranking),
    size = 3.5) + scale_color_viridis_c(option = "plasma") + scale_x_continuous(limits = c(-0.75,
    2.31), breaks = c(-0.75, 0, 0.75, 1.5, 2.25)) + scale_y_continuous(limits = c(-0.75,
    2.31), breaks = c(-0.75, 0, 0.75, 1.5, 2.25)) + theme_classic() + theme(legend.position = "none",
    plot.margin = margin(0, 0, 0, 0), axis.text = element_blank(), axis.title = element_text(size = 16))

week46 <- ggplot(weekly.blups, aes(x = week6, y = week4)) + geom_abline(intercept = 0,
    slope = among.corr$value[26]) + geom_point(aes(color = ranking), size = 3.5) +
    scale_color_viridis_c(option = "plasma") + scale_x_continuous(limits = c(-0.75,
    2.31), breaks = c(-0.75, 0, 0.75, 1.5, 2.25)) + scale_y_continuous(limits = c(-0.75,
    2.31), breaks = c(-0.75, 0, 0.75, 1.5, 2.25)) + theme_classic() + theme(legend.position = "none",
    axis.title = element_blank(), axis.text = element_blank(), plot.margin = margin(0,
        0, 0, 0))

week47 <- ggplot(weekly.blups, aes(x = week7, y = week4)) + geom_abline(intercept = 0,
    slope = among.corr$value[27]) + geom_point(aes(color = ranking), size = 3.5) +
    scale_color_viridis_c(option = "plasma") + scale_x_continuous(limits = c(-0.75,
    2.31), breaks = c(-0.75, 0, 0.75, 1.5, 2.25)) + scale_y_continuous(limits = c(-0.75,
    2.31), breaks = c(-0.75, 0, 0.75, 1.5, 2.25)) + theme_classic() + theme(legend.position = "none",
    axis.title = element_blank(), axis.text = element_blank(), plot.margin = margin(0,
        0, 0, 0))

week48 <- ggplot(weekly.blups, aes(x = week8, y = week4)) + geom_abline(intercept = 0,
    slope = among.corr$value[28]) + geom_point(aes(color = ranking), size = 3.5) +
    scale_color_viridis_c(option = "plasma") + scale_x_continuous(limits = c(-0.75,
    2.31), breaks = c(-0.75, 0, 0.75, 1.5, 2.25)) + scale_y_continuous(limits = c(-0.75,
    2.31), breaks = c(-0.75, 0, 0.75, 1.5, 2.25)) + theme_classic() + theme(legend.position = "none",
    axis.title = element_blank(), axis.text = element_blank(), plot.margin = margin(0,
        0, 0, 0))

week49 <- ggplot(weekly.blups, aes(x = week9, y = week4)) + geom_abline(intercept = 0,
    slope = among.corr$value[29]) + geom_point(aes(color = ranking), size = 3.5) +
```

```
    scale_color_viridis_c(option = "plasma") + scale_x_continuous(limits = c(-0.75,
    2.31), breaks = c(-0.75, 0, 0.75, 1.5, 2.25)) + scale_y_continuous(limits = c(-0.75,
    2.31), breaks = c(-0.75, 0, 0.75, 1.5, 2.25)) + theme_classic() + theme(legend.position = "none",
    axis.title = element_blank(), axis.text = element_blank(), plot.margin = margin(0,
        0, 0, 0))

week410 <- ggplot(weekly.blups, aes(x = week10, y = week4)) + geom_abline(intercept = 0,
    slope = among.corr$value[30]) + geom_point(aes(color = ranking), size = 3.5) +
    scale_color_viridis_c(option = "plasma") + scale_x_continuous(limits = c(-0.75,
    2.31), breaks = c(-0.75, 0, 0.75, 1.5, 2.25)) + scale_y_continuous(limits = c(-0.75,
    2.31), breaks = c(-0.75, 0, 0.75, 1.5, 2.25)) + theme_classic() + theme(legend.position = "none",
    axis.title = element_blank(), axis.text = element_blank(), plot.margin = margin(0,
        0, 0, 0))

# week 5 plots

week56 <- ggplot(weekly.blups, aes(x = week6, y = week5)) + geom_abline(intercept = 0,
    slope = among.corr$value[31]) + ylab("Week 5") + xlab("Week 6") + geom_point(aes(color = ranking),
    size = 3.5) + scale_color_viridis_c(option = "plasma") + scale_x_continuous(limits = c(-0.75,
    2.31), breaks = c(-0.75, 0, 0.75, 1.5, 2.25)) + scale_y_continuous(limits = c(-0.75,
    2.31), breaks = c(-0.75, 0, 0.75, 1.5, 2.25)) + theme_classic() + theme(legend.position = "none",
    plot.margin = margin(0, 0, 0, 0), axis.text = element_blank(), axis.title = element_text(size = 16))

week57 <- ggplot(weekly.blups, aes(x = week7, y = week5)) + geom_abline(intercept = 0,
    slope = among.corr$value[32]) + geom_point(aes(color = ranking), size = 3.5) +
    scale_color_viridis_c(option = "plasma") + scale_x_continuous(limits = c(-0.75,
    2.31), breaks = c(-0.75, 0, 0.75, 1.5, 2.25)) + scale_y_continuous(limits = c(-0.75,
    2.31), breaks = c(-0.75, 0, 0.75, 1.5, 2.25)) + theme_classic() + theme(legend.position = "none",
    axis.title = element_blank(), axis.text = element_blank(), plot.margin = margin(0,
        0, 0, 0))

week58 <- ggplot(weekly.blups, aes(x = week8, y = week5)) + geom_abline(intercept = 0,
    slope = among.corr$value[33]) + geom_point(aes(color = ranking), size = 3.5) +
    scale_color_viridis_c(option = "plasma") + scale_x_continuous(limits = c(-0.75,
    2.31), breaks = c(-0.75, 0, 0.75, 1.5, 2.25)) + scale_y_continuous(limits = c(-0.75,
    2.31), breaks = c(-0.75, 0, 0.75, 1.5, 2.25)) + theme_classic() + theme(legend.position = "none",
    axis.title = element_blank(), axis.text = element_blank(), plot.margin = margin(0,
        0, 0, 0))

week59 <- ggplot(weekly.blups, aes(x = week9, y = week5)) + geom_abline(intercept = 0,
    slope = among.corr$value[34]) + geom_point(aes(color = ranking), size = 3) +
    scale_color_viridis_c(option = "plasma") + scale_x_continuous(limits = c(-0.75,
    2.31), breaks = c(-0.75, 0, 0.75, 1.5, 2.25)) + scale_y_continuous(limits = c(-0.75,
    2.31), breaks = c(-0.75, 0, 0.75, 1.5, 2.25)) + theme_classic() + theme(legend.position = "none",
    axis.title = element_blank(), axis.text = element_blank(), plot.margin = margin(0,
        0, 0, 0))

week510 <- ggplot(weekly.blups, aes(x = week10, y = week5)) + geom_abline(intercept = 0,
    slope = among.corr$value[35]) + geom_point(aes(color = ranking), size = 3.5) +
    scale_color_viridis_c(option = "plasma") + scale_x_continuous(limits = c(-0.75,
    2.31), breaks = c(-0.75, 0, 0.75, 1.5, 2.25)) + scale_y_continuous(limits = c(-0.75,
    2.31), breaks = c(-0.75, 0, 0.75, 1.5, 2.25)) + theme_classic() + theme(legend.position = "none",
    axis.title = element_blank(), axis.text = element_blank(), plot.margin = margin(0,
```

```
        0, 0, 0))


# week 6 plots

week67 <- ggplot(weekly.blups, aes(x = week7, y = week6)) + geom_abline(intercept = 0,
    slope = among.corr$value[36]) + ylab("Week 6") + xlab("Week 7") + geom_point(aes(color = ranking),
    size = 3.5) + scale_color_viridis_c(option = "plasma") + scale_x_continuous(limits = c(-0.75,
    2.31), breaks = c(-0.75, 0, 0.75, 1.5, 2.25)) + scale_y_continuous(limits = c(-0.75,
    2.31), breaks = c(-0.75, 0, 0.75, 1.5, 2.25)) + theme_classic() + theme(legend.position = "none",
    plot.margin = margin(0, 0, 0, 0), axis.text = element_blank(), axis.title = element_text(size = 16))

week68 <- ggplot(weekly.blups, aes(x = week8, y = week6)) + geom_abline(intercept = 0,
    slope = among.corr$value[37]) + geom_point(aes(color = ranking), size = 3.5) +
    scale_color_viridis_c(option = "plasma") + scale_x_continuous(limits = c(-0.75,
    2.31), breaks = c(-0.75, 0, 0.75, 1.5, 2.25)) + scale_y_continuous(limits = c(-0.75,
    2.31), breaks = c(-0.75, 0, 0.75, 1.5, 2.25)) + theme_classic() + theme(legend.position = "none",
    axis.title = element_blank(), axis.text = element_blank(), plot.margin = margin(0,
        0, 0, 0))

week69 <- ggplot(weekly.blups, aes(x = week9, y = week6)) + geom_abline(intercept = 0,
    slope = among.corr$value[38]) + geom_point(aes(color = ranking), size = 3.5) +
    scale_color_viridis_c(option = "plasma") + scale_x_continuous(limits = c(-0.75,
    2.31), breaks = c(-0.75, 0, 0.75, 1.5, 2.25)) + scale_y_continuous(limits = c(-0.75,
    2.31), breaks = c(-0.75, 0, 0.75, 1.5, 2.25)) + theme_classic() + theme(legend.position = "none",
    axis.title = element_blank(), axis.text = element_blank(), plot.margin = margin(0,
        0, 0, 0))

week610 <- ggplot(weekly.blups, aes(x = week10, y = week6)) + geom_abline(intercept = 0,
    slope = among.corr$value[39]) + geom_point(aes(color = ranking), size = 3.5) +
    scale_color_viridis_c(option = "plasma") + scale_x_continuous(limits = c(-0.75,
    2.31), breaks = c(-0.75, 0, 0.75, 1.5, 2.25)) + scale_y_continuous(limits = c(-0.75,
    2.31), breaks = c(-0.75, 0, 0.75, 1.5, 2.25)) + theme_classic() + theme(legend.position = "none",
    axis.title = element_blank(), axis.text = element_blank(), plot.margin = margin(0,
        0, 0, 0))

# week 7 plots

week78 <- ggplot(weekly.blups, aes(x = week8, y = week7)) + geom_abline(intercept = 0,
    slope = among.corr$value[40]) + ylab("Week 7") + xlab("Week 8") + geom_point(aes(color = ranking),
    size = 3.5) + scale_color_viridis_c(option = "plasma") + scale_x_continuous(limits = c(-0.75,
    2.31), breaks = c(-0.75, 0, 0.75, 1.5, 2.25)) + scale_y_continuous(limits = c(-0.75,
    2.31), breaks = c(-0.75, 0, 0.75, 1.5, 2.25)) + theme_classic() + theme(legend.position = "none",
    plot.margin = margin(0, 0, 0, 0), axis.text = element_blank(), axis.title = element_text(size = 16))

week79 <- ggplot(weekly.blups, aes(x = week9, y = week7)) + geom_abline(intercept = 0,
    slope = among.corr$value[41]) + geom_point(aes(color = ranking), size = 3.5) +
    scale_color_viridis_c(option = "plasma") + scale_x_continuous(limits = c(-0.75,
    2.31), breaks = c(-0.75, 0, 0.75, 1.5, 2.25)) + scale_y_continuous(limits = c(-0.75,
    2.31), breaks = c(-0.75, 0, 0.75, 1.5, 2.25)) + theme_classic() + theme(legend.position = "none",
    axis.title = element_blank(), axis.text = element_blank(), plot.margin = margin(0,
        0, 0, 0))
```

```r
week710 <- ggplot(weekly.blups, aes(x = week10, y = week7)) + geom_abline(intercept = 0,
    slope = among.corr$value[42]) + geom_point(aes(color = ranking), size = 3.5) +
    scale_color_viridis_c(option = "plasma") + scale_x_continuous(limits = c(-0.75,
    2.31), breaks = c(-0.75, 0, 0.75, 1.5, 2.25)) + scale_y_continuous(limits = c(-0.75,
    2.31), breaks = c(-0.75, 0, 0.75, 1.5, 2.25)) + theme_classic() + theme(legend.position = "none",
    axis.title = element_blank(), axis.text = element_blank(), plot.margin = margin(0,
        0, 0, 0))

# Week 8 plots

week89 <- ggplot(weekly.blups, aes(x = week9, y = week8)) + geom_abline(intercept = 0,
    slope = among.corr$value[43]) + ylab("Week 8") + xlab("Week 9") + geom_point(aes(color = ranking),
    size = 3.5) + scale_color_viridis_c(option = "plasma") + scale_x_continuous(limits = c(-0.75,
    2.31), breaks = c(-0.75, 0, 0.75, 1.5, 2.25)) + scale_y_continuous(limits = c(-0.75,
    2.31), breaks = c(-0.75, 0, 0.75, 1.5, 2.25)) + theme_classic() + theme(legend.position = "none",
    plot.margin = margin(0, 0, 0, 0), axis.text = element_blank(), axis.title = element_text(size = 16))

week810 <- ggplot(weekly.blups, aes(x = week10, y = week8)) + geom_abline(intercept = 0,
    slope = among.corr$value[44]) + geom_point(aes(color = ranking), size = 3.5) +
    scale_color_viridis_c(option = "plasma") + scale_x_continuous(limits = c(-0.75,
    2.31), breaks = c(-0.75, 0, 0.75, 1.5, 2.25)) + scale_y_continuous(limits = c(-0.75,
    2.31), breaks = c(-0.75, 0, 0.75, 1.5, 2.25)) + theme_classic() + theme(legend.position = "none",
    axis.title = element_blank(), axis.text = element_blank(), plot.margin = margin(0,
        0, 0, 0))

# week 9 plots

week910 <- ggplot(weekly.blups, aes(x = week10, y = week9)) + geom_abline(intercept = 0,
    slope = among.corr$value[45]) + ylab("Week 9") + xlab("Week 10") + geom_point(aes(color = ranking),
    size = 3.5) + scale_color_viridis_c(option = "plasma") + scale_x_continuous(limits = c(-0.75,
    2.31), breaks = c(-0.75, 0, 0.75, 1.5, 2.25)) + scale_y_continuous(limits = c(-0.75,
    2.31), breaks = c(-0.75, 0, 0.75, 1.5, 2.25)) + theme_classic() + theme(legend.position = "none",
    plot.margin = margin(0, 0, 0, 0), axis.text = element_blank(), axis.title = element_text(size = 16))


# with patchwork instead

layout <- "
aAbBcCdDe
#EfFgGhHi
##IjJkKlL
###mMnNoO
####pPqQr
WWWW#RsSt
WWWW##TuU
WWWW###vV
WWWW####w
"

matrix.fig2 <- week12 + week13 + week14 + week15 + week16 + week17 + week18 +
    week19 + week110 + week23 + week24 + week25 + week26 + week27 + week28 +
    week29 + week210 + week34 + week35 + week36 + week37 + week38 + week39 +
    week310 + week45 + week46 + week47 + week48 + week49 + week410 + week56 +
```

```
    week57 + week58 + week59 + week510 + week67 + week68 + week69 + week610 +
    week78 + week79 + week710 + week89 + week810 + week910 + corr.over.time +
    plot_layout(design = layout)

ggsave("Fig 4_upper right_weekly scatter and corr over time 220222.png", plot = matrix.fig2,
    height = 15, width = 15, dpi = 600)
ggsave("Fig 4_upper right_weekly scatter and corr over time 220222.tiff", plot = matrix.fig2,
    height = 15, width = 15, dpi = 600)
```

5 - SUPPLEMENTAL FIGURES

Fig - behavioral correlations

```
# DAY 1 ONLY

set.seed(410)
behav.day1.cor <- MCMCglmm(cbind(speed.med, propmoving, distmoved.iqr, borderdist.med) ~
    trait - 1, random = ~us(trait):picomp, rcov = ~us(trait):units, family = c(rep("gaussian",
    4)), prior = prior.cov4, nitt = 5000 * 100, thin = 100, burnin = 1000, verbose = F,
    data = day1.com)

# Among indv corr on Day 1 ----
behav.day1.among <- matrix(posterior.mode(posterior.cor(behav.day1.cor$VCV[,
    1:16])), 4, 4, dimnames = list(c("Med.speed", "Prop.moving", "IQR.speed",
    "Med.border"), c("Med.speed", "Prop.moving", "IQR.speed", "Med.border")))

day1.corr <- ggcorrplot(behav.day1.among, type = "lower", lab = T) + theme(legend.position = "none") +
    annotate("text", label = "A", x = 0.6, y = 3.4, size = 5)

# ENTIRE OBSERVATION PERIOD
set.seed(100)
behav.all.cor <- MCMCglmm(cbind(speed.med, propmoving, distmoved.iqr, borderdist.med) ~
    trait - 1, random = ~us(trait):picomp, rcov = ~us(trait):units, family = c(rep("gaussian",
    4)), prior = prior.cov4, nitt = 5000 * 100, thin = 100, burnin = 1000, verbose = F,
    data = indv.com)

# Model for entire observation period ----
behav.matrix.all <- matrix(posterior.mode(posterior.cor(behav.all.cor$VCV[,
    1:16])), 4, 4, dimnames = list(c("Med.speed", "Prop.moving", "IQR.speed",
    "Med.border"), c("Med.speed", "Prop.moving", "IQR.speed", "Med.border")))


entire.corr <- ggcorrplot(behav.matrix.all, type = "lower", lab = T) + theme(axis.text.y = element_blanl
    annotate("text", label = "B", x = 0.6, y = 3.4, size = 5)


layout <- "
AB
"

corrplots <- day1.corr + entire.corr + plot_layout(design = layout)
```

```
# ggsave(file = 'Fig_behav correlations_220209.tiff', plot = corrplots,
# height = 5, width = 10, dpi = 300)
```

Fig - growth rates

```
indv.tl <- indv.com %>%
    group_by(picomp, week) %>%
    summarise(TL = unique(TL))


colors.id <- c("#abcd53", "#9b51ea", "#73e24b", "#d24cd8", "#bdd830", "#506af2",
    "#e4c82d", "#7a69db", "#4ba338", "#dd47b2", "#5ae08c", "#e34086", "#6cddba",
    "#e43649", "#4bd0d7", "#e64a22", "#5283e2", "#e6a63e", "#b16dd7", "#d0bc51",
    "#9180d5", "#e77e2e", "#6fb2e6", "#bc6c21", "#5c81bc", "#a47e27", "#bc65b1",
    "#9bd88b", "#d45267", "#449769", "#d9634d", "#6f8e3c", "#e3a0dc", "#8c833d",
    "#9b76b4", "#c9ca83", "#c86992", "#e5a775", "#d07878", "#b47046")

growth.plot <- ggplot(indv.tl, aes(x = week, y = TL, group = picomp, color = picomp)) +
    geom_line(size = 1) + xlab("Week") + ylab("Total length (cm)") + scale_x_continuous(limits = c(1,
    10), breaks = c(1, 2, 3, 4, 5, 6, 7, 8, 9, 10)) + ylim(c(0.75, 3)) + scale_color_viridis_d(option =
    theme_classic() + theme(axis.text.x = element_text(size = 12), axis.title.x = element_text(size = 1
    axis.text.y = element_text(size = 12), axis.title.y = element_text(size = 16),
    legend.position = "none")


# ggsave(file = 'Fig_body size over time_220202.tiff', plot = growth.plot,
# height = 6, width = 7, dpi = 300)
```

Fig - effects of body size on behavior

We have really strong statistical support to show that aspects of body size and growth rate don't seem to really explain variation in behavior, but would be good to have some nice graphs to back this up too.

```
# weekly body size by weekly behavior
body.behav <- indv.com %>%
    group_by(week, picomp) %>%
    summarise(speed = median(speed.med), body = first(TL))

plasma_pal <- viridis::plasma(n = 26)
plasma_rand <- sample(plasma_pal)

body.plot <- ggplot(body.behav, aes(x = body, y = speed, group = picomp, color = picomp)) +
    geom_point() + stat_smooth(method = "lm", se = F) + xlab("Weekly body size (cm)") +
    ylab("Weekly median swimming speed") + scale_color_manual(values = plasma_rand) +
    theme_classic() + theme(legend.position = "none")

# overall body size versus overall behavior
body.behav2 <- indv.com %>%
    group_by(picomp) %>%
    summarise(speed = median(speed.med), body = mean(TL))
```

```r
body.plot2 <- ggplot(body.behav2, aes(x = body, y = speed)) + geom_point(size = 3) +
    xlab("Average body size") + ylab("Overall median swimming speed") + theme_classic()


# day 1 behavior
day1.body <- day1 %>%
    group_by(picomp) %>%
    summarise(body = first(TL), speed = median(speed.med), mother = first(mother))

plasma_pal <- viridis::plasma(n = 9)
plasma_rand <- sample(plasma_pal)

body.plot3 <- ggplot(day1.body, aes(x = body, y = speed, color = mother)) +
    geom_point(size = 3) + xlab("Body size on day one") + ylab("Median swimming speed on day one") +
    scale_color_manual(values = plasma_rand) + theme_classic()
```

Fig - growth rate

```r
growth.behav <- size.data %>%
    group_by(week, picomp) %>%
    summarise(speed = median(speed.med), growth = first(growth.rate))

plot <- ggplot(growth.behav, aes(x = growth, y = speed, color = picomp, group = picomp)) +
    stat_smooth(method = "lm", se = F)

plot <- ggplot(growth.behav, aes(x = growth, y = speed, color = picomp, group = picomp)) +
    xlim(0, 0.5) + geom_point()
```

Fig - maternal effects

Is there any evidence that babies from the same mother are more similar in their behavior? Based on the models there is no support for this statistically, but a graphical representation will be nice

```r
plasma_pal <- viridis::plasma(n = 40)
plasma_rand <- sample(plasma_pal)

# one point for every hour in day 1
mom.plot <- ggplot(day1.com, aes(x = mother, y = speed.med)) + geom_boxplot() +
    geom_jitter(width = 0.2, size = 2, aes(color = picomp)) + scale_color_manual(values = plasma_rand) +
    xlab("Mother identity") + ylab("Median speed on day one") + theme_classic() +
    theme(legend.position = "none")

# now we just have one data point per indv for the first day
indv.ave.day1 <- day1 %>%
    group_by(picomp) %>%
    summarise(mother = first(mother), speed = median(speed.med))

mom.plot <- ggplot(indv.ave.day1, aes(x = mother, y = speed)) + geom_boxplot() +
    geom_jitter(width = 0.2) + xlab("Mother identity") + ylab("Median speed on day one") +
    theme_classic()
```

```
indv.ave.week1 <- indv %>%
    filter(obs < 8)


mom.plot <- ggplot(indv.ave.week1, aes(x = mother, y = speed.med)) + geom_boxplot() +
    geom_jitter(width = 0.2) + xlab("Mother identity") + ylab("Median speed on day one") +
    theme_classic()

# now let's look across the whole observation period

plasma_pal9 <- viridis::plasma(n = 9)
plasma_rand <- sample(plasma_pal9)


indv2.ave <- indv %>%
    group_by(picomp) %>%
    arrange(obs) %>%
    mutate(speed.7day = rollmean(speed.med, k = 7, fill = NA))

obs.plot <- ggplot(indv2.ave, aes(x = obs, y = sqrt(speed.7day), group = picomp,
    color = mother)) + geom_line(size = 1) + xlab("Entire observation period") +
    ylab("Median swimming speed") + scale_color_manual(values = plasma_rand) +
    theme_classic() + theme(axis.text.x = element_text(size = 10), axis.title.x = element_text(size = 1[illegible]
    axis.text.y = element_text(size = 10), axis.title.y = element_text(size = 14),
    legend.position = "none")
```
