# Supplementary material for: The emergence and development of behavioral individuality in clonal fish
Source: Nat Commun. 2022 Oct 28;13:6419. doi: 10.1038/s41467-022-34113-y (PMC9616841; doi:10.1038/s41467-022-34113-y)
Supplement: Supplementary file 2 — Reporting Summary [file 41467_2022_34113_MOESM2_ESM.pdf]

nature portfolio

Corresponding author(s): Laskowski, Kate

Last updated by author(s): Oct 3, 2022

Reporting Summary

Nature Portfolio wishes to improve the reproducibility of the work that we publish. This form provides structure for consistency and transparency in reporting. For further information on Nature Portfolio policies, see our Editorial Policies and the Editorial Policy Checklist.

Statistics

For all statistical analyses, confirm that the following items are present in the figure legend, table legend, main text, or Methods section.

n/a	Confirmed	
☐	☒	The exact sample size (*n*) for each experimental group/condition, given as a discrete number and unit of measurement
☐	☒	A statement on whether measurements were taken from distinct samples or whether the same sample was measured repeatedly
☐	☒	The statistical test(s) used AND whether they are one- or two-sided<br>*Only common tests should be described solely by name; describe more complex techniques in the Methods section.*
☐	☒	A description of all covariates tested
☐	☒	A description of any assumptions or corrections, such as tests of normality and adjustment for multiple comparisons
☐	☒	A full description of the statistical parameters including central tendency (e.g. means) or other basic estimates (e.g. regression coefficient) AND variation (e.g. standard deviation) or associated estimates of uncertainty (e.g. confidence intervals)
☐	☒	For null hypothesis testing, the test statistic (e.g. F, t, r) with confidence intervals, effect sizes, degrees of freedom and P value noted<br>*Give P values as exact values whenever suitable.*
☐	☒	For Bayesian analysis, information on the choice of priors and Markov chain Monte Carlo settings
☐	☒	For hierarchical and complex designs, identification of the appropriate level for tests and full reporting of outcomes
☒	☐	Estimates of effect sizes (e.g. Cohen's d, Pearson's r), indicating how they were calculated

Our web collection on statistics for biologists contains articles on many of the points above.

Software and code

Policy information about availability of computer code

Data collection	We used custom scripts (pierecorder, Jolles 2020 JOSS; cited in our main manuscript) to collect the photos from which we then used BioTracker 3.2.1 (freely available online: https://www.igb-berlin.de/en/biotracker) to identify the fish's location (X Y location) within each photo.
Data analysis	Data analysis was conducted using Bayesian hierarchical models (MCMCglmm package) in R v4.2.1. All code to reproduce results is provided as Supplementary Information (Supplemental Note II).

For manuscripts utilizing custom algorithms or software that are central to the research but not yet described in published literature, software must be made available to editors and reviewers. We strongly encourage code deposition in a community repository (e.g. GitHub). See the Nature Portfolio guidelines for submitting code & software for further information.

Data

Policy information about availability of data

All manuscripts must include a data availability statement. This statement should provide the following information, where applicable:

- Accession codes, unique identifiers, or web links for publicly available datasets
- A description of any restrictions on data availability
- For clinical datasets or third party data, please ensure that the statement adheres to our policy

The data generated in this study have been deposited in Dryad Depository [https://doi.org/10.25338/B8XW7G]; An R markdown file recreating the results and figures is included in the Supplemental Information (Supplemental Note II – Code to Reproduce Results).

Field-specific reporting

Please select the one below that is the best fit for your research. If you are not sure, read the appropriate sections before making your selection.

☒ Life sciences ☐ Behavioural & social sciences ☐ Ecological, evolutionary & environmental sciences

For a reference copy of the document with all sections, see nature.com/documents/nr-reporting-summary-flat.pdf

Life sciences study design

All studies must disclose on these points even when the disclosure is negative.

Sample size	We collected complete data for the entire 10-week observation period on a total of 26 individuals. We aimed to have a sample size ~25 individuals as previous behavioral work in our lab demonstrated that with repeated observations of behavior, this sample size was sufficient to estimate among-individual variation in both intercepts and slopes (if it is present). Additionally, published power analyses (e.g. Martin et al. 2011 Methods Ecol Evol) suggest that a total sample size of 200 (e.g. 20 individuals measured 10 times) provides sufficient power to estimate random slopes across individuals (as we did in our study).
Data exclusions	We initially set up a total of 40 individuals in the tracking tanks, to account for the potential loss of individuals throughout the experiment (e.g. due to mortality, camera failures, etc). We were able to achieve complete observations (10-weeks) on a total of 26 individuals. We excluded data from individuals that we did not achieve complete (10-week) observations on (camera malfunctions prevented data collection on four individuals, two individuals jumped into neighboring tanks causing the loss of data of all four individuals as we could not verify their identity; four newborn individuals escaped through holes in the water outlet of the tanks; and four individuals died as newborns). This exclusion was done as we are primarily interested in the estimation of variance components and so we wanted to maintain similar levels of total behavioral variation throughout the entire observation period. To check our results for robustness however, we performed our analyses including these individuals (reported in our Supplemental Note II) and find that our results are the same.
Replication	There is considerable data to demonstrate that individuals of many species, including fish like the Amazon molly, exhibit consistent individual differences in behavior, so our findings here replicate those previous findings. We only set up one set of experimental individuals so there is no independent replication of our findings in this particular study. However, we ensured that our finding that individuals consistently differed in behavior so early in life was robust within our dataset by testing whether individuals consistently differed in all measured behavioral variables, and whether patterns of variation were similar across the first days/weeks of life. Regardless of the behavioral measure, or time frame, we found very strong evidence that individuals consistently differed in behavior and so we have internal replication of our results.
Randomization	Offspring from the same and different mothers were placed randomly into the tracking tanks to minimize systematic variation that might be attributed to physical location.
Blinding	Blinding was not relevant for our study as there were no treatments applied.

Reporting for specific materials, systems and methods

We require information from authors about some types of materials, experimental systems and methods used in many studies. Here, indicate whether each material, system or method listed is relevant to your study. If you are not sure if a list item applies to your research, read the appropriate section before selecting a response.

Materials & experimental systems

n/a	Involved in the study	
☒	☐	Antibodies
☒	☐	Eukaryotic cell lines
☒	☐	Palaeontology and archaeology
☐	☒	Animals and other organisms
☒	☐	Human research participants
☒	☐	Clinical data
☒	☐	Dual use research of concern

Methods

n/a	Involved in the study	
☒	☐	ChIP-seq
☒	☐	Flow cytometry
☒	☐	MRI-based neuroimaging

Animals and other organisms

Policy information about studies involving animals; ARRIVE guidelines recommended for reporting animal research

Laboratory animals	Poecilia formosa (Amazon molly - adult (~1 year old) females were used as breeding mothers; 1 day - 10-week old females were used as the experimental offspring; NB: Amazon mollies are a unisexual, all-female species), Poecilia mexicana (Atlantic mollies - adult males acted as sperm donors for breeding mothers)
Wild animals	NA

Field-collected samples	NA
Ethics oversight	All experimental protocols were in accordance with local and federal guidelines and approved by the Landesamt fur Gesundheit und Soziales (LaGeSo, the appropriate governing body in Berlin Germany) # G-0224/20

Note that full information on the approval of the study protocol must also be provided in the manuscript.
